# One-pot palladium-catalyzed synthesis of sulfonyl fluorides from aryl bromides†
†Electronic supplementary information (ESI) available: Experimental details and supporting characterisation data. See DOI: 10.1039/c6sc03924c



**DOI:** 10.1039/c6sc03924c

**Published:** 2016-10-11

**Authors:** Alyn T. Davies, John M. Curto, Scott W. Bagley, Michael C. Willis

**Affiliations:** a Department of Chemistry , University of Oxford , Chemical Research Laboratory , Mansfield Road , Oxford , OX1 3TA , UK . Email: michael.willis@chem.ox.ac.uk; b CVMET Medicinal Chemistry , Pfizer Inc. , Eastern Point Road , Groton , Connecticut 06340 , USA . Email: scott.w.bagley@pfizer.com

## Abstract

A mild, efficient synthesis of sulfonyl fluorides from aryl and heteroaryl bromides utilizing palladium catalysis is described. The process involves the initial palladium-catalyzed sulfonylation of aryl bromides using DABSO as an SO_2_ source, followed by *in situ* treatment of the resultant sulfinate with the electrophilic fluorine source NFSI. This sequence represents the first general method for the sulfonylation of aryl bromides, and offers a practical, one-pot alternative to previously described syntheses of sulfonyl fluorides, allowing rapid access to these biologically important molecules. Excellent functional group tolerance is demonstrated, with the transformation successfully achieved on a number of active pharmaceutical ingredients, and their precursors. The preparation of peptide-derived sulfonyl fluorides is also demonstrated.

## Abstract 2

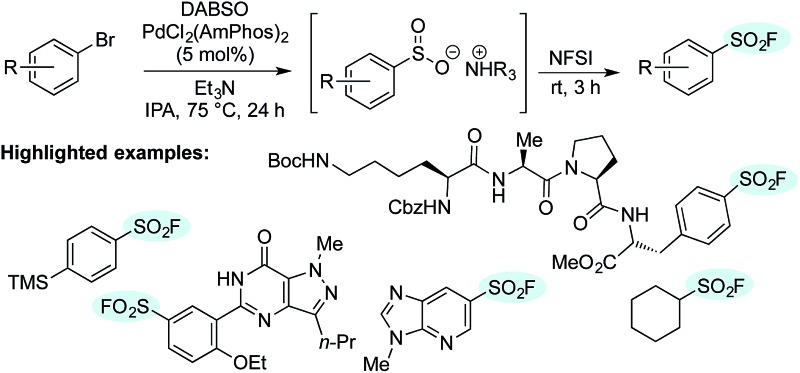
A mild, efficient synthesis of sulfonyl fluorides from aryl and heteroaryl bromides utilizing palladium catalysis is described.

## Introduction

**Fig. 1:**
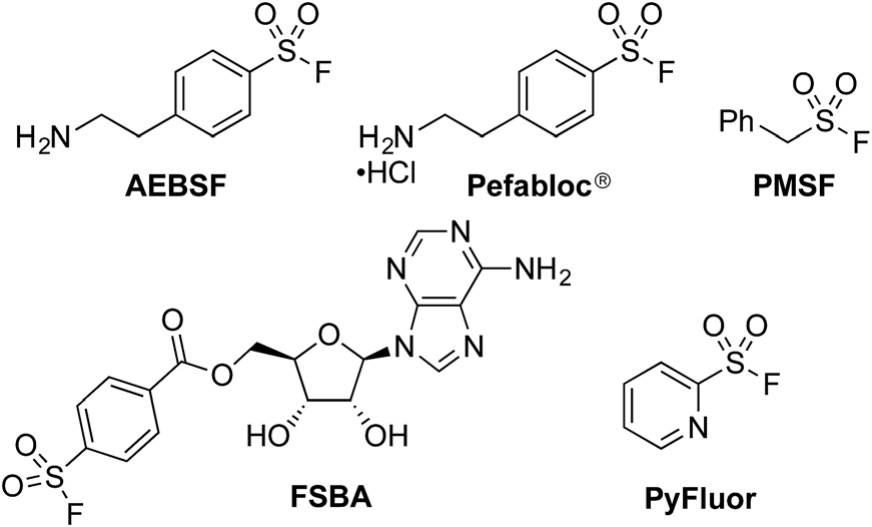
A selection of sulfonyl fluorides used as biological probes and chemical reagents.

The sulfonyl fluoride functional group has become widely adopted throughout the field of chemical biology due to its unique balance between reactivity and stability under physiological conditions.^1^ This is exemplified by the widespread use of (2-aminoethyl)benzenesulfonyl fluoride (AEBSF), its HCl salt Pefabloc®, and phenylmethylsulfonyl fluoride (PMSF) as serine protease inhibitors which are widely used in the preparation of cell lysates (Fig. 1).^2^ Sulfonyl fluorides are also used as chemical biology probes for targeting nucleophilic amino acids;^3^ for example, 5′-fluorosulfonylbenzoyl 5′-adenosine (FSBA), an ATP-binding protein inhibitor, covalently binds to lysine residues.^4^ Sulfonyl fluorides have also shown promising results as fluorinating reagents.^5^ In particular, the recent work of Doyle *et al.* has shown the potential of 2-pyridinesulfonyl fluoride (PyFluor) to act as a selective, thermally stable and inexpensive deoxyfluorination reagent (Fig. 1).^5*c*^

**Scheme 1:**
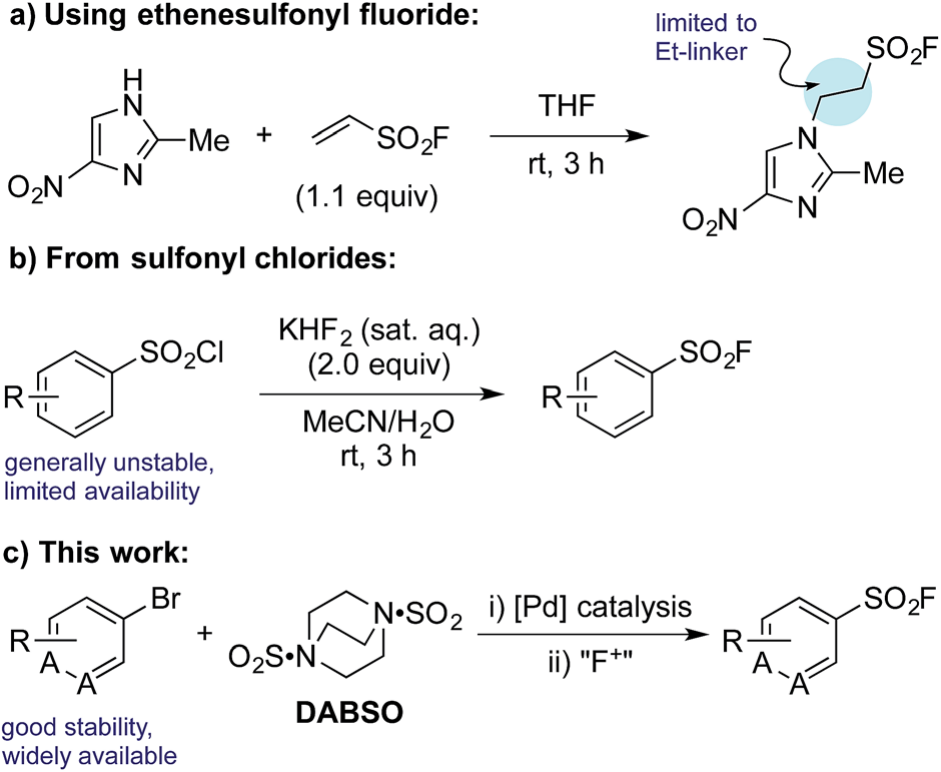
Representative examples of common synthetic routes to sulfonyl fluorides and the approach reported in this Edge Article.

Despite their clear utility, direct synthetic approaches towards sulfonyl fluorides are limited. One commonly utilized method of sulfonyl fluoride synthesis is through 1,4-addition type reactions using the highly reactive Michael acceptor ethenesulfonyl fluoride (ESF).^1,6^ These reactions proceed in excellent yields, however, due to the nature of the reagent, the products are limited to ethyl-linked sulfonyl fluorides (Scheme 1a). Preformed sulfonates can also be converted to the corresponding sulfonyl fluorides in moderate yields by treatment with DAST.^7^ Alternatively, sulfonyl fluorides can be accessed through the corresponding sulfonyl chlorides; combination of the chloride with toxic potassium bifluoride is the most useful process,^1^ although alternative reagents, including KF/18-crown-6,^8^ are also known (Scheme 1b). Unfortunately, sulfonyl chlorides can often be challenging to prepare in their own right, and their inherent high reactivity means that they are poor candidates to survive intact for even a short synthetic sequence. In addition, these methods often prove incompatible with the highly functionalized bioactive molecules to which sulfonyl fluorides are commonly attached, and as such alternative methods for their synthesis are highly desirable. A mild, late stage functionalization approach would address these issues, and in particular would allow significantly greater tolerance towards sensitive functional groups. In this Edge Article we document the realization of such an approach, based on the use of palladium catalysis and readily available aryl- and heteroaryl bromide substrates (Scheme 1c).

## Results and discussion

Our laboratories have shown a particular interest in the chemistry of sulfur-containing compounds, and we have previously described a variety of methods for the synthesis of sulfones, sulfonamides, sulfoxides and many other sulfur-based functional groups.^9^ The driving force behind this chemistry has been the recent emergence of reagents that function as sulfur dioxide surrogates, such as DABSO (1,4-diazabicyclo[2.2.2]octane-bis(sulfur dioxide)) and K_2_S_2_O_5_, which have proven to be a versatile reagents for the introduction of SO_2_.^9o,10^ We set out to exploit our experience with SO_2_ chemistry to develop a synthetic procedure which could alleviate many of the issues associated with sulfonyl fluoride synthesis, focusing on generating a method tolerant to the wide variety of functional groups often present in sulfonyl fluoride-based biological probes. In order to deliver a general method, aryl bromides were targeted as starting materials over the more reactive, but less readily available aryl iodides. Although there are limited reports of the successful use of aryl bromides as substrates in sulfonylation processes, the yields achieved are at best moderate.^11^

Our investigation began by exploring the reactivity of 4-bromobiphenyl under previously reported conditions for sulfinate synthesis from aryl iodides, and pleasingly 33% consumption of **1a** was observed (Table 1, entry 1). Consumption of the aryl bromide **1a** was used as an indication of reactivity; the insolubility of sulfinate **2a** prevented an accurate determination of conversion relative to an internal standard (benzophenone). In order to avoid undesired reactivity, the reaction was monitored for the reduction product, biphenyl **3a**. An evaluation of electron-rich phosphine ligands was subsequently undertaken, as previous work had shown these were the most active in similar systems (entries 2–7).^12^ To our delight, the AmPhos ligand (**L6**) in combination with palladium(ii) acetate showed excellent consumption of the aryl bromide **1a** with negligible amounts of the reduction product being formed (entry 7). By comparison, the Shavnya conditions^11^ using K_2_S_2_O_5_ as the SO_2_ source proved highly active, however, significant quantities of **3a** were observed (entry 8). For convenience in experimental set up, the reaction was attempted with commercial preformed Pd/AmPhos complex and consumption of **1** was increased to 91% (entry 9). With efficient conditions for converting aryl bromides to aryl sulfinates achieved, these conditions were then examined in potential fluorination processes. Fluorination of sulfinate intermediate **2a** proved to be straightforward, and could be accomplished in a one-pot procedure with no change of solvent. Aryl bromide **1a** was subjected to the previously optimized reaction conditions to generate the intermediate sulfinate **2a**, subsequent treatment with 1.5 equivalents of NFSI produced the desired sulfonyl fluoride **4a** in 84% isolated yield. As a result, these conditions were taken forward to evaluate the substrate scope for this transformation.

**Table 1:** Optimization of sulfinate formation from 4-bromobiphenyl^*a*^

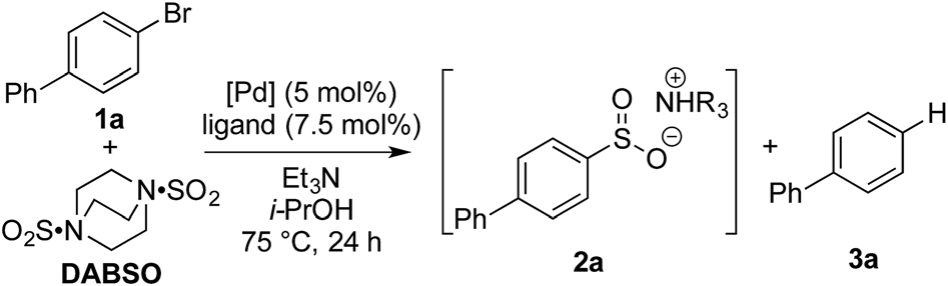				
Entry	[Pd]	Ligand	Consumption of **1** ^*b*^	Reduction to **3** ^*b*^
1	Pd(OAc)_2_	PAd_2_ *n*-Bu	33%	2%
2	Pd(OAc)_2_	**L1**	37%	3%
3	Pd(OAc)_2_	**L2**	28%	2%
4	Pd(OAc)_2_	**L3**	33%	2%
5	Pd(OAc)_2_	**L4**	58%	2%
6	Pd(OAc)_2_	**L5**	77%	10%
7	Pd(OAc)_2_	**L6**	83%	3%
8^*c*^	Pd(OAc)_2_	PPh_3_/1,10-phen	98%	29%
9	PdCl_2_(AmPhos)_2_	n/a	91%	1%
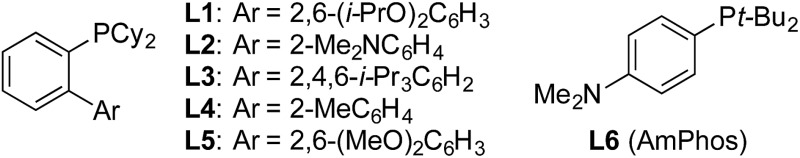				

^*a*^Reaction conditions: aryl bromide (0.4 mmol), [Pd] (5 mol%), ligand (7.5 mol%), DABSO (0.6 equiv.), Et_3_N (3.0 equiv.), *i*-PrOH [0.2 M], 75 °C, 24 h.

^*b*^Consumption of **1a** and reduction to **3a** measured by HPLC relative to benzophenone as an internal standard.

^*c*^K_2_S_2_O_5_ (2.0 equiv.), TBAB (1.1 equiv.), NaCO_2_H (2.2 equiv.) and MeCN (1.4 mL) at 70 °C.

Upon examination the reaction proved tolerant of a wide variety of electron-donating and electron-withdrawing functional groups (Table 2). The *para*-chloro substitution (**4i**) is of particular interest as a handle for further manipulation. A number of sensitive moieties could also be incorporated using this chemistry, such as silane (**4k**), Weinreb amide (**4m**), indole (**4n**), 4-quinazolinone (**4o**) and indazole (**4p**) functionalities. In addition to aryl bromides, the use of aryl iodide substrates is also facilitated, as exemplified by *para*-tolyl sulfonyl fluoride (**4f**).

**Table 2:** Scope of aryl bromides for the palladium-catalyzed synthesis of sulfonyl fluorides^*a*^

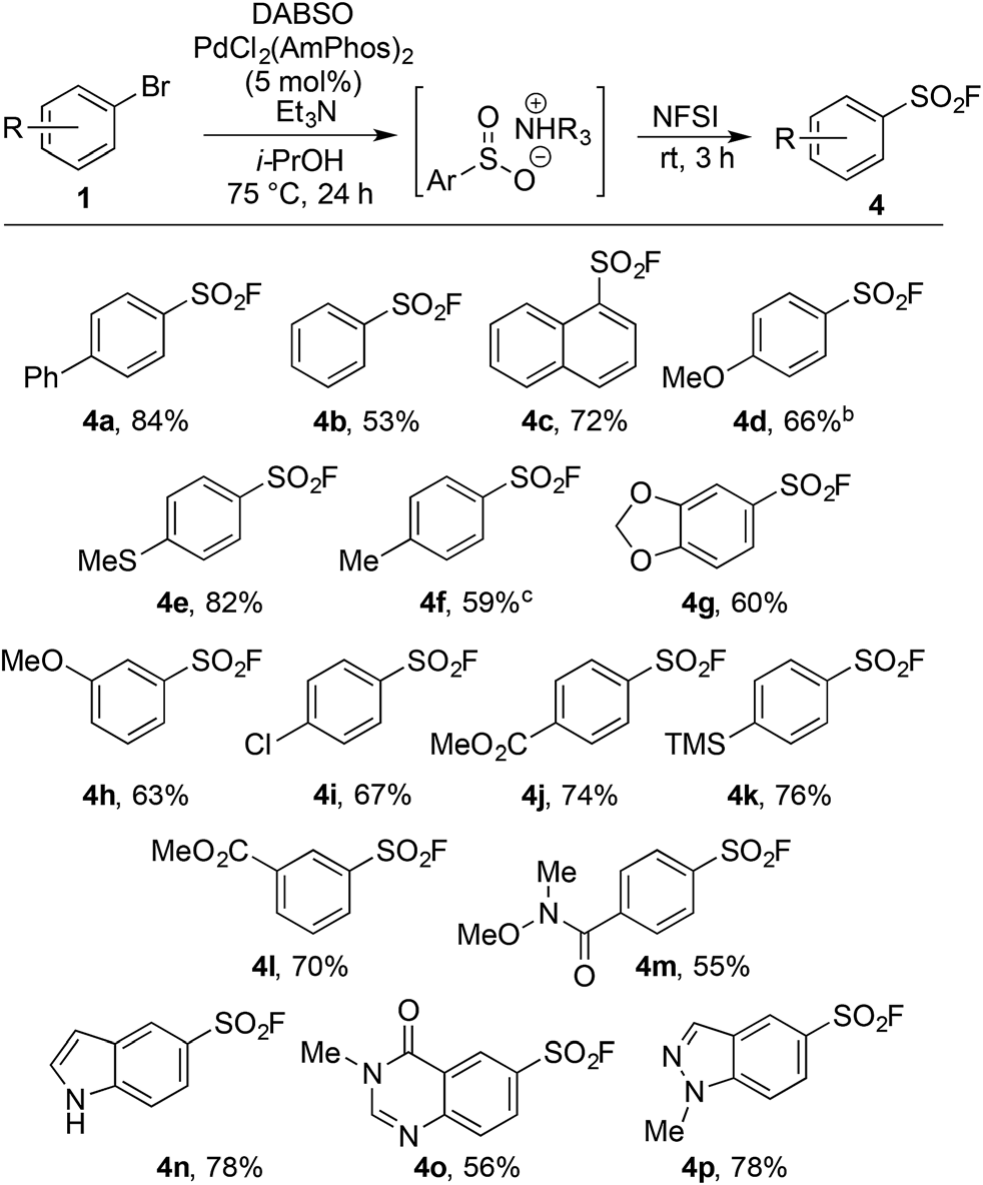

^*a*^Reaction conditions: (i) aryl bromide (0.4 mmol), PdCl_2_(AmPhos)_2_ (5 mol%), DABSO (0.6 equiv.), Et_3_N (3.0 equiv.), *i*-PrOH [0.2 M], 75 °C, 24 h, (ii) NFSI (1.5 equiv.), rt, 3 h. Isolated yields.

^*b*^First step heated under microwave conditions (90 °C, 1 h).

^*c*^Aryl iodide used as starting material.

An on-going challenge in the synthesis of sulfur-containing compounds is the functionalization of heteroaromatics.^9g,h,13^ In the present chemistry hetero-aryl substrates were tolerated when the bromine was positioned on a benzenoid ring (Table 2), however, undertaking the reaction directly on a heteroaromatic ring proved difficult. Bromide **5a**, when subjected to the conditions in Table 2, provided sulfonyl fluoride **6a** in only 15%, with undesired biaryl dimer and palladium black being observed.^12^ Microwave conditions were investigated to achieve higher reaction temperatures in the presence of *i*-PrOH, producing **6a** in 35% at 110 °C. Alternative bases were explored and methyl(dicyclohexyl)amine provided **6a** in 53% (Table 3). The increased steric bulk of methyl(dicyclohexyl)amine is believed to decrease the rate of homo cross-coupling and accelerate the formation of palladium(0).^14^ These conditions proved general for a variety of substituted bromo-pyridines, providing sulfonyl fluorides in adequate yields (Table 3). Non-pyridine heteroaromatics, which are common in drug-like molecules, also formed sulfonyl fluorides efficiently (Table 3, **6e–6h**), yet bromo-imidazoles, -pyrazoles and -pyrimidines proved recalcitrant for sulfonyl fluoride formation.^12^

**Table 3:** Scope of heteroaryl bromides for the palladium-catalyzed synthesis of sulfonyl fluorides^*a*^

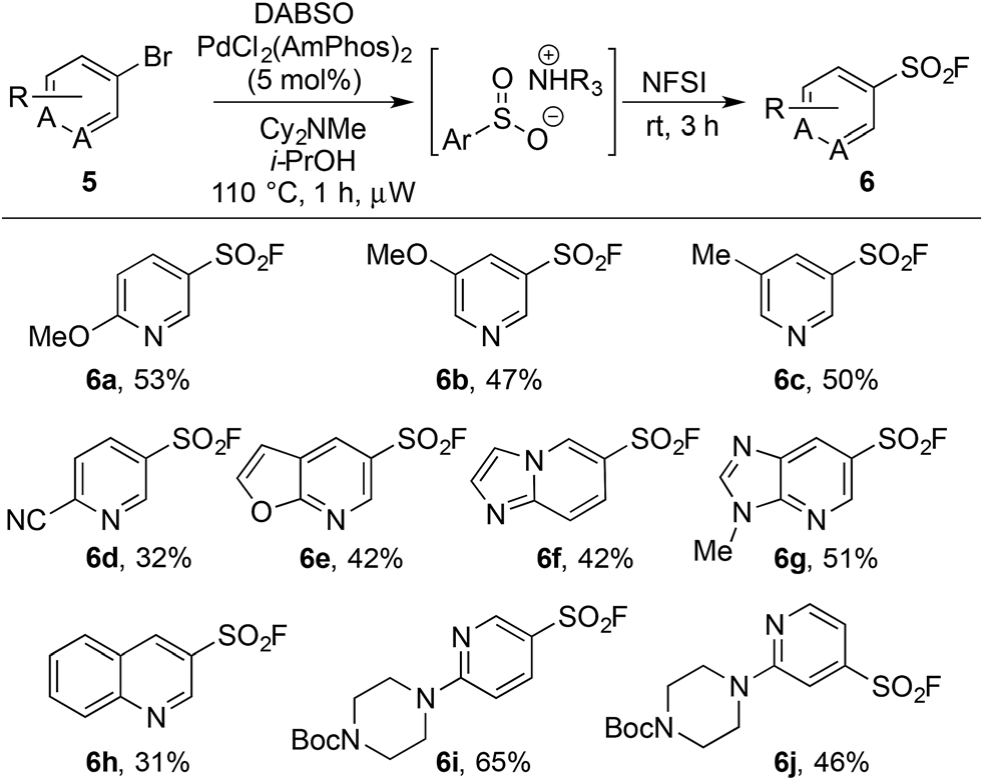

^*a*^Reaction conditions: (i) aryl bromide (0.4 mmol), PdCl_2_(AmPhos)_2_ (5 mol%), DABSO (1.0 equiv.), Cy_2_NMe (3.0 equiv.), *i*-PrOH [0.2 M], 110 °C, 1 h, μW heating. (ii) NFSI (1.5 equiv.), rt, 3 h. Isolated yields.

Encouraged by the scope of this method the sulfonyl fluoride moiety was incorporated into late-stage pharmaceutical intermediates (Table 4). The mild nature of the reaction allowed the straightforward preparation of sulfonyl fluoride derivatives of celecoxib (**4q**), sildenafil (**4r**), *N*-Boc paroxetine (**4s**) and *N*-Boc sertraline (**4t**) in good yields from the appropriately halogenated intermediates. The ability to functionalize highly advanced compounds with a sulfonyl fluoride handle showcases the utility of this methodology to quickly prepare chemical biology probes or templates for further elaboration (*e.g.* sulfonamides),^15^ and would be extremely challenging using existing methods.

**Table 4:** Preparation of sulfonyl fluoride containing pharmaceutical derivatives^*a*^

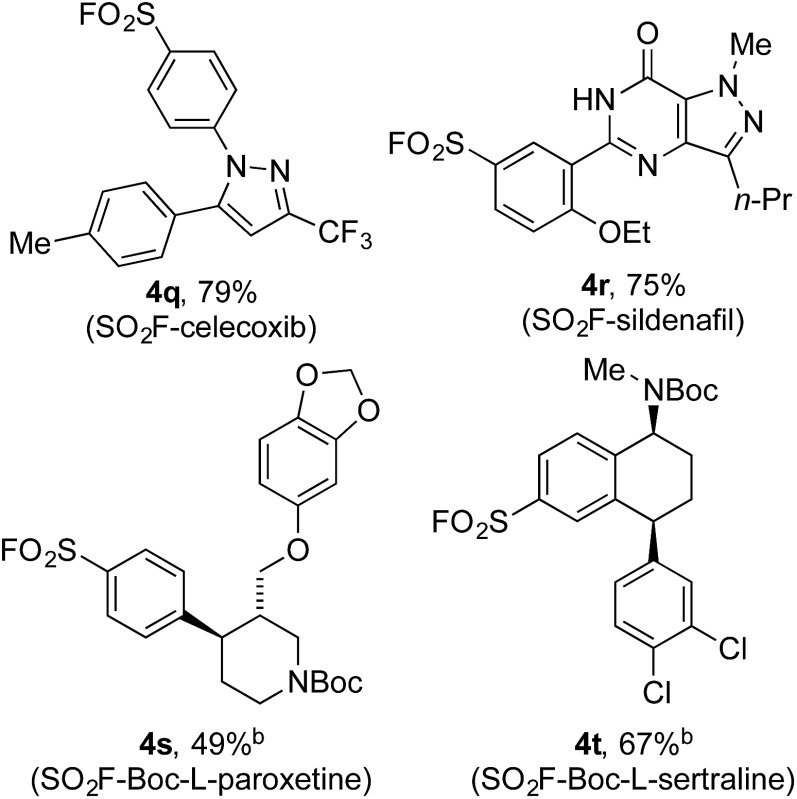

^*a*^Reaction conditions: (i) aryl bromide (0.4 mmol), PdCl_2_(AmPhos)_2_ (5 mol%), DABSO (0.6 equiv.), Et_3_N (3.0 eq.), *i*-PrOH [0.2 M], 75 °C, 16 h. (ii) NFSI (1.5 equiv.), rt, 3 h. Isolated yields.

^*b*^Aryl iodide used as starting material.

**Scheme 2:**
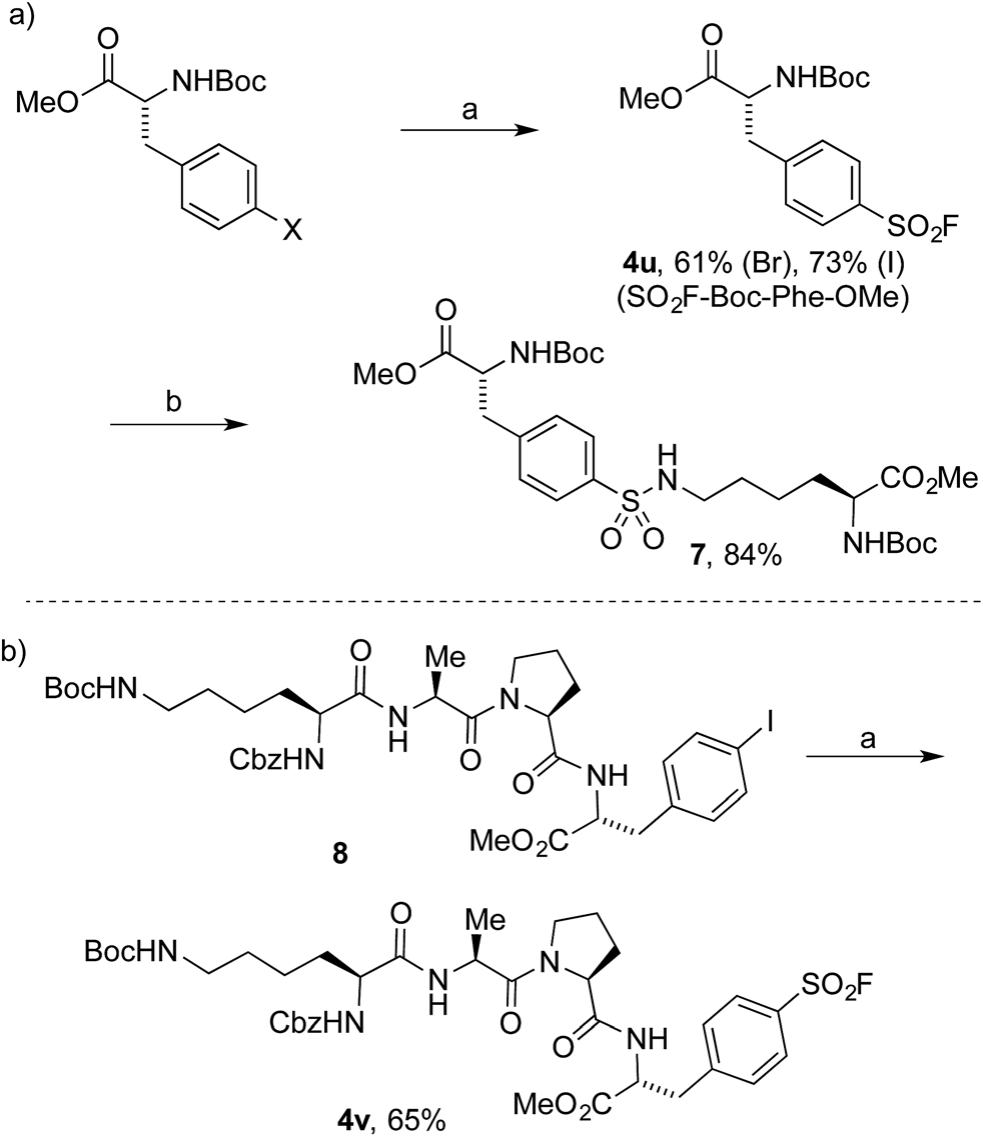
Synthesis of amino acid and peptidic sulfonyl fluorides. (a) Reaction conditions: (i) aryl halide (0.4 mmol), PdCl_2_(AmPhos)_2_ (5 mol%), DABSO (0.6 equiv.), Et_3_N (3.0 equiv.), *i*-PrOH [0.2 M], 75 °C, 16 h. (ii) NFSI (1.5 equiv.), rt, 3 h. (b) *N*-Boc-l-Lys-OMe, *i*-Pr_2_NEt, DMSO, 100 °C, 15 h. Isolated yields.

To date, the synthesis of sulfonyl fluoride labelled amino acids and peptides is limited to incorporation of fragments previously modified with SO_2_–X functionality.^16^ Application of our Pd-catalyzed method to *N*-Boc-l-4-halophenylalanine methyl esters successfully delivered the derivatized analogue **4u** in 61% and 73% yield from the 4-bromo and 4-iodo derivatives, respectively (Scheme 2a). Subsequently, compound **4u** was reacted with *N*-Boc-l-lysine methyl ester to deliver the tail-to-tail linked sulfonamide (**7**) in 84% yield, thus validating the ability of the sulfonyl fluoride moiety to react with nucleophilic residues, central to their use as chemical biology probes. Halogenated tetramer (**8**),^17^ prepared *via* HATU-mediated peptide coupling, was gratifyingly converted to its sulfonyl fluoride derivative (**4v**) in 65% yield (Scheme 2b). The ability to quickly prepare peptide-based sulfonyl fluoride probes could lead to increased exploration of protein binding pockets containing nucleophilic residues such as cysteine, tyrosine, lysine, serine and threonine^3*a*^ or for use as irreversible inhibitors.^18^

The limitations inherent to using ESF to prepare sulfonyl fluorides (Scheme 1) highlight the unmet need for methods to generate diverse alkyl sulfonyl fluorides. Previous work in our groups has shown that organometallic reagents and DABSO can combine to generate sulfinate intermediates.^9j,9l^ This method, complementary to the conditions developed in Table 1, provides an opportunity to access a variety of alkyl and aryl sulfonyl fluorides. Utilizing the fluorination conditions in Table 2, the sulfinate intermediate derived from reaction of DABSO with aryl, benzylic and alkyl Grignard reagents provided sulfonyl fluorides in excellent yield (Table 5). On-going work in our laboratory is focusing on expanding this orthogonal method to additional organometallic substrates.

**Table 5:** Synthesis of sulfonyl fluorides from Grignard reagents^*a*^

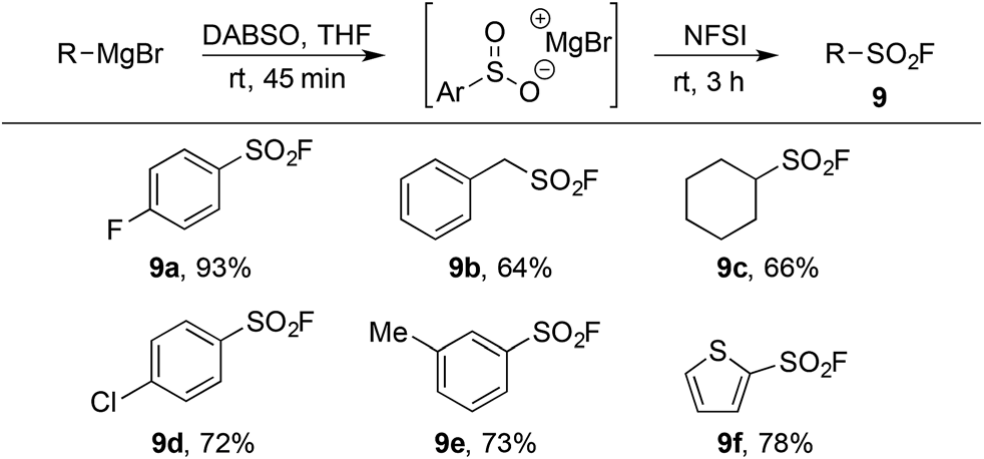

^*a*^Reaction conditions (i) R–MgBr (2.0 mmol), DABSO (1.0 equiv.), THF [0.5 M], rt, 45 min. (ii) NFSI (1.5 equiv.), 0 °C – rt, 3 h. Isolated yields.

## Conclusions

A palladium-catalyzed approach to the synthesis of sulfonyl fluorides from aryl bromides has been developed. The initial step of this sequence represents the first general method for the sulfonylation of aryl bromides. The overall transformation proceeds smoothly on a wide variety of substrates and minor modifications of the reaction conditions allow the use of heteroaryl bromides and organometallic reagents. Of particular note is the tolerance of different functional groups, which allows the transformation to be undertaken on complex molecules, such as active pharmaceutical ingredients, their precursors and peptides. This catalytic, operationally simple method has the ability to expand the scope of sulfonyl fluorides available for investigation as biological probes and has the potential to become a preferred method for complex sulfonyl fluoride synthesis.
