# Supplementary material for: One-pot palladium-catalyzed synthesis of sulfonyl fluorides from aryl bromides
Source: Chem Sci. 2016 Oct 11;8(2):1233–7. doi: 10.1039/c6sc03924c (PMC5369543; doi:10.1039/c6sc03924c)
Supplement: Supplementary file 1 [file SC-008-C6SC03924C-s001.pdf]

One-Pot Palladium-Catalyzed Synthesis of Sulfonyl Fluorides from Aryl Bromides

Alyn T. Davies,[†] John M. Curto,[‡] Scott W. Bagley,*[‡] and Michael C. Willis*[†]

[†] *Department of Chemistry, University of Oxford, Chemistry Research Laboratory, Mansfield Road, Oxford OX1 3TA, United Kingdom*
[‡] *CVMET Medicinal Chemistry, Pfizer, Inc., Eastern Point Road, Groton, Connecticut 06340, United States*

E-mail: michael.willis@chem.ox.ac.uk
E-mail: scott.w.bagley@pfizer.com

Contents

1 Experimental 2
1.1 General information 2
1.2 Optimisation of Palladium-Catalyzed Sulfinate Synthesis with DABSO 3
1.3 Optimisation of Palladium-Catalyzed Sulfinate Synthesis with $K_2S_2O_5$ 4
1.4 Synthesis of Aryl Bromides 4
1.5 Palladium-Catalyzed Synthesis of Sulfonyl Fluorides 5
1.6 Palladium-Catalyzed Synthesis of Pyridyl Sulfonyl Fluorides 12
1.7 Palladium-Catalyzed Synthesis of Sulfonyl Fluoride-Containing Active Pharmaceutical Ingredients 17
1.8 Palladium-Catalyzed Synthesis of Peptidyl Sulfonyl Fluorides 19
1.9 Synthesis of Sulfonyl Fluorides from Grignard Reagents 22
2 References 26
3 NMR Spectra 27
3.1 NMR Spectra of Aryl Bromides 27
3.2 NMR Spectra of Sulfonyl Fluorides Synthesized by Palladium Catalysis 28
3.3 NMR Spectra of Pyridyl Sulfonyl Fluorides Synthesized by Palladium Catalysis 56
3.4 NMR Spectra of Sulfonyl Fluoride-Containing Active Pharmaceutical Ingredients 80
3.5 NMR Spectra of Sulfonyl Fluorides Synthesized from Grignard Reagents 95

1 Experimental

1.1 General information

Chemicals were purchased from Sigma Aldrich, Alfa Aesar, Apollo Scientific or Acros Organics and used without further purification. Pd(AmPhos)$_2$Cl$_2$ precatalyst (dichlorobis[di-*tert*-butyl(4-dimethylaminophenyl)phosphine]palladium(II)) was purchased from Johnson-Matthey (CAS# 887919-35-9, cat # Pd-132).

Solvents were purchased from Sigma Aldrich, Fischer Scientific or Rathburn and unless otherwize mentioned, used without further purification. "Pet. ether" refers to the fraction of petroleum ether boiling in the range 40-60 °C. Anhydrous THF, CH_2Cl_2, CH_3CN, Et_2O, MeOH and toluene were obtained from an in-house solvent drying system having passed through dried alumina columns.

Reactions were performed with continuous magnetic stirring, under an atmosphere of nitrogen (passed through a Drierite® filled tube), unless otherwize stated, and all glassware was dried in an oven (>200 °C, overnight) and allowed to cool under vacuum prior to use. "Reaction tube" refers to a 10 mL CEM microwave reaction vial. Unless stated otherwise, reactions were carried out at room temperature (~23 °C). Cooling between –20 °C and –78 °C was achieved using a dry ice/acetone bath. Flash column chromatography was performed using Apollo Scientific silica gel 60 (particle size 0.040-0.063 mm) with the indicated eluents, or using an automated system with prepackaged columns and gradient elution. Thin layer chromatography (TLC) analysis was carried out on Merck Kieselgel 60 PF254 pre-coated aluminium backed sheets and visualized either by UV fluorescence (254 nm) and/or staining with potassium permanganate solution.

NMR spectra were recored at ambient temperature on a 200 MHz, 400 MHz, 500 MHz or 600 MHz spectrometer. Chemical shifts (δ) reported are in parts per million (ppm) and referenced to the residual solvent peak(s). Coupling constants (*J*) are given in Hertz (Hz). Assignments were made on the basis of chemical shift, coupling constants and comparison with spectra of related compounds. Singal multiplicities are denoted as: s, singlet; d, doublet; t, triplet; q, quartet; quin, quintet; sext, sextet; dd, doublet of doublets; dt, doublet of triplets; m, multiplet; br, broad.

Melting points were measured on a Leica Gallen III hot-stage microscope. Low resolution mass spectra were recored on a Fisons Platform spectrometer (ESI). High-resolution mass spectrometry (HRMS) was performed via atmospheric pressure chemical ionization (APCI) or electrospray ionization (ESI) sources. *m*/*z* Ratios are reported in Daltons; high resolution values are calculated to four decimal places from the molecular formula. Infrared spectra were determined with an internal range of 600-4000 cm^{-1}.

1.2 Optimisation of Palladium-Catalyzed Sulfinate Synthesis with DABSO

Table S1: Optimization of Palladium-Catalyzed Sulfinate Synthesis with DABSO

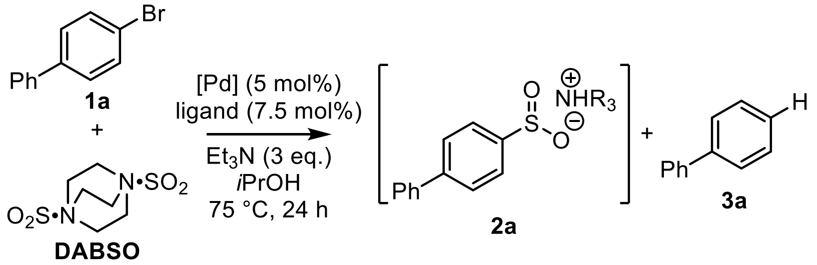

Entry	[Pd]	Ligand	Consumption of 1a	Reduction to 3a
1	$Pd(OAc)_2$	$PdAd_2$*n*-Bu	33%	2%
2	$Pd(OAc)_2$	**L1**	37%	3%
3	$Pd(OAc)_2$	**L2**	28%	2%
4	$Pd(OAc)_2$	**L3**	33%	2%
5	$Pd(OAc)_2$	**L4**	58%	2%
6	$Pd(OAc)_2$	**L5**	77%	10%
7	$Pd(OAc)_2$	**L6**	83%	3%
8	$Pd(OAc)_2$	**L7**	8%	3%
9	$Pd(OAc)_2$	**L8**	20%	10%
10	$Pd(OAc)_2$	**L9**	14%	6%
11	$Pd(OAc)_2$	**L10**	30%	7%
12	$Pd(OAc)_2$	**L11**	10%	0%
13	$Pd(OAc)_2$	**L12**	28%	2%
14	$PdCl_2(AmPhos)_2$	N/A	91%	1%

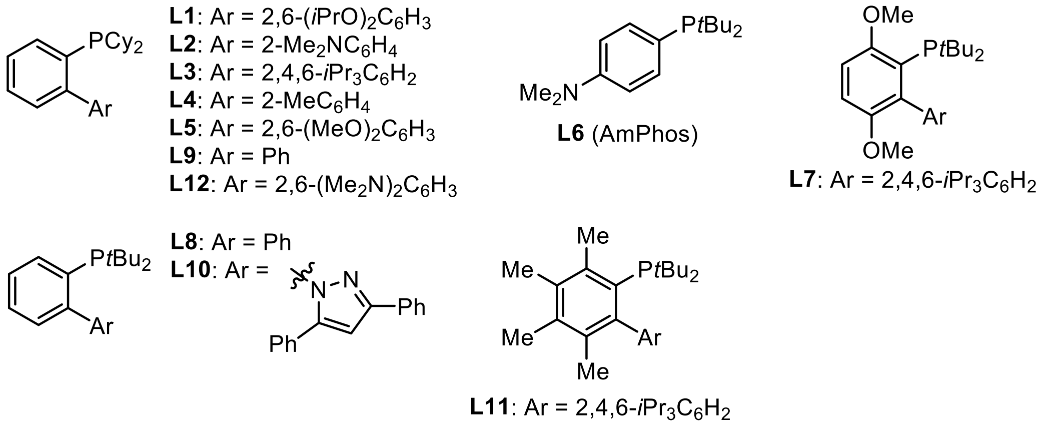

1.3 Optimisation of Palladium-Catalyzed Sulfinate Synthesis with $K_2S_2O_5$

Table S2: Optimization of Palladium-Catalyzed Sulfinate Synthesis with $K_2S_2O_5$

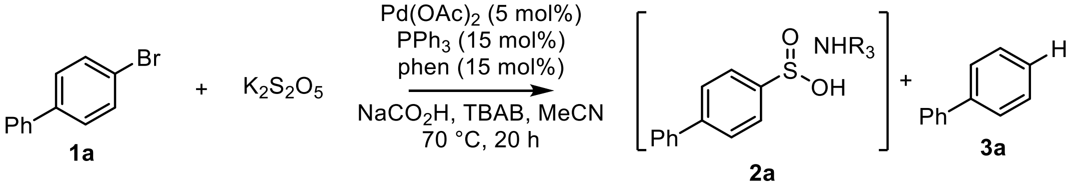

Entry	Variation	Consumption of 1a	Reduction to 3a
1	None	98%	29%
2	IPA instead of MeCN, no $NaCO_2H$	13%	4%
3	No 1,10-phenanthroline	56%	56%
4	No TBAB	71%	23%
5	No PPh_3	2%	2%

1.4 Synthesis of Aryl Bromides

4-Bromo-*N*-methoxy-*N*-methylbenzamide (**S1**)

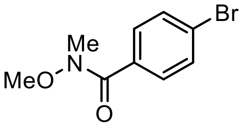

Triethylamine (0.64 mL, 4.6 mmol) was added dropwize to a suspension of *N*,*O*-dimethylhydroxylamine hydrochloride (215 mg, 2.2 mmol) in CH_2Cl_2 (5 mL) at 0 °C. A solution of 4-bromobenzoyl chloride (439 mg, 2.0 mmol) in CH_2Cl_2 (5 mL) was added dropwize at 0 °C and the suspension warmed to room temperature and left stirring for 3 h. The reaction was quenched with sat. aq. $NaHCO_3$ (20 mL) and extracted with CH_2Cl_2. The organic layers were dried ($MgSO_4$), filtered and concentrated *in vacuo* to leave the crude product, which was purified by column chromatography on silica (30% Et_2O in pet. ether) to leave 4-bromo-*N*-methoxy-*N*-methylbenzamide as a colourless oil (414 mg, 1.70 mmol, 85%) with spectroscopic data in accordance with the literature;[1] ^{1}H NMR (400 MHz, $CDCl_3$) δ_H: 7.61 – 7.51 (m, 4H), 3.53 (s, 3H), 3.35 (s, 3H); ^{13}C {^{1}H} NMR (101 MHz, $CDCl_3$) δ_H: 168.7, 132.8, 131.3, 130.0, 125.2, 61.2, 33.5; LRMS (ESI) *m*/*z* 244 ([M(^{79}Br)+H]$^+$), 246 ([M(^{81}Br)+H]$^+$), 266 ([M(^{79}Br)+Na]$^+$), 268 ([M(^{81}Br)+Na]$^+$); HRMS (ESI) found *m*/*z* 243.99701 [M+H]$^+$, $C_9H_{11}{}^{79}BrNO_2$ requires *m*/*z* 243.99677, found *m*/*z* 245.99484 [M+H]$^+$, $C_9H_{11}{}^{81}BrNO_2$ requires *m*/*z* 145.99472.

1.5 Palladium-Catalyzed Synthesis of Sulfonyl Fluorides

General Procedure A: Synthesis of sulfonyl fluorides from aryl bromides, DABSO and NFSI as exemplified by the preparation of 4-phenylbenzenesulfonyl fluoride (**4a**)

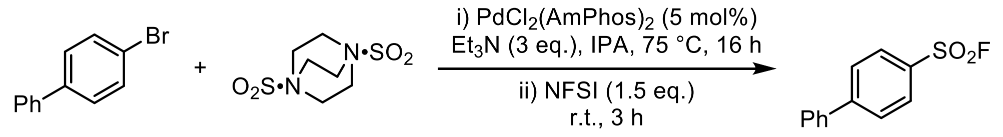

A glass reaction tube was charged with DABSO (58 mg, 0.24 mmol, 0.6 eq.), $PdCl_2(AmPhos)_2$ (14.2 mg, 0.020 mmol, 0.05 eq.) and 4-bromobiphenyl (93 mg, 0.40 mmol, 1.0 eq.), sealed with a rubber septum and evacuated and filled with N_2 four times. Anhydrous isopropanol (1.5 mL) and anhydrous triethylamine (167 µL, 1.2 mmol, 3.0 eq.) were added sequentially through the septum and the reaction mixture stirred under positive pressure of N_2 in a preheated aluminium heating block at 75 °C for 24 h. After cooling to r.t., NFSI (189 mg, 0.6 mmol, 1.5 eq.) was added and the reaction mixture stirred for 3 h until completion. The reaction mixture was concentrated *in vacuo*, then dissolved in EtOAc and filtered through celite. The filtrate was washed with sat. aq. $Na_2S_2O_3$ and brine, dried ($MgSO_4$), filtered and concentrated *in vacuo* to leave the crude product, which was purified by column chromatography on silica (20% CH_2Cl_2 in pet. ether) to leave 4-phenylbenzenesulfonyl fluoride as a white crystalline solid (79.7 mg, 0.337 mmol, 84%); *mp* 81-82 °C; IR ν_{max} (neat)/cm^{-1} 1589, 1479, 1406, 1209, 1186, 1099, 1007; ^{1}H NMR (400 MHz, $CDCl_3$) δ_H: 8.12 – 8.04 (m, 2H), 7.86 – 7.80 (m, 2H), 7.67 – 7.58 (m, 2H), 7.44 – 7.55 (m, 3H); ^{19}F NMR (377 MHz, $CDCl_3$) δ_F: 66.5; ^{13}C {^{1}H} NMR (101 MHz, $CDCl_3$) δ_C: 148.7, 138.5, 131.4 (d, *J* 24.7 Hz), 129.3, 129.2, 129.0, 128.2, 127.5; HRMS (CI) found *m/z* 254.0645 $[M+H]^+$, $C_{12}H_9FO_2S$ requires *m/z* 254.0646.

Benzenesulfonyl fluoride (**4b**)

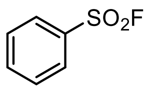

Prepared according to general procedure A using DABSO (58 mg, 0.24 mmol), $PdCl_2(AmPhos)_2$ (14.2 mg, 0.020 mmol), bromobenzene (43 µL, 0.40 mmol), anhydrous isopropanol (1.5 mL), anhydrous triethylamine (167 µL, 1.2 mmol) and NFSI (189 mg, 0.6 mmol). The crude product was purified by column chromatography on silica (10% CH_2Cl_2 in pet. ether) to leave benzenesulfonyl fluoride as a colourless oil (33.6 mg, 0.210 mmol, 53%) with spectroscopic data in accordance with the literature;[2] ^{1}H NMR (400 MHz, $CDCl_3$) δ_H: 8.05 – 8.00 (m, 2H), 7.84 – 7.74 (m, 1H), 7.64 (app tt, *J* 7.7, 1.1 Hz, 2H); ^{19}F NMR (377 MHz, $CDCl_3$) δ_F: 65.9; ^{13}C {^{1}H} NMR (101 MHz, $CDCl_3$) δ_C: 135.6, 133.1 (d, *J* 23.9 Hz), 129.7, 128.4; HRMS not available.

Naphthalene-1-sulfonyl fluoride (**4c**)

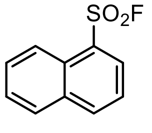

Prepared according to general procedure A using DABSO (58 mg, 0.24 mmol), $PdCl_2(AmPhos)_2$ (14.2 mg, 0.020 mmol), 1-bromonaphthalene (56 μL, 0.40 mmol), anhydrous isopropanol (1.5 mL), anhydrous triethylamine (167 μL, 1.2 mmol) and NFSI (189 mg, 0.6 mmol). The crude product was purified by column chromatography on silica (10% CH_2Cl_2 in pet. ether) to leave naphthalene-1-sulfonyl fluoride as a colourless oil (60.6 mg, 0.288 mmol, 72%); IR ν_{max} (neat)/cm^{-1} 1395, 1364, 1350, 1206, 1194; ^1H NMR (400 MHz, $CDCl_3$) δ_H: 8.54 (dd, *J* 8.4, 2.3 Hz, 1H), 8.36 (app dt, *J* 7.4, 0.9 Hz, 1H), 8.22 (d, *J* 8.3 Hz, 1H), 7.99 (dd, *J* 8.2, 1.3 Hz, 1H), 7.77 (ddd, *J* 8.5, 7.0, 1.4 Hz, 1H), 7.68 (ddd, *J* 8.1, 6.9, 1.1 Hz, 1H), 7.60 (ddd, *J* 8.6, 7.5, 1.5 Hz, 1H); ^{19}F NMR (377 MHz, $CDCl_3$) δ_F: 62.6; ^{13}C {^1H} NMR (101 MHz, $CDCl_3$) δ_C: 137.0, 134.0, 131.10, 131.08, 129.5, 129.2, 128.3, 127.8, 124.2, 124.1; HRMS (CI) found *m*/*z* 228.0489 $[M+NH_4]^+$, $C_{10}H_{11}FNO_2S$ requires *m*/*z* 228.0489.

4-Methoxybenzenesulfonyl fluoride (**4d**)

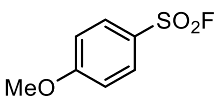

Prepared according to general procedure A using DABSO (58 mg, 0.24 mmol), $PdCl_2(AmPhos)_2$ (14.2 mg, 0.020 mmol), 4-bromoanisole (50 μL, 0.40 mmol), anhydrous isopropanol (1.5 mL), anhydrous triethylamine (167 μL, 1.2 mmol) and NFSI (189 mg, 0.60 mmol), with the first step being heated under microwave irradiation at 90 °C for 1 h. The crude product was purified by column chromatography on silica (20% CH_2Cl_2 in pet. ether) to leave 4-methoxybenzenesulfonyl fluoride as a colourless oil (50.1 mg, 0.263 mmol, 66%) with spectroscopic data in accordance with the literature;[3] ^1H NMR (400 MHz, $CDCl_3$) δ_H: 7.99 – 7.89 (m, 2H), 7.11 – 7.01 (m, 2H), 3.92 (s, 3H); ^{19}F NMR (377 MHz, $CDCl_3$) δ_F: 67.3; ^{13}C {^1H} NMR (101 MHz, $CDCl_3$) δ_C: 165.2, 130.9, 124.1 (d, *J* 24.8 Hz), 114.9, 55.9; HRMS (CI) found *m*/*z* 191.0174 $[M+H]^+$, $C_7H_7FO_3S$ requires *m*/*z* 191.0173.

4-(Methylthio)benzenesulfonyl fluoride (**4e**)

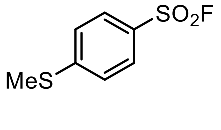

Prepared according to general procedure A using DABSO (58 mg, 0.24 mmol), $PdCl_2(AmPhos)_2$ (14.2 mg, 0.020 mmol), 4-bromothioanisole (93 mg, 0.40 mmol), anhydrous isopropanol (1.5 mL), anhydrous triethylamine (167 μL, 1.2 mmol) and NFSI (189 mg, 0.6 mmol). The crude product was

purified by column chromatography on silica (25% CH_2Cl_2 in pet. ether) to leave 4-(methylthio)benzenesulfonyl fluoride as a white crystalline solid (67.6 mg, 0.328 mmol, 82%); *mp* 62-63 °C; IR ν_{max} (neat)/cm^{-1} 2928, 1576, 1476, 1449, 1391, 1381, 1207, 1107, 1080; ^{1}H NMR (400 MHz, $CDCl_3$) δ_H: 7.91 – 7.83 (m, 2H), 7.33 – 7.42 (m, 2H), 2.55 (s, 3H); ^{19}F NMR (377 MHz, $CDCl_3$) δ_F: 66.9; ^{13}C {^{1}H} NMR (101 MHz, $CDCl_3$) δ_C: 150.3, 128.6, 128.0 (d, *J* 24.7), 125.4, 14.6; HRMS (CI) found *m*/*z* 224.0215 $[M+NH_4]^+$, $C_7H_{11}FNO_2S_2$ requires *m*/*z* 224.0210.

4-Methylbenzenesulfonyl fluoride (**4f**)

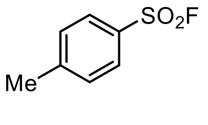

Prepared according to general procedure A using DABSO (58 mg, 0.24 mmol), $PdCl_2(AmPhos)_2$ (14.2 mg, 0.020 mmol), 4-iodotoluene (87 mg, 0.40 mmol), anhydrous isopropanol (1.5 mL), anhydrous triethylamine (167 µL, 1.2 mmol) and NFSI (189 mg, 0.6 mmol). The crude product was purified by column chromatography on silica (10% CH_2Cl_2 in pet. ether) to leave 4-methylbenzenesulfonyl fluoride as a colourless oil (41.2 mg, 0.237 mmol, 58%) with spectroscopic data in accordance with the literature;[2] ^{1}H NMR (400 MHz, $CDCl_3$) δ_H: 7.90 (d, *J* 8.4 Hz, 2H), 7.42 (d, *J* 8.5 Hz, 2H), 2.49 (s, 3H); ^{19}F NMR (377 MHz, $CDCl_3$) δ_F: 66.3; ^{13}C {^{1}H} NMR (101 MHz, $CDCl_3$) δ_C: 147.1, 130.3, 130.1 (d, *J* 24.5 Hz), 128.5, 21.9; HRMS (CI) found *m*/*z* 192.0485 $[M+NH_4]^+$, $C_7H_{11}FNO_2S$ requires *m*/*z* 192.0489.

Benzo[*d*][1,3]dioxole-5-sulfonyl fluoride (**4g**)

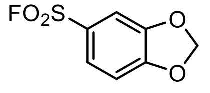

Prepared according to general procedure A using DABSO (58 mg, 0.24 mmol), $PdCl_2(AmPhos)_2$ (14.2 mg, 0.020 mmol), 5-bromobenzo[*d*][1,3]dioxole (48 µL, 0.40 mmol), anhydrous isopropanol (1.5 mL), anhydrous triethylamine (167 µL, 1.2 mmol) and NFSI (189 mg, 0.6 mmol). The crude product was purified by column chromatography on silica (30% CH_2Cl_2 in hexane) to leave benzo[*d*][1,3]dioxole-5-sulfonyl fluoride as a white crystalline solid (48.7 mg, 0.239 mmol, 60%); *mp* 71-72 °C; IR ν_{max} (neat)/cm^{-1} 2922, 1501, 1483, 1433, 1396, 1246, 1202, 1167, 1116, 1060, 1032; ^{1}H NMR (400 MHz, $CDCl_3$) δ_H: 7.61 (ddd, *J* 8.3, 1.9, 0.8, 1H), 7.37 (d, *J* 1.9, 1H), 6.97 (dd, *J* 8.3, 0.9, 1H), 6.16 (s, 2H); ^{19}F NMR (377 MHz, $CDCl_3$) δ_F: 66.9; ^{13}C {^{1}H} NMR (101 MHz, $CDCl_3$) δ_C: 153.9, 148.7, 125.2, 108.8, 108.2, 103.0, 1 × Ar*C* not observed; HRMS (CI) found *m*/*z* 222.0236 $[M+NH_4]^+$, $C_7H_9FNO_4S$ requires *m*/*z* 222.0231.

3-Methoxybenzenesulfonyl fluoride (**4h**)

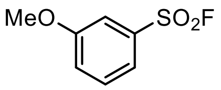

Prepared according to general procedure A using DABSO (58 mg, 0.24 mmol), $PdCl_2(AmPhos)_2$ (14.2 mg, 0.020 mmol), 3-bromoanisole (51 µL, 0.40 mmol), anhydrous isopropanol (1.5 mL), anhydrous triethylamine (167 µL, 1.2 mmol) and NFSI (189 mg, 0.6 mmol). The crude product was purified by column chromatography on silica (20% CH_2Cl_2 in hexane) to leave 3-methoxybenzenesulfonyl fluoride as a colourless oil (47.5 mg, 0.250 mmol, 63%); IR ν_{max} (neat)/cm^{-1} 1601, 1487, 1404, 1327, 1292, 1248, 1206, 1032; ^{1}H NMR (400 MHz, $CDCl_3$) δ_H: 7.60 (ddd, *J* 7.8, 1.8, 0.8, 1H), 7.53 (app td, *J* 8.0, 1.1, 1H), 7.47 (dd, *J* 2.5, 1.7, 1H), 7.28 (app ddt, *J* 8.0, 2.4, 0.7, 1H), 3.89 (s, 3H); ^{19}F NMR (377 MHz, $CDCl_3$) δ_F: 65.6; ^{13}C {^{1}H} NMR (101 MHz, $CDCl_3$) δ_C: 160.2, 134.0 (d, *J* 24.0 Hz), 130.7, 122.2, 120.6, 112.6, 55.9; HRMS (CI) found *m*/*z* 191.0167 $[M+H]^+$, $C_7H_7FO_3S$ requires *m*/*z* 191.0173.

4-Chlorobenzenesulfonyl fluoride (**4i**)

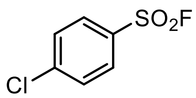

Prepared according to general procedure A using DABSO (58 mg, 0.24 mmol), $PdCl_2(AmPhos)_2$ (14.2 mg, 0.020 mmol), 1-bromo-4-chlorobenzene (77 mg, 0.40 mmol), anhydrous isopropanol (1.5 mL), anhydrous triethylamine (167 µL, 1.2 mmol) and NFSI (189 mg, 0.6 mmol). The crude product was purified by column chromatography on silica (10% CH_2Cl_2 in pet. ether) to leave 4-chlorobenzenesulfonyl fluoride as a white crystalline solid (52.4 mg, 0.268 mmol, 67%) with spectroscopic data in accordance with the literature;[4] *mp* 45-46 °C; ^{1}H NMR (400 MHz, $CDCl_3$) δ_H: 7.99 – 7.93 (m, 2H), 7.65 – 7.59 (m, 2H); ^{19}F NMR (377 MHz, $CDCl_3$) δ_F: 66.5; ^{13}C {^{1}H} NMR (101 MHz, $CDCl_3$) δ_C: 142.7, 131.4 (d, *J* 25.9 Hz), 130.1, 129.9; HRMS (CI) found *m*/*z* 211.9947 $[M+NH_4]^+$, $C_6H_8{}^{35}ClFNO_2S$ requires *m*/*z* 211.9943.

Methyl 4-(fluorosulfonyl)benzoate (**4j**)

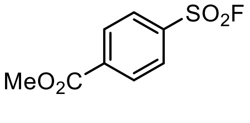

Prepared according to general procedure A using DABSO (58 mg, 0.24 mmol), $PdCl_2(AmPhos)_2$ (14.2 mg, 0.020 mmol), methyl 4-bromobenzoate (86 mg, 0.40 mmol), anhydrous isopropanol (1.5 mL), anhydrous triethylamine (167 µL, 1.2 mmol) and NFSI (189 mg, 0.6 mmol). The crude product

was purified by column chromatography on silica (40% CH_2Cl_2 in hexane) to leave methyl 4-(fluorosulfonyl)benzoate as a white crystalline solid (64.9 mg, 0.297 mmol, 74%); *mp* 86-87 °C; IR ν_{max} (neat)/cm^{-1} 3055, 1726, 1440, 1408, 1277, 1207, 1092, 1014; ^{1}H NMR (400 MHz, $CDCl_3$) δ_H: 8.28 (d, *J* 7.8 Hz, 2H), 8.09 (d, *J* 8.6 Hz, 2H), 3.99 (s, 3H); ^{19}F NMR (377 MHz, $CDCl_3$) δ_F: 65.8; ^{13}C {^{1}H} NMR (101 MHz, $CDCl_3$) δ_C: 164.9, 136.7 (d, *J* 25.4 Hz), 136.5, 130.7, 128.5, 53.0; HRMS not available.

4-(Trimethylsilyl)benzenesulfonyl fluoride (**4k**)

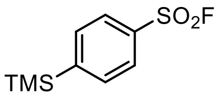

Prepared according to general procedure A using DABSO (58 mg, 0.24 mmol), $PdCl_2(AmPhos)_2$ (14.2 mg, 0.020 mmol), 1-bromo-4-(trimethylsilyl)benzene (78 µL, 0.40 mmol), anhydrous isopropanol (1.5 mL), anhydrous triethylamine (167 µL, 1.2 mmol) and NFSI (189 mg, 0.6 mmol). The crude product was purified by column chromatography on silica (10% CH_2Cl_2 in pet. ether) to leave 4-(trimethylsilyl)benzenesulfonyl fluoride as a colourless oil (70.7 mg, 0.304 mmol, 70%); IR ν_{max} (neat)/cm^{-1} 2959, 1406, 1252, 1213, 1192, 1111, 1090; ^{1}H NMR (400 MHz, $CDCl_3$) δ_H: 7.96 (d, *J* 8.2 Hz, 2H), 7.77 (app dd, *J* 8.4, 1.0 Hz, 2H), 0.33 (s, 9H); ^{19}F NMR (377 MHz, $CDCl_3$) δ_F: 65.9; ^{13}C {^{1}H} NMR (101 MHz, $CDCl_3$) δ_C: 151.3, 134.5, 133.2 (d, *J* 24.3 Hz), 127.2, −1.4; HRMS (CI) found *m*/*z* 250.0734 $[M+NH_4]^+$, $C_9H_{17}FNO_2SSi$ requires *m*/*z* 250.0728.

Methyl 3-(fluorosulfonyl)benzoate (**4l**)

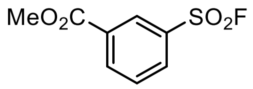

Prepared according to general procedure A using DABSO (58 mg, 0.24 mmol), $PdCl_2(AmPhos)_2$ (14.2 mg, 0.020 mmol), methyl 3-bromobenzoate (86 mg, 0.40 mmol), anhydrous isopropanol (1.5 mL), anhydrous triethylamine (167 µL, 1.2 mmol) and NFSI (189 mg, 0.6 mmol). The crude product was purified by column chromatography on silica (40% CH_2Cl_2 in pet. ether) to leave methyl 3-(fluorosulfonyl)benzoate as a white crystalline solid (61.4 mg, 0.281 mmol, 70%); *mp* 65-66 °C; IR ν_{max} (neat)/cm^{-1} 2963, 1720, 1601, 1445, 1402, 1286, 1273, 1207, 1125, 1082; ^{1}H NMR (400 MHz, $CDCl_3$) δ_H: 8.66 (app t, *J* 1.8, 1H), 8.43 (app dt, *J* 8.0, 1.6, 1H), 8.19 (app dt, *J* 8.0, 1.6, 1H), 7.75 (app tt, *J* 7.9, 0.7, 1H), 3.98 (s, 3H); ^{19}F NMR (377 MHz, $CDCl_3$) δ_F: 66.0; ^{13}C {^{1}H} NMR (101 MHz, $CDCl_3$) δ_C: 164.6, 136.3, 133.7 (d, *J* 26.0), 132.2, 132.1, 130.1, 129.6, 52.9; HRMS (CI) found *m*/*z* 236.0386 $[M+NH_4]^+$, $C_8H_{11}FNO_4S$ requires *m*/*z* 236.0387.

4-(Methoxy(methyl)carbamoyl)benzenesulfonyl fluoride (**4m**)

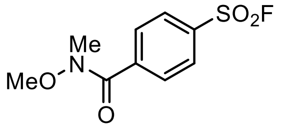

Prepared according to general procedure A using DABSO (58 mg, 0.24 mmol), $PdCl_2(AmPhos)_2$ (14.2 mg, 0.020 mmol), 4-bromo-*N*-methoxy-*N*-methylbenzamide (98 mg, 0.40 mmol), anhydrous isopropanol (1.5 mL), anhydrous triethylamine (167 μL, 1.2 mmol) and NFSI (189 mg, 0.6 mmol). The crude product was purified by column chromatography on silica (100% CH_2Cl_2) to leave 4-(methoxy(methyl)carbamoyl)benzenesulfonyl fluoride as a colourless oil (54.4 mg, 0.220 mmol, 55%); IR ν_{max} (neat)/cm^{-1} 2918, 1645, 1393, 1206, 1092, 1069, 1015; ^{1}H NMR (400 MHz, $CDCl_3$) δ_H: 8.09 – 8.03 (m, 2H), 7.93 – 7.87 (m, 2H), 3.53 (s, 3H), 3.40 (s, 3H); ^{19}F {^{1}H} NMR (377 MHz, $CDCl_3$) δ_F: 65.9; ^{13}C {^{1}H} NMR (101 MHz, $CDCl_3$) δ_C: 167.4, 141.1, 134.4 (d, *J* 25.3 Hz), 129.3, 128.2, 61.5, 33.1; HRMS (CI) found *m*/*z* 248.0391 $[M+H]^+$, $C_9H_{11}FNO_4S$ requires *m*/*z* 248.0387.

Indole-5-sulfonyl fluoride (**4n**)

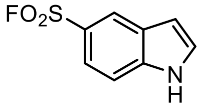

Prepared according to general procedure A using DABSO (58 mg, 0.24 mmol), $PdCl_2(AmPhos)_2$ (14.2 mg, 0.020 mmol), 5-bromoindole (78 mg, 0.40 mmol), anhydrous isopropanol (1.5 mL), anhydrous triethylamine (167 μL, 1.2 mmol) and NFSI (189 mg, 0.6 mmol). The crude product was purified by column chromatography on silica (50% CH_2Cl_2 in pet. ether) to leave indole-5-sulfonyl fluoride as a white crystalline solid (62.4 mg, 0.313 mmol, 78%); *mp* 133-134 °C; IR ν_{max} (neat)/cm^{-1} 3401, 1608, 1381, 1352, 1331, 1218, 1181, 1062; ^{1}H NMR (400 MHz, $CDCl_3$) δ_H: 8.73 (s, 1H), 8.37 (d, *J* 1.8 Hz, 1H), 7.78 (dd, *J* 8.7, 1.9 Hz, 1H), 7.57 (d, *J* 8.7 Hz, 1H), 7.43 (dd, *J* 3.3, 2.4 Hz, 1H), 6.74 (app dt, *J* 3.0, 1.3 Hz, 1H); ^{19}F NMR (377 MHz, $CDCl_3$) δ_F: 68.4; ^{13}C {^{1}H} NMR (101 MHz, $CDCl_3$) δ_C: 139.0, 127.51, 127.45, 123.5 (d, *J* 23.5 Hz), 123.4, 121.2, 112.1, 104.6; HRMS (CI) found *m*/*z* 199.0095 $[M]^+$, $C_8H_6FNO_2S$ requires *m*/*z* 199.0103.

3-Methyl-4-oxo-3,4-dihydroquinazoline-6-sulfonyl fluoride (**4o**)

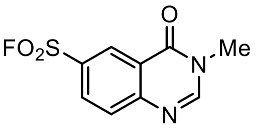

Prepared according to general procedure A using DABSO (58 mg, 0.24 mmol), $PdCl_2(AmPhos)_2$ (14.2 mg, 0.020 mmol), 6-bromo-3-methylquinazolin-4(3*H*)-one (96 mg, 0.40 mmol), anhydrous

isopropanol (1.5 mL), anhydrous triethylamine (167 µL, 1.2 mmol) and NFSI (189 mg, 0.6 mmol). The crude product was purified by column chromatography on silica (10-100% EtOAc in heptane) to provide 3-methyl-4-oxo-3,4-dihydroquinazoline-6-sulfonyl fluoride as a white solid (54 mg, 0.22 mmol, 56%); IR ν_{max} (neat)/cm^{-1} 1687, 1603, 1408, 1209; ^{1}H NMR (400 MHz, DMSO) δ_H: 8.68 (d, *J* 2.0 Hz, 1H), 8.63 (s, 1H), 8.41 (dd, *J* 8.8, 2.1 Hz, 1H), 7.97 (d, *J* 8.6 Hz, 1H), 3.54 (s, 3H); ^{19}F {^{1}H} NMR (377 MHz, DMSO) δ_F: 67.0; ^{13}C {^{1}H} NMR (101 MHz, DMSO) δ_C: 159.6, 153.0, 152.6, 132.0, 129.8, 128.7 (d, *J* 25 Hz), 128.0, 121.9, 33.9; HRMS (ESI$^+$) found *m/z* 243.0227 [M+H]$^+$, $C_9H_8FN_2O_3S$ requires *m/z* 243.0234.

1-Methyl-1*H*-indazole-5-sulfonyl fluoride (**4p**)

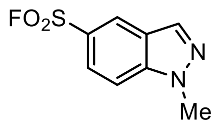

Prepared according to general procedure A using DABSO (58 mg, 0.24 mmol), $PdCl_2(AmPhos)_2$ (14.2 mg, 0.020 mmol), 5-bromo-1-methyl-1*H*-indazole (84 mg, 0.40 mmol), anhydrous isopropanol (1.5 mL), anhydrous triethylamine (167 µL, 1.2 mmol) and NFSI (189 mg, 0.6 mmol). The crude product was purified by column chromatography on silica (10-30% EtOAc in heptane) to leave 1-methyl-1*H*-indazole-5-sulfonyl fluoride as a white solid (67 mg, 0.31 mmol, 78%); IR ν_{max} (neat)/cm^{-1} 1608, 1402, 1212, 1186, 739; ^{1}H NMR (400 MHz, $CDCl_3$) δ_H: 8.52 (s, 1H), 8.21 (s, 1H), 7.94 (dd, *J* 9.1, 1.5 Hz, 1H), 7.60 (d, *J* 8.8 Hz, 1H), 4.17 (s, 3H); ^{19}F {^{1}H} NMR (377 MHz, $CDCl_3$) δ_F: 68.0; ^{13}C {^{1}H} NMR (101 MHz, $CDCl_3$) δ_C: 141.7, 135.0, 124.9, 124.7, 124.6, 123.2, 110.3, 36.0; HRMS (ESI$^+$) found *m/z* 215.0276 [M+H]$^+$, $C_8H_8FN_2O_2S$ requires *m/z* 215.0285.

1.6 Palladium-Catalyzed Synthesis of Pyridyl Sulfonyl Fluorides

Table S3: Optimization of Sulfonyl Fluoride Synthesis from Heteroaromatic Bromides

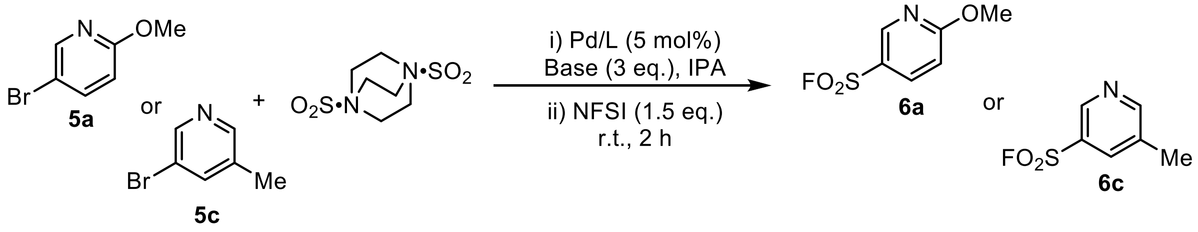

Starting Material (SM)	Pd/L (5 mol %)	Base	Conditions[a]	Yield (%)
**5a**	Pd(AmPhos)$_2$Cl$_2$	NEt$_3$	75 °C, 16 h	15
**5a**	Pd(OAc)2, CataCXium A	NEt$_3$	75 °C, 16 h	<10
**5a**	Pd(AmPhos)$_2$Cl$_2$	NEt$_3$	110 °C, 1.5 h, □W	35
**5a**	Pd(OAc)2, CataCXium A	NEt$_3$	110 °C, 1.5 h, □W	38
**5a**	Pd(AmPhos)$_2$Cl$_2$	Cy$_2$NMe	110 °C, 1.5 h, □W	44
**5a**	Pd(OAc)2, CataCXium A	Cy$_2$NMe	110 °C, 1.5 h, □W	40
**5a**	Pd(AmPhos)$_2$Cl$_2$	Cy$_2$NMe	110 °C, 1.5 h, □W[b]	53
**5a**	Pd(OAc)2, CataCXium A	Cy$_2$NMe	110 °C, 1.5 h, □W[b]	47
**5c**	Pd(AmPhos)$_2$Cl$_2$	NEt$_3$	75 °C, 16 h	0
**5c**	Pd(AmPhos)$_2$Cl$_2$	NEt$_3$	110 °C, 1.5 h, □W	36
**5c**	Pd(AmPhos)$_2$Cl$_2$	NEt$_3$	140 °C, 1.5 h, □W	27
**5c**	Pd(AmPhos)$_2$Cl$_2$	Cy$_2$NMe	110 °C, 1.5 h, □W[b]	50

[a] Reaction conditions: i) Aryl bromide (0.4 mmol), Pd/L (5 mol %), DABSO (0.6 eq), Base (3 eq), *i*PrOH (0.2 M) ii) NFSI (1.5 eq), r.t., 2 h. Isolated yields shown [b] DABSO (1.0 equiv)

General Procedure B: Synthesis of sulfonyl fluorides from heteroaromatic bromides, DABSO and NFSI as exemplified by the preparation of 6-methoxypyridine-3-sulfonyl fluoride (**6a**)

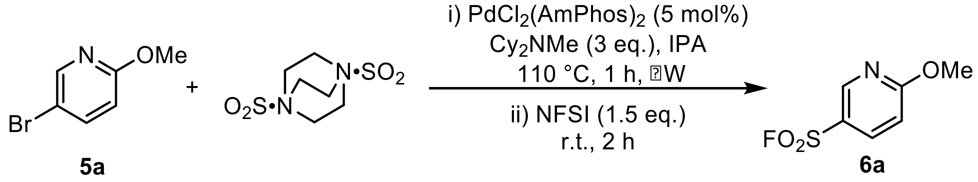

An 8 mL microwave vial was charged with DABSO (96 mg, 0.4 mmol), PdCl$_2$(AmPhos)$_2$ (14.2 mg, 0.020 mmol) and 3-bromo-6-methoxypyridine (89 mg, 0.40 mmol). A solution of *N,N*-dicylclohexylmethylamine (257 µL, 1.2 mmol) in anhydrous isopropanol (1.6 mL) was added, the vial was sealed with a Teflon cap, sparged for 5 minutes with N$_2$ and subject to microwave conditions at 110°C for 1 h. After cooling to r.t., NFSI (189 mg, 0.6 mmol) was added and the reaction mixture stirred for 2 h until completion. The reaction mixture was diluted with H$_2$O (10 mL) and extracted with EtOAc (2 × 15 mL), dried over anhydrous Na$_2$SO$_4$, filtered and concentrated *in vacuo* to leave the crude product, which was purified by column chromatography on silica (0-30% EtOAc in heptane) to provide 6-methoxypyridine-3-sulfonyl fluoride as a white solid (36 mg, 0.19 mmol, 47%); IR ν_{max} (neat)/cm^{-1} 1589, 1486, 1409, 1209; ^{1}H NMR (400 MHz, CDCl$_3$) δ_H: 8.81 (s, 1H), 8.07 (dd, *J* 9.1, 2.6 Hz, 1H), 6.92 (d, *J* 8.8 Hz, 1H), 4.06 (s, 3H); ^{19}F {^{1}H} NMR (377 MHz, CDCl$_3$) δ_F: 68.6; ^{13}C

{^{1}H} NMR (101 MHz, $CDCl_3$) δ_C: 168.2, 149.4, 137.9, 122.5 (d, *J* 25 Hz), 112.1, 54.8; LRMS (AP+) *m*/*z* 192.3 ($[M+H]^+$), HRMS (ESI^+) compound was not stable to ionization.

5-Methoxypyridine-3-sulfonyl fluoride (**6b**)

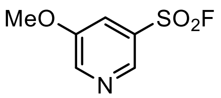

Prepared according to general procedure B using DABSO (96 mg, 0.40 mmol), $PdCl_2(AmPhos)_2$ (14.2 mg, 0.020 mmol), 3-bromo-5-methoxypyridine (89 mg, 0.40 mmol), anhydrous isopropanol (1.6 mL), dicylclohexylmethylamine (257 µL, 1.2 mmol) and NFSI (189 mg, 0.6 mmol). The crude product was purified by column chromatography on silica (0-30% EtOAc in heptane) to provide 5-methoxypyridine-3-sulfonyl fluoride as a white solid (36 mg, 0.19 mmol, 47%); IR ν_{max} (neat)/cm^{-1} 1582, 1425, 1411, 1276, 1210; ^{1}H NMR (400 MHz, $CDCl_3$) δ_H: 8.81 (s, 1H), 8.67 (d, *J* 2.7 Hz, 1H), 7.68 (dd, *J* 2.7, 2.0 Hz, 1H), 3.97 (s, 3H); ^{19}F {^{1}H} NMR (377 MHz, $CDCl_3$) δ_F: 67.9; ^{13}C {^{1}H} NMR (101 MHz, $CDCl_3$) δ_C: 155.6, 145.4, 140.6, 130.2 (d, *J* 24.9 Hz), 118.2, 56.3; HRMS (ESI^+) found *m*/*z* 192.0117 $[M+H]^+$, $C_6H_7FNO_3S$ requires *m*/*z* 192.0125.

5-Methylpyridine-3-sulfonyl fluoride (**6c**)

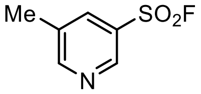

Prepared according to general procedure B using DABSO (96 mg, 0.40 mmol), $PdCl_2(AmPhos)_2$ (14.2 mg, 0.020 mmol), 3-bromo-5-methylpyridine (67 mg, 0.40 mmol), anhydrous isopropanol (1.6 mL), dicylclohexylmethylamine (257 µL, 1.2 mmol) and NFSI (189 mg, 0.6 mmol). The crude product was purified by column chromatography on silica (0-10% EtOAc in heptane) to provide 5-methylpyridine-3-sulfonyl fluoride as a white solid (35 mg, 0.20 mmol, 50%); IR ν_{max} (neat)/cm^{-1} 3030, 1568, 1406, 1208, 776; ^{1}H NMR (400 MHz, $CDCl_3$) δ_H: 9.03 (s, 1H), 8.82 (s, 1H), 8.09 (s, 1H), 2.51 (s, 3H); ^{19}F {^{1}H} NMR (377 MHz, $CDCl_3$) δ_F: 67.9; ^{13}C {^{1}H} NMR (101 MHz, $CDCl_3$) δ_C: 158.6, 146.1, 135.9, 134.9, 129.7 (d, *J* 24.9 Hz), 18.3; LRMS (AP+) *m*/*z* 176.3 ($[M+H]^+$), HRMS (ESI^+) compound was not stable to ionization.

6-Cyanopyridine-3-sulfonyl fluoride (**6d**)

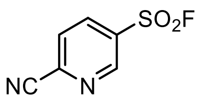

Prepared according to general procedure B using DABSO (96 mg, 0.40 mmol), $PdCl_2(AmPhos)_2$ (14.2 mg, 0.020 mmol), 5-bromopicolinonitrile (89 mg, 0.40 mmol), anhydrous isopropanol (1.6 mL),

dicylclohexylmethylamine (257 µL, 1.2 mmol) and NFSI (189 mg, 0.6 mmol). The crude product was purified by column chromatography on silica (0-30% EtOAc in heptane) to provide 6-cyanopyridine-3-sulfonyl fluoride as a white solid (24 mg, 0.13 mmol, 32%); IR ν_{max} (neat)/cm^{-1} 1576, 1418, 1218, 821, 649; ^{1}H NMR (400 MHz, $CDCl_3$) δ_H: 9.26-9.38 (d, *J* = 2.3 Hz, 1H), 8.49 (dd, *J* = 8.2, 2.3 Hz, 1H), 8.00 (d, *J* = 8.2 Hz, 1H); ^{19}F {^{1}H} NMR (377 MHz, $CDCl_3$) δ_F: 68.4; ^{13}C {^{1}H} NMR (101 MHz, $CDCl_3$) δ_C: 149.8, 139.4, 137.6, 132.8 (d, *J* = 28.6 Hz), 128.6, 115.3; HRMS (ESI$^+$) found *m*/*z* 186.9969 [M+H]$^+$, $C_6H_4FN_2O_2S$ requires *m*/*z* 186.9972.

Furo[2,3-b]pyridine-5-sulfonyl fluoride (**6e**)

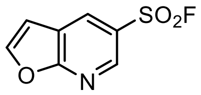

Prepared according to general procedure B using DABSO (96 mg, 0.40 mmol), $PdCl_2(AmPhos)_2$ (14.2 mg, 0.020 mmol), 5-bromofuro[2,3-b]pyridine (79 mg, 0.40 mmol), anhydrous isopropanol (1.6 mL), dicylclohexylmethylamine (257 µL, 1.2 mmol) and NFSI (189 mg, 0.6 mmol). The crude product was purified by column chromatography on silica (0-30% EtOAc in heptane) to provide furo[2,3-b]pyridine-5-sulfonyl fluoride as an white solid (34 mg, 0.17 mmol, 42%); IR ν_{max} (neat)/cm^{-1} 1582, 1410, 1212, 774; ^{1}H NMR (400 MHz, $CDCl_3$) δ_H: 8.99 (d, *J* = 1.2 Hz, 1H), 8.61 (d, *J* = 2.3 Hz, 1H), 7.97 (d, *J* = 2.3 Hz, 1H), 7.01 (d, *J* = 2.3\ Hz, 1H); ^{19}F {^{1}H} NMR (377 MHz, $CDCl_3$) δ_F: 69.8; ^{13}C {^{1}H} NMR (101 MHz, $CDCl_3$) δ_C: 164.4, 148.3, 144.6, 131.2, 126.0 (d, *J* = 26 Hz), 119.9, 106.7; HRMS (ESI$^+$) found *m*/*z* 201.9970 [M+H]$^+$, $C_7H_5FNO_3S$ requires *m*/*z* 201.9969.

Imidazo[1,2-a]pyridine-6-sulfonyl fluoride (**6f**)

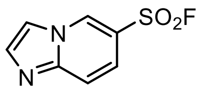

Prepared according to general procedure B using DABSO (96 mg, 0.40 mmol), $PdCl_2(AmPhos)_2$ (14.2 mg, 0.020 mmol), 6-bromoimidazo[1,2-a]pyridine (79 mg, 0.40 mmol), anhydrous isopropanol (1.6 mL), dicylclohexylmethylamine (257 µL, 1.2 mmol) and NFSI (189 mg, 0.6 mmol). The crude product was purified by column chromatography on silica (0-100% EtOAc in heptane) to provide imidazo[1,2-a]pyridine-6-sulfonyl fluoride as an off-white solid (34 mg, 0.17 mmol, 42%); IR ν_{max} (neat)/cm^{-1} 1626, 1439, 1417, 1240, 1198; ^{1}H NMR (400 MHz, $CDCl_3$) δ_H: 9.00 (s, 1H), 7.87 (d, *J* 1.2 Hz, 1H), 7.78-7.85 (m, 2H), 7.57 (dd, *J* 9.8, 2.0 Hz, 1H); ^{19}F {^{1}H} NMR (377 MHz, $CDCl_3$) δ_F: 67.9; ^{13}C {^{1}H} NMR (101 MHz, $CDCl_3$) δ_C: 145.1, 137.1, 130.4, 120.4, 119.8 (d, *J* 29 Hz), 119.1, 114.6; HRMS (ESI$^+$) found *m*/*z* 201.0125 [M+H]$^+$, $C_7H_6FN_2O_2S$ requires *m*/*z* 201.0129.

Imidazo[4,5-b]pyridine-6-sulfonyl fluoride (**6g)**

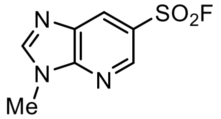

Prepared according to general procedure B using DABSO (96 mg, 0.40 mmol), $PdCl_2(AmPhos)_2$ (14.2 mg, 0.020 mmol), 6-bromo-3-methyl-3H-imidazo[4,5-b]pyridine (85 mg, 0.40 mmol), anhydrous isopropanol (1.6 mL), dicylclohexylmethylamine (257 µL, 1.2 mmol) and NFSI (189 mg, 0.6 mmol). The crude product was purified by column chromatography on silica (100% EtOAc) to provide 3-methyl-3H-imidazo[4,5-b]pyridine-6-sulfonyl fluoride as a white solid (44 mg, 0.20 mmol, 51%); IR ν_{max} (neat)/cm^{-1} 3033, 1602, 1425, 1397, 1252; ^{1}H NMR (400 MHz, $CDCl_3$) δ_H: 9.03 (s, 1H), 8.67 (d, *J* 2.0 Hz, 1H), 8.29 (s, 1H), 4.02 (s, 3H); ^{19}F {^{1}H} NMR (377 MHz, $CDCl_3$) δ_F: 70.3; ^{13}C {^{1}H} NMR (101 MHz, $CDCl_3$) δ_C: 151.1, 148.3, 144.2, 134.3, 128.8, 124.2 (d, *J* 26 Hz), 30.3; HRMS (ESI$^+$) found *m*/*z* 216.0232 [M+H]$^+$, $C_7H_7FN_3O_2S$ requires *m*/*z* 216.0238.

Quinoline-3-sulfonyl fluoride (**6h**)

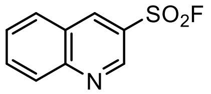

Prepared according to general procedure B using DABSO (96 mg, 0.40 mmol), $PdCl_2(AmPhos)_2$ (14.2 mg, 0.020 mmol), 3-bromoquinoline (83 mg, 0.40 mmol), anhydrous isopropanol (1.6 mL), dicylclohexylmethylamine (257 µL, 1.2 mmol) and NFSI (189 mg, 0.6 mmol). The crude product was purified by column chromatography on silica (0-50% EtOAc in heptane) to provide quinoline-3-sulfonyl fluoride as a white solid (26 mg, 0.12 mmol, 31%); IR ν_{max} (neat)/cm^{-1} 3071, 1618, 1587, 1421, 1210, 788; ^{1}H NMR (400 MHz, $CDCl_3$) δ_H: 9.36 (d, *J* 2.0 Hz, 1H), 8.91 (d, *J* 2.3 Hz, 1H), 8.29 (d, *J* 8.6 Hz, 1H), 7.96-8.13 (m, 2H), 7.74-7.87 (m, 1H); ^{19}F {^{1}H} NMR (377 MHz, $CDCl_3$) δ_F: 68.8; ^{13}C {^{1}H} NMR (101 MHz, $CDCl_3$) δ_C: 150.4, 146.0, 138.9, 134.2, 129.9, 129.4, 129.1, 126.3 (d, *J* 25.7), 125.8; HRMS (ESI$^+$) found *m*/*z* 212.0169 [M+H]$^+$, $C_9H_6FNO_2S$ requires *m*/*z* 212.0176.

tert-Butyl 4-(5-(fluorosulfonyl)pyridin-2-yl)piperazine-1-carboxylate (**6i**)

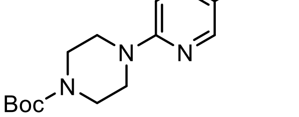

Prepared according to general procedure B using DABSO (96 mg, 0.40 mmol), $PdCl_2(AmPhos)_2$ (14.2 mg, 0.020 mmol), tert-butyl 4-(5-bromopyridin-2-yl)piperazine-1-carboxylate (89 mg, 0.40 mmol), anhydrous isopropanol (1.6 mL), dicylclohexylmethylamine (257 µL, 1.2 mmol) and NFSI

(189 mg, 0.6 mmol). The crude product was purified by column chromatography on silica (0-30% EtOAc in heptane) to provide tert-butyl 4-(5-(fluorosulfonyl)pyridin-2-yl)piperazine-1-carboxylate as a white solid (90 mg, 0.26 mmol, 65%); IR ν_{max} (neat)/cm^{-1} 2978, 1695, 1591, 1404, 1245, 1203; ^{1}H NMR (400 MHz, $CDCl_3$) δ_H: 8.68 (s, 1H), 7.89 (dd, *J* 9.2, 2.5 Hz, 1H), 6.65 (d, *J* 9.4 Hz, 1H), 3.71-3.86 (m, 4H), 3.46-3.65 (m, 4H), 1.49 (s, 9H); ^{19}F {^{1}H} NMR (377 MHz, $CDCl_3$) δ_F: 69.1; ^{13}C {^{1}H} NMR (101 MHz, $CDCl_3$) δ_C: 160.8, 154.5, 150.2, 137.1, 115.9 (d, *J* 24.9 Hz), 105.3, 80.4, 44.3, 28.4; HRMS (ESI$^+$) found *m/z* 368.1045 $[M+Na]^+$, $C_{14}H_{20}FN_3NaO_4S$ requires *m/z* 368.1051.

tert-Butyl 4-(4-(fluorosulfonyl)pyridin-2-yl)piperazine-1-carboxylate (**6j**)

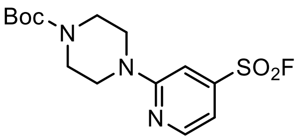

Prepared according to general procedure B using DABSO (96 mg, 0.40 mmol), $PdCl_2(AmPhos)_2$ (14.2 mg, 0.020 mmol), tert-butyl 4-(4-bromopyridin-2-yl)piperazine-1-carboxylate (89 mg, 0.40 mmol), anhydrous isopropanol (1.6 mL), dicylclohexylmethylamine (257 µL, 1.2 mmol) and NFSI (189 mg, 0.6 mmol). The crude product was purified by column chromatography on silica (0-30% EtOAc in heptane) to provide tert-butyl 4-(4-(fluorosulfonyl)pyridin-2-yl)piperazine-1-carboxylate as a white solid (63 mg, 0.18 mmol, 46%); IR ν_{max} (neat)/cm^{-1} 2978, 1694, 1589, 1413, 1242; ^{1}H NMR (400 MHz, $CDCl_3$) δ_H: 8.44 (d, *J* 5.1 Hz, 1H), 7.04-7.10 (m, 2H), 3.62-3.76 (m, 4H), 3.45-3.62 (m, 4H), 1.50 (s, 9H); ^{19}F {^{1}H} NMR (377 MHz, $CDCl_3$) δ_F: 63.1; ^{13}C {^{1}H} NMR (101 MHz, $CDCl_3$) δ_C: 159.1, 154.6, 150.3, 142.6 (d, *J* 26 Hz), 108.8, 104.1, 80.3, 44.6, 28.4; HRMS (ESI$^+$) found *m/z* 368.1037 $[M+Na]^+$, $C_{14}H_{20}FN_3NaO_4S$ requires *m/z* 368.1051.

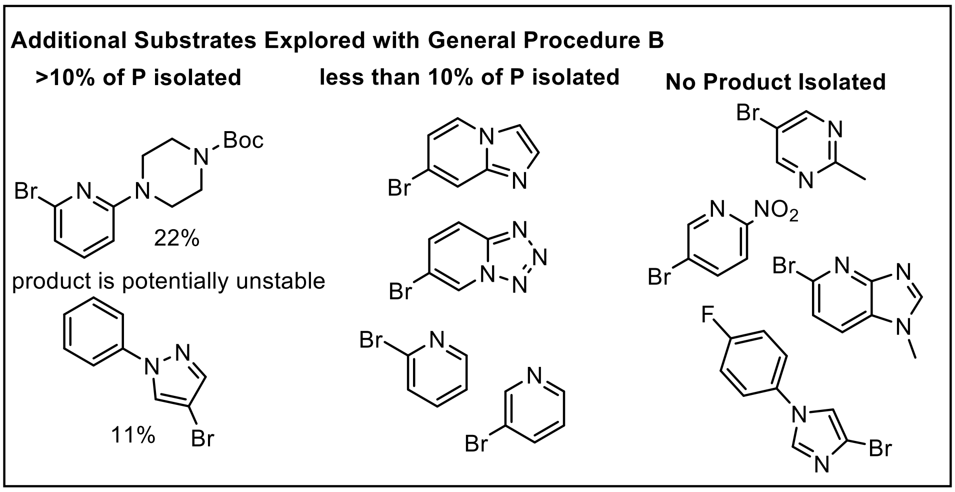

1.7 Palladium-Catalyzed Synthesis of Sulfonyl Fluoride-Containing Active Pharmaceutical Ingredients

4-(5-(*p*-Tolyl)-3-(trifluoromethyl)-1*H*-pyrazol-1-yl)benzenesulfonyl fluoride (4-SO_2F-celecoxib, **4q**)

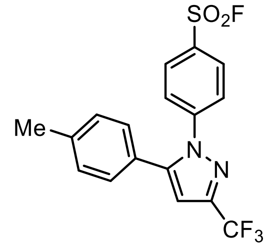

Prepared according to general procedure A using DABSO (58 mg, 0.24 mmol), $PdCl_2(AmPhos)_2$ (14.2 mg, 0.020 mmol), 1-(4-bromophenyl)-5-(*p*-tolyl)-3-(trifluoromethyl)-1*H*-pyrazole (4-bromo celecoxib, 152 mg, 0.40 mmol), anhydrous isopropanol (1.5 mL), anhydrous triethylamine (167 µL, 1.2 mmol) and NFSI (189 mg, 0.6 mmol). The crude product was purified by column chromatography on silica (0-40% EtOAc in heptane) to leave 4-(5-(*p*-tolyl)-3-(trifluoromethyl)-1*H*-pyrazol-1-yl)benzenesulfonyl fluoride (4-SO_2F-celecoxib) as a white solid (116 mg, 0.302 mmol, 75%); IR ν_{max} (neat)/cm^{-1} 1597, 1500, 1471, 1417, 1372, 1237, 1212, 1161, 1135, 1096, 973, 784, 628; ^{1}H NMR (600 MHz, $CDCl_3$) δ_H 8.00 (d, J=8.80 Hz, 2H), 7.61 (d, J=8.80 Hz, 2H), 7.23 (d, J=8.22 Hz, 2H), 7.15 (d, J=8.22 Hz, 2H), 6.77 (s, 1H), 2.42 (s, 3H); ^{13}C NMR (151 MHz, $CDCl_3$) δ_C ppm 145.5, 144.8, 144.7 (q, *J*=39.2 Hz), 140.2, 131.9 (d, *J*=26.1 Hz), 129.9, 129.5, 128.7, 125.53, 125.49, 120.9 (q, *J*=269.0 Hz), 107.0, 21.3; ^{19}F NMR (376 MHz, $CDCl_3$) δ_F: 66.4 (s, 1F), −62.6 (s, 3F); HRMS (ESI$^+$) found *m*/*z* 385.0620 $[M+H]^+$, $C_{17}H_{13}F_4N_2O_2S$ requires *m*/*z* 385.0628.

4-Ethoxy-3-(1-methyl-7-oxo-3-propyl-6,7-dihydro-1*H*-pyrazolo[4,3-d]pyrimidin-5-yl)benzenesulfonyl fluoride (5-SO_2F-sildenafil, **4r**)

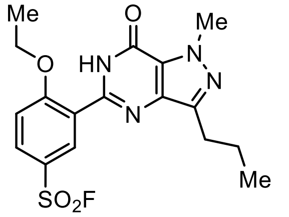

Prepared according to general procedure A using DABSO (58 mg, 0.24 mmol), $PdCl_2(AmPhos)_2$ (14.2 mg, 0.020 mmol), 5-(5-bromo-2-ethoxyphenyl)-1-methyl-3-propyl-1,6-dihydro-7*H*-pyrazolo[4,3-d]pyrimidin-7-one (5-bromo sildenafil, 157 mg, 0.40 mmol), anhydrous isopropanol (1.5 mL), anhydrous triethylamine (167 µL, 1.2 mmol) and NFSI (189 mg, 0.6 mmol). The crude product was purified by column chromatography on silica (0-40 % EtOAc in heptane) to leave 4-ethoxy-3-(1-methyl-7-oxo-3-propyl-6,7-dihydro-1*H*-pyrazolo[4,3-d]pyrimidin-5-yl)benzenesulfonyl fluoride (5-SO_2F-sildenafil) as a white solid (118 mg, 0.299 mmol, 75%); IR ν_{max} (neat)/cm^{-1} 3329, 2961, 1682,

1534, 1490, 1408, 1280, 1207, 1158, 770, 593; ^{1}H NMR (600 MHz, $CDCl_3$) δ_H: 10.77 (br. s., 1 H), 9.08 (d, *J*=2.3 Hz, 1 H), 8.08 (dd, *J*=8.8, 2.3 Hz, 1 H), 7.25 (d, *J*=9.4 Hz, 1 H), 4.44 (q, *J*=7.0 Hz, 2 H), 4.28 (s, 3 H), 2.95 (t, *J*=7.6 Hz, 2 H), 1.87 (sxt, *J*=7.5 Hz, 2 H), 1.67 (t, *J*=7.0 Hz, 3 H), 1.04 (t, *J*=7.3 Hz, 3 H); ^{13}C NMR (151 MHz, $CDCl_3$) δ_C 161.1, 153.5, 147.2, 145.6, 138.2, 132.4, 132.2, 126.1 (d, J=26.1 Hz), 124.5, 122.0, 113.6, 66.5, 38.2, 27.6, 22.3, 14.4, 14.0; ^{19}F NMR (376 MHz, $CDCl_3$) δ_F: 66.9; HRMS (ESI$^+$) found *m/z* 395.1189 [M+H]$^+$, $C_{17}H_{20}FN_4O_4S$ requires *m/z* 395.1184.

tert-Butyl (3*S*,4*R*)-3-((benzo[d][1,3]dioxol-5-yloxy)methyl)-4-(4-(fluorosulfonyl)phenyl)piperidine-1-carboxylate (*N*-Boc-6-SO_2F-paroxetine, **4s**)

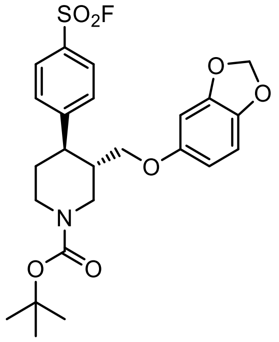

Prepared according to general procedure A using DABSO (58 mg, 0.24 mmol), $PdCl_2(AmPhos)_2$ (14.2 mg, 0.020 mmol), *tert*-butyl (3*S*,4*R*)-3-((benzo[d][1,3]dioxol-5-yloxy)methyl)-4-(4-iodophenyl)piperidine-1-carboxylate (*N*-Boc-4-iodo paroxetine, 215 mg, 0.40 mmol), anhydrous isopropanol (1.5 mL), anhydrous triethylamine (167 µL, 1.2 mmol) and NFSI (189 mg, 0.6 mmol). The crude product was purified by column chromatography on silica (0-40 % EtOAc in heptane) to leave *tert*-butyl (3*S*,4*R*)-3-((benzo[d][1,3]dioxol-5-yloxy)methyl)-4-(4-(fluorosulfonyl)phenyl)piperidine-1-carboxylate (*N*-Boc-6-SO_2F-paroxetine) as a white solid (95 mg, 0.192 mmol, 48%); IR ν_{max} (neat)/cm^{-1} 2980, 1689, 1504, 1489, 1471, 1420, 1407, 1239, 1215, 1185, 1135, 769; ^{1}H NMR (600 MHz, $CDCl_3$) δ_H 7.94 (d, *J*=8.2 Hz, 2 H), 7.46 (d, *J*=8.2 Hz, 2 H), 6.64 (d, *J*=8.8 Hz, 1 H), 6.33 (d, *J*=2.3 Hz, 1 H), 6.12 (dd, *J*=8.2, 2.3 Hz, 1 H), 5.90 (s, 2 H), 4.43 (br. s., 1 H), 4.29 (br. s., 1 H), 3.61 (dd, *J*=9.4, 2.9 Hz, 1 H), 3.46 (dd, *J*=9.4, 5.9 Hz, 1 H), 2.79 - 3.01 (m, 3 H), 2.10 (br. s., 1 H), 1.82 - 1.89 (m, 1 H), 1.76 (qd, *J*=12.5, 3.5 Hz, 1 H), 1.62 (m, *J*=12.3 Hz, 1 H), 1.51 (s, 9 H); ^{13}C NMR (151 MHz, $CDCl_3$) δ_C 154.7, 153.9, 152.4, 148.3, 141.9, 131.4 (d, *J*=25.1 Hz), 128.9, 128.8, 107.9, 105.4, 101.2, 97.9, 80.0, 68.4, 45.0, 41.6, 33.4, 28.4; ^{19}F NMR (376 MHz, $CDCl_3$) δ_F: 66.1; HRMS (ESI$^+$) found *m/z* 494.1645 [M+H]$^+$, $C_{24}H_{29}FNO_7S$ requires *m/z* 494.1643.

tert-Butyl ((1*S*,4*S*)-4-(3,4-dichlorophenyl)-6-(fluorosulfonyl)-1,2,3,4-tetrahydronaphthalen-1-yl)(methyl)carbamate (*N*-Boc-6-SO_2F-sertraline, **4t**)

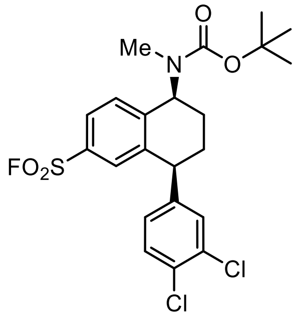

Prepared according to general procedure A using DABSO (58 mg, 0.24 mmol), $PdCl_2(AmPhos)_2$ (14.2 mg, 0.020 mmol), *tert*-butyl ((1*S*,4*S*)-4-(3,4-dichlorophenyl)-6-iodo-1,2,3,4-tetrahydronaphthalen-1-yl)(methyl)carbamate (*N*-Boc-6-iodo sertraline, 213 mg, 0.4 mmol), anhydrous isopropanol (1.5 mL), anhydrous triethylamine (167 µL, 1.2 mmol) and NFSI (189 mg, 0.6 mmol). The crude product was purified by column chromatography on silica (0-40 % EtOAc in heptane) to leave *tert*-butyl ((1*S*,4*S*)-4-(3,4-dichlorophenyl)-6-(fluorosulfonyl)-1,2,3,4-tetrahydronaphthalen-1-yl)(methyl)carbamate (*N*-Boc-6-SO_2F-sertraline) as a white solid (131 mg, 0.268 mmol, 67%); IR ν_{max} (neat)/cm^{-1} 2980, 1686, 1470, 1412, 1391, 1367, 1345, 1321, 1217, 1161, 1140; ^{1}H NMR (400 MHz, $CDCl_3$) δ_H: δ 7.89 (d, *J*=7.8 Hz, 1H), 7.63 (s., 1H), 7.49 (d, *J*=7.8 Hz, 1H), 7.38 (d, *J*=8.2 Hz, 1H), 7.08 (s, 1H), 6.77 (d, *J*=7.8 Hz, 1H), 5.20-5.57 (m, 1H), 4.30 (d, *J*=2.9 Hz, 1H), 2.68 (s, 3H), 2.22-2.41 (m, 1H), 2.07 (d, *J*=12.3 Hz, 1H), 1.83 (dd, *J*= 7.6, 4.1 Hz, 2H), 1.54 (s, 9H); ^{13}C NMR (151 MHz, $CDCl_3$) δ_C 156.3, 146.0, 145.4, 140.3, 132.8, 131.8 (d, *J* = 26.1 Hz), 130.9, 130.7, 130.5, 130.4, 128.7, 127.8, 126.7, 80.5, 54.6, 43.0, 30.2, 29.8, 28.4, 20.8; ^{19}F NMR (376 MHz, $CDCl_3$) δ_F: 66.1; HRMS (ESI$^+$) found *m*/*z* 488.0847 [M+H]$^+$, $C_{22}H_{25}Cl_2FNO_4S$ requires *m*/*z* 488.0860.

1.8 Palladium-Catalyzed Synthesis of Peptidyl Sulfonyl Fluorides

N-Boc-4-Sulfonylfluoride phenylalanine methyl ester (**4u**)

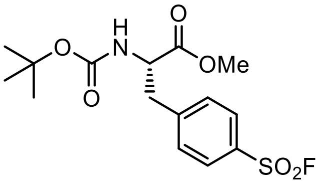

Prepared according to general procedure A using DABSO (58 mg, 0.24 mmol), $PdCl_2(AmPhos)_2$ (14.2 mg, 0.020 mmol), *N*-Boc-4-iodo phenylalanine methyl ester (162 mg, 0.40 mmol), anhydrous isopropanol (1.5 mL), anhydrous triethylamine (167 µL, 1.2 mmol) and NFSI (189 mg, 0.6 mmol). The crude product was purified by column chromatography on silica (0-40 % EtOAc in heptane) to leave *N*-Boc-4-sulfonylfluoride phenylalanine methyl ester as a white solid (105 mg, 0.291 mmol,

73%); IR ν_{max} (neat)/cm^{-1} 1744, 1711, 1405, 1366, 1280, 1212, 1161, 1056, 1019, 767, 630, 594; ^{1}H NMR (400 MHz, $CDCl_3$) δ_H: 7.95 (d, *J* 8.2 Hz, 2H), 7.42 (d, *J* 8.2 Hz, 2H), 5.06 (d, *J* 7.4 Hz, 1H), 4.66 (d, *J* 6.2 Hz, 1H), 3.76 (s, 3H), 3.31 (dd, *J* 13.9, 5.7 Hz, 1H), 3.13 (dd, *J* 13.5, 6.4 Hz, 1H), 1.42 (s, 9H); ^{13}C NMR (101 MHz, $CDCl_3$) δ_C: 171.5, 154.9, 145.2, 131.7, 131.5, 130.6, 128.5, 100.0, 80.4, 54.0, 52.6, 38.6, 28.2; ^{19}F NMR (376 MHz, $CDCl_3$) δ_F: 66.1; HRMS (ESI$^+$) found *m/z* 384.0879 $[M+Na]^+$, $C_{15}H_{20}FNNaO_6S$ requires *m/z* 384.0888.

Additionally, compound **4u** was prepared from *N*-Boc-4-bromo phenylalanine methyl ester according to general procedure A using DABSO (58 mg, 0.24 mmol), $PdCl_2(AmPhos)_2$ (14.2 mg, 0.020 mmol), *N*-Boc-4-bromo phenylalanine methyl ester (144 mg, 0.40 mmol), anhydrous isopropanol (1.5 mL), anhydrous triethylamine (167 μL, 1.2 mmol) and NFSI (189 mg, 0.6 mmol). The crude product was purified by column chromatography on silica (0-40 % EtOAc in heptane) to leave *N*-Boc-4-sulfonylfluoride phenylalanine methyl ester as a white solid (105 mg, 0.291 mmol, 61%). The spectra matched the desired product **4u** as described above.

Methyl N^2-(*tert*-butoxycarbonyl)-N^6-((4-((*S*)-2-((*tert*-butoxycarbonyl)amino)-3-methoxy-3-oxopropyl)phenyl)sulfonyl)-*L*-lysinate (**7**)

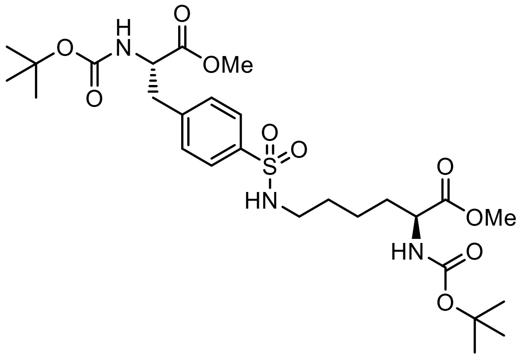

A glass reaction tube was charged with *N*-Boc-4-sulfonylfluoride phenylalanine methyl ester (**4u**, 30 mg, 0.08 mmol), dissolved in DMSO (420 μL) and treated with DIPEA (43 μL, 0.25 mmol) and methyl (tert-butoxycarbonyl)-*L*-lysinate (62 mg, 0.21 mmol). After 15 h at 100 °C, the reaction was allowed to cool to r.t., diluted with brine (10 mL) and extracted with EtOAc (2x15 mL), dried over anhydrous Na_2SO_4, filtered and concentrated *in vacuo* to leave the crude product, which was purified by column chromatography on silica (0-100% EtOAc in heptane) to provide methyl N^2-(*tert*-butoxycarbonyl)-N^6-((4-((*S*)-2-((*tert*-butoxycarbonyl)amino)-3-methoxy-3-oxopropyl)phenyl)sulfonyl)-*L*-lysinate as a colourless oil (42 mg, 0.070 mmol, 84%); IR ν_{max} (neat)/cm^{-1} 3291, 2979, 2362, 1745, 1697, 1161; ^{1}H NMR (400 MHz, $CDCl_3$) δ_H: 7.77 (d, *J* = 7.6 Hz, 2H), 7.27-7.34 (m, 2H), 5.11 (d, *J* = 7.6 Hz, 2H), 4.93 (br. s., 1H), 4.61 (d, *J* = 6.5 Hz, 1H), 4.23 (d, *J* = 4.7 Hz, 1H), 3.72 (s, 6H), 3.22 (dd, *J* = 13.5, 5.3 Hz, 1H), 3.08 (dd, *J* = 13.2, 6.2 Hz, 1H), 2.92 (q, *J* = 6.5 Hz, 2H), 1.67-1.79 (m, 1H), 1.53-1.62 (m, 1H), 1.45-1.53 (m, 2H), 1.36-1.45 (m, 18H), 1.29-

1.35 (m, 2H); ^{13}C {^{1}H} NMR (101 MHz, $CDCl_3$) δ_C: 173.1, 171.8, 155.44, 155.0, 141.5, 138.6, 130.0, 127.2, 80.1, 79.9, 54.1, 53.0, 52.4, 52.3, 42.8, 38.2, 32.2, 28.9, 28.3, 28.2, 22.2; HRMS (ESI$^+$) found *m*/*z* 624.2551 [M+Na]$^+$, $C_{27}H_{43}N_3NaO_{10}S$ requires *m*/*z* 624.2561.

N-Cbz-*N*'-Boc-Lys-Ala-Pro-(4-iodo)Phe-OMe (**8**)

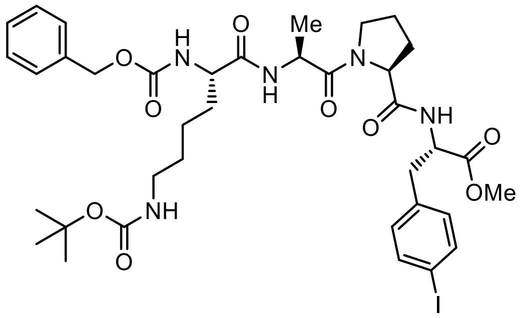

N-Boc-4-iodo phenylalanine methyl ester (*N*-Boc-(4-iodo)Phe, 380 mg, 0.94 mmol) was taken up in 20 % TFA/CH_2Cl_2 (7.5 mL) and stirred for 45 min at r.t. The solution was concentrated under reduced pressure and the resultant oil was taken up in 50 mL EtOAc and washed with sat. aq. $NaHCO_3$ (25 mL). The organic layer was dried over Na_2SO_4, filtered and concentrated. The resultant deprotected amine was taken up in DMF (5 mL) and treated with Et_3N (0.26 mL, 1.88 mmol) and *N*-Boc-Pro-Ala-OH (269 mg, 0.94 mmol). The solution was treated with HATU (441 mg, 1.13 mmol) and the resultant yellow solution was stirred at r.t. for 18 h. The reaction was quenched with sat. aq. $NaHCO_3$ (5 mL) and stirred for 30 min at r.t. The mixture was diluted with H_2O (50 mL) and extracted with EtOAc (2 × 50 mL). The combined organic extracts were dried over Na_2SO_4, filtered and concentrated. The crude product was purified by flash chromatography (20-100% EtOAc in heptane) to yield the desired target *N*-Boc-Ala-Pro-(4-iodo)Phe-OMe as a white solid (412 mg, 0.718 mmol, 77%).

N-Boc-Ala-Pro-(4-iodo)Phe-OMe (308 mg, 0.54 mmol) was taken up in 20 % TFA/CH_2Cl_2 (7.5 mL) and stirred for 45 min at r.t. The solution was concentrated under reduced pressure and the resultant oil was taken up in EtOAc (50 mL) and washed with sat. aq. $NaHCO_3$ (25 mL). The organic layer was dried over Na_2SO_4, filtered and concentrated. The resultant deprotected amine was taken up in DMF (5 mL) and treated with Et_3N (0.15 mL, 1.07 mmol) and *N*-Cbz-*N*'-Boc-Lys-OH (204 mg, 0.54 mmol). The solution was treated with HATU (253 mg, 0.65 mmol) and the resultant yellow solution was stirred at r.t. for 18 h. The reaction was quenched with sat. aq. $NaHCO_3$ (5 mL) and stirred for 30 min at r.t. The mixture was diluted with H_2O (50 mL) and extracted with EtOAc (2 × 50 mL). The

combined organic extracts were dried over Na_2SO_4, filtered and concentrated. The crude product was purified by flash chromatography (20-100% EtOAc in heptane) to yield the desired target *N*-Cbz-*N*'-Boc-Lys-Ala-Pro-(4-iodo)Phe-OMe as a white solid (241 mg, 0.288 mmol, 54%); IR ν_{max} (neat)/cm^{-1}; 3305, 2975, 1670, 1525, 1486, 1455, 1402, 1250, 1171, 849; ^{1}H NMR (600 MHz, dmso-d$_6$, cis/trans isomers observed, major peaks listed) δ_H 8.17 (d, *J*=7.04 Hz, 1H), 8.00 (d, *J*=7.04 Hz, 1H), 7.62 (d, *J*=8.22 Hz, 2H), 7.27-7.40 (m, 5H), 7.05 (d, *J*=8.22 Hz, 2H), 6.74 (br. s., 1H), 5.01 (s, 2H), 4.46-4.55 (m, 1H), 4.39-4.45 (m, 1H), 4.34 (dd, *J*=3.52, 8.22 Hz, 1H), 3.91-3.98 (m, 1H), 3.58 (s, 3H), 3.50 (d, *J*=6.46 Hz, 1H), 2.83-3.05 (m, 4H), 1.90-2.10 (m, 2H), 1.79-1.90 (m, 2H), 1.69-1.76 (m, 1H), 1.54-1.64 (m, 1H), 1.42-1.52 (m, 1H), 1.21-1.41 (m, 12H), 1.14-1.20 (m, 2H), 0.97-0.98 (m, 1H); ^{13}C NMR (151 MHz, dmso-d$_6$, cis/trans isomers observed, major peaks listed) δ_C 171.6, 171.5, 171.4, 170.4, 165.6, 155.9, 155.5, 137.0, 136.9, 136.8, 131.6, 131.5, 128.3, 127.7, 127.6, 92.4, 77.3, 65.3, 59.0, 54.5, 53.2, 51.8, 46.6, 46.0, 40.0, 39.9, 39.8, 39.6, 39.4, 39.2, 39.1, 36.0, 31.5, 31.2, 29.1, 28.8, 28.2, 24.3, 22.8, 22.1, 17.0, 13.9; HRMS (ESI$^+$) found *m*/*z* 836.2730 [M+H]$^+$, $C_{37}H_{51}IN_5O_9$ requires *m*/*z* 836.2726.

N-Cbz-*N*'-Boc-Lys-Ala-Pro-(4-sulfonylfluoride)Phe-OMe (**4v**)

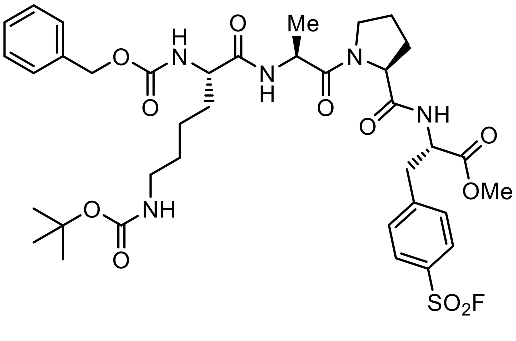

Prepared according to general procedure A using DABSO (12 mg, 0.048 mmol), $PdCl_2(AmPhos)_2$ (2.8 mg, 0.004 mmol), *N*-Cbz-*N*'-Boc-Lys-Ala-Pro-(4-iodo)Phe-OMe (67 mg, 0.08 mmol), anhydrous isopropanol (1 mL), anhydrous triethylamine (33 µL, 0.24 mmol) and NFSI (38 mg, 0.12 mmol). The crude product was purified by column chromatography on silica (5% MeOH in CH_2Cl_2) to provide *N*-Cbz-*N*'-Boc-Lys-Ala-Pro-(4-sulfonylfluoride)Phe-OMe as a colorless oil (41 mg, 0.0518 mmol, 65 %): IR ν_{max} (neat)/cm^{-1}; 2973, 2945, 2487, 2011, 1972, 1652, 1558; ^{1}H NMR (400 MHz, dmso-d$_6$) cis/trans isomers observed); ^{19}F NMR (376 MHz, DMSO) δ_F: 66.5; ^{13}C {^{1}H} NMR (101 MHz, dmso-d$_6$) cis/trans isomers observed; LRMS (ESI) *m*/*z* 792.7 ([M+H]$^+$), HRMS (ESI$^+$) found *m*/*z* 792.3274 [M+H]$^+$, $C_{37}H_{51}FN_5O_{11}S$ requires *m*/*z* 792.3284.

1.9 Synthesis of Sulfonyl Fluorides from Grignard Reagents

General Procedure C: Synthesis of sulfonyl fluorides from Grignard reagents, DABSO and NFSI as exemplified by the preparation of 4-fluorobenzenesulfonyl fluoride (**9a**)

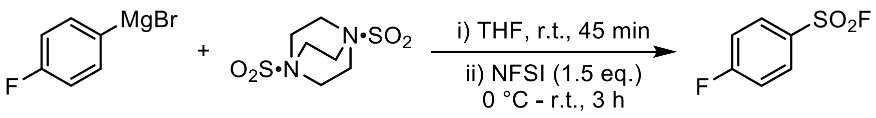

DABSO (240 mg, 1.0 mmol, 0.5 eq.) was suspended in anhydrous THF (4 mL) and the suspension purged with N_2 for 3 min. 4-Fluorophenylmagnesium bromide solution (1.84 mL, 2.0 mmol, 1.0 eq., 0.92 M in THF) was added dropwize and the mixture stirred at r.t. for 45 min. The solution was cooled to 0 °C, then NFSI (946 mg, 3.0 mmol, 1.5 eq.) was added portionwize at 0 °C and the reaction mixture stirred at r.t. for 3 h. The mixture was quenched with sat. aq. NH_4Cl and partitioned between EtOAc and brine, washing with EtOAc. The combined organic layers were dried ($MgSO_4$), filtered and concentrated *in vacuo* to leave the crude product, which was purified by column chromatography on silica (10% CH_2Cl_2 in pet. ether) to leave 4-fluorobenzenesulfonyl fluoride as a colourless oil (329.5 mg, 1.85 mmol, 93%) with spectroscopic data in accordance with the literature;[2] ^{1}H NMR (400 MHz, $CDCl_3$) δ_H: 8.04 (dd, *J* 9.0, 4.8 Hz, 2H), 7.29 (dd, *J* 9.0, 7.9 Hz, 2H); ^{19}F NMR (377 MHz, $CDCl_3$) δ_F: 66.8, −99.6 (ddd, *J* 7.9, 4.9, 3.2 Hz); ^{13}C {^{1}H} NMR (101 MHz, $CDCl_3$) δ_C: 166.3 (d, *J* 259.9 Hz), 143.1 (d, *J* 3.2 Hz), 129.6 (d, *J* 10.3 Hz), 117.0 (d, *J* 23.1 Hz); HRMS (CI) found *m*/*z* 178.9979 $[M+H]^+$, $C_6H_5F_2O_2S$ requires *m*/*z* 178.9973.

Phenylmethanesulfonyl fluoride (**9b**)

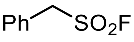

Prepared according to general procedure C using DABSO (240 mg, 1.0 mmol), benzylmagnesium chloride solution (1.68 mL, 2.0 mmol, 1.19 M in THF), anhydrous THF (4 mL) and NFSI (946 mg, 3.0 mmol). The crude product was purified by column chromatography on silica (10% CH_2Cl_2 in pet. ether) to leave 3-methyl-4-oxo-3,4-dihydroquinazoline-6-sulfonyl fluoride as a white crystalline solid (222.8 mg, 1.28 mmol, 64%) with spectroscopic data in accordance with the literature;[2] *mp* 98-99 °C; ^{1}H NMR (400 MHz, $CDCl_3$) δ_H: 7.48 – 7.41 (m, 5H), 4.60 (d, *J* 3.3 Hz, 2H); ^{19}F NMR (377 MHz, $CDCl_3$) δ_F: 51.4 (t, *J* 3.3 Hz); ^{13}C {^{1}H} NMR (101 MHz, $CDCl_3$) δ_C: 130.7, 129.9, 129.3, 125.5, 56.8 (d, *J* 17.5 Hz); HRMS (CI) found *m*/*z* 192.0488 $[M+NH_4]^+$, $C_7H_{11}FNO_2S$ requires *m*/*z* 192.0489.

Cyclohexanesulfonyl fluoride (**9c**)

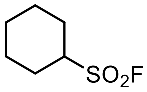

Prepared according to general procedure C using DABSO (240 mg, 1.0 mmol), cyclohexylmagnesium chloride solution (1.43 mL, 2.0 mmol, 1.40 M in THF), anhydrous THF (4 mL) and NFSI (946 mg, 3.0 mmol). The crude product was purified by column chromatography on silica (10% CH_2Cl_2 in pet. ether) to leave cyclohexanesulfonyl fluoride as a colourless oil (220.0 mg, 1.32 mmol, 66%) with

spectroscopic data in accordance with the literature;[4] ^{1}H NMR (400 MHz, $CDCl_3$) δ_H: 3.30 (ttd, *J* 12.1, 3.5, 1.6 Hz, 1H), 2.30 (dddt, *J* 12.0, 3.8, 2.5, 1.4 Hz, 2H), 2.00 – 1.91 (m, 2H), 1.80 – 1.61 (m, 3H), 1.43 – 1.20 (m, 3H); ^{19}F NMR (377 MHz, $CDCl_3$) δ_F: 40.8; ^{13}C NMR (101 MHz, $CDCl_3$) δ: 61.0 (d, *J* 12.7 Hz), 26.5, 24.72, 24.67; HRMS (CI) found *m*/*z* 184.0807 $[M+NH_4]^+$, $C_6H_{15}FNO_2S$ requires *m*/*z* 184.0802.

4-Chlorobenzenesulfonyl fluoride (**9d**)

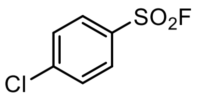

Prepared according to general procedure C using DABSO (240 mg, 1.0 mmol), 4-chlorophenylmagnesium bromide solution (2.67 mL, 2.0 mmol, 0.75 M in 2-MeTHF), anhydrous THF (4 mL) and NFSI (946 mg, 3.0 mmol). The crude product was purified by column chromatography on silica (10% CH_2Cl_2 in pet. ether) to leave 4-chlorobenzenesulfonyl fluoride as a white crystalline solid (278.7 mg, 1.43 mmol, 72%) with spectroscopic data in accordance with the literature;[4] *mp* 42-44 °C; ^{1}H NMR (400 MHz, $CDCl_3$) δ_H: 7.94 (d, *J* 8.8 Hz, 2H), 7.59 (d, *J* 8.8 Hz, 2H); ^{19}F NMR (377 MHz, $CDCl_3$) δ_F: 66.6; ^{13}C {^{1}H} NMR (101 MHz, $CDCl_3$) δ_C: 142.1, 130.0 (d, *J* 25.3 Hz), 129.9, 127.9; HRMS not available.

3-Methylbenzenesulfonyl fluoride (**9e**)

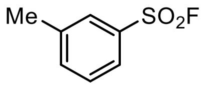

Prepared according to general procedure C using DABSO (240 mg, 1.0 mmol), 3-methylphenylmagnesium bromide solution (3.17 mL, 2.0 mmol, 0.63 M in THF), anhydrous THF (4 mL) and NFSI (946 mg, 3.0 mmol). The crude product was purified by column chromatography on silica (10% CH_2Cl_2 in pet. ether) to leave 3-methylbenzenesulfonyl fluoride as a colourless oil (252.3 mg, 1.45 mmol, 73%); IR ν_{max} (neat)/cm^{-1} 1404, 1321, 1200, 1098; ^{1}H NMR (400 MHz, $CDCl_3$) δ_H: 7.84 – 7.78 (m, 2H), 7.61 – 7.55 (m, 1H), 7.54 – 7.47 (m, 1H), 2.47 (s, 2H); ^{19}F NMR (377 MHz, $CDCl_3$) δ_F: 65.8; ^{13}C {^{1}H} NMR (101 MHz, $CDCl_3$) δ_C: 140.2, 136.4, 132.9 (d, *J* 23.7 Hz), 129.5, 128.6, 125.5, 21.3; HRMS (CI) found *m*/*z* 192.0493 $[M+NH_4]^+$, $C_7H_{11}FNO_2S$ requires *m*/*z* 192.0489.

Thiophene-2-sulfonyl fluoride (**9f**)

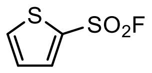

Prepared according to general procedure C using DABSO (240 mg, 1.0 mmol), 2-thienylmagnesium bromide solution (2.70 mL, 2.0 mmol, 0.74 M in THF), anhydrous THF (4 mL) and NFSI (946 mg,

3.0 mmol). The crude product was purified by column chromatography on silica (10% CH_2Cl_2 in pet. ether) to leave thiophene-2-sulfonyl fluoride as a yellow solid (258.4 mg, 1.55 mmol, 78%) with spectroscopic data in accordance with the literature;[2] *mp* 39-40 °C; ^{1}H NMR (400 MHz, $CDCl_3$) δ_H: 7.87 (dd, *J* 3.9, 1.4 Hz, 1H), 7.81 (dd, *J* 5.0, 1.4 Hz, 1H), 7.17 (dd, *J* 5.0, 3.9 Hz, 1H); ^{19}F NMR (377 MHz, $CDCl_3$) δ_F: 71.8; ^{13}C {^{1}H} NMR (101 MHz, $CDCl_3$) δ_C: 147.1, 135.4, 134.1, 127.5; HRMS not available.

2 References

[1] C. W. Muir, A. R. Kennedy, J. M. Redmond, A. J. B. Watson, *Org. Biomol. Chem.* **2013**, *11*, 3337-3340.

[2] L. Tang, Y. Yang, L. Wen, X. Yang, Z. Wang, *Green Chemistry* **2016**, *18*, 1224-1228.

[3] L. Matesic, N. A. Wyatt, B. H. Fraser, M. P. Roberts, T. Q. Pham, I. Greguric, *J. Org. Chem.* **2013**, *78*, 11262-11270.

[4] M. Kirihara, S. Naito, Y. Nishimura, Y. Ishizuka, T. Iwai, H. Takeuchi, T. Ogata, H. Hanai, Y. Kinoshita, M. Kishida, K. Yamazaki, T. Noguchi, S. Yamashoji, *Tetrahedron* **2014**, *70*, 2464-2471.

3 NMR Spectra

3.1 NMR Spectra of Aryl Bromides

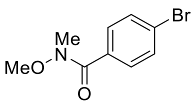

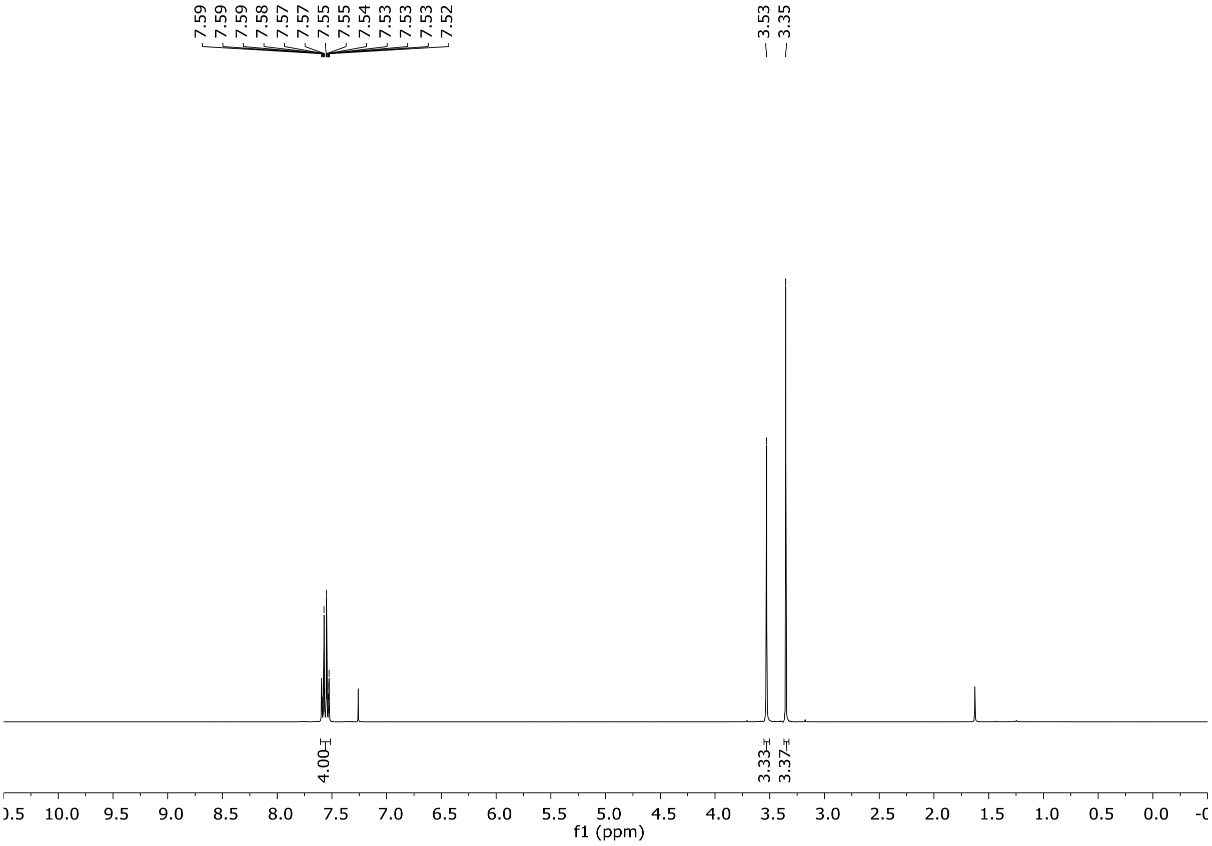

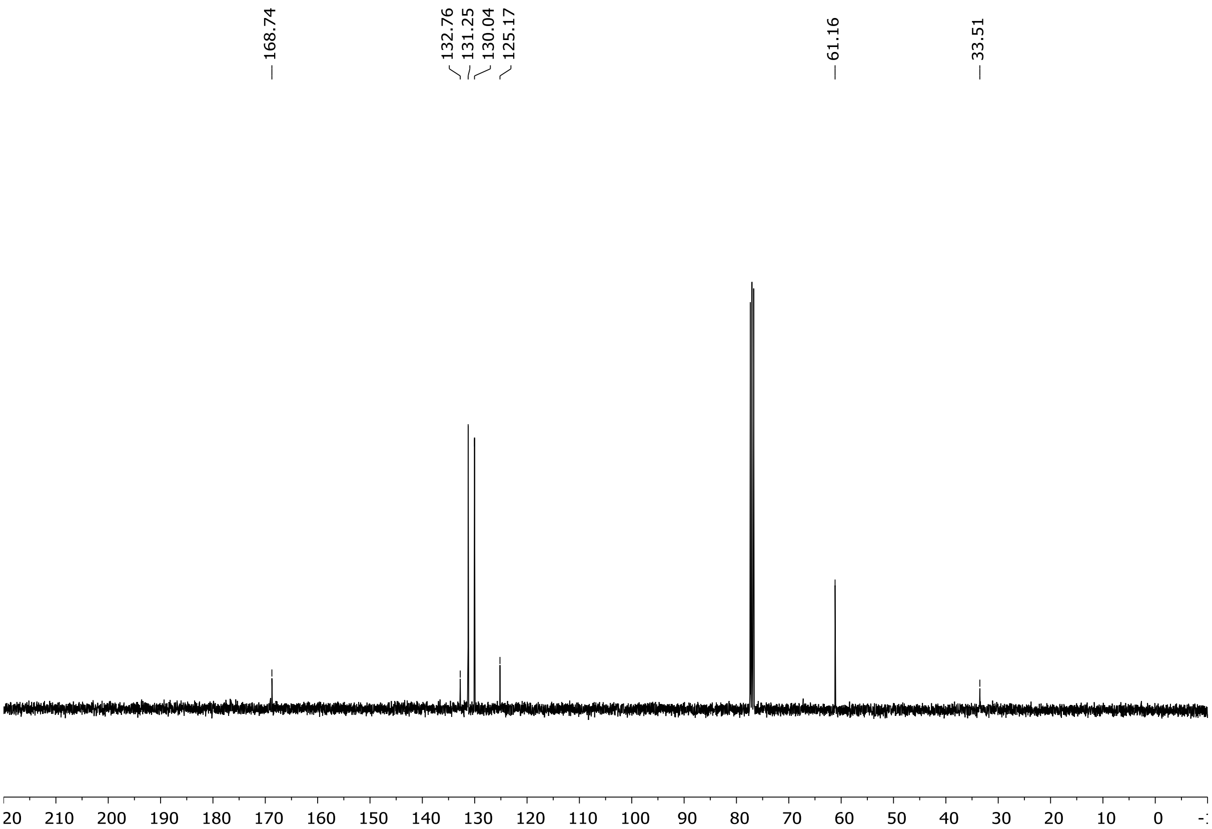

3.2 NMR Spectra of Sulfonyl Fluorides Synthesized by Palladium Catalysis

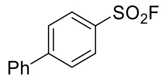

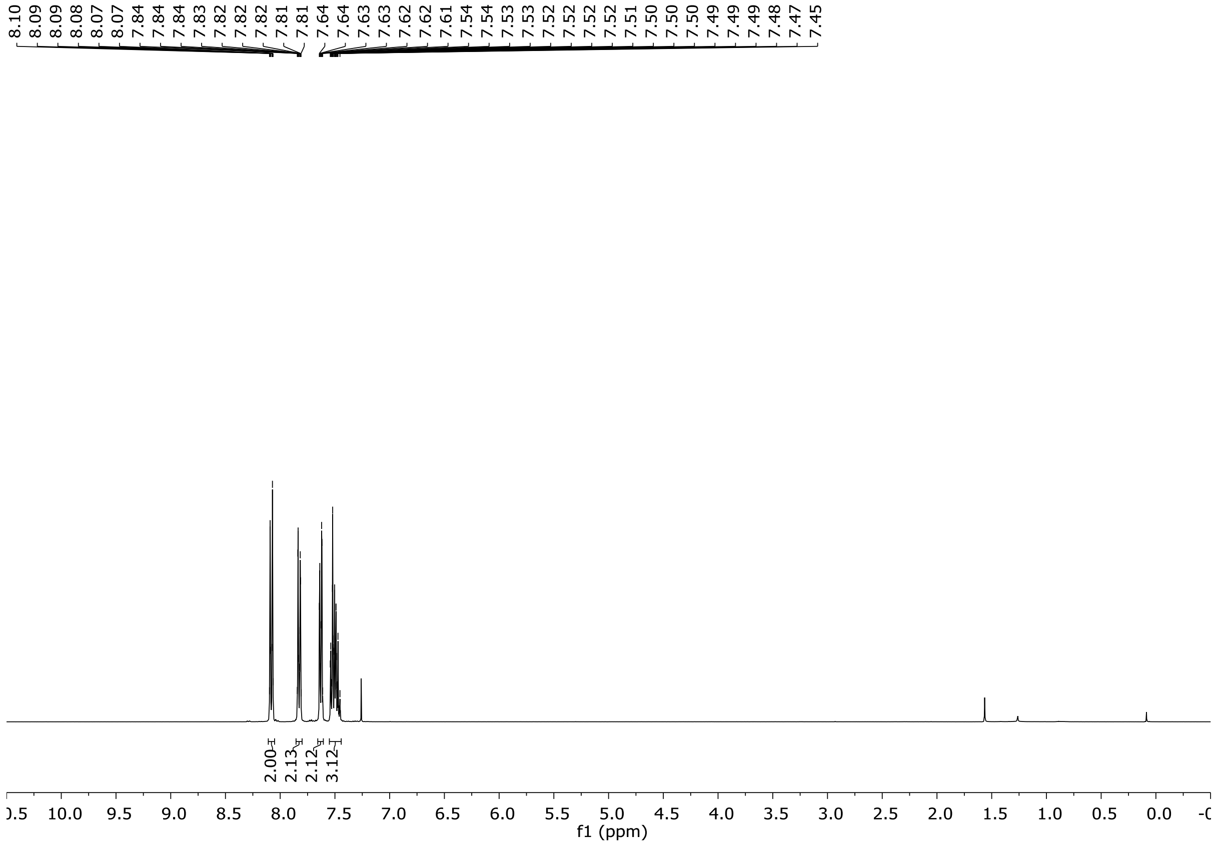

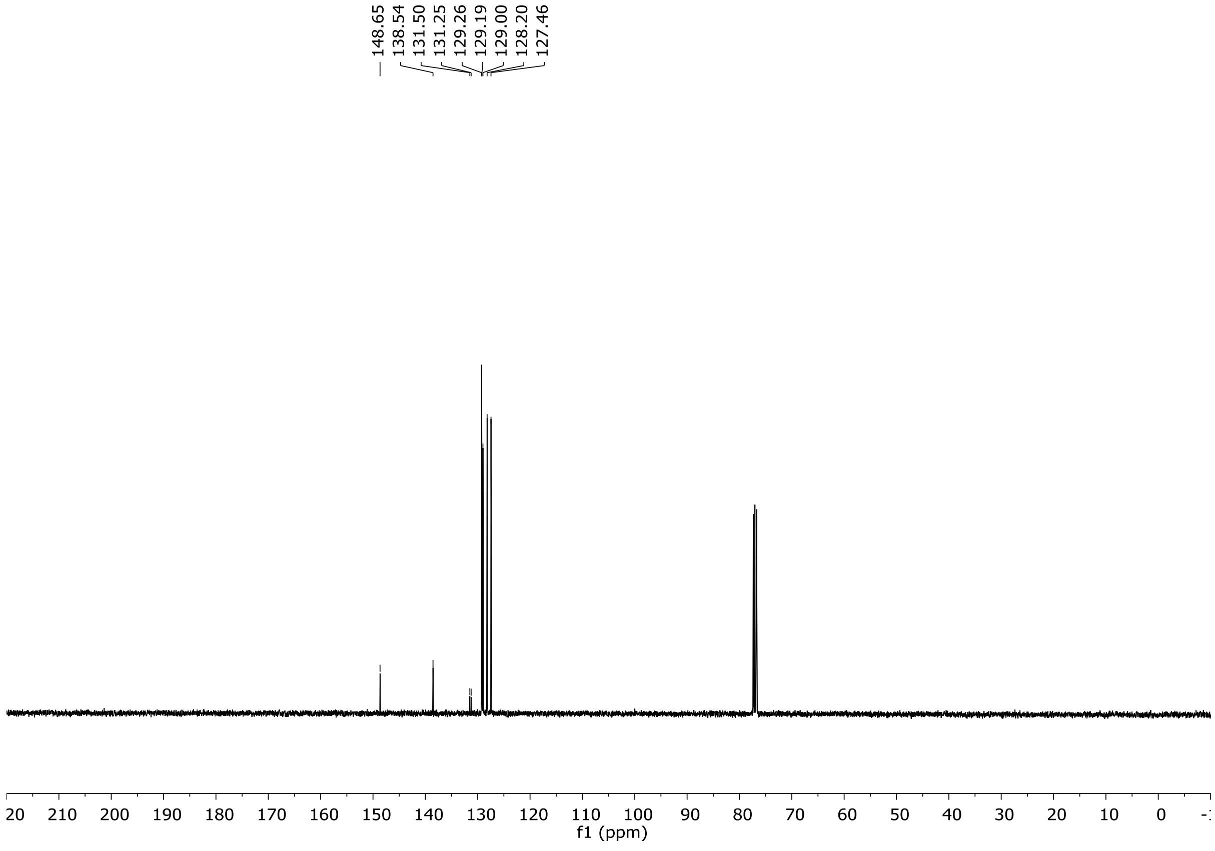

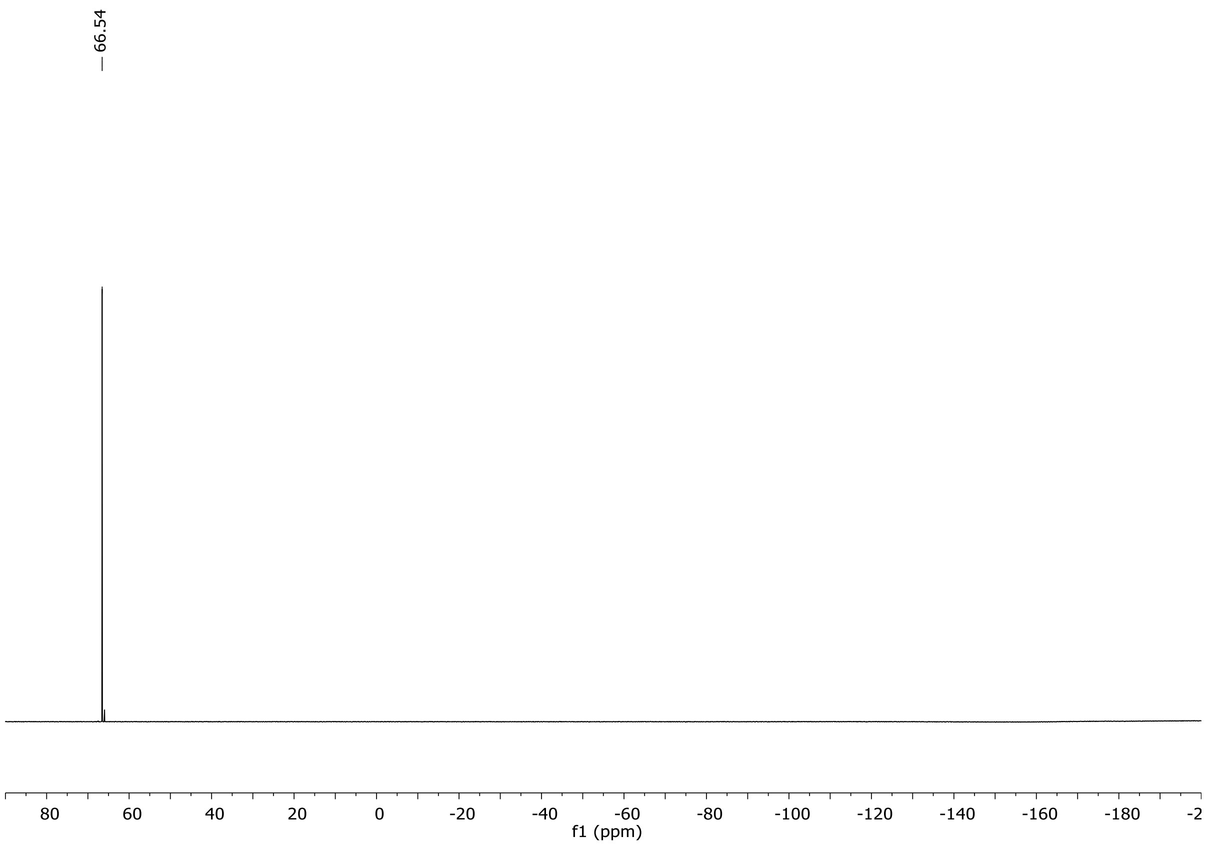

66.54
80
60
40
20
0
-20
-40
-60
-80
-100
-120
-140
-160
-180
-2
f1 (ppm)

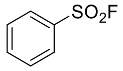
SO2F

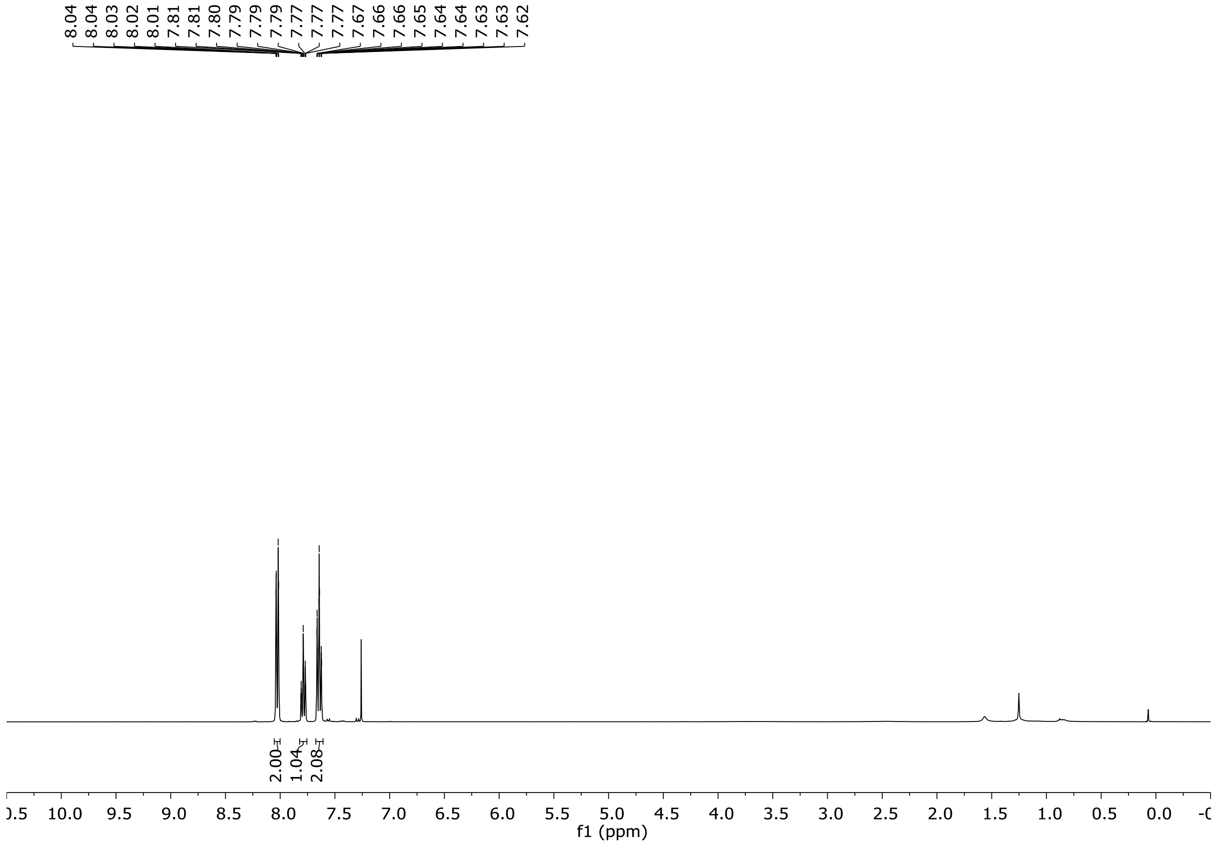
8.04
8.04
8.03
8.02
8.01
7.81
7.81
7.80
7.79
7.79
7.79
7.77
7.77
7.77
7.67
7.66
7.66
7.65
7.64
7.64
7.63
7.63
7.62
2.00
1.04
2.08
f1 (ppm)

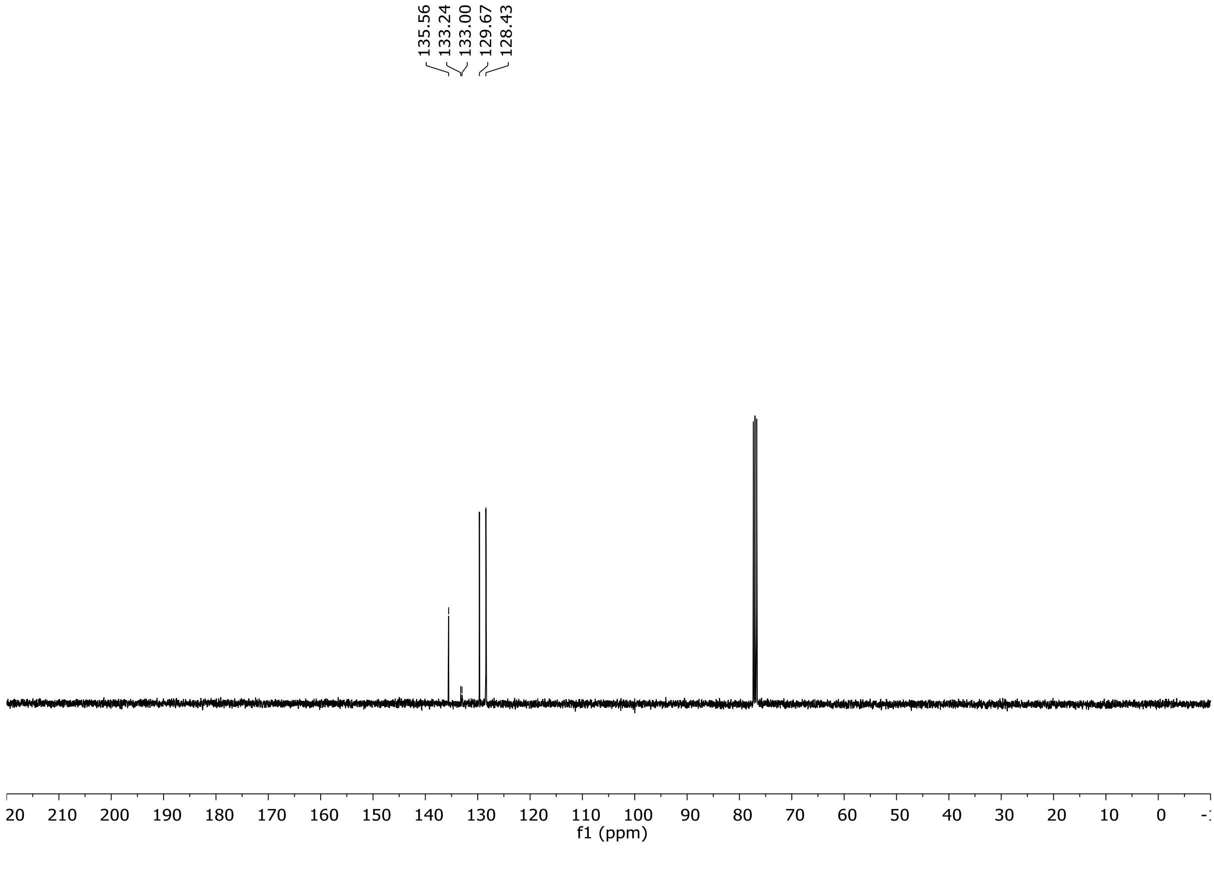
135.56
133.24
133.00
129.67
128.43
f1 (ppm)

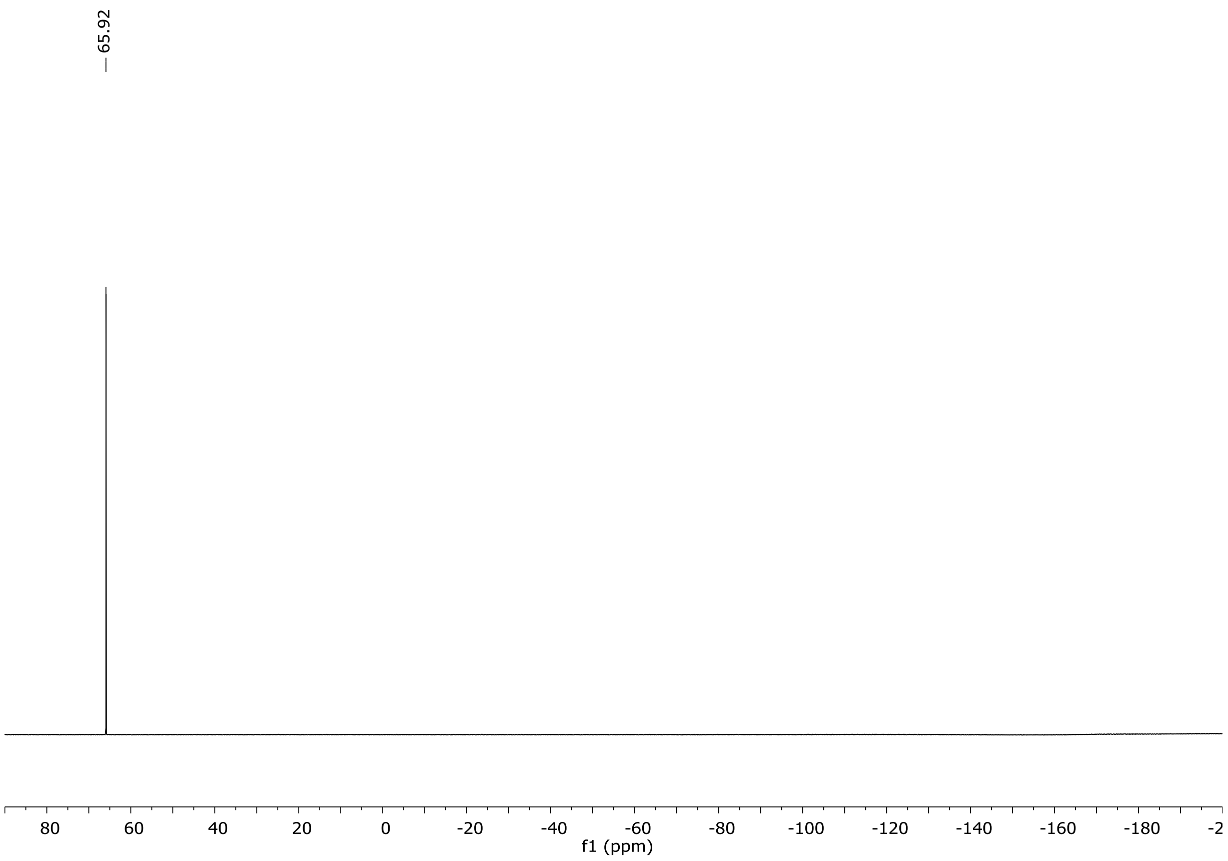
65.92
80 60 40 20 0 -20 -40 -60 -80 -100 -120 -140 -160 -180 -2
f1 (ppm)

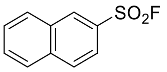
SO2F

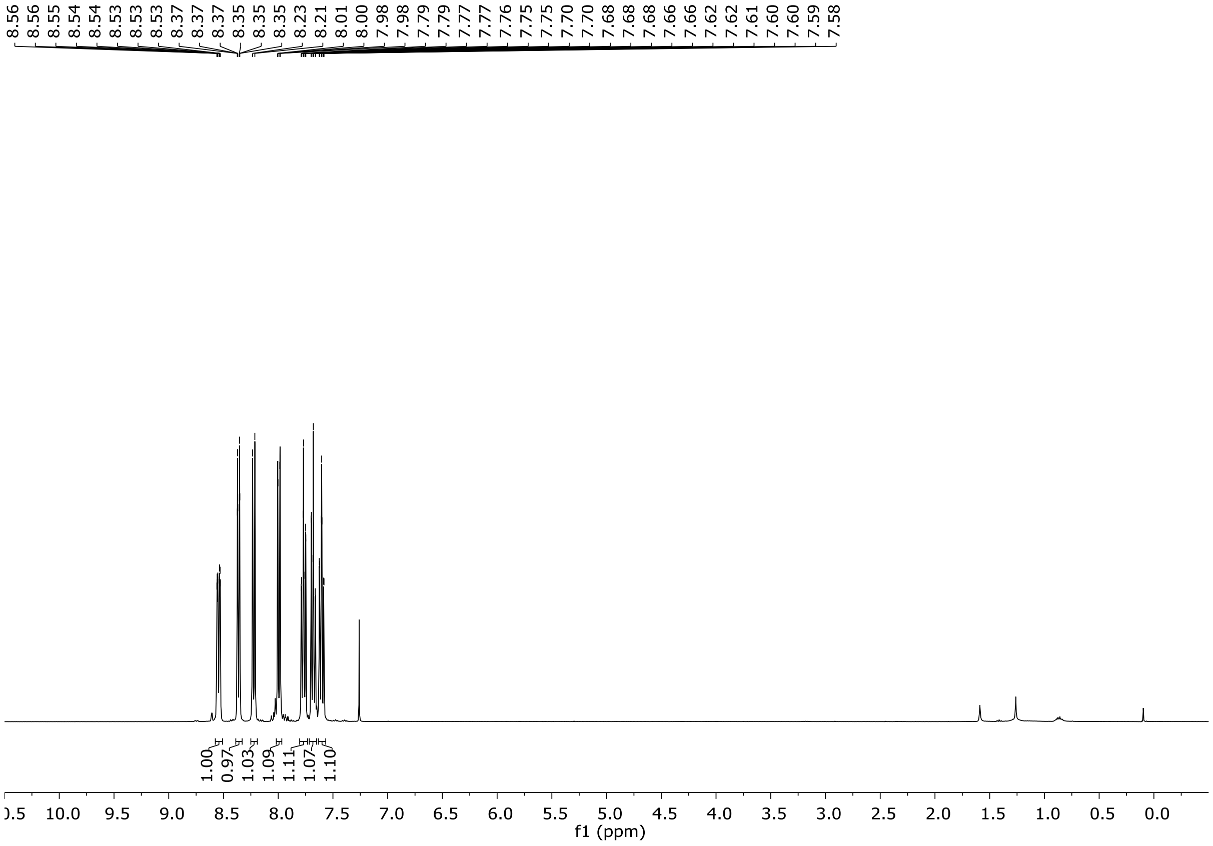
8.56 8.56 8.55 8.54 8.54 8.53 8.53 8.53 8.37 8.37 8.37 8.37 8.35 8.35 8.35 8.23 8.21 8.01 8.00 7.98 7.98 7.79 7.79 7.77 7.77 7.76 7.75 7.75 7.70 7.70 7.68 7.68 7.66 7.66 7.62 7.62 7.61 7.60 7.60 7.59 7.58
1.00 0.97 1.03 1.09 1.11 1.07 1.10
f1 (ppm)

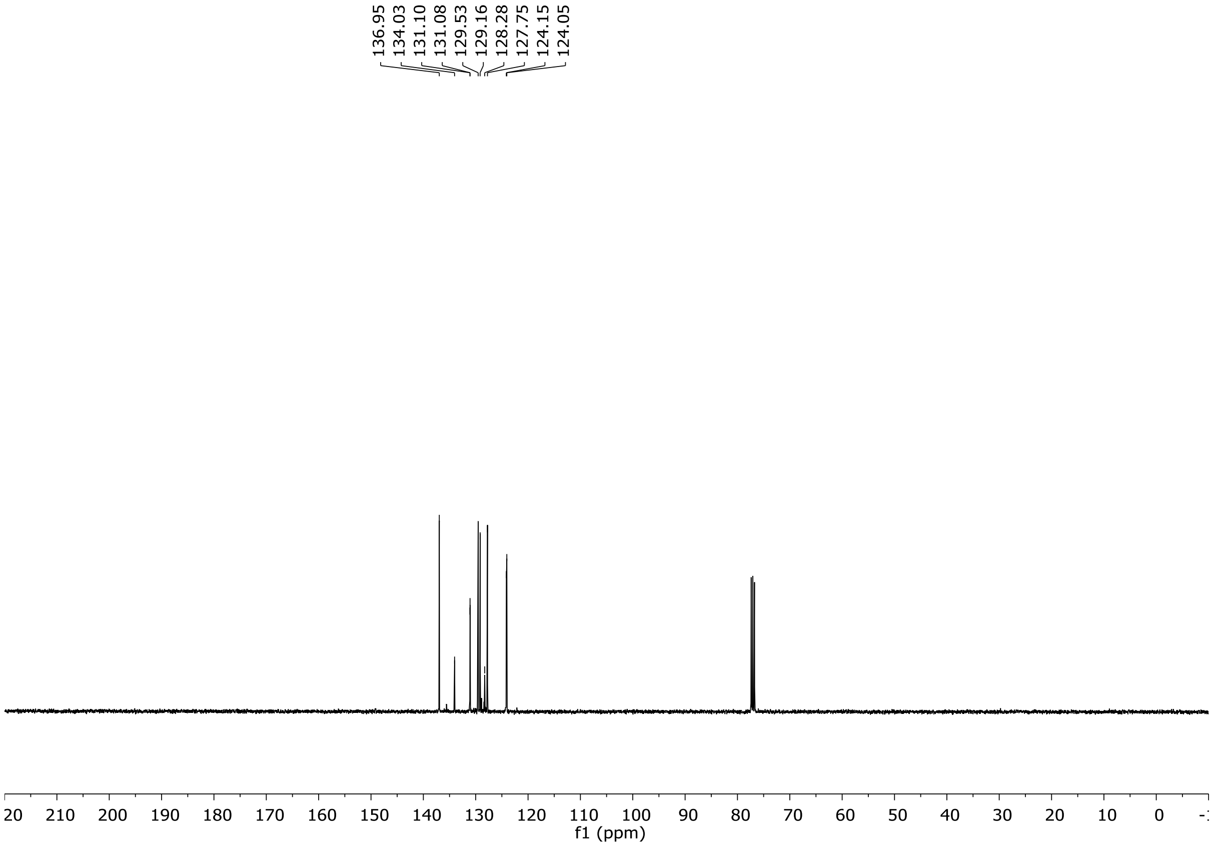
136.95 134.03 131.10 131.08 129.53 129.16 128.28 127.75 124.15 124.05
f1 (ppm)

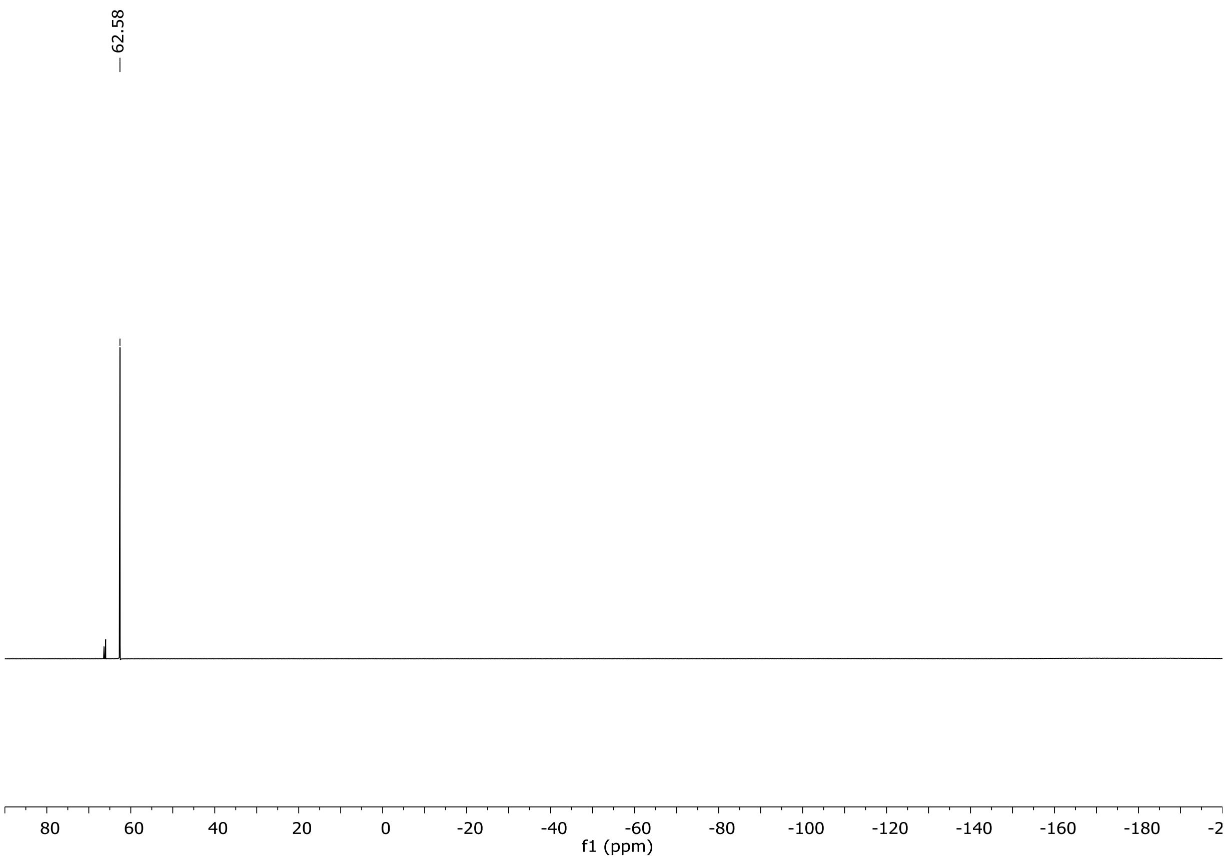
62.58
80
60
40
20
0
-20
-40
-60
-80
-100
-120
-140
-160
-180
-2
f1 (ppm)

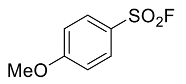
SO2F
MeO

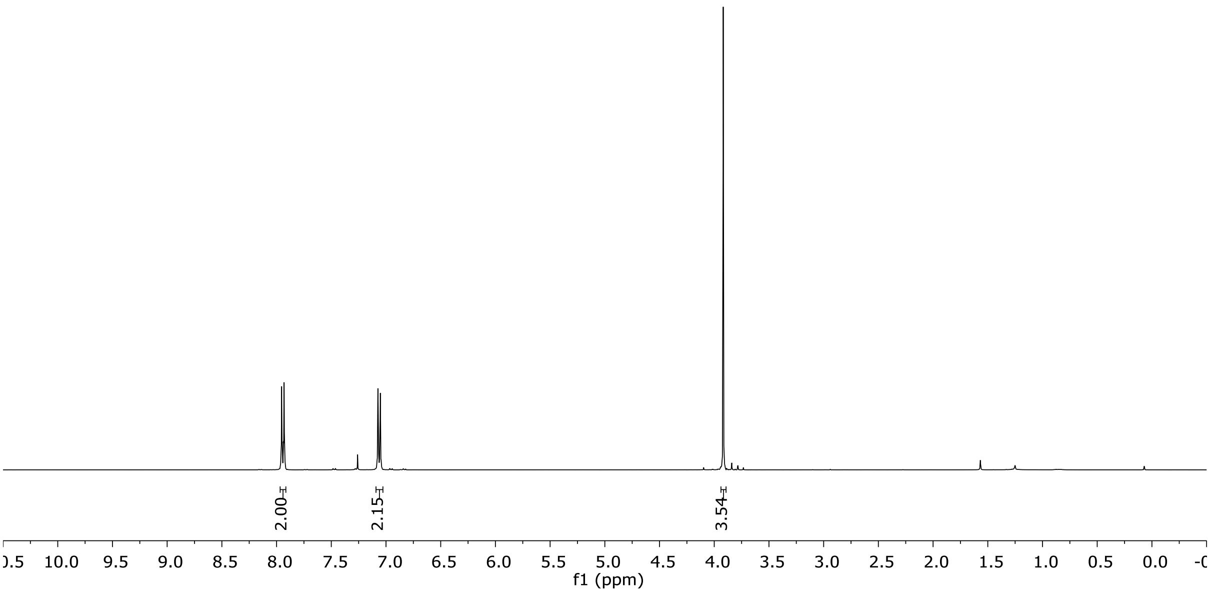
2.00
2.15
3.54
10.0 9.5 9.0 8.5 8.0 7.5 7.0 6.5 6.0 5.5 5.0 4.5 4.0 3.5 3.0 2.5 2.0 1.5 1.0 0.5 0.0
f1 (ppm)

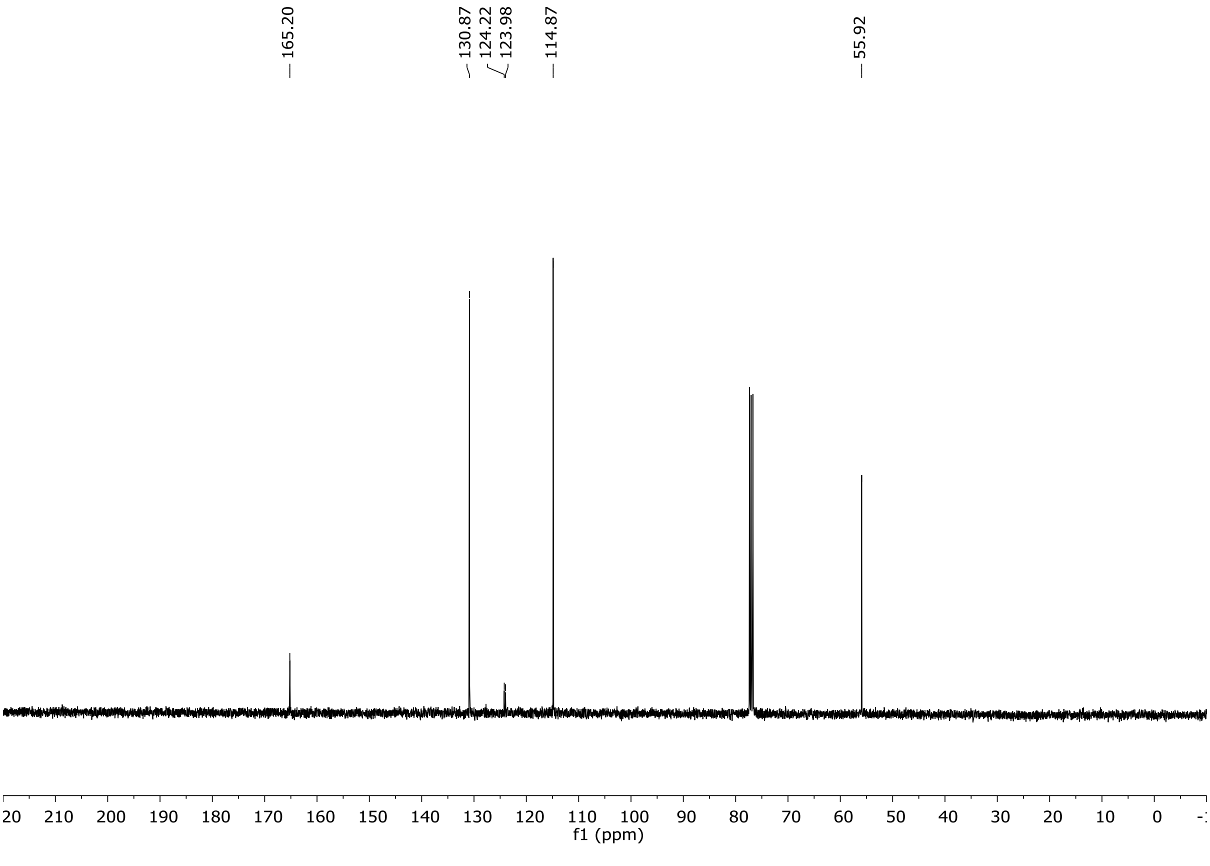
165.20
130.87
124.22
123.98
114.87
55.92
210 200 190 180 170 160 150 140 130 120 110 100 90 80 70 60 50 40 30 20 10 0
f1 (ppm)

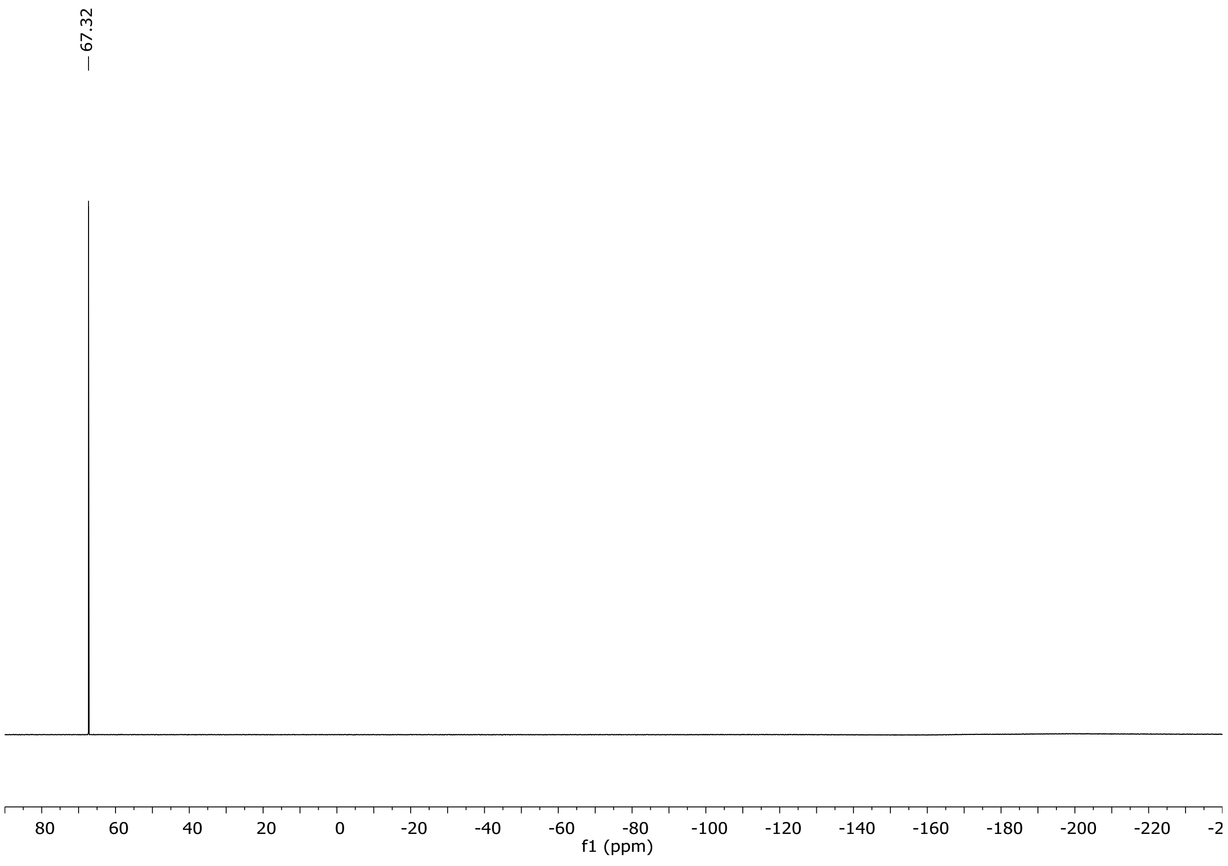

67.32
80
60
40
20
0
-20
-40
-60
-80
-100
-120
-140
-160
-180
-200
-220
-2
f1 (ppm)

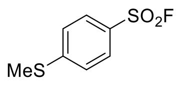
SO2F
MeS

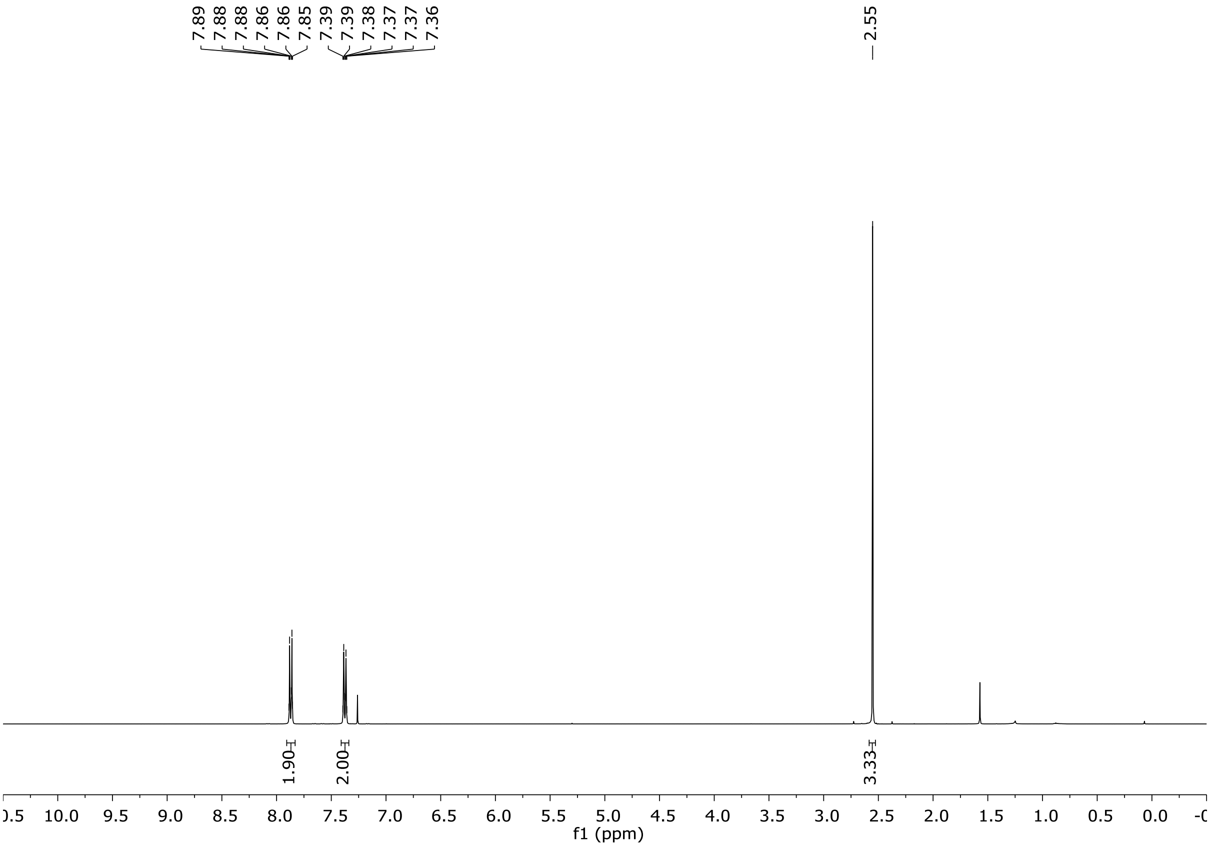
7.89
7.88
7.88
7.86
7.86
7.85
7.39
7.39
7.38
7.37
7.37
7.36
2.55
1.90
2.00
3.33
f1 (ppm)

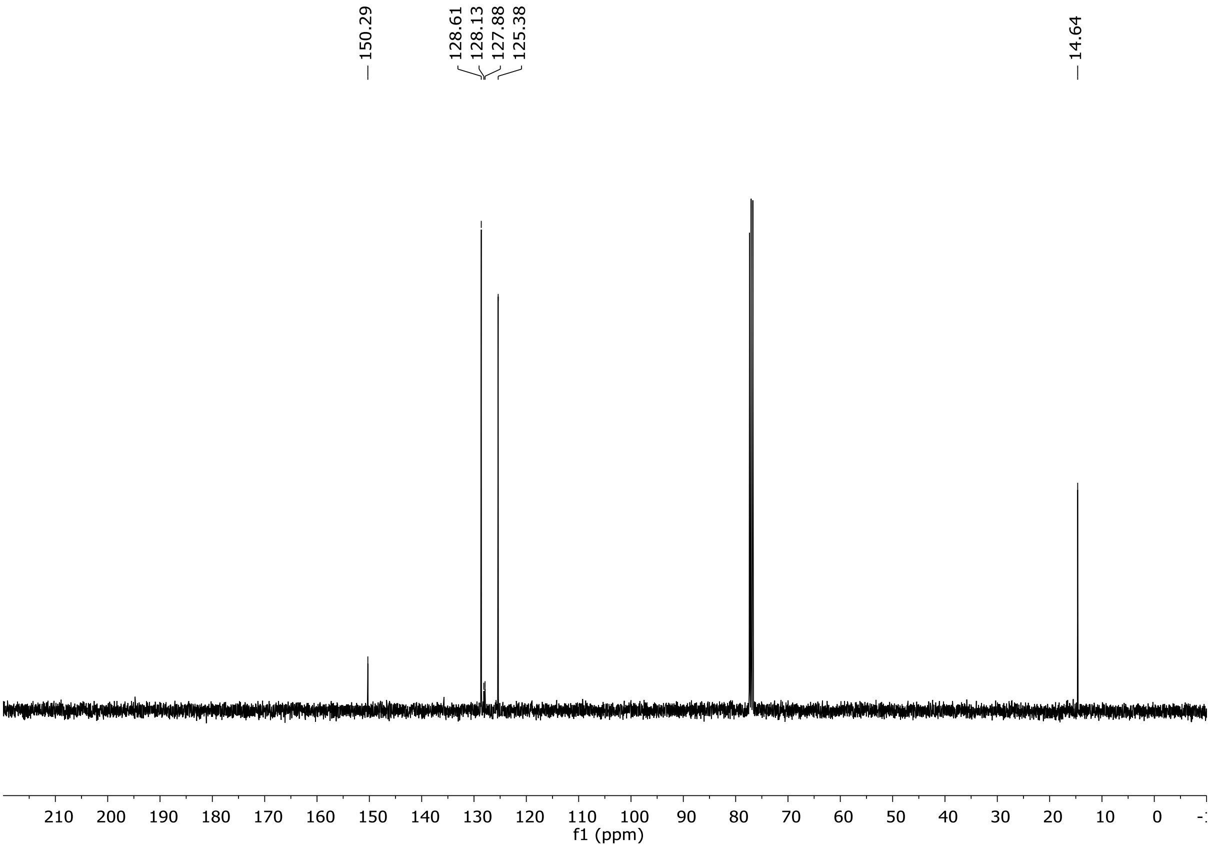
150.29
128.61
128.13
127.88
125.38
14.64
f1 (ppm)

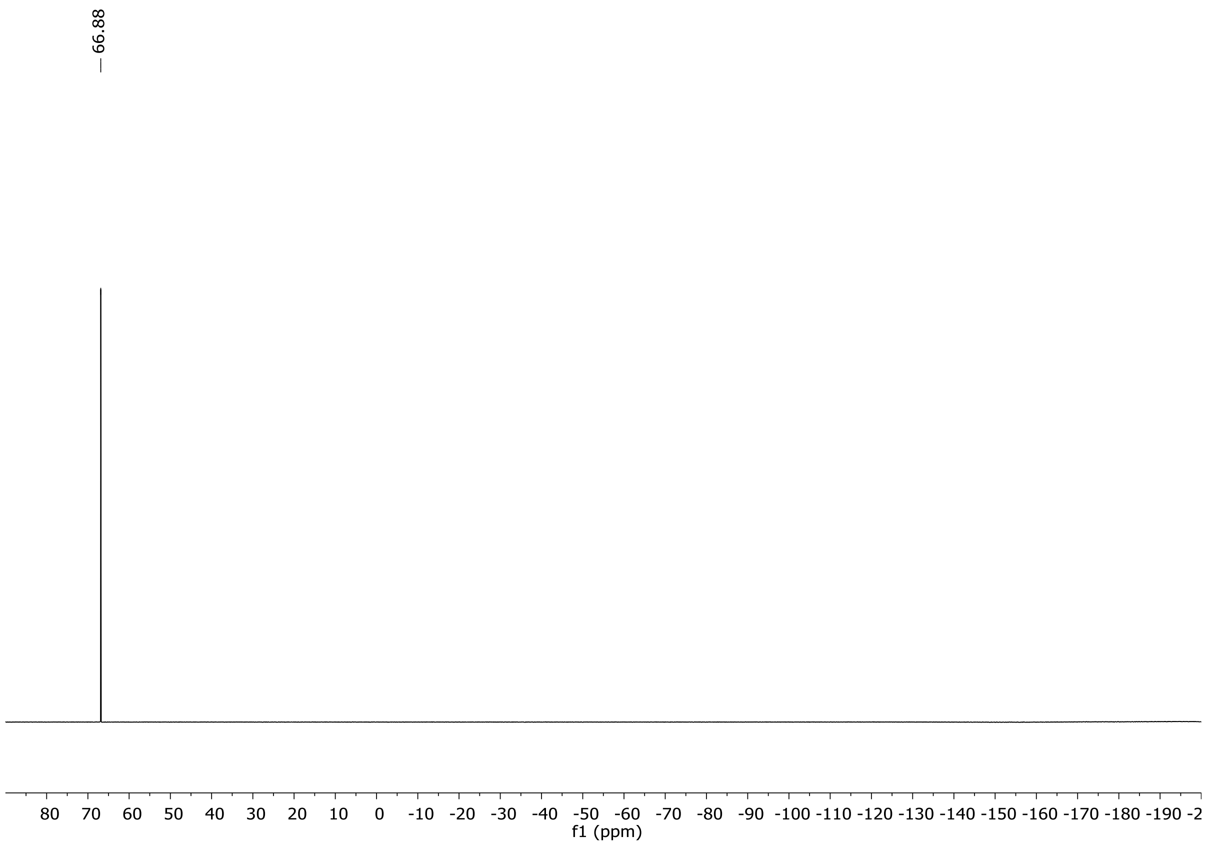
66.88
80 70 60 50 40 30 20 10 0 -10 -20 -30 -40 -50 -60 -70 -80 -90 -100 -110 -120 -130 -140 -150 -160 -170 -180 -190 -2
f1 (ppm)

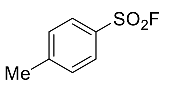
SO2F
Me

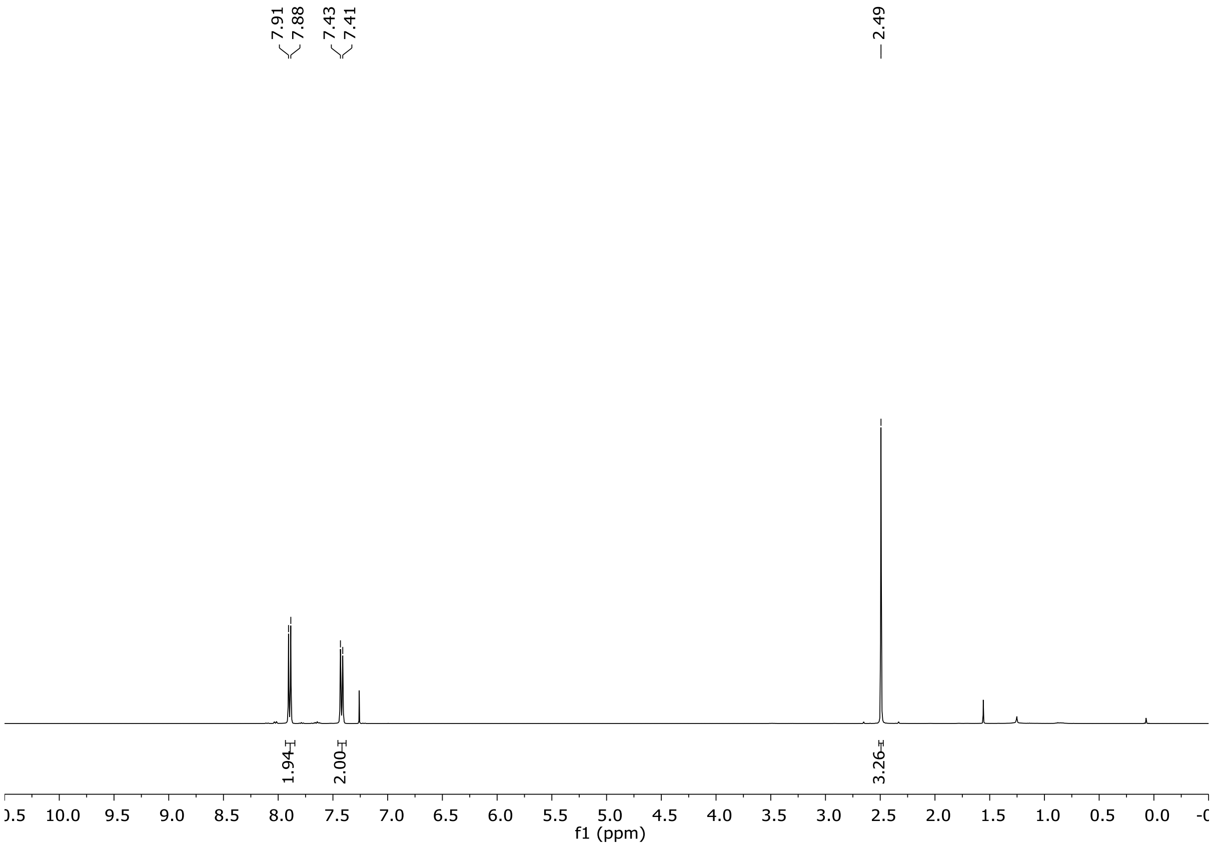
7.91
7.88
7.43
7.41
2.49
1.94
2.00
3.26
10.0 9.5 9.0 8.5 8.0 7.5 7.0 6.5 6.0 5.5 5.0 4.5 4.0 3.5 3.0 2.5 2.0 1.5 1.0 0.5 0.0
f1 (ppm)

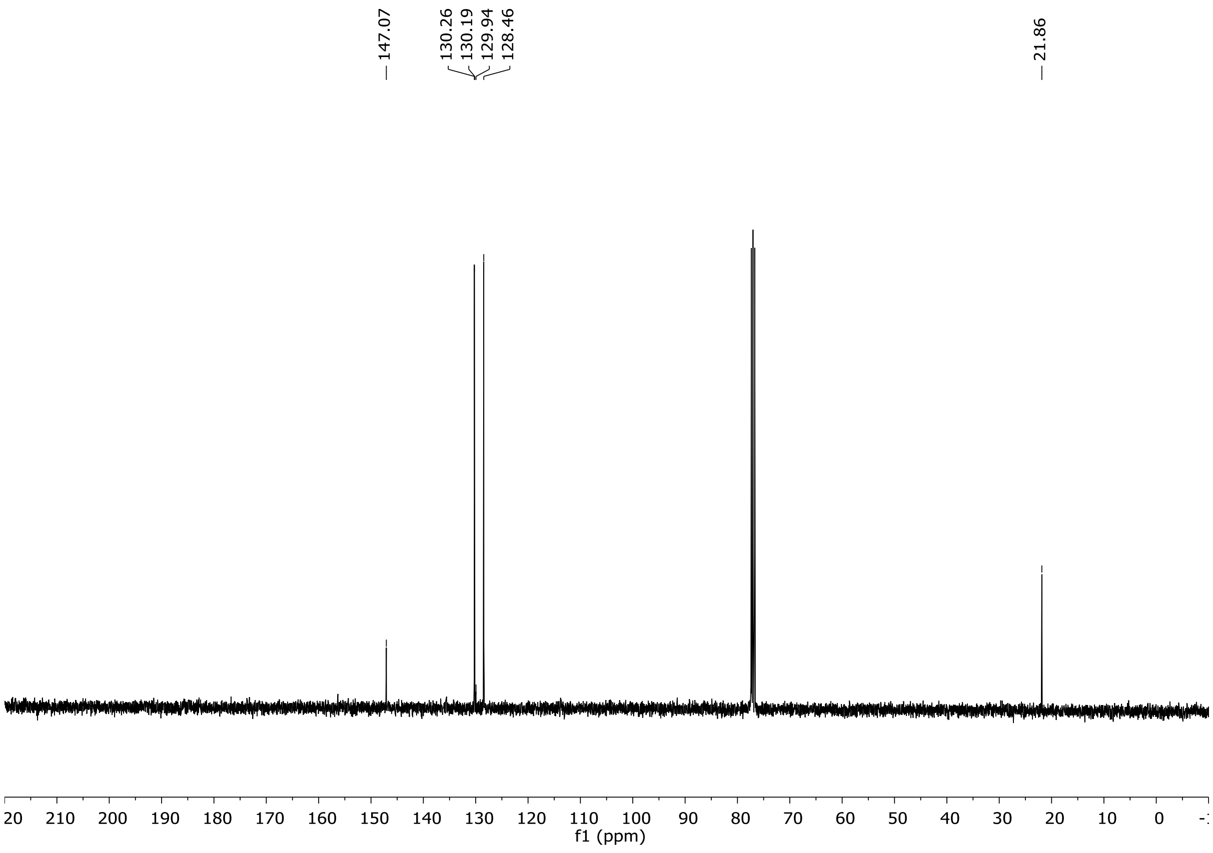
147.07
130.26
130.19
129.94
128.46
21.86
210 200 190 180 170 160 150 140 130 120 110 100 90 80 70 60 50 40 30 20 10 0
f1 (ppm)

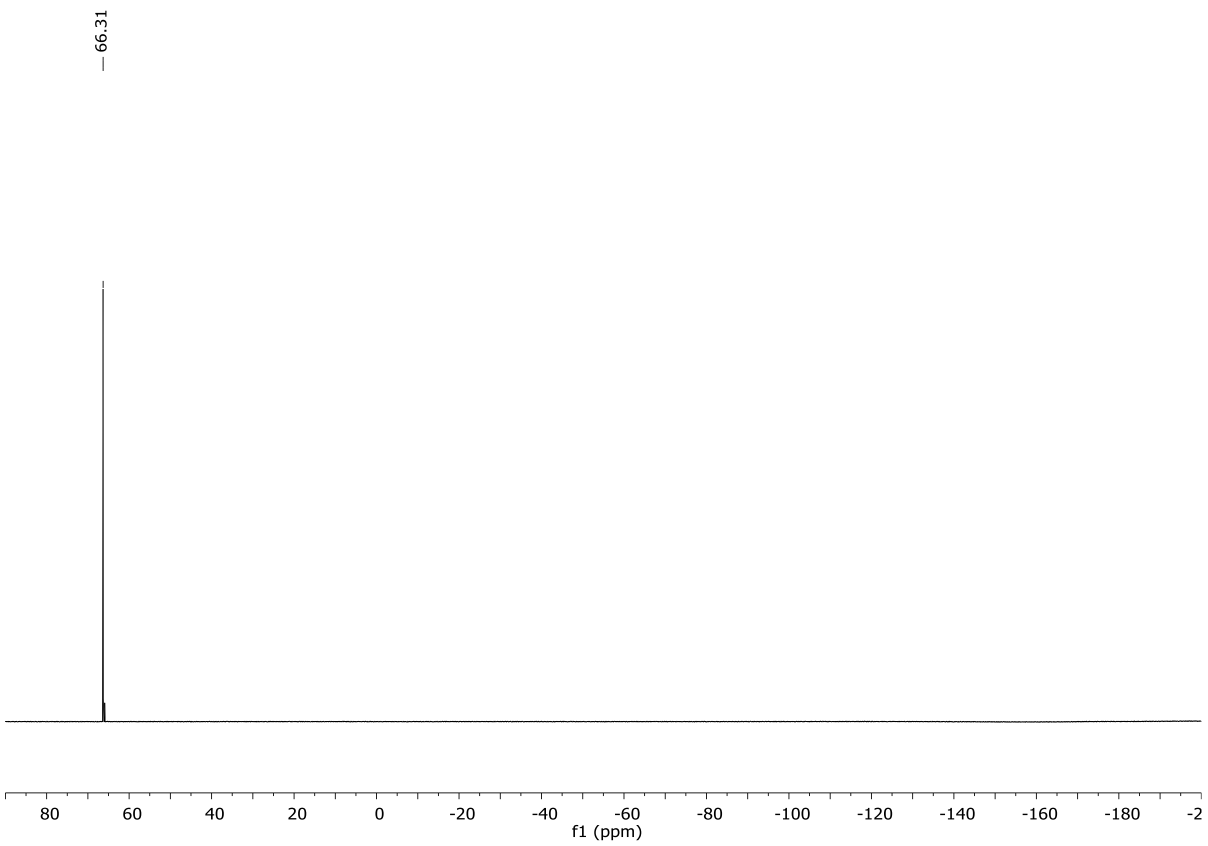

66.31
80
60
40
20
0
-20
-40
-60
-80
-100
-120
-140
-160
-180
-2
f1 (ppm)

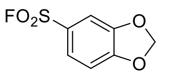
FO2S
O
O

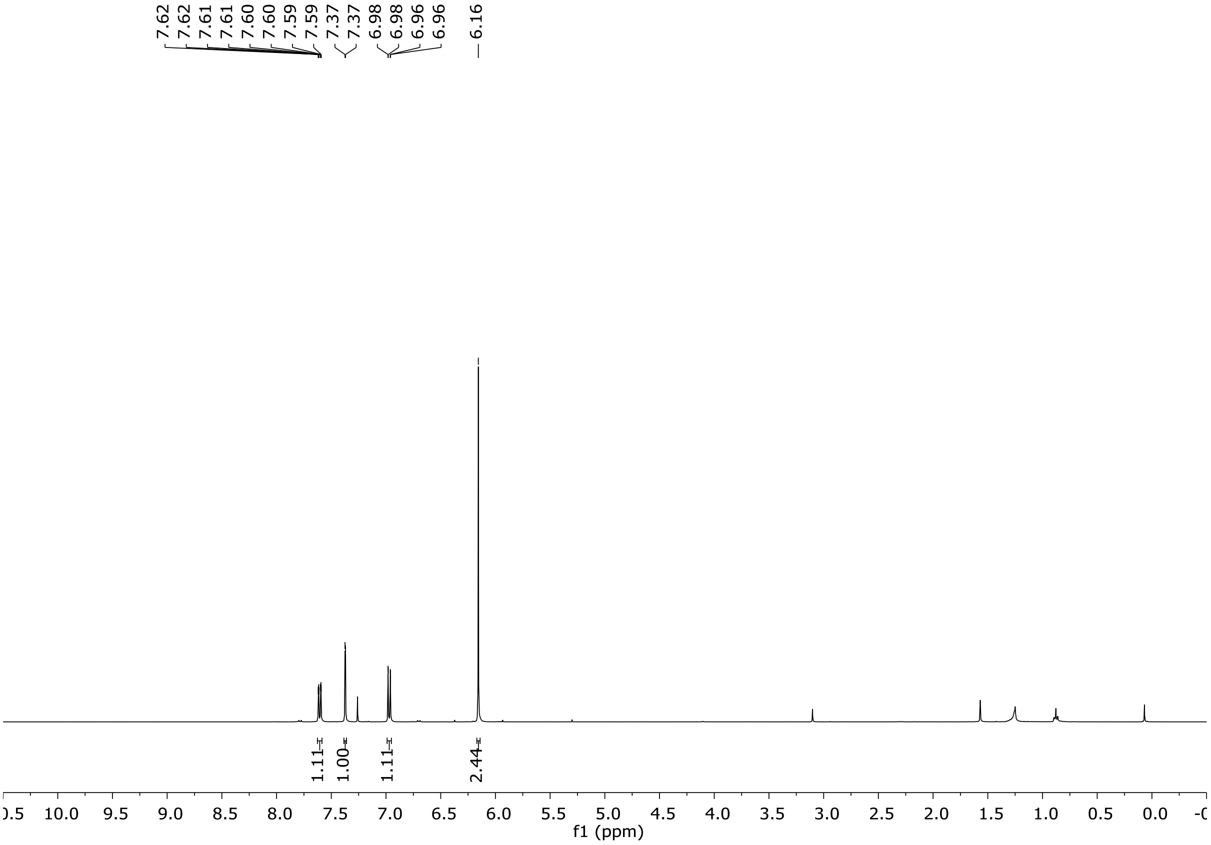
7.62
7.62
7.61
7.61
7.60
7.60
7.59
7.59
7.37
7.37
6.98
6.98
6.96
6.96
6.16
1.11
1.00
1.11
2.44
0.5 10.0 9.5 9.0 8.5 8.0 7.5 7.0 6.5 6.0 5.5 5.0 4.5 4.0 3.5 3.0 2.5 2.0 1.5 1.0 0.5 0.0
f1 (ppm)

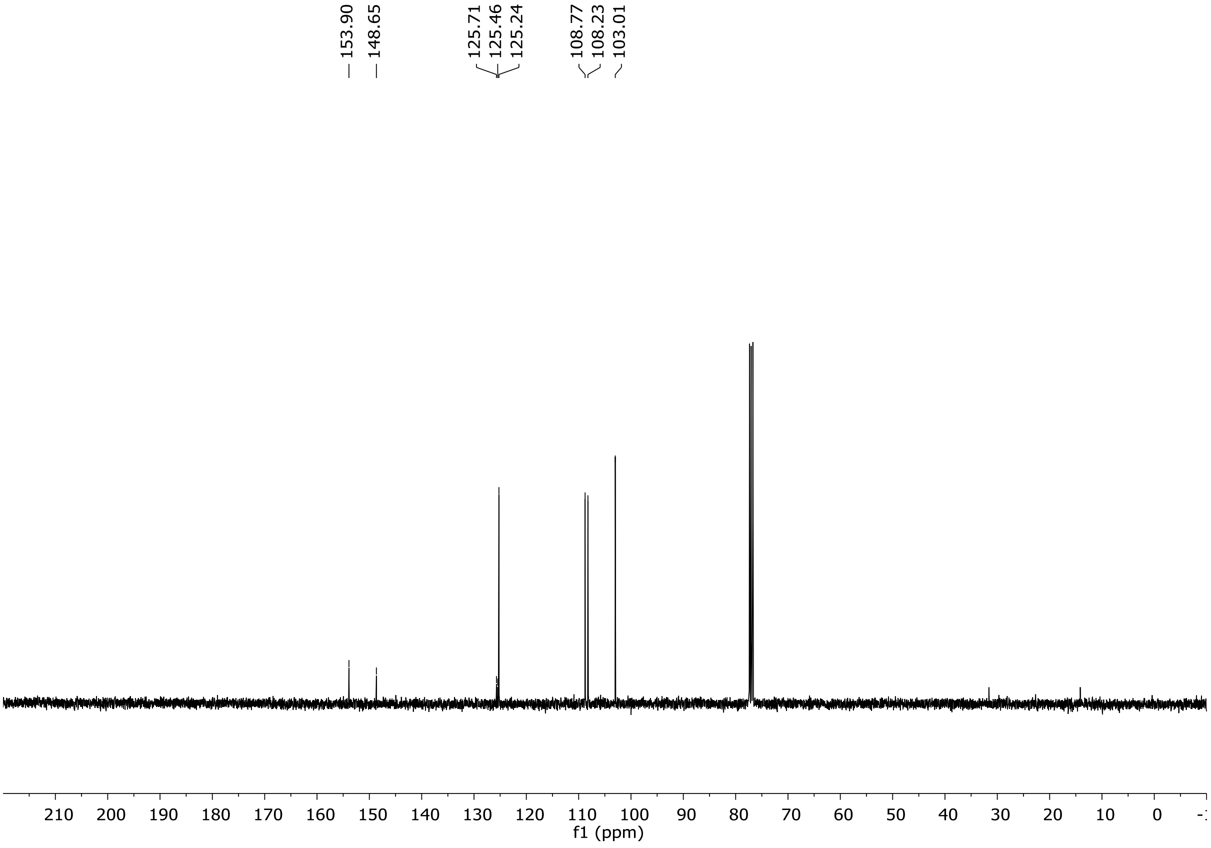
153.90
148.65
125.71
125.46
125.24
108.77
108.23
103.01
210 200 190 180 170 160 150 140 130 120 110 100 90 80 70 60 50 40 30 20 10 0
f1 (ppm)

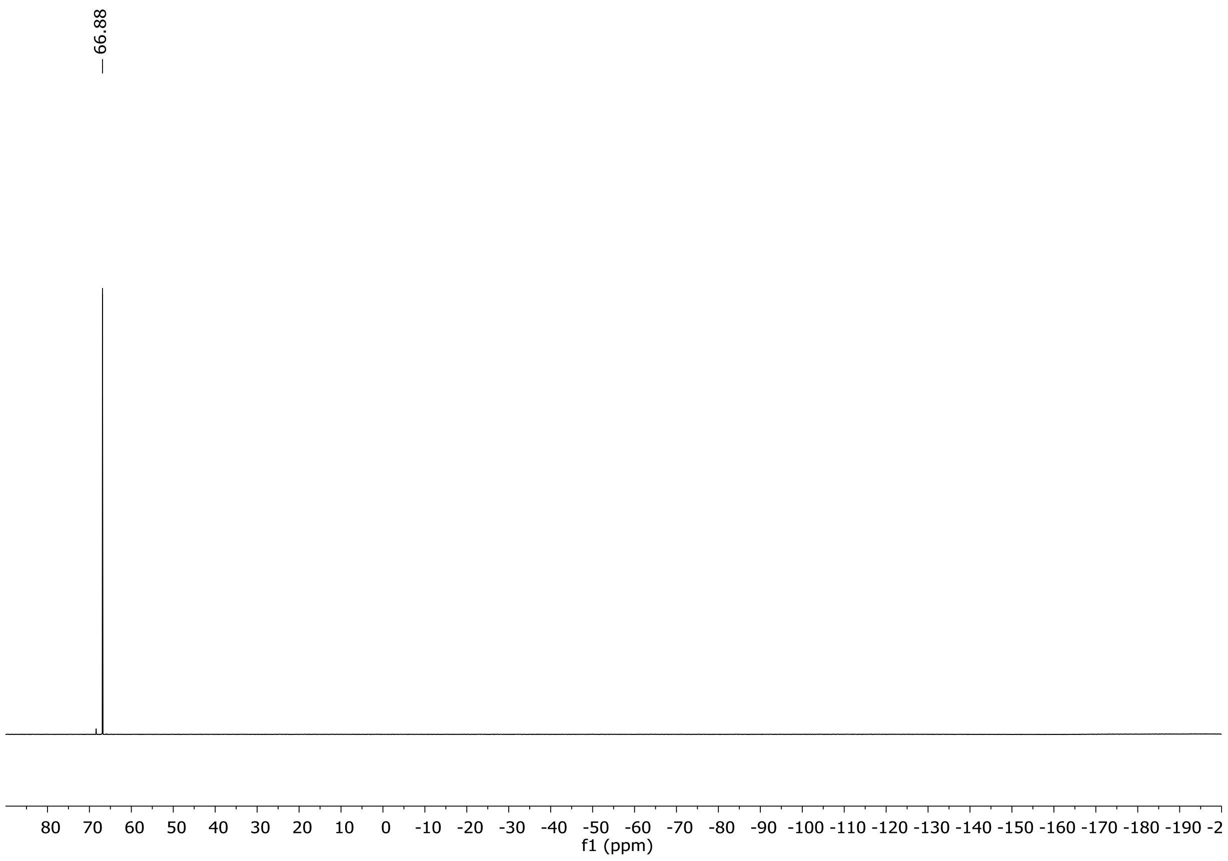

66.88
80 70 60 50 40 30 20 10 0 -10 -20 -30 -40 -50 -60 -70 -80 -90 -100 -110 -120 -130 -140 -150 -160 -170 -180 -190 -2
f1 (ppm)

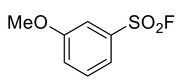
MeO
SO2F

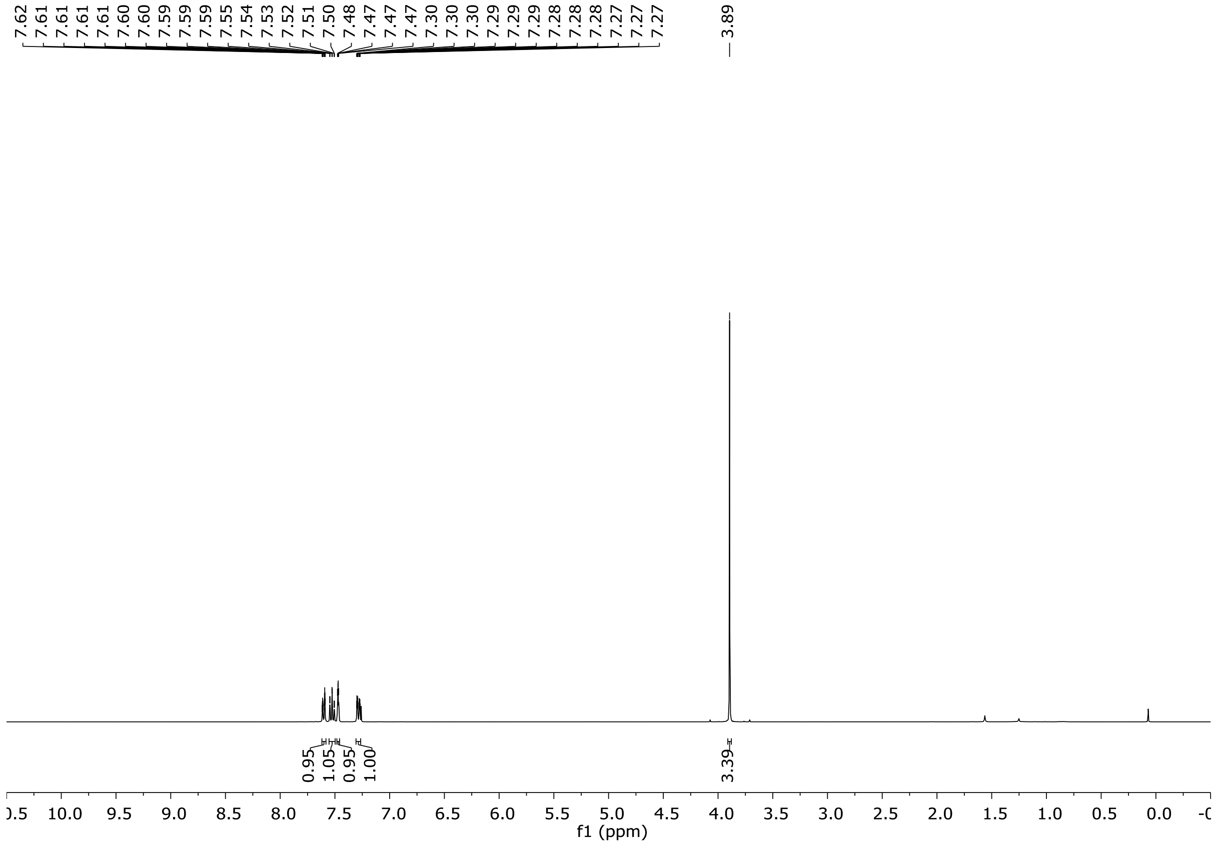
7.62 7.61 7.61 7.61 7.60 7.59 7.59 7.59 7.55 7.54 7.53 7.52 7.51 7.50 7.48 7.47 7.47 7.47 7.30 7.30 7.30 7.29 7.29 7.29 7.28 7.28 7.28 7.27 7.27 7.27
3.89
0.95
1.05
0.95
1.00
3.39
f1 (ppm)

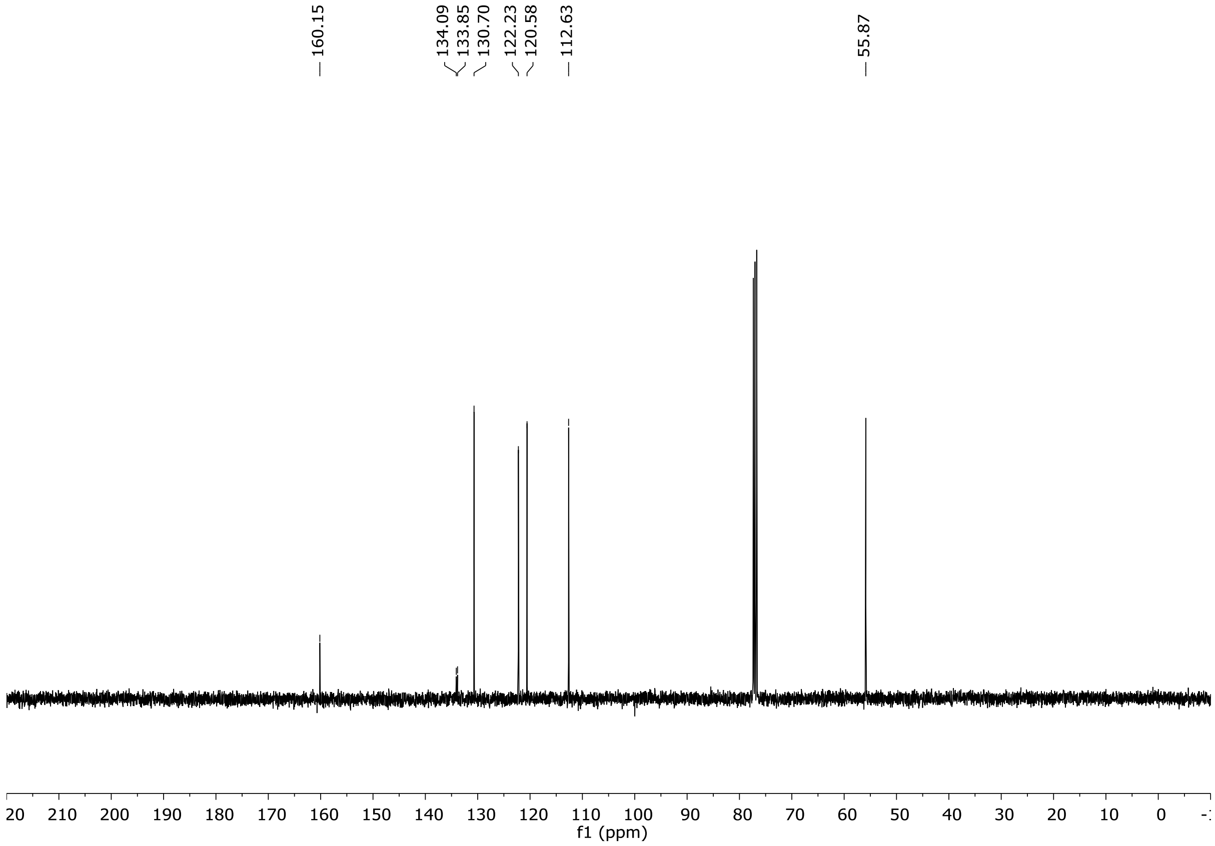
160.15
134.09
133.85
130.70
122.23
120.58
112.63
55.87
f1 (ppm)

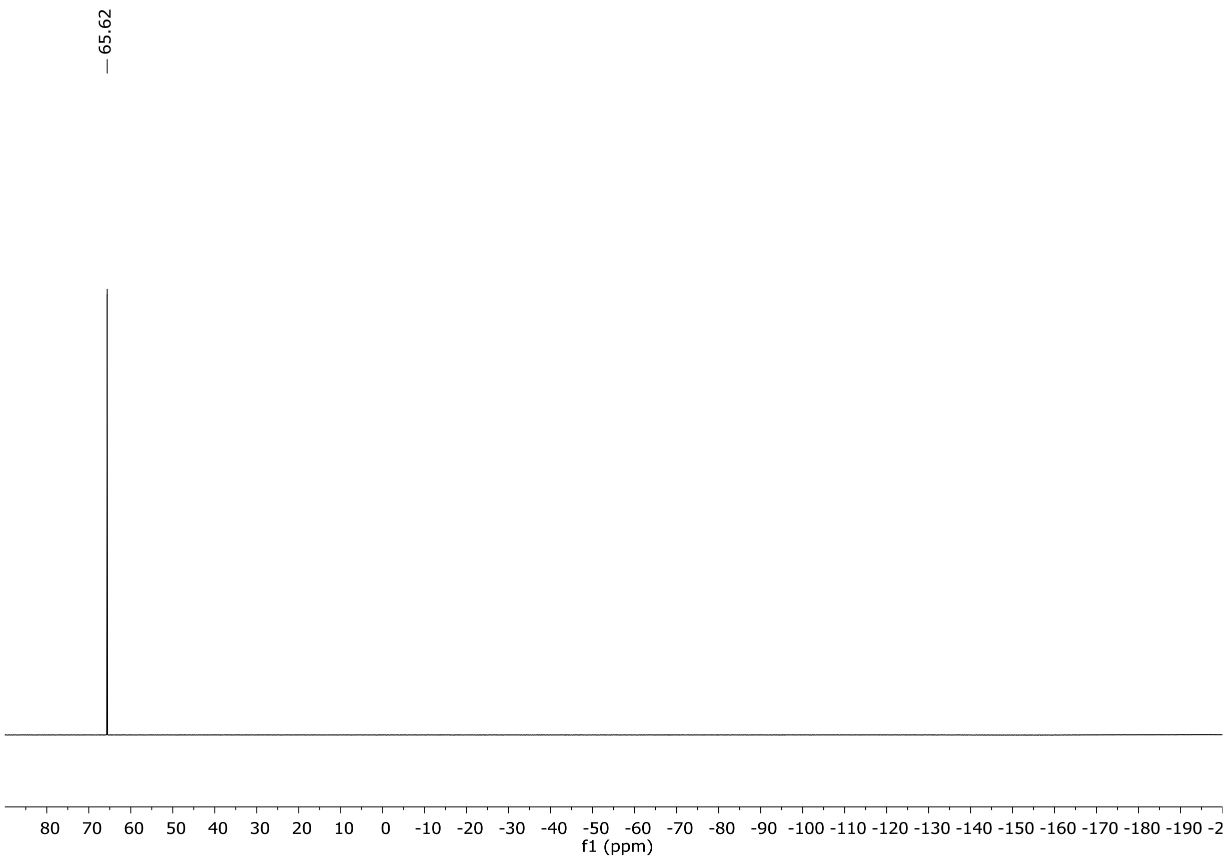

65.62
80 70 60 50 40 30 20 10 0 -10 -20 -30 -40 -50 -60 -70 -80 -90 -100 -110 -120 -130 -140 -150 -160 -170 -180 -190 -2
f1 (ppm)

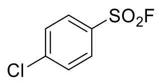
SO2F
Cl

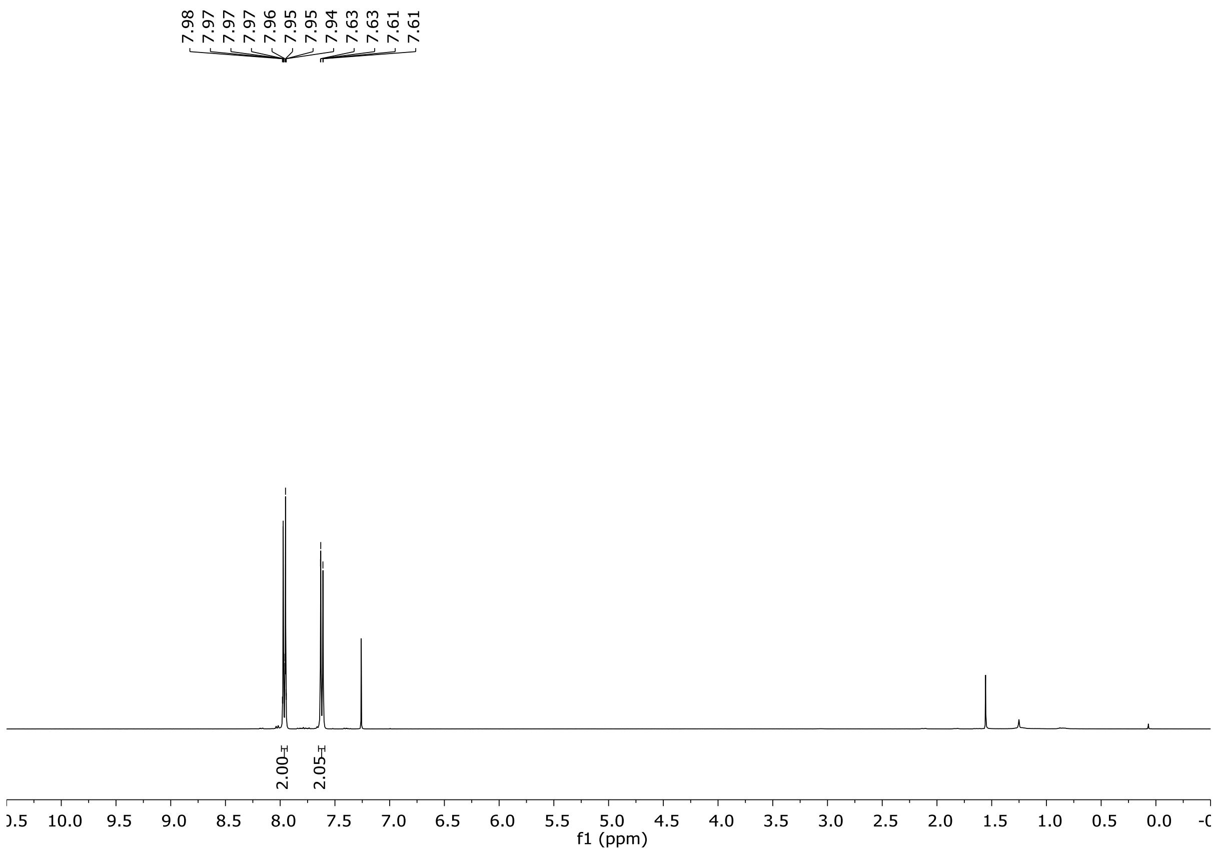
7.98
7.97
7.97
7.97
7.96
7.95
7.95
7.94
7.63
7.63
7.61
7.61
2.00
2.05
f1 (ppm)

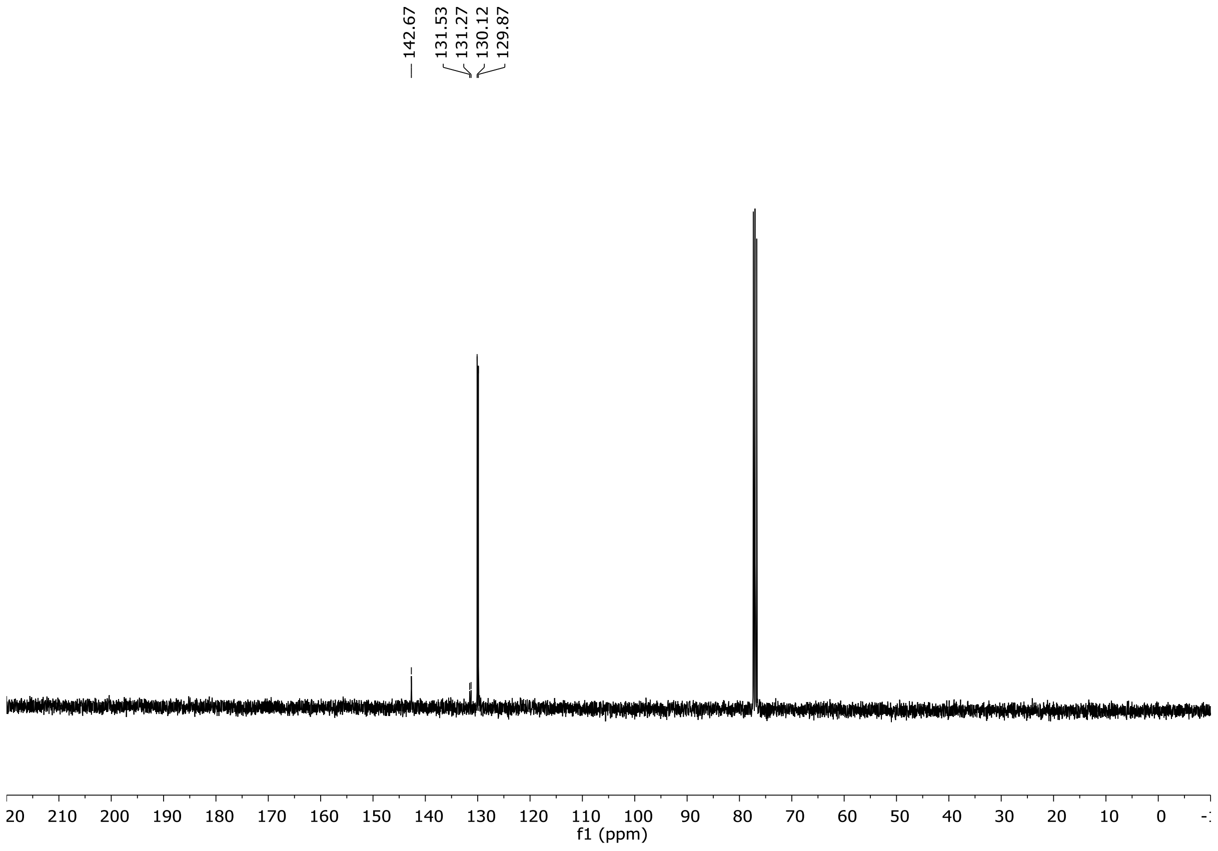
142.67
131.53
131.27
130.12
129.87
f1 (ppm)

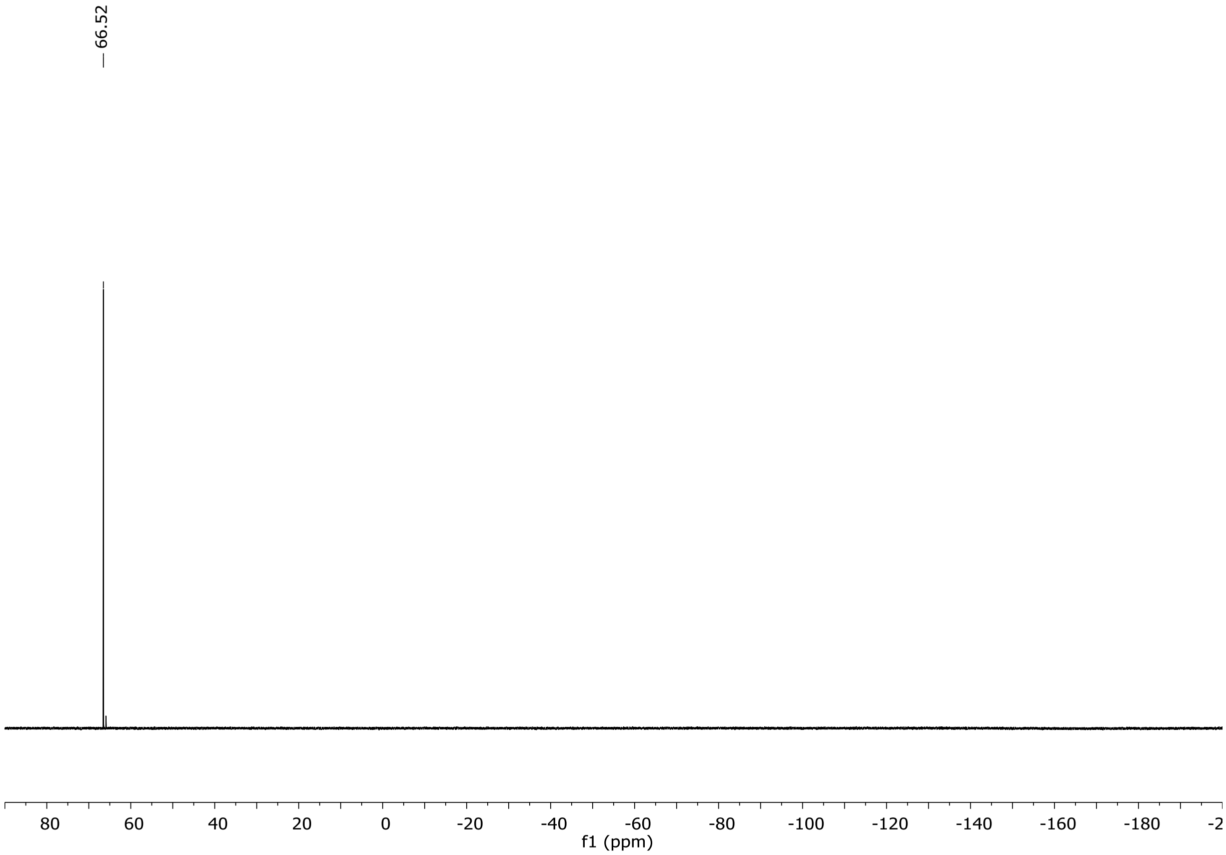

66.52
80
60
40
20
0
-20
-40
-60
-80
-100
-120
-140
-160
-180
-2
f1 (ppm)

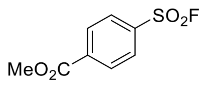
SO2F
MeO2C

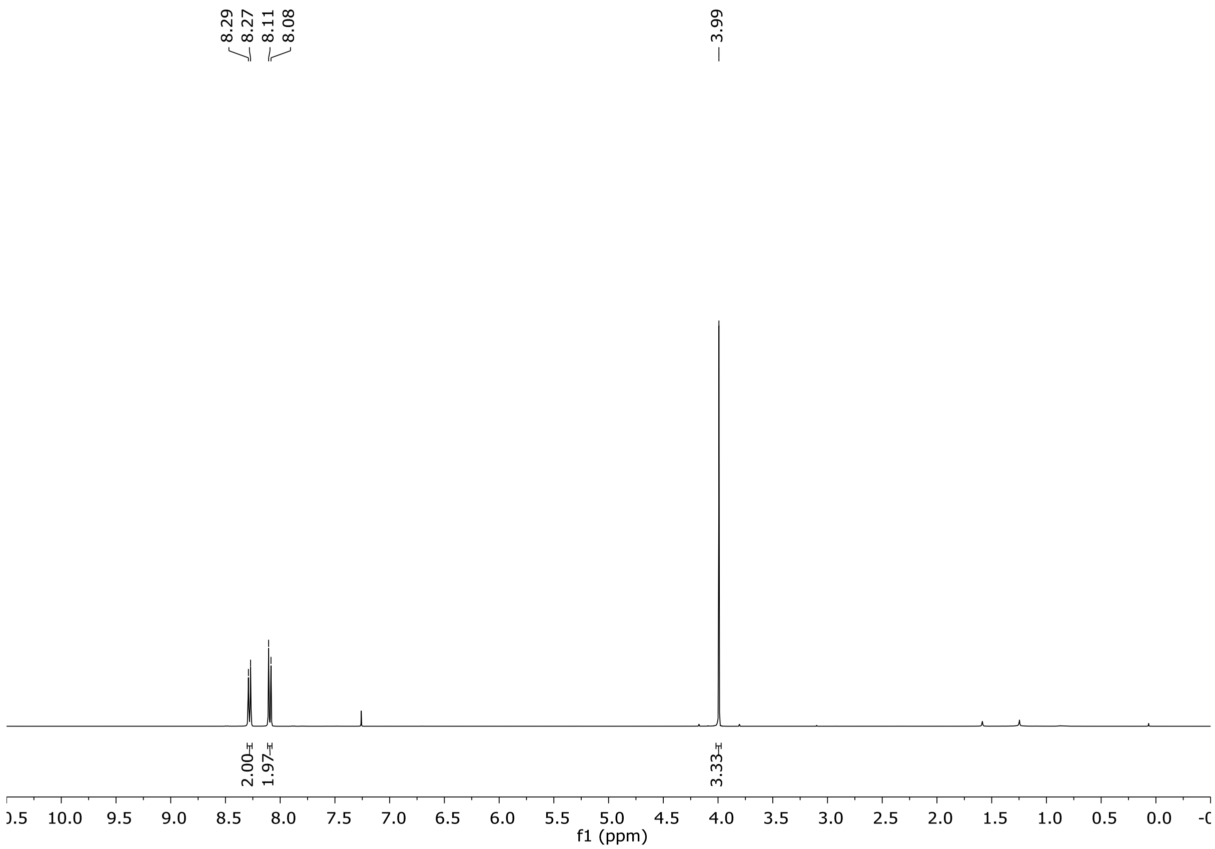
8.29
8.27
8.11
8.08
3.99
2.00
1.97
3.33
f1 (ppm)

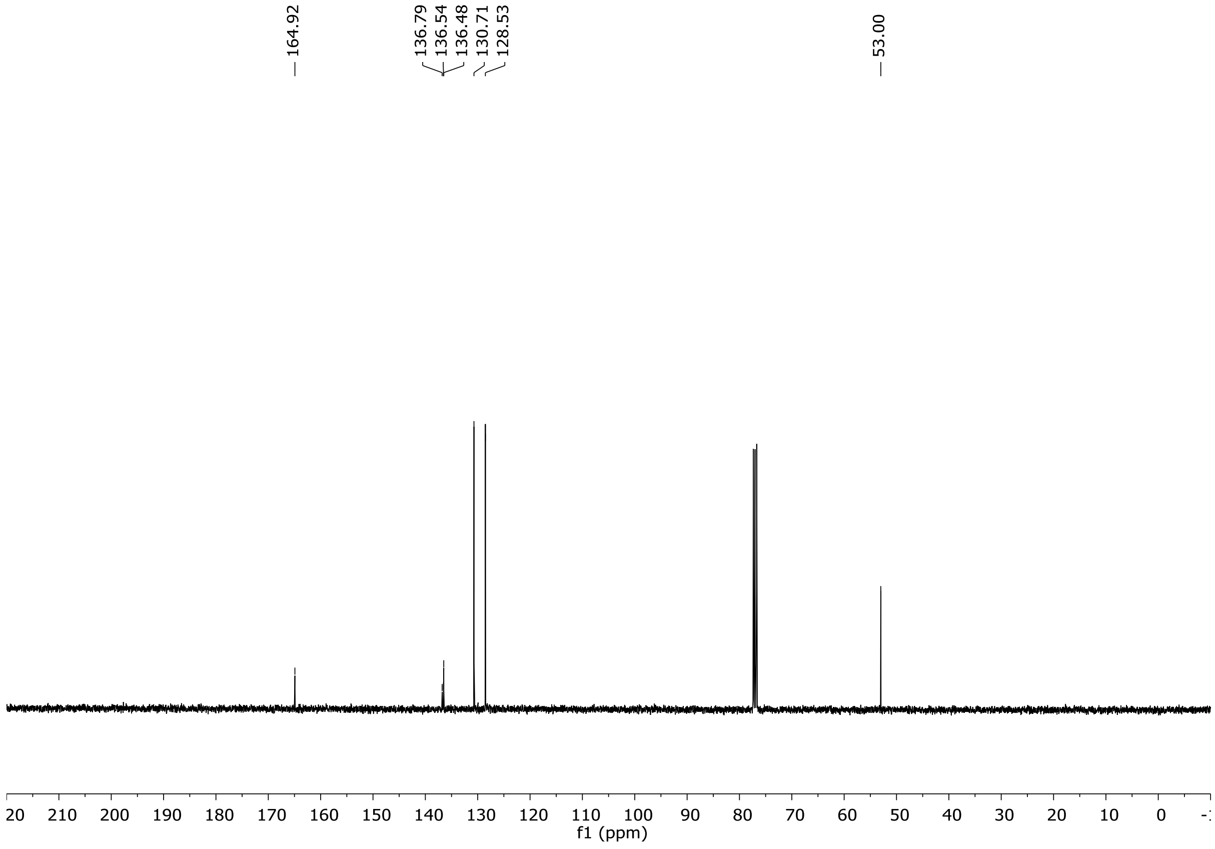
164.92
136.79
136.54
136.48
130.71
128.53
53.00
f1 (ppm)

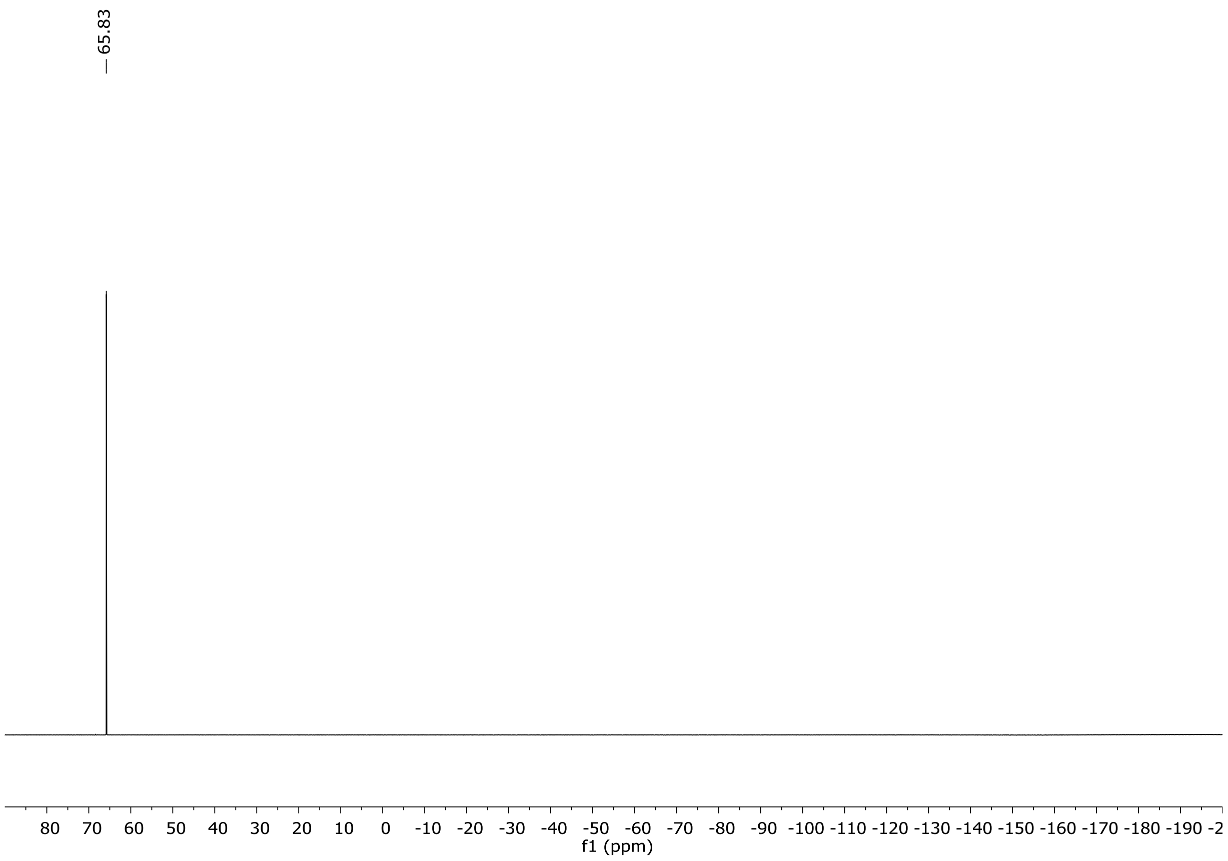
65.83
80 70 60 50 40 30 20 10 0 -10 -20 -30 -40 -50 -60 -70 -80 -90 -100 -110 -120 -130 -140 -150 -160 -170 -180 -190 -2
f1 (ppm)

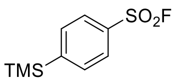
SO2F
TMS

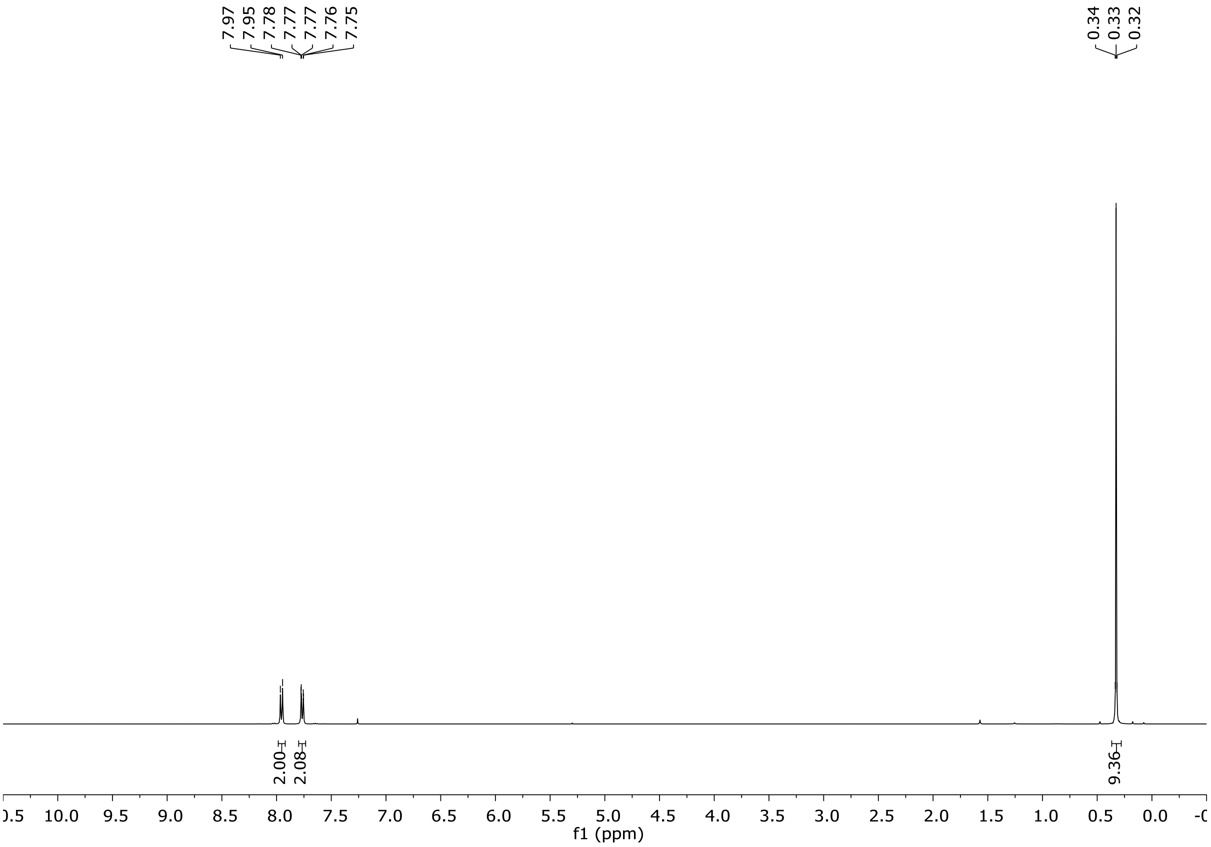
7.97
7.95
7.78
7.77
7.77
7.76
7.75
0.34
0.33
0.32
2.00
2.08
9.36
f1 (ppm)

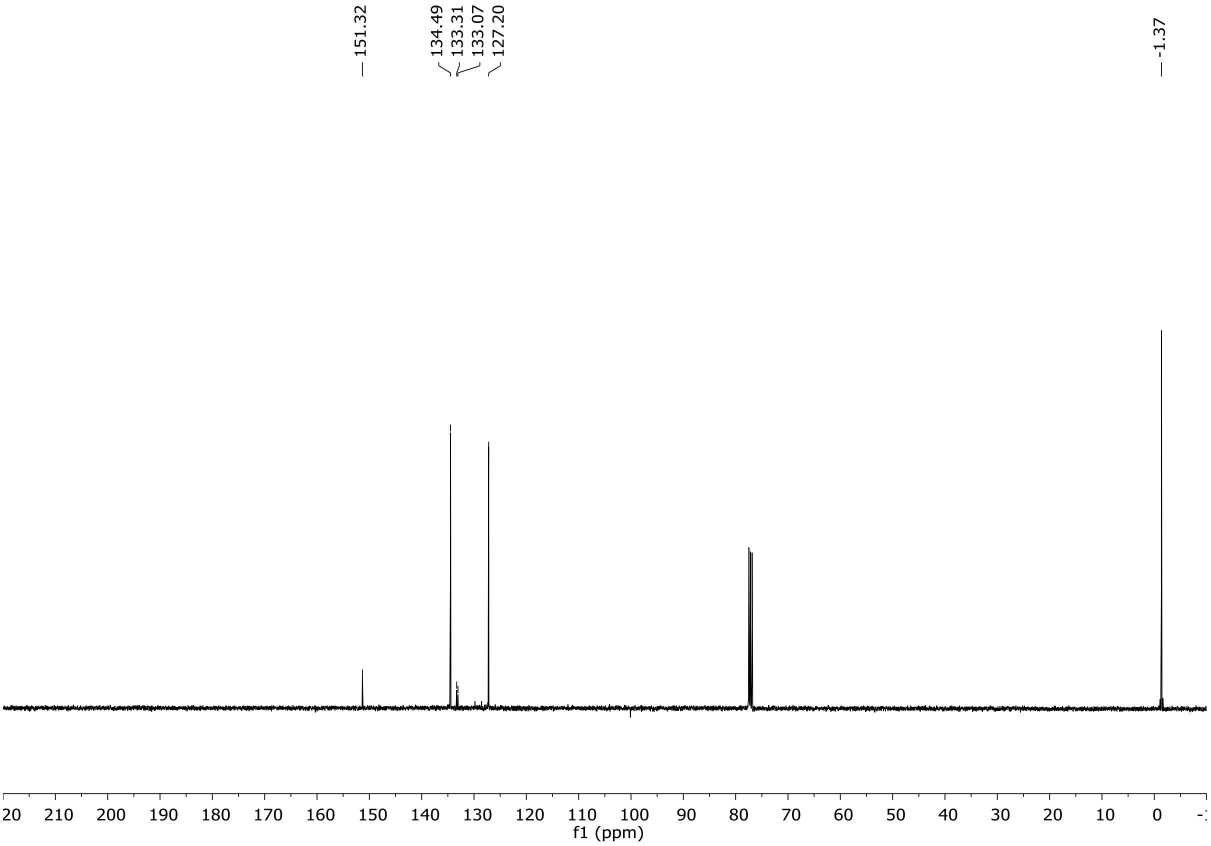
151.32
134.49
133.31
133.07
127.20
-1.37
f1 (ppm)

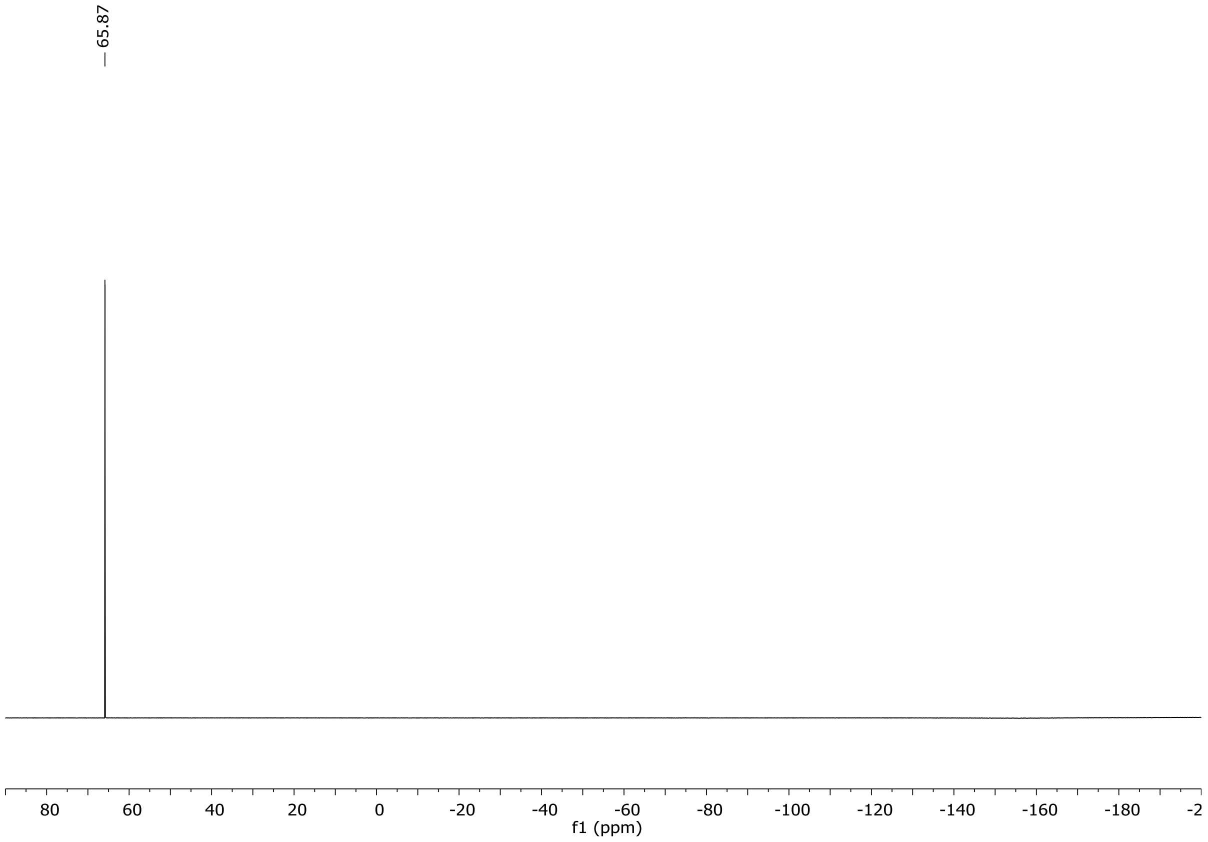
65.87
49
80 60 40 20 0 -20 -40 -60 -80 -100 -120 -140 -160 -180 -2
f1 (ppm)

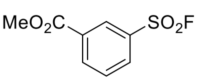
MeO$_2$C
SO$_2$F

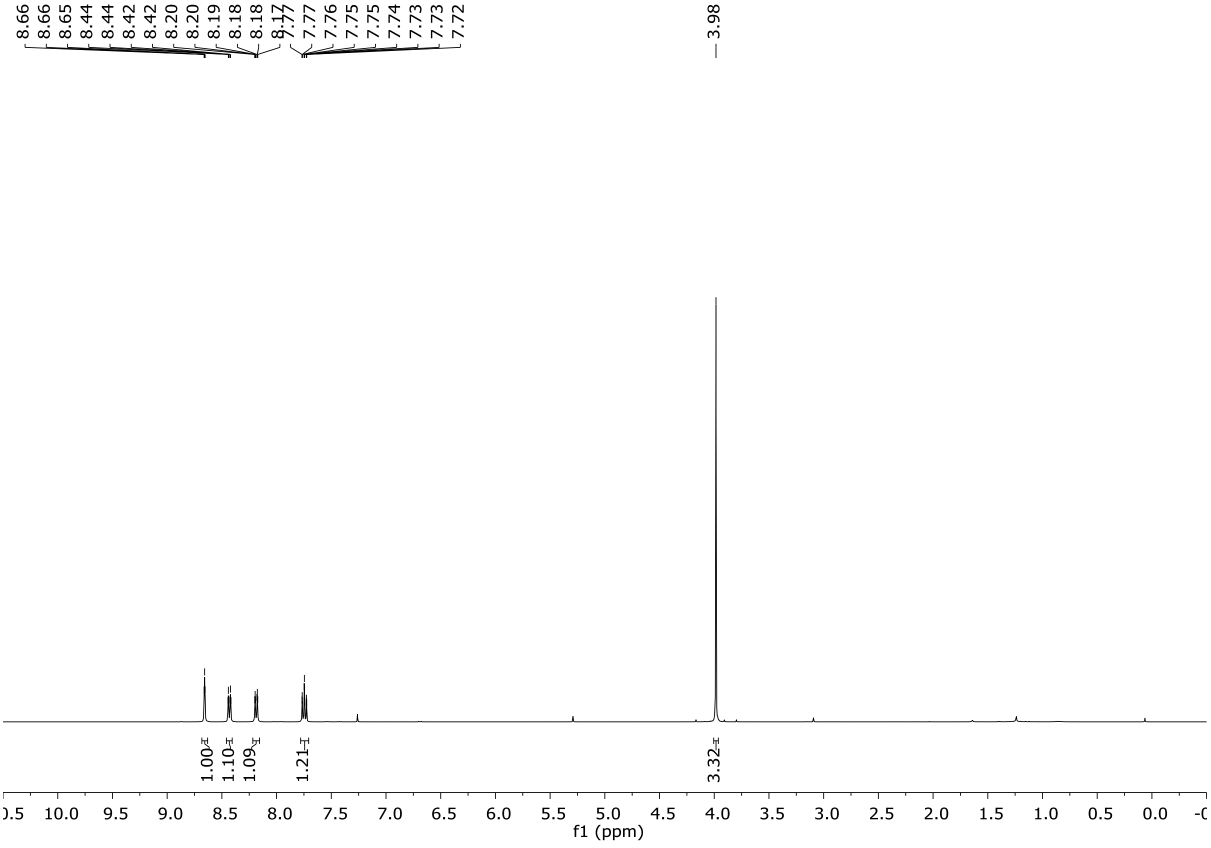
8.66
8.66
8.65
8.44
8.44
8.42
8.42
8.20
8.20
8.19
8.18
8.18
8.17
7.77
7.77
7.76
7.75
7.75
7.74
7.73
7.73
7.72
3.98
1.00
1.10
1.09
1.21
3.32
f1 (ppm)

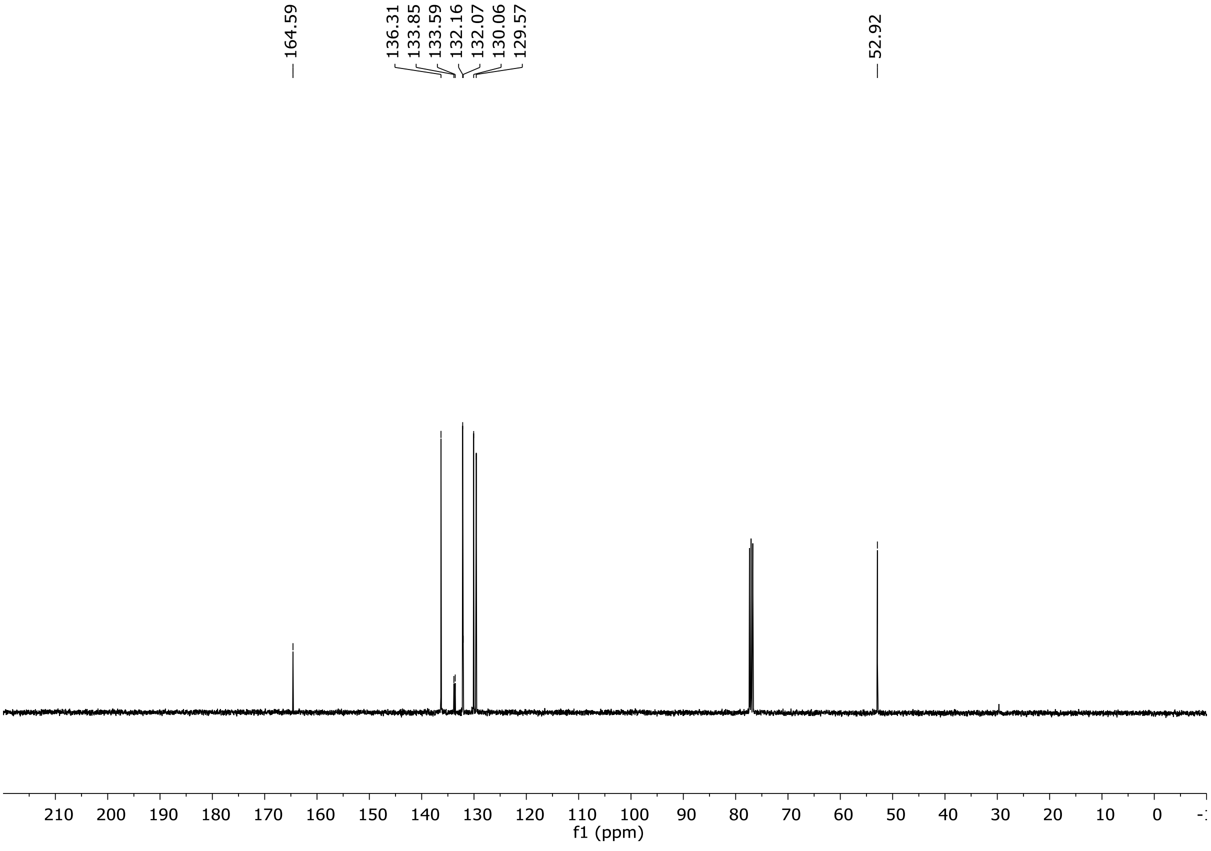
164.59
136.31
133.85
133.59
132.16
132.07
130.06
129.57
52.92
f1 (ppm)

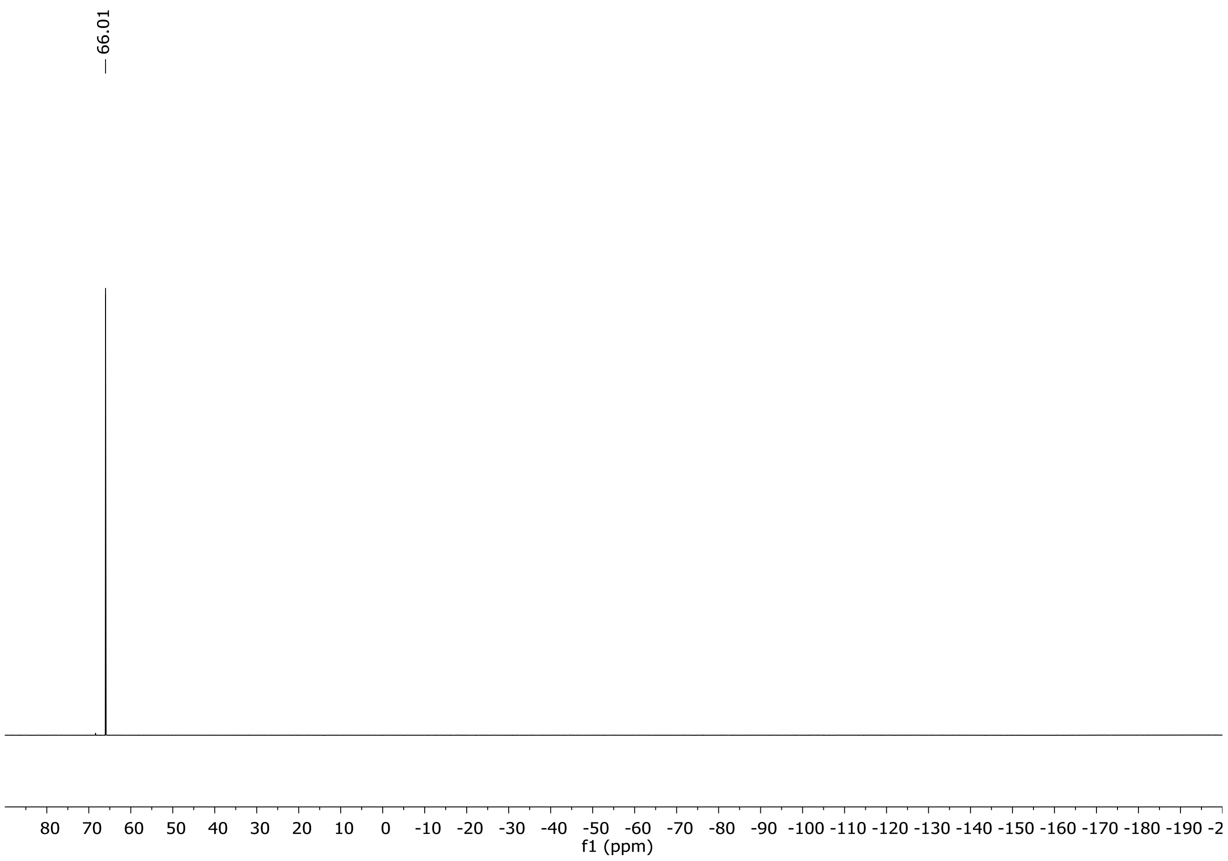
66.01
80 70 60 50 40 30 20 10 0 -10 -20 -30 -40 -50 -60 -70 -80 -90 -100 -110 -120 -130 -140 -150 -160 -170 -180 -190 -2
f1 (ppm)

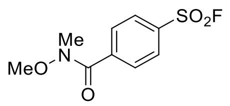
SO2F
Me
N
MeO
O

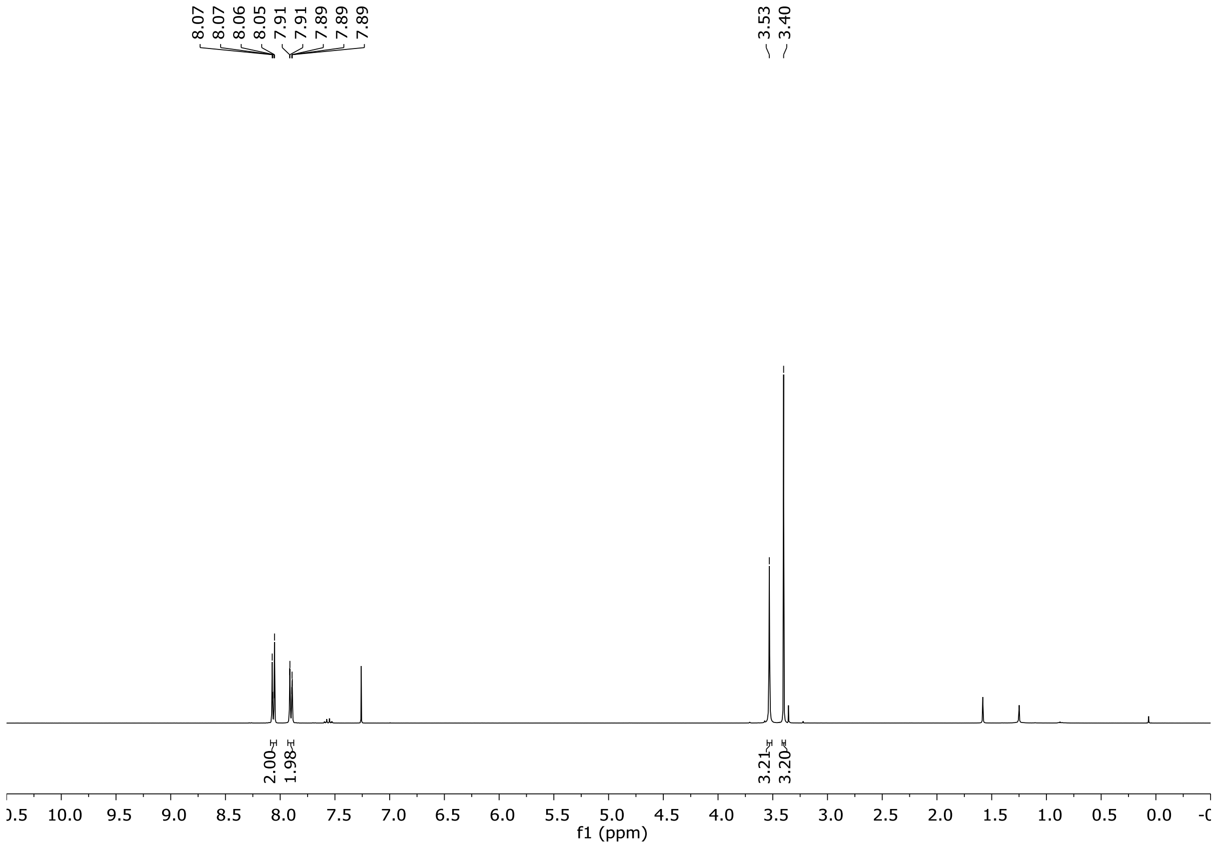
8.07
8.07
8.06
8.05
7.91
7.91
7.89
7.89
7.89
3.53
3.40
2.00
1.98
3.21
3.20
f1 (ppm)

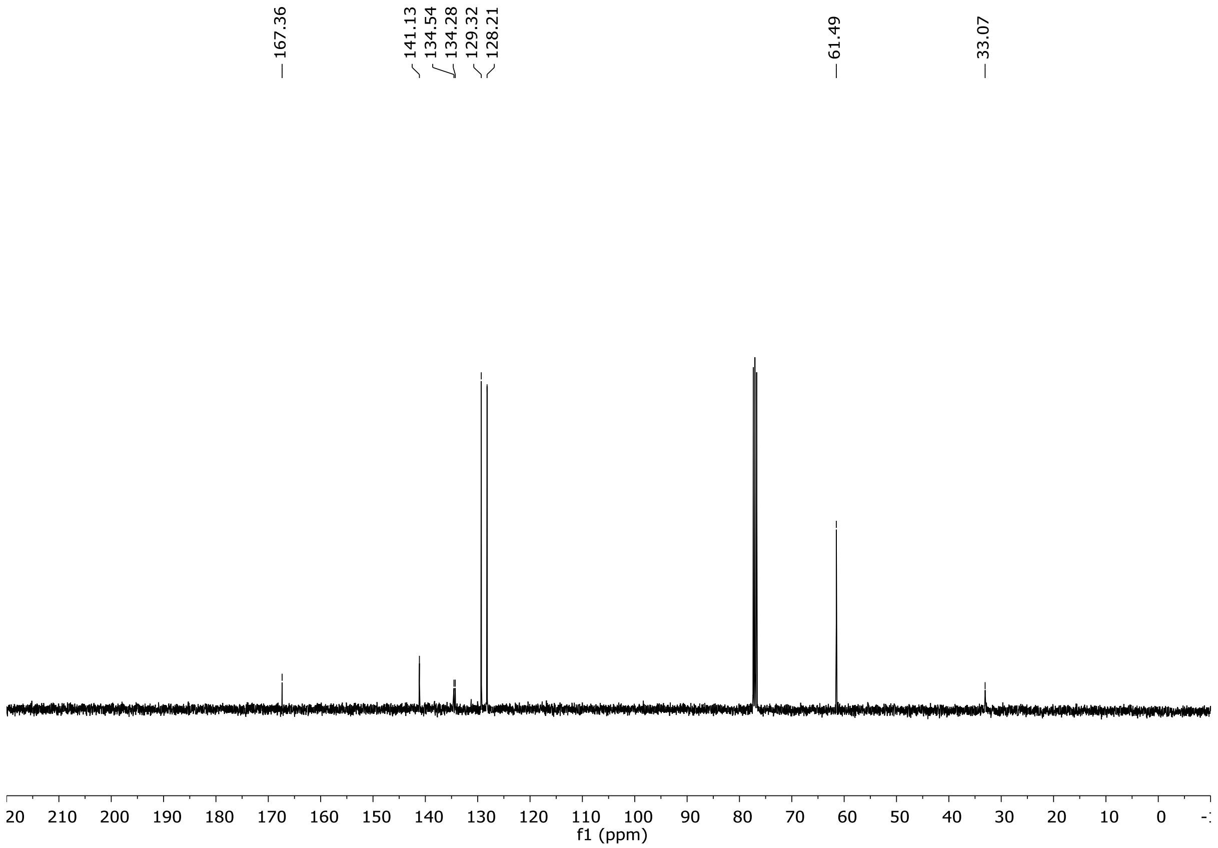
167.36
141.13
134.54
134.28
129.32
128.21
61.49
33.07
f1 (ppm)

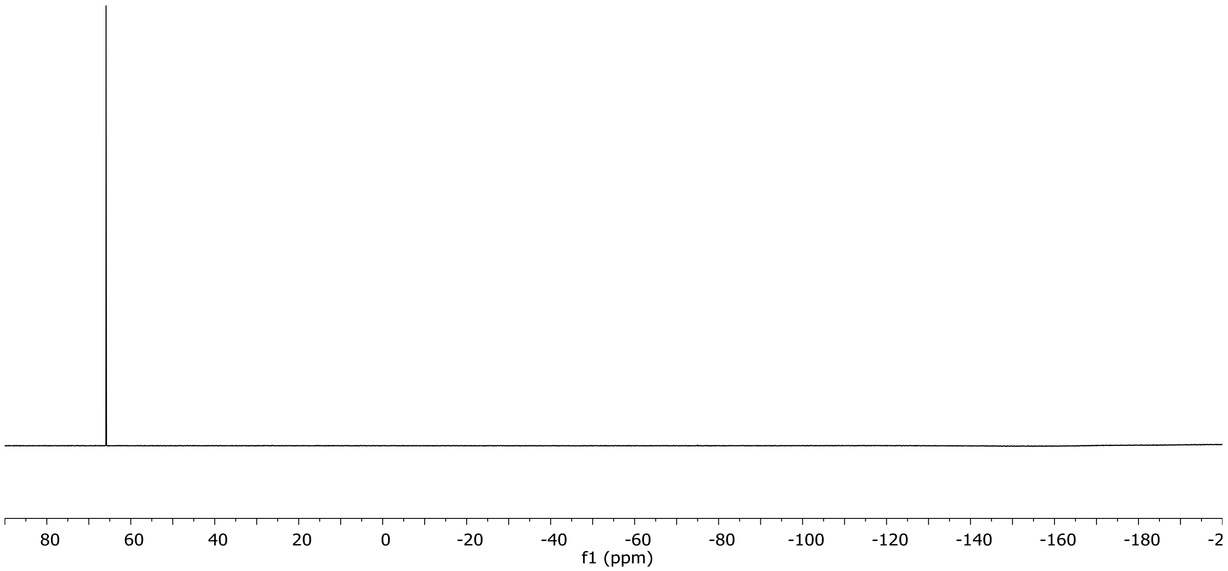
80 60 40 20 0 -20 -40 -60 -80 -100 -120 -140 -160 -180 -2
f1 (ppm)

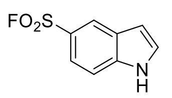
FO$_2$S
N
H

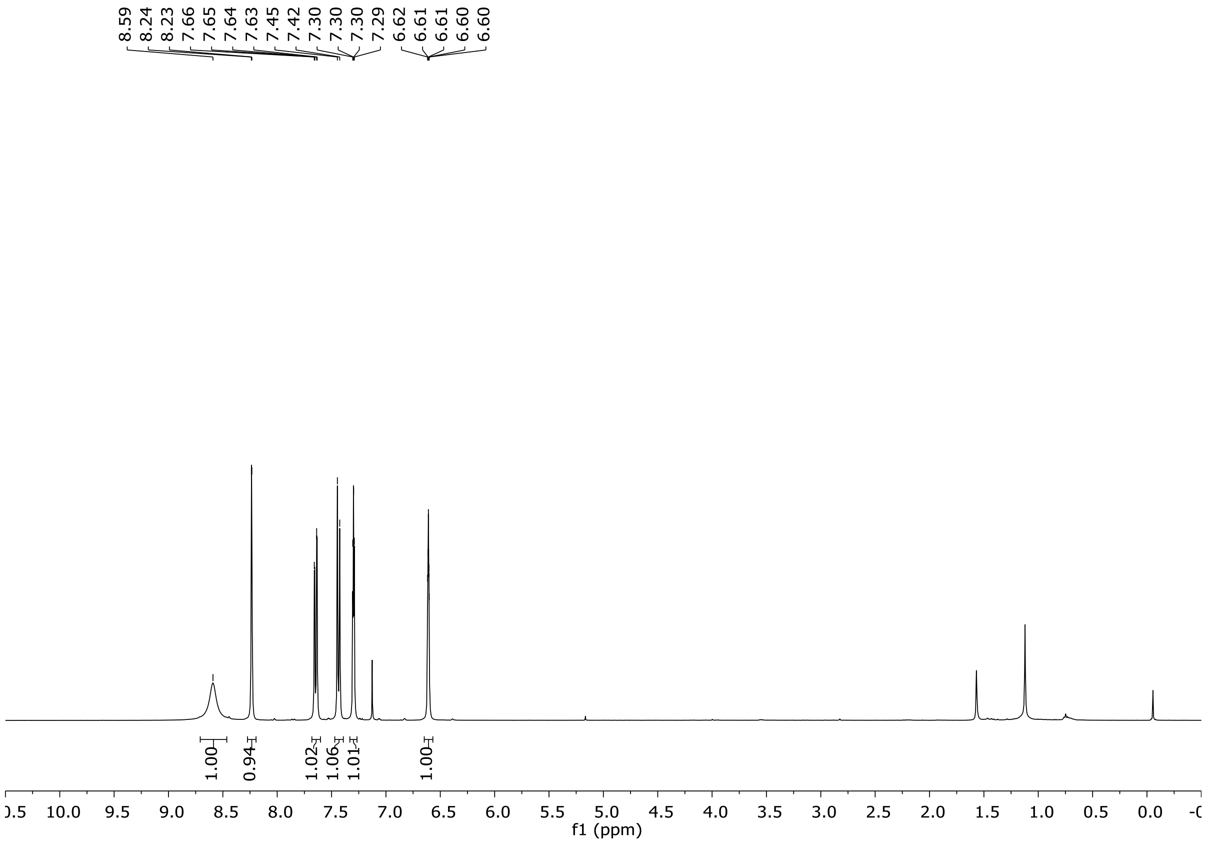
8.59
8.24
8.23
7.66
7.65
7.64
7.63
7.45
7.42
7.30
7.30
7.30
7.29
6.62
6.61
6.61
6.60
6.60
1.00
0.94
1.02
1.06
1.01
1.00
f1 (ppm)

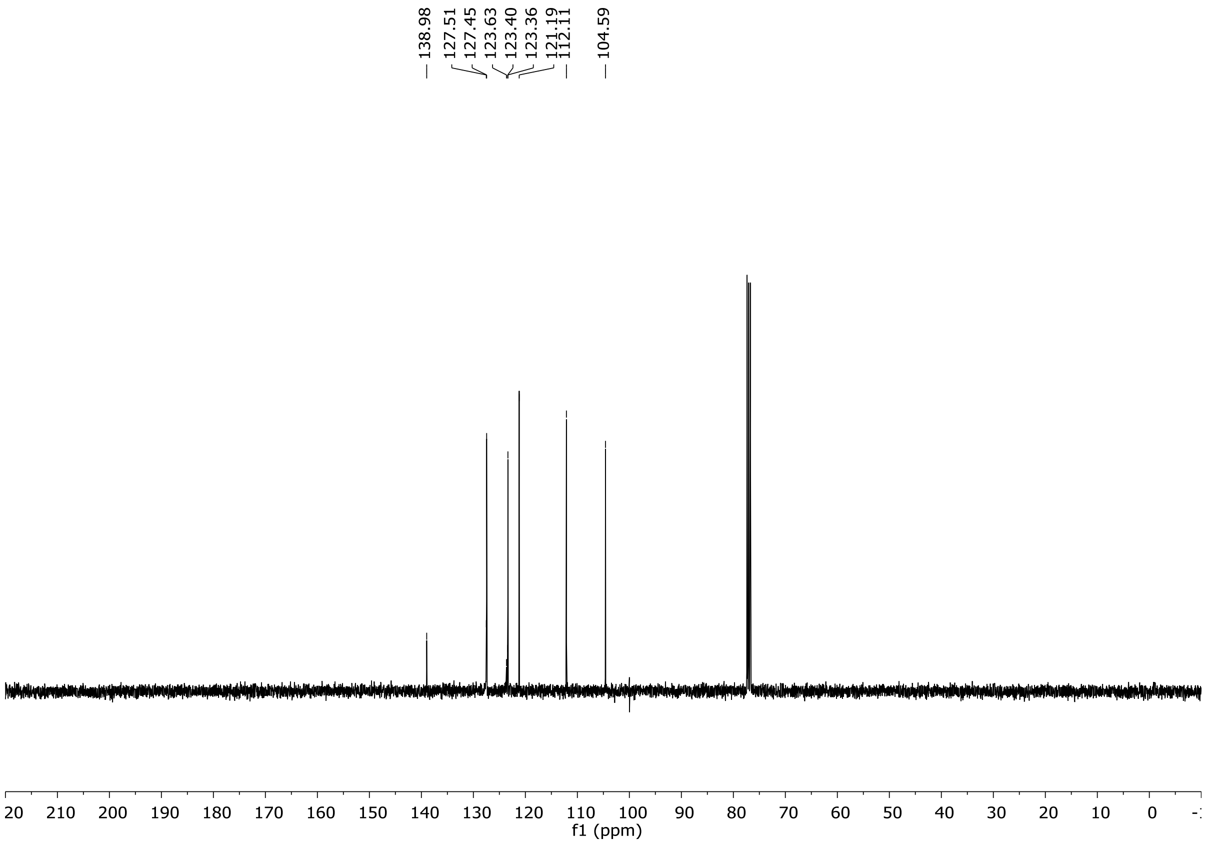
138.98
127.51
127.45
123.63
123.40
123.36
121.19
112.11
104.59
f1 (ppm)

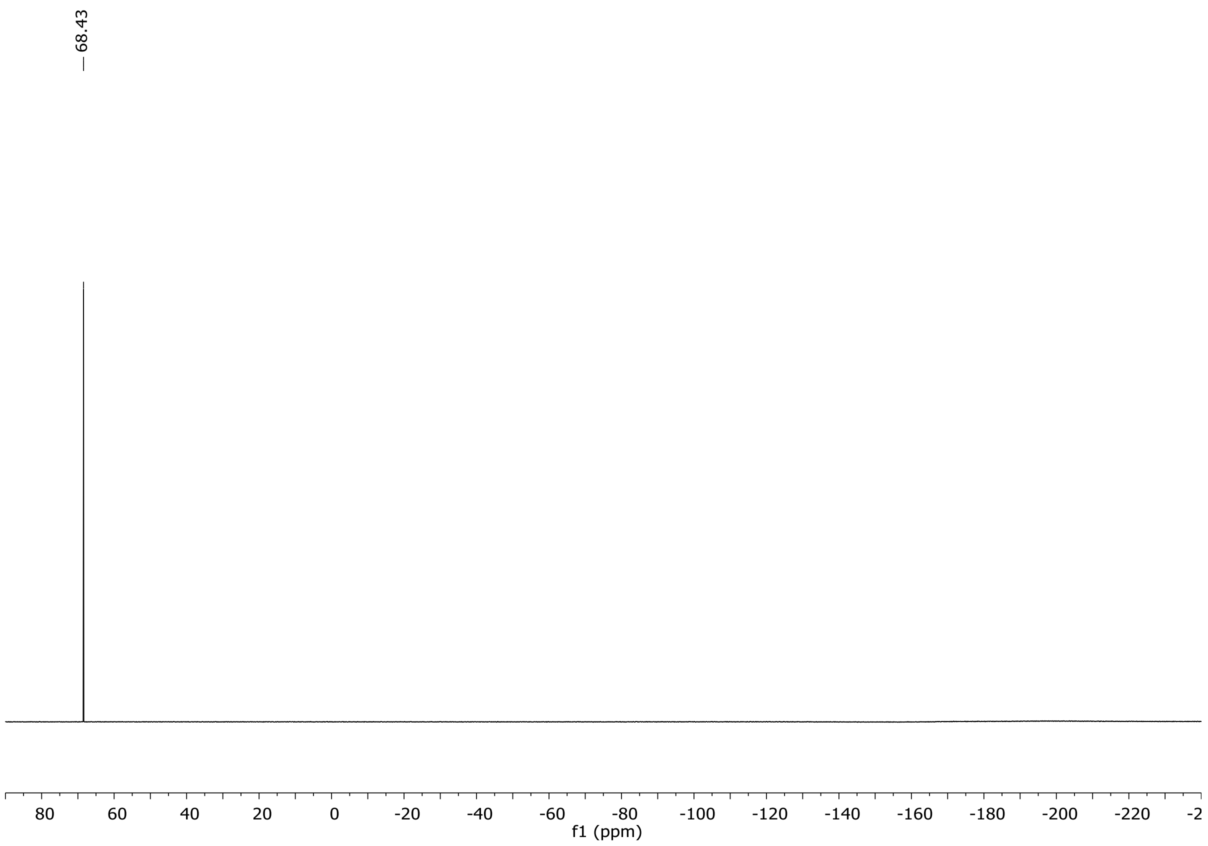
68.43
80
60
40
20
0
-20
-40
-60
-80
-100
-120
-140
-160
-180
-200
-220
-2
f1 (ppm)

3.3 NMR Spectra of Pyridyl Sulfonyl Fluorides Synthesized by Palladium Catalysis

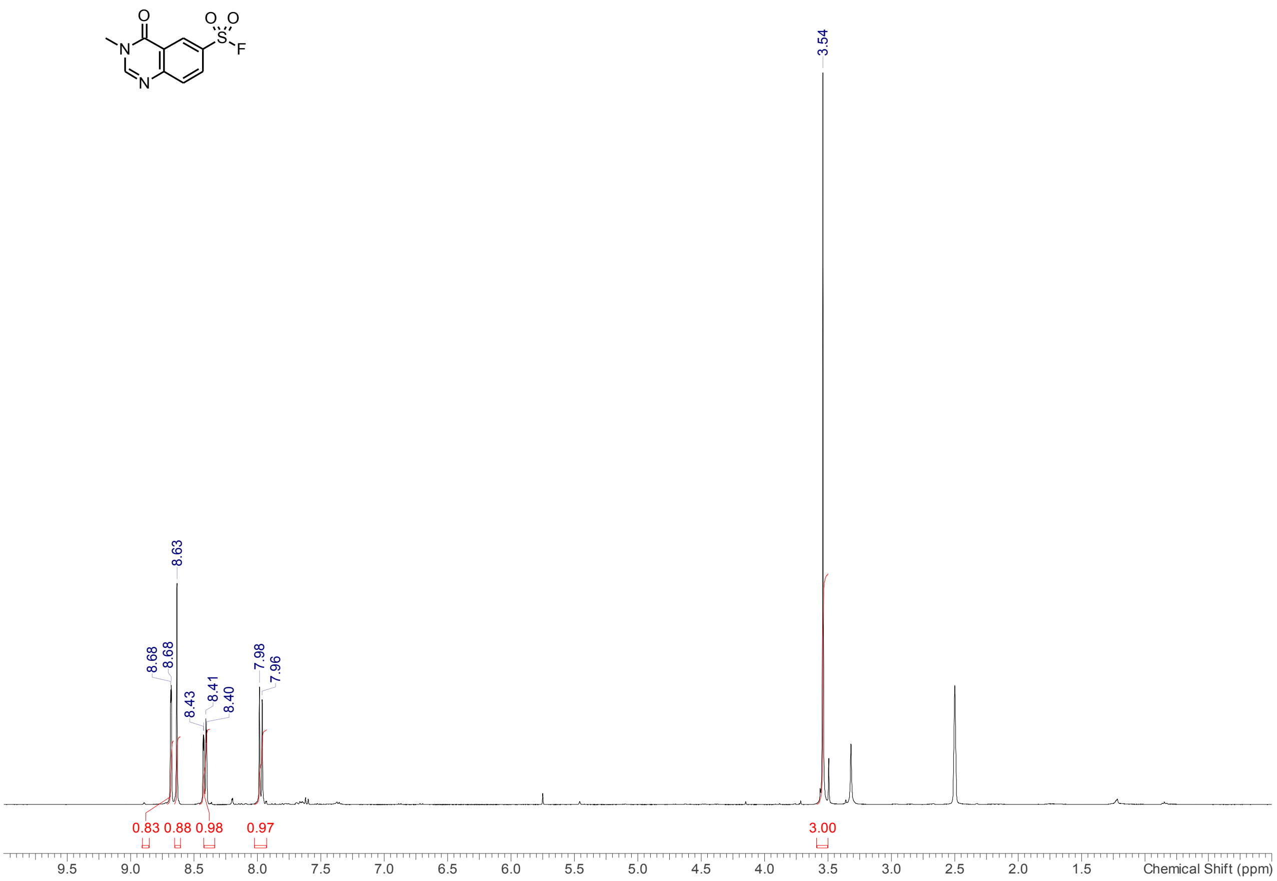

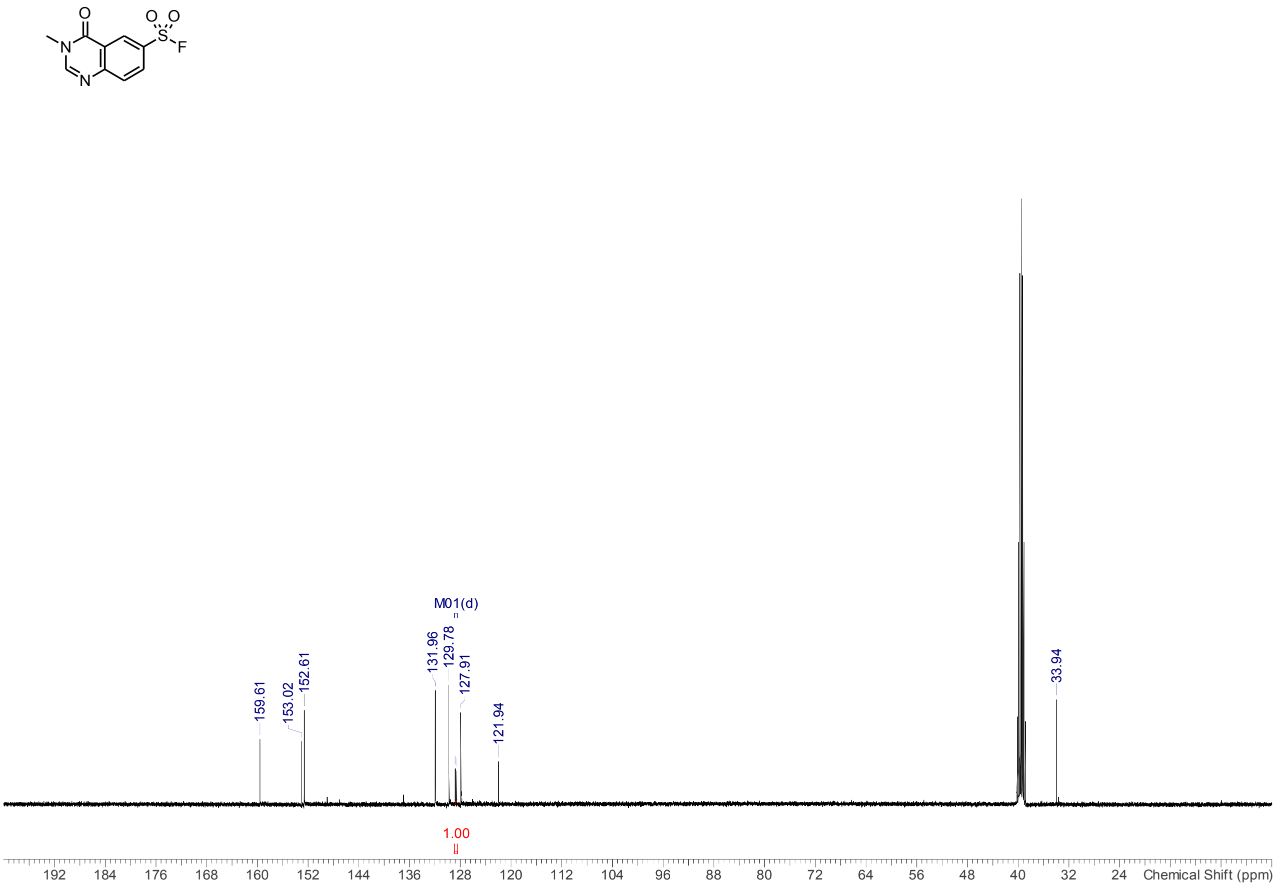

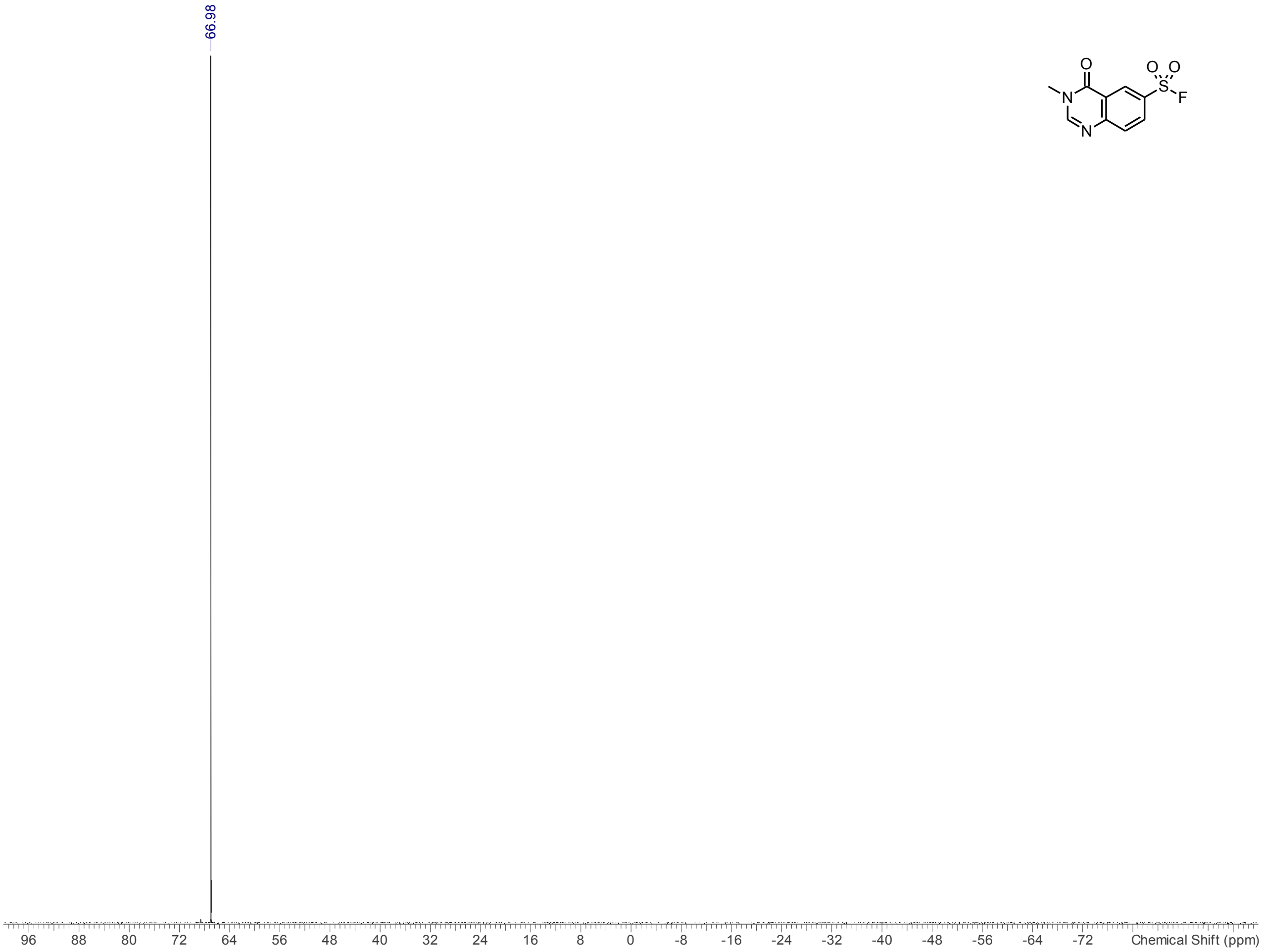
66.98
96 88 80 72 64 56 48 40 32 24 16 8 0 -8 -16 -24 -32 -40 -48 -56 -64 -72
Chemical Shift (ppm)

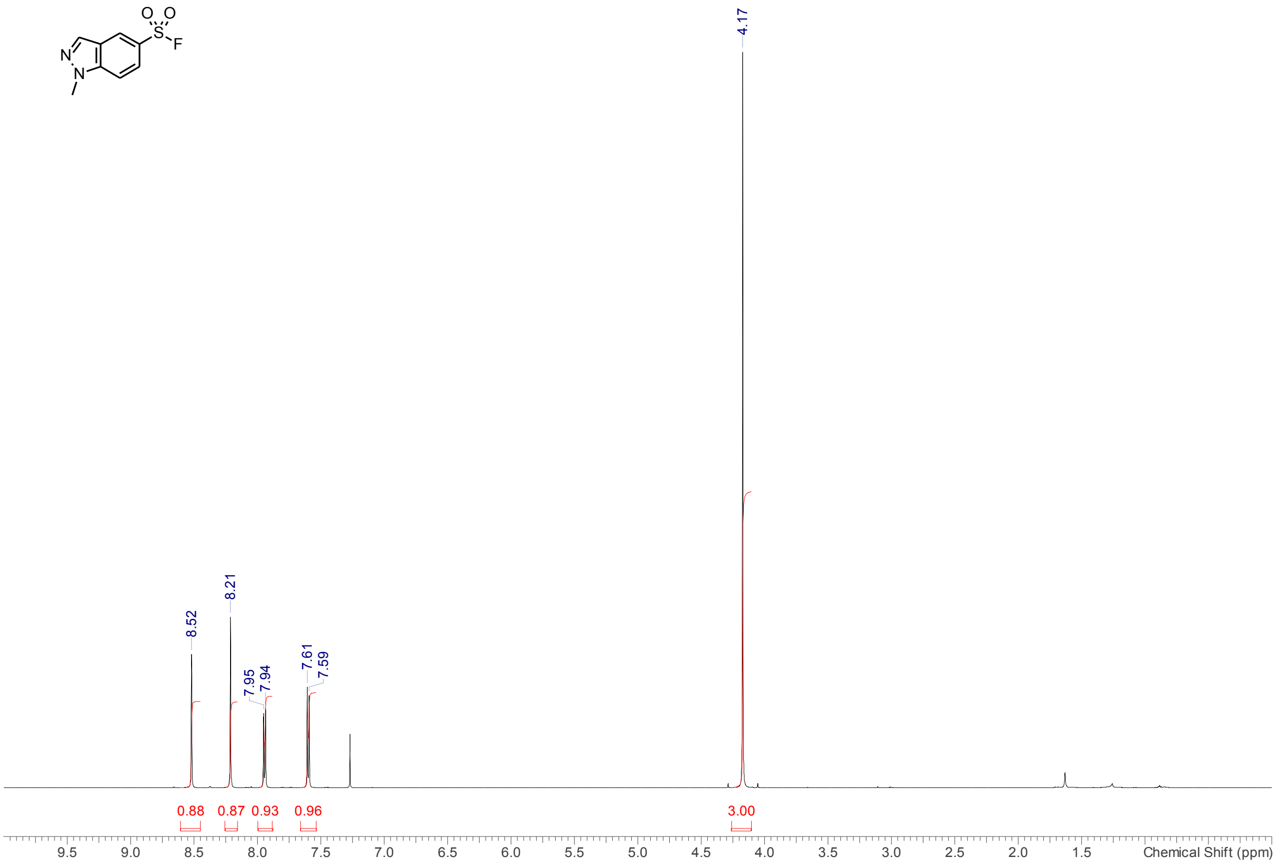
O O
S
F
N
N
8.52
8.21
7.95
7.94
7.61
7.59
4.17
0.88
0.87
0.93
0.96
3.00
9.5 9.0 8.5 8.0 7.5 7.0 6.5 6.0 5.5 5.0 4.5 4.0 3.5 3.0 2.5 2.0 1.5
Chemical Shift (ppm)

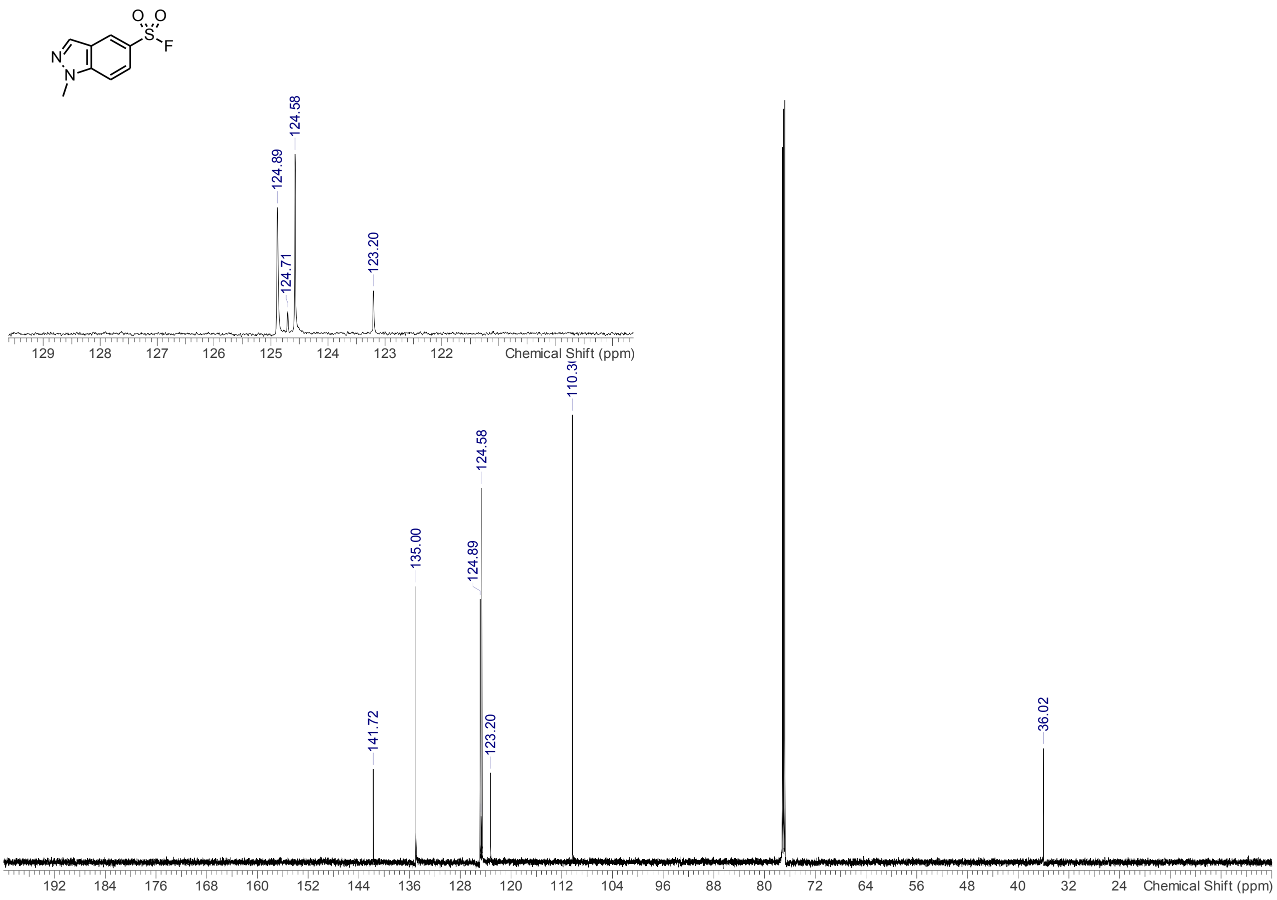
O O
S
F
N
N
124.58
124.89
124.71
123.20
129 128 127 126 125 124 123 122
Chemical Shift (ppm)
110.31
124.58
135.00
124.89
141.72
123.20
36.02
192 184 176 168 160 152 144 136 128 120 112 104 96 88 80 72 64 56 48 40 32 24
Chemical Shift (ppm)

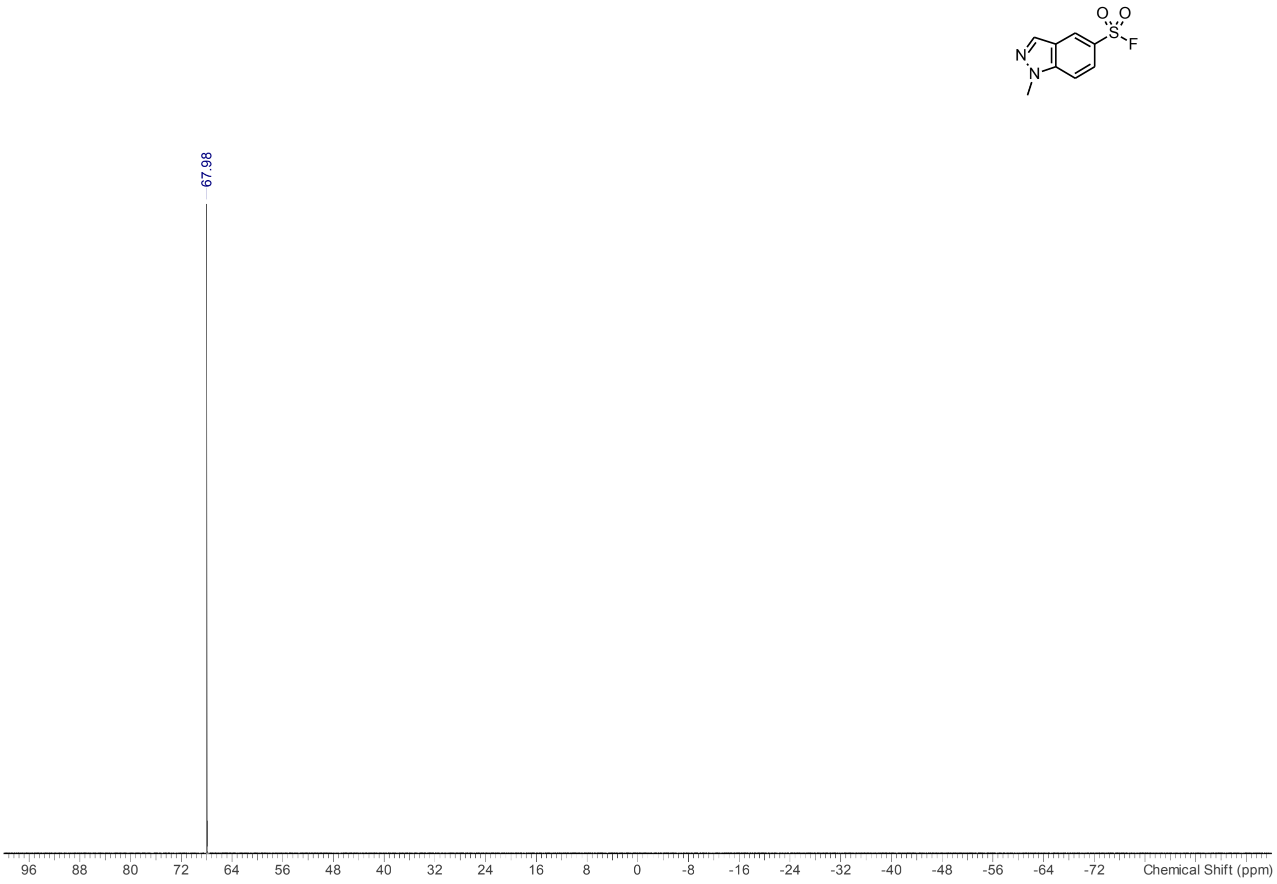
O O
S
F
N
N
67.98
96 88 80 72 64 56 48 40 32 24 16 8 0 -8 -16 -24 -32 -40 -48 -56 -64 -72
Chemical Shift (ppm)

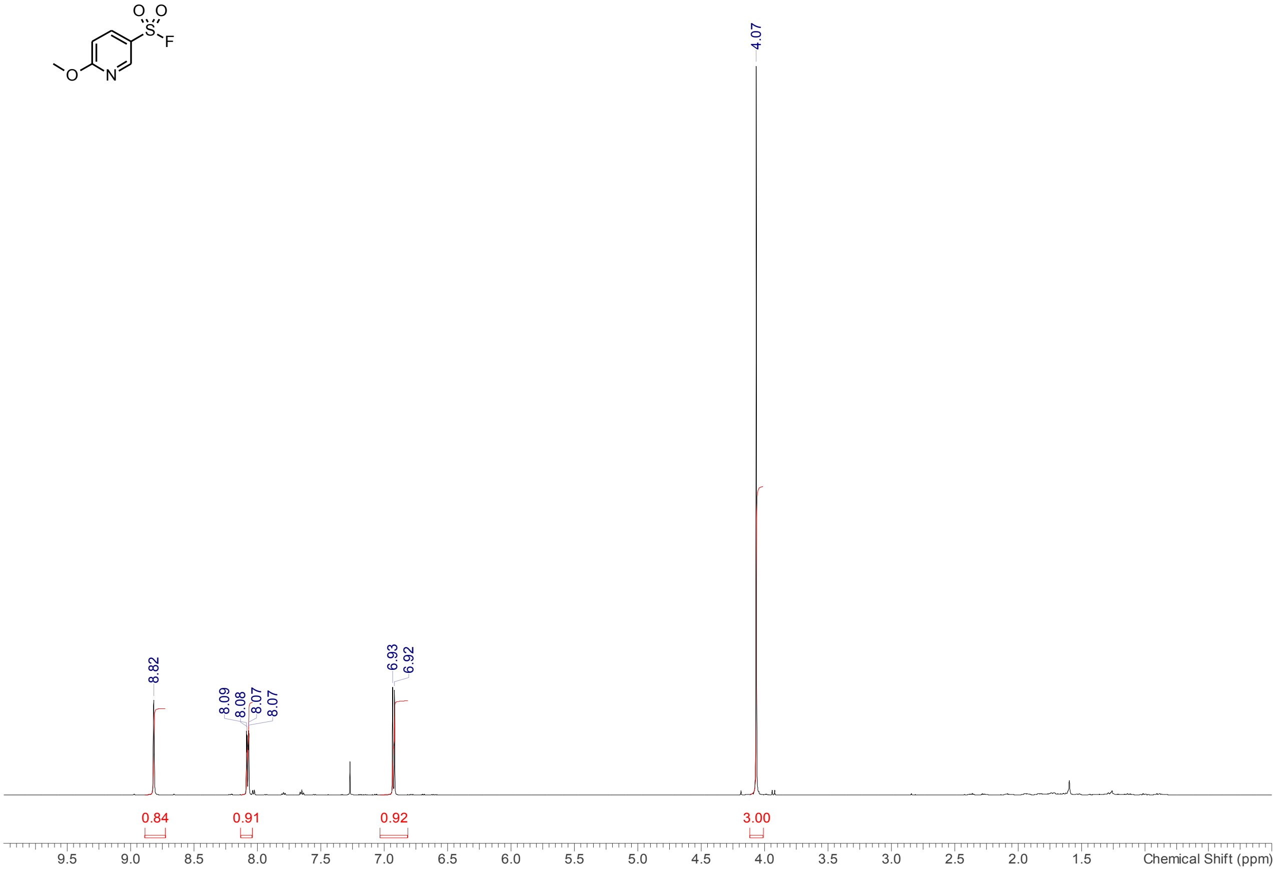
O O
S
F
O
N
8.82
8.09
8.08
8.07
8.07
6.93
6.92
4.07
0.84
0.91
0.92
3.00
9.5 9.0 8.5 8.0 7.5 7.0 6.5 6.0 5.5 5.0 4.5 4.0 3.5 3.0 2.5 2.0 1.5
Chemical Shift (ppm)

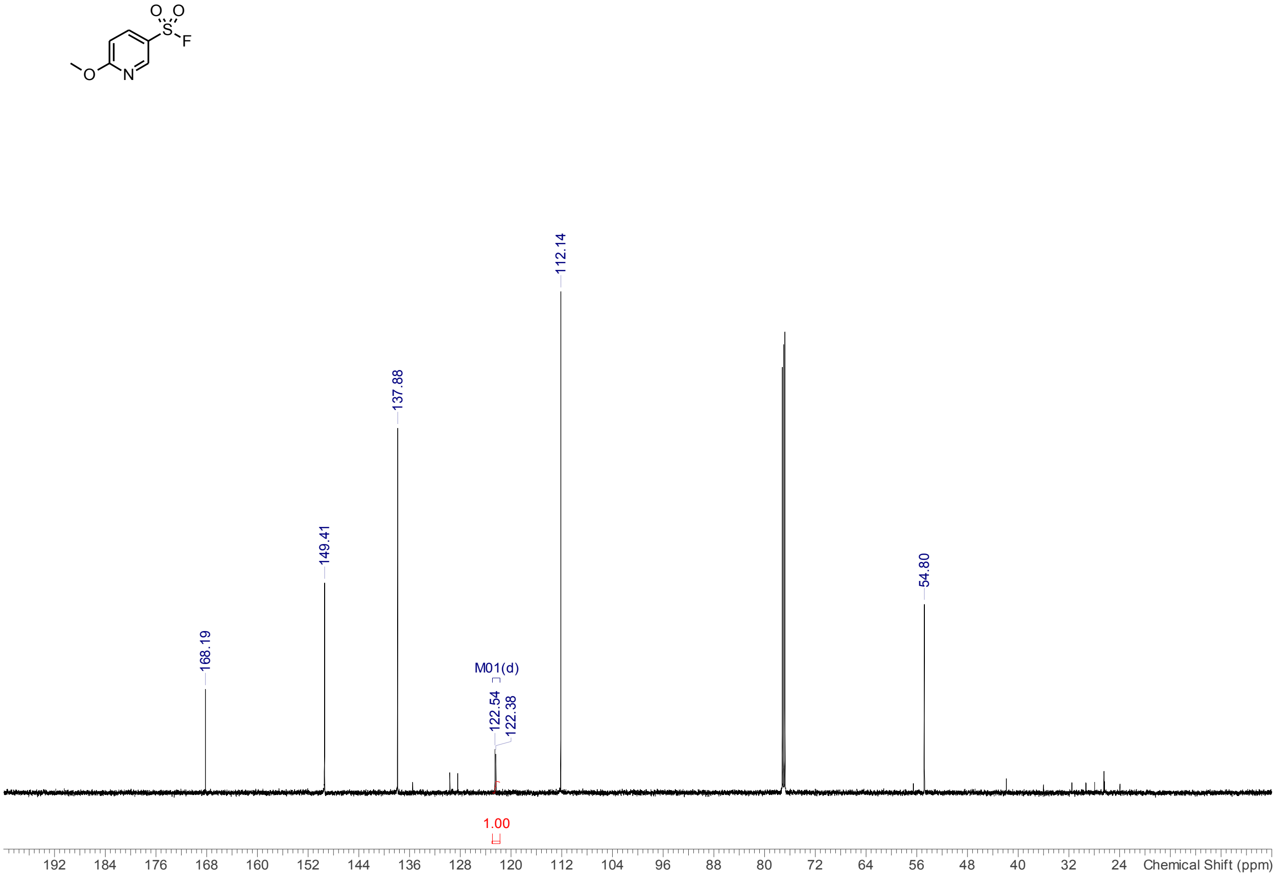
O O
S
F
O
N
168.19
149.41
137.88
M01(d)
122.54
122.38
112.14
54.80
1.00
192 184 176 168 160 152 144 136 128 120 112 104 96 88 80 72 64 56 48 40 32 24
Chemical Shift (ppm)

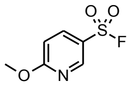

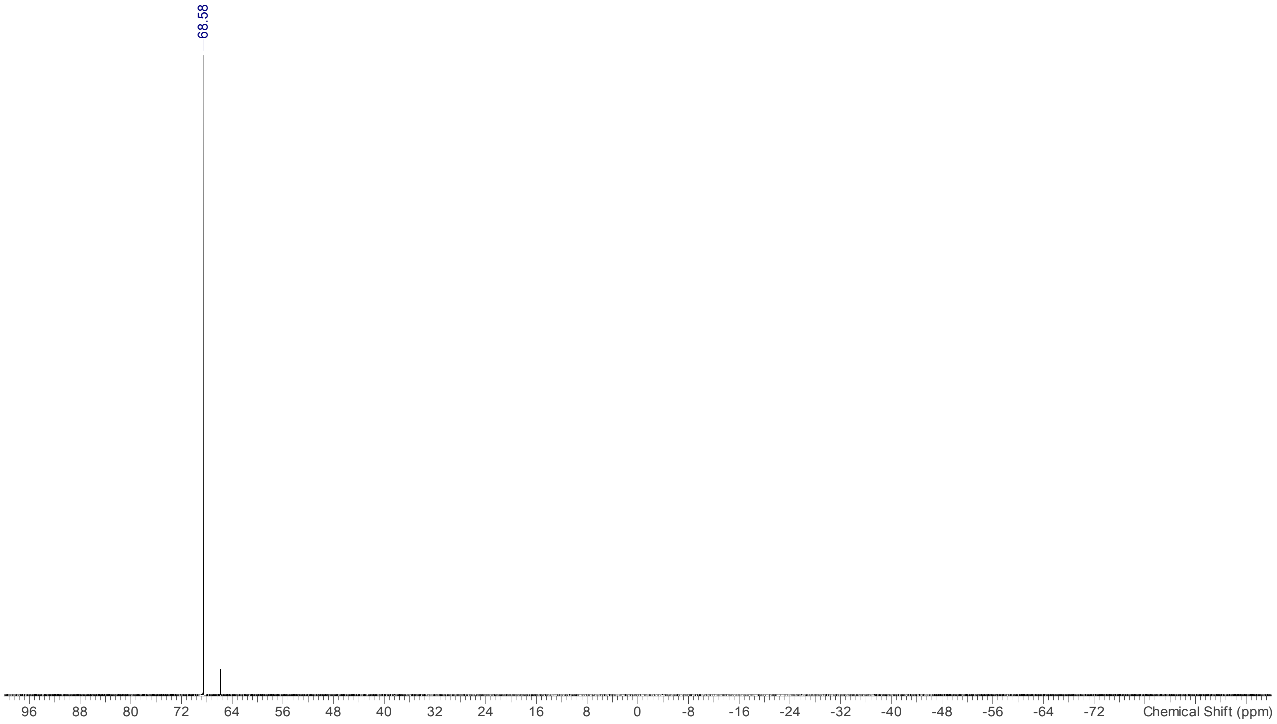
68.58
96
88
80
72
64
56
48
40
32
24
16
8
0
-8
-16
-24
-32
-40
-48
-56
-64
-72
Chemical Shift (ppm)

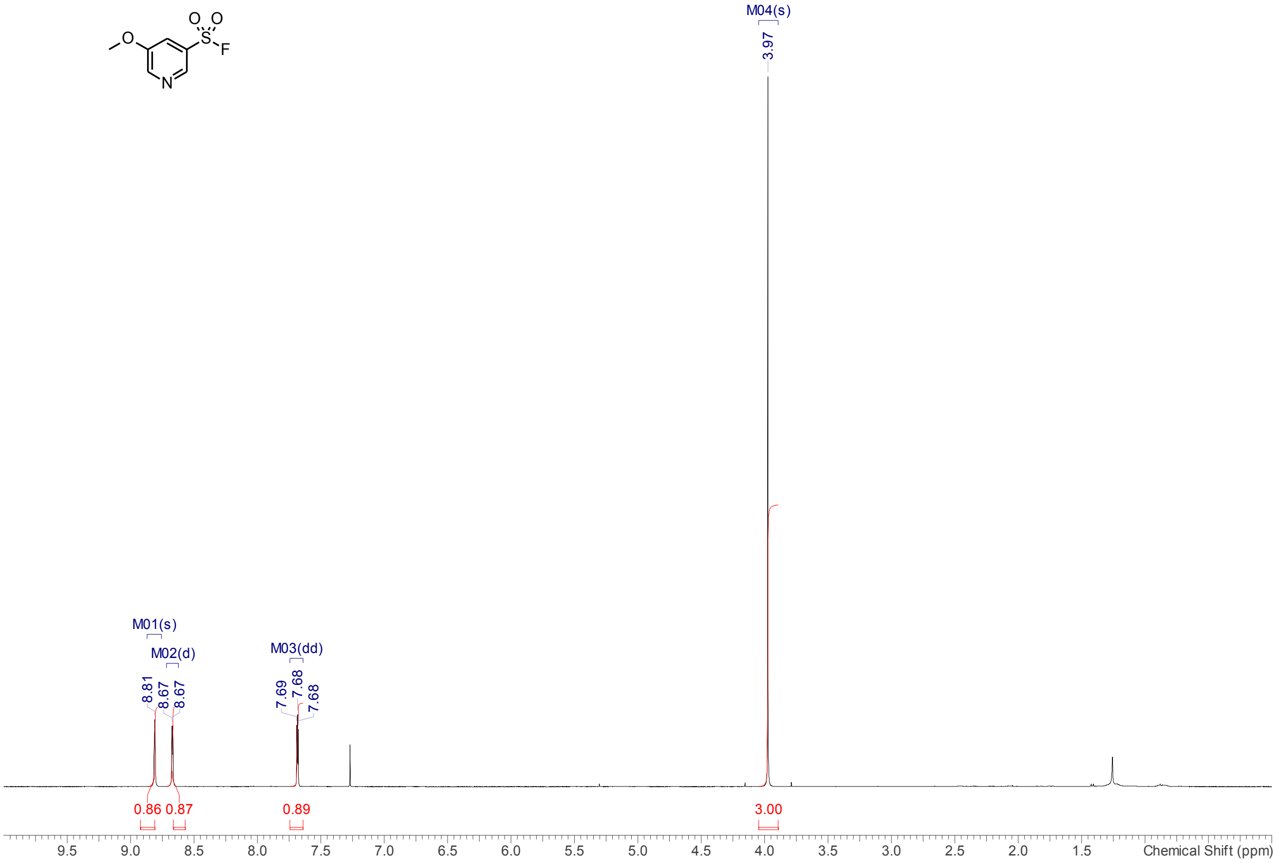
M04(s)
3.97
M01(s)
M02(d)
M03(dd)
8.81
8.67
8.67
7.69
7.68
7.68
0.86
0.87
0.89
3.00
Chemical Shift (ppm)

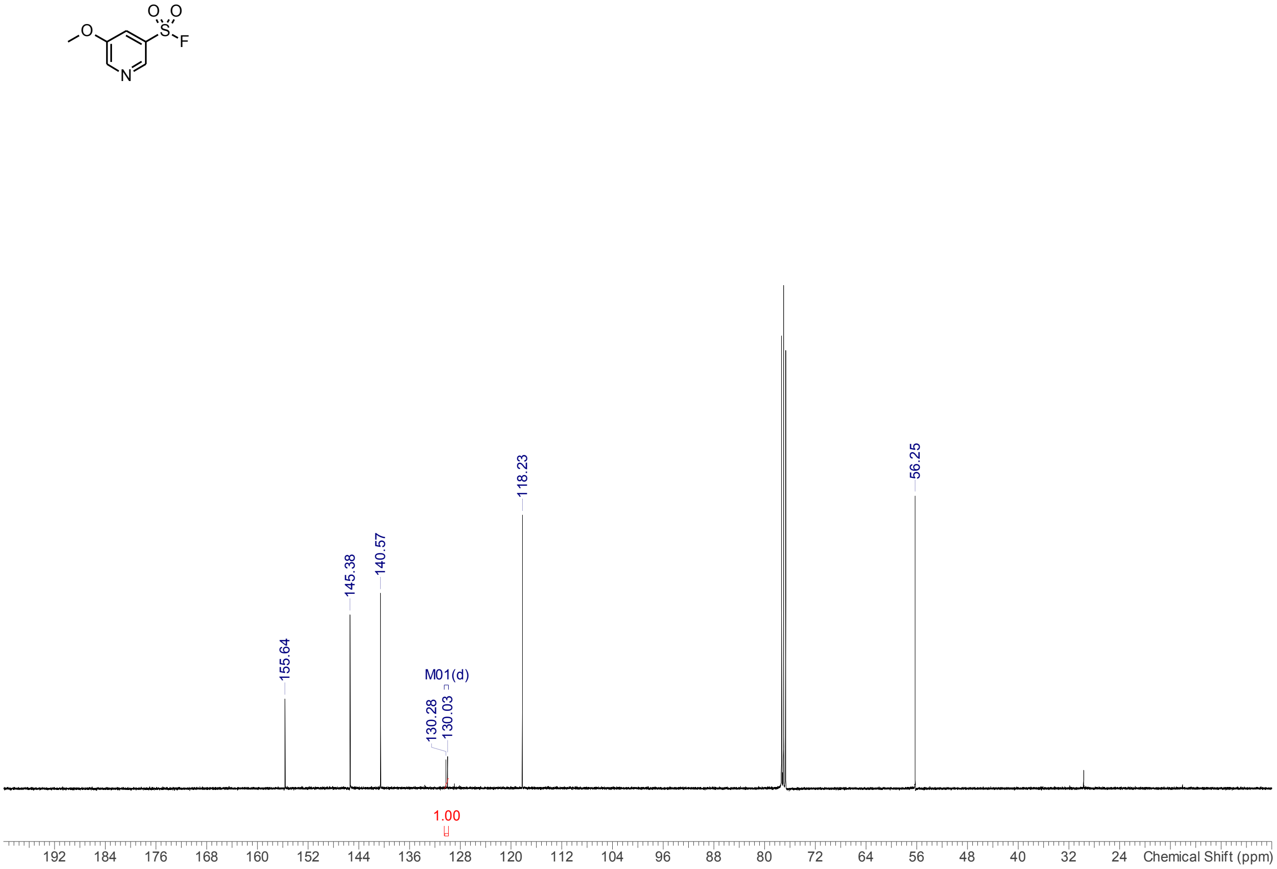
155.64
145.38
140.57
M01(d)
130.28
130.03
118.23
56.25
1.00
Chemical Shift (ppm)

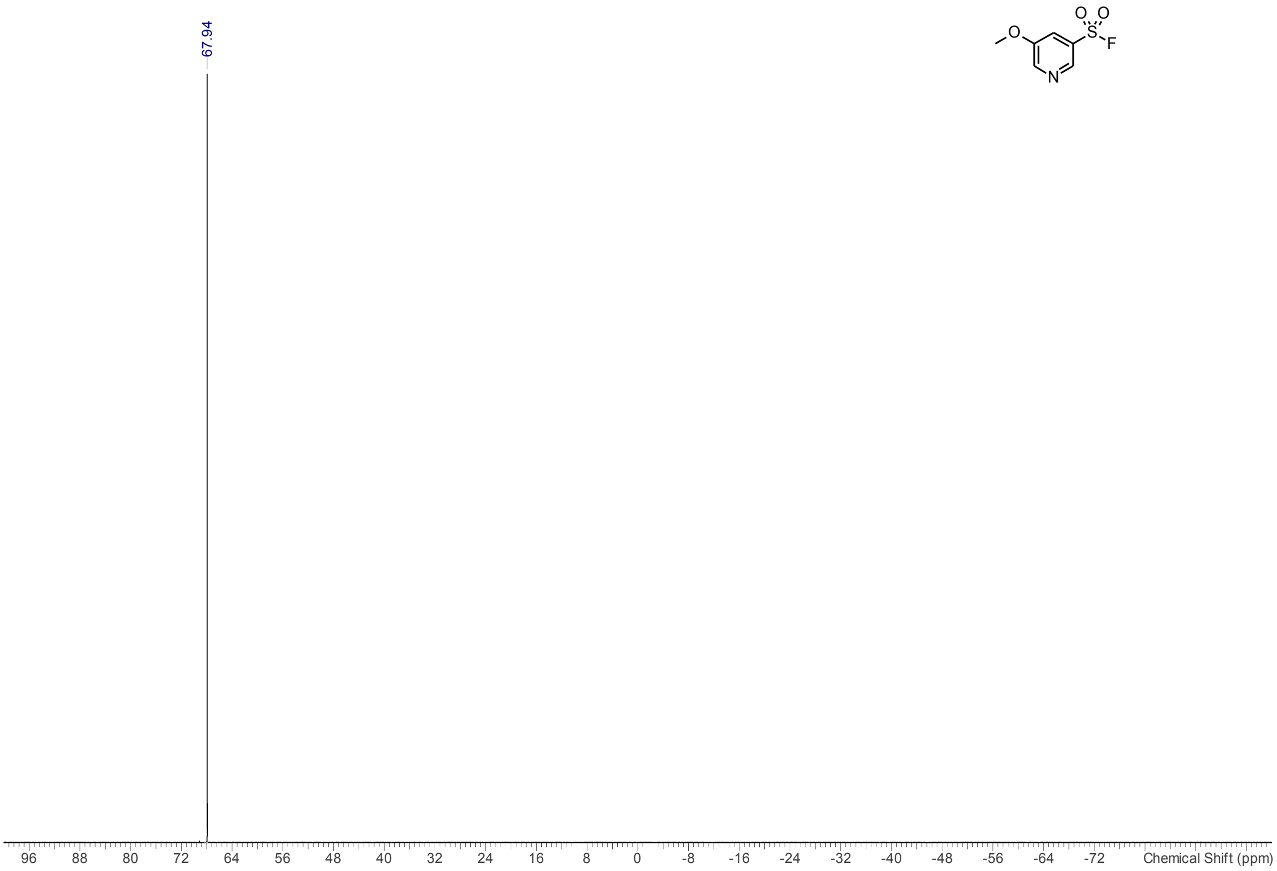
67.94
96 88 80 72 64 56 48 40 32 24 16 8 0 -8 -16 -24 -32 -40 -48 -56 -64 -72 Chemical Shift (ppm)

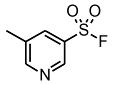

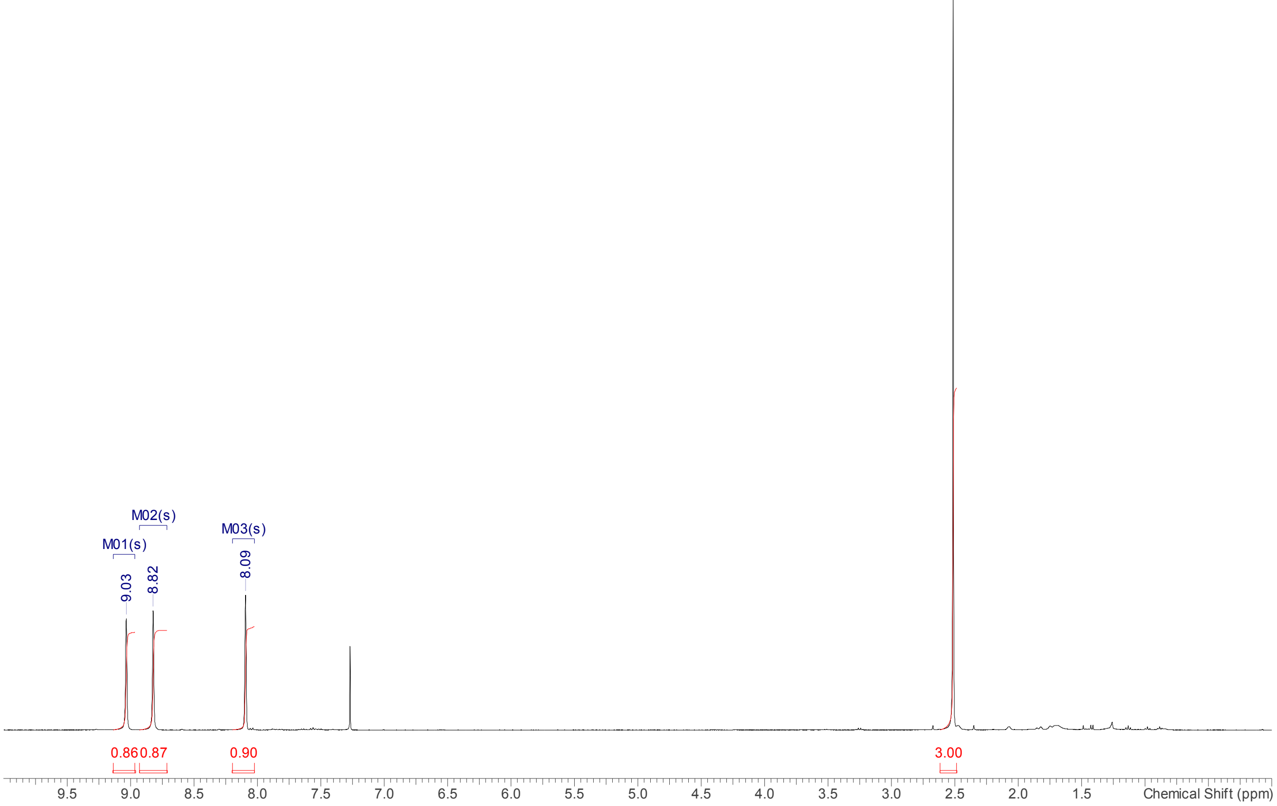
M01(s)
M02(s)
M03(s)
M04(s)
9.03
8.82
8.09
2.51
0.86
0.87
0.90
3.00
Chemical Shift (ppm)

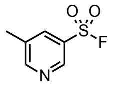

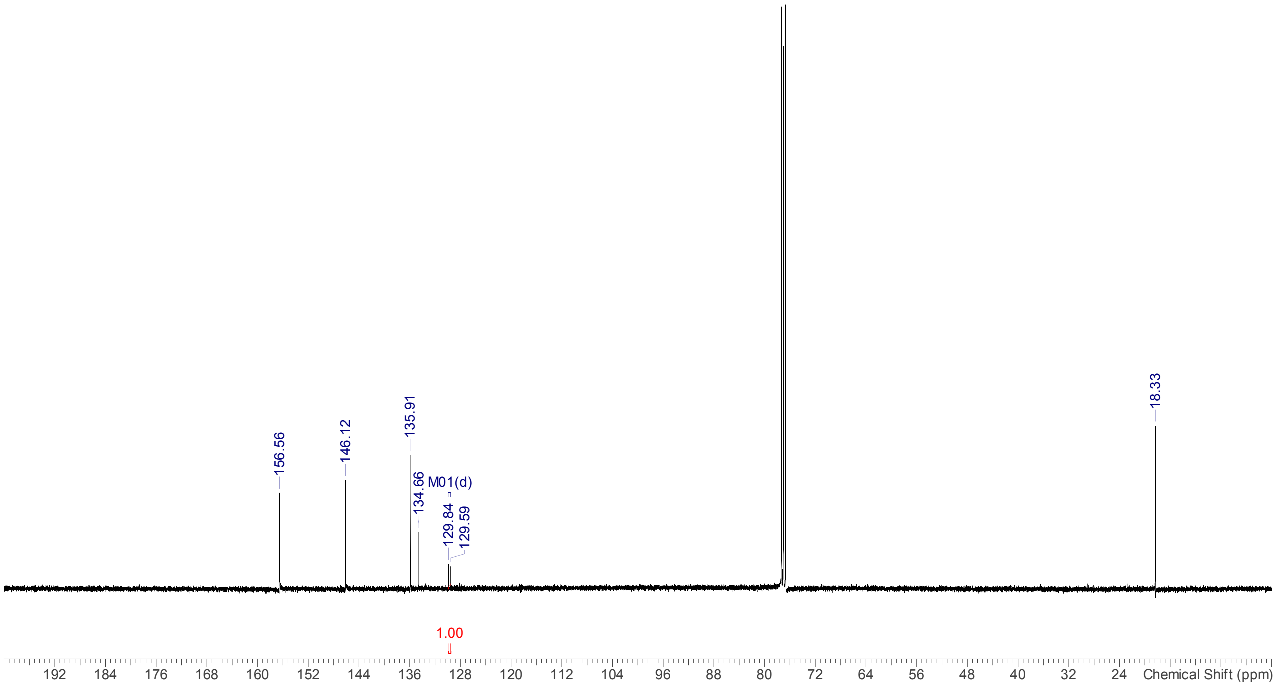
156.56
146.12
135.91
134.66
M01(d)
129.84
129.59
18.33
1.00
Chemical Shift (ppm)

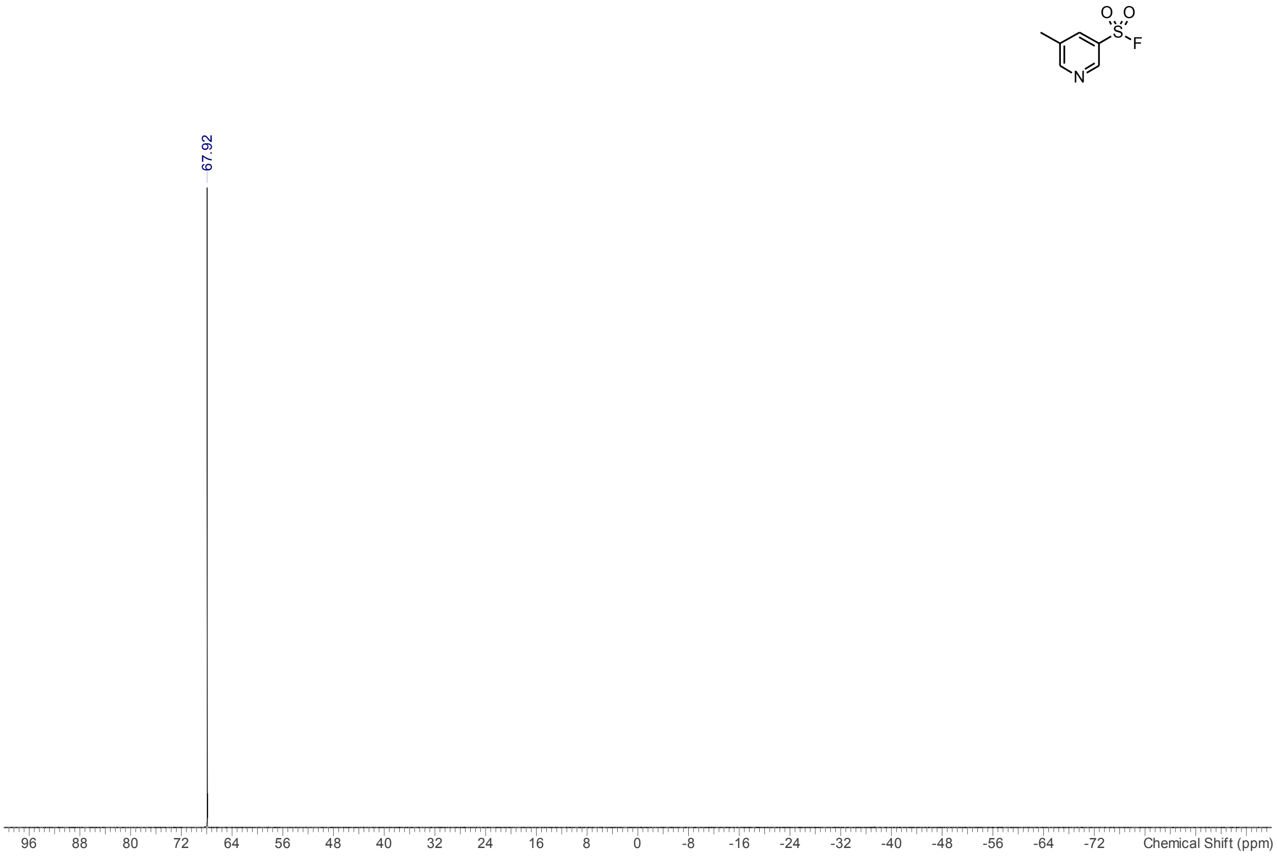

67.92
96 88 80 72 64 56 48 40 32 24 16 8 0 -8 -16 -24 -32 -40 -48 -56 -64 -72 Chemical Shift (ppm)

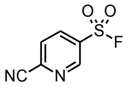

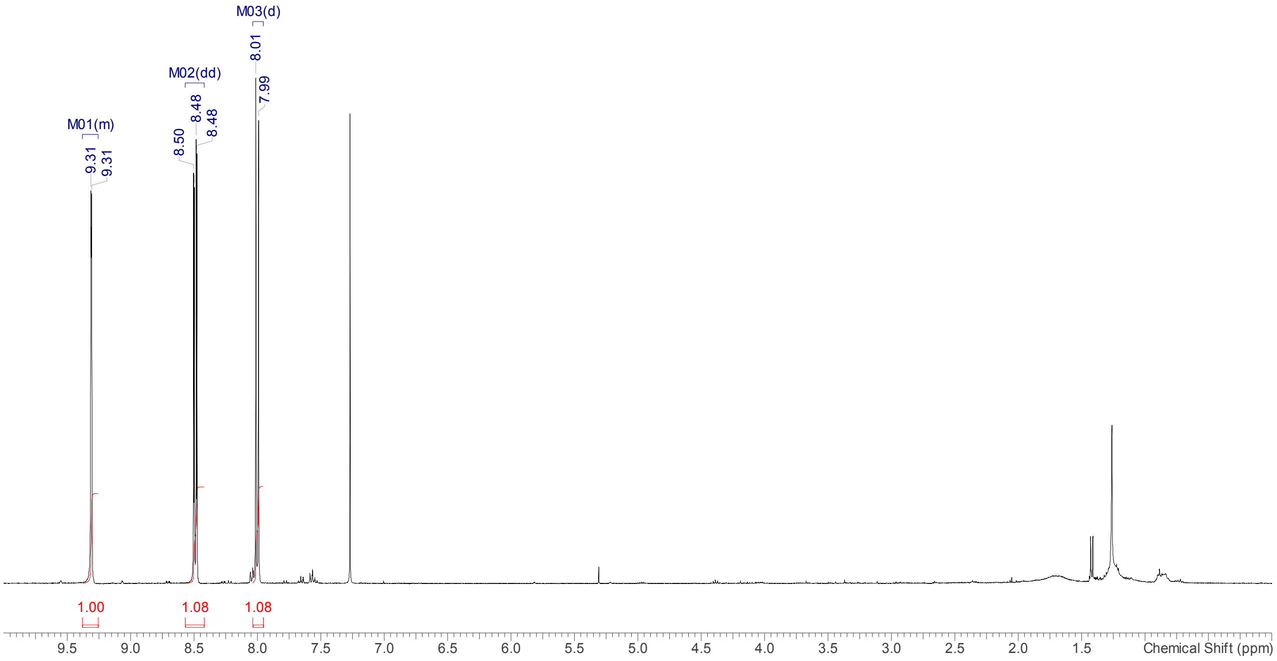
M03(d)
8.01
7.99
M02(dd)
8.48
8.48
8.50
M01(m)
9.31
9.31
1.00
1.08
1.08
9.5 9.0 8.5 8.0 7.5 7.0 6.5 6.0 5.5 5.0 4.5 4.0 3.5 3.0 2.5 2.0 1.5
Chemical Shift (ppm)

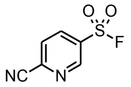

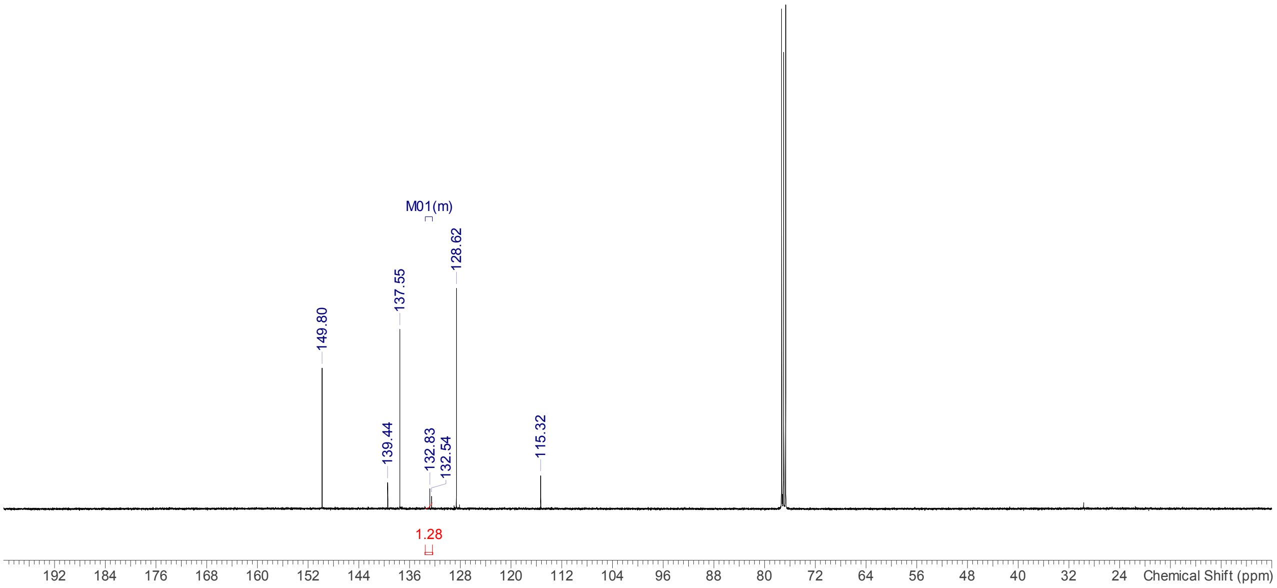
M01(m)
149.80
137.55
128.62
139.44
132.83
132.54
115.32
1.28
192 184 176 168 160 152 144 136 128 120 112 104 96 88 80 72 64 56 48 40 32 24
Chemical Shift (ppm)

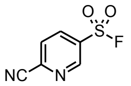

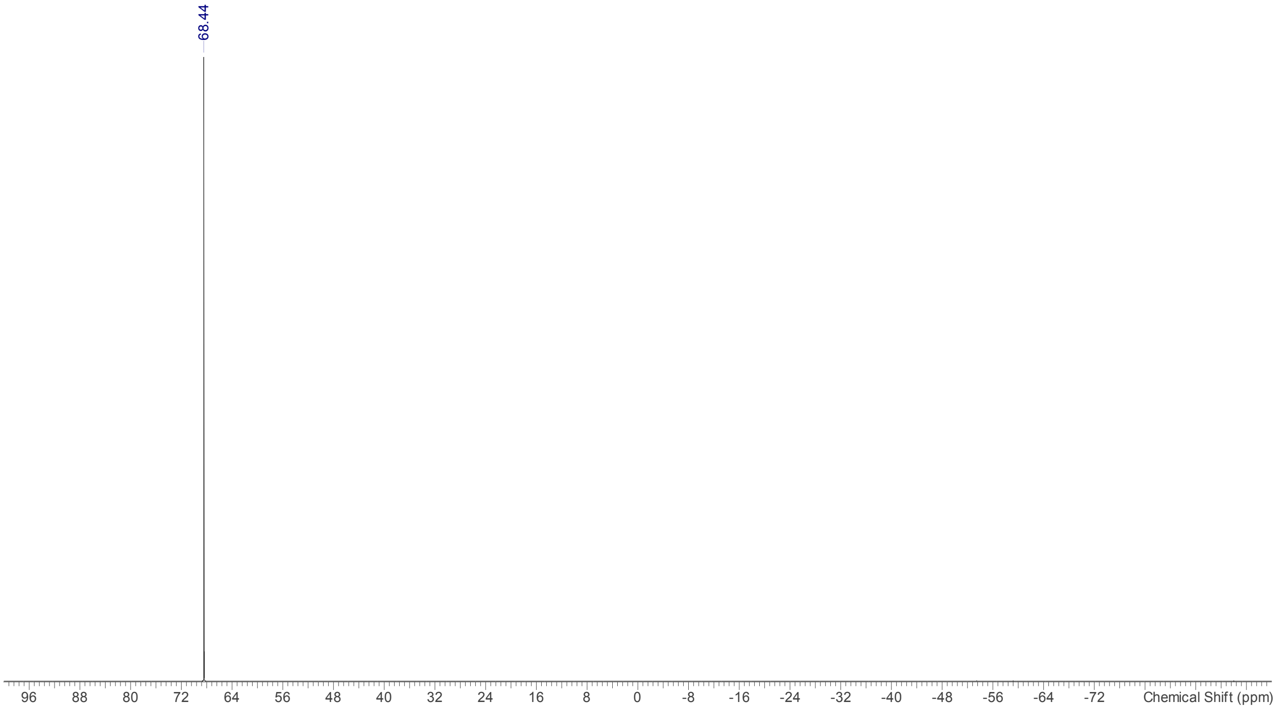

68.44
96 88 80 72 64 56 48 40 32 24 16 8 0 -8 -16 -24 -32 -40 -48 -56 -64 -72 Chemical Shift (ppm)

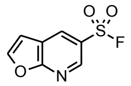
O
O
S
F
O
N

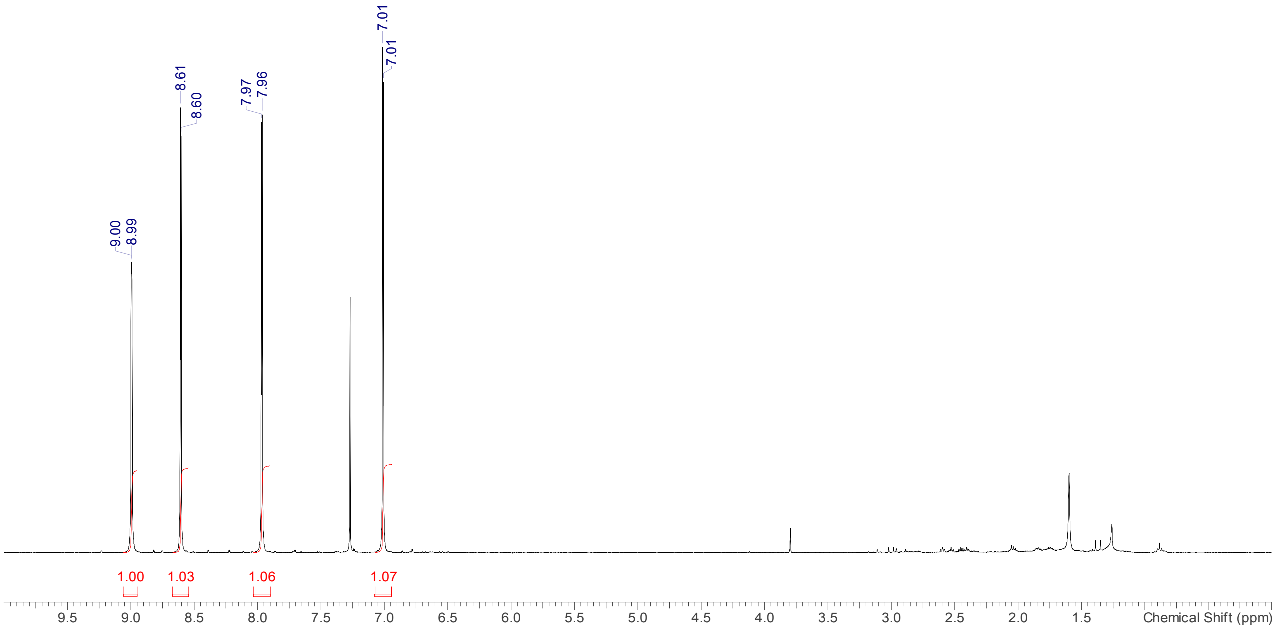
9.00
8.99
8.61
8.60
7.97
7.96
7.01
7.01
1.00
1.03
1.06
1.07
9.5
9.0
8.5
8.0
7.5
7.0
6.5
6.0
5.5
5.0
4.5
4.0
3.5
3.0
2.5
2.0
1.5
Chemical Shift (ppm)

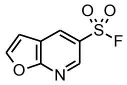
O
O
S
F
O
N

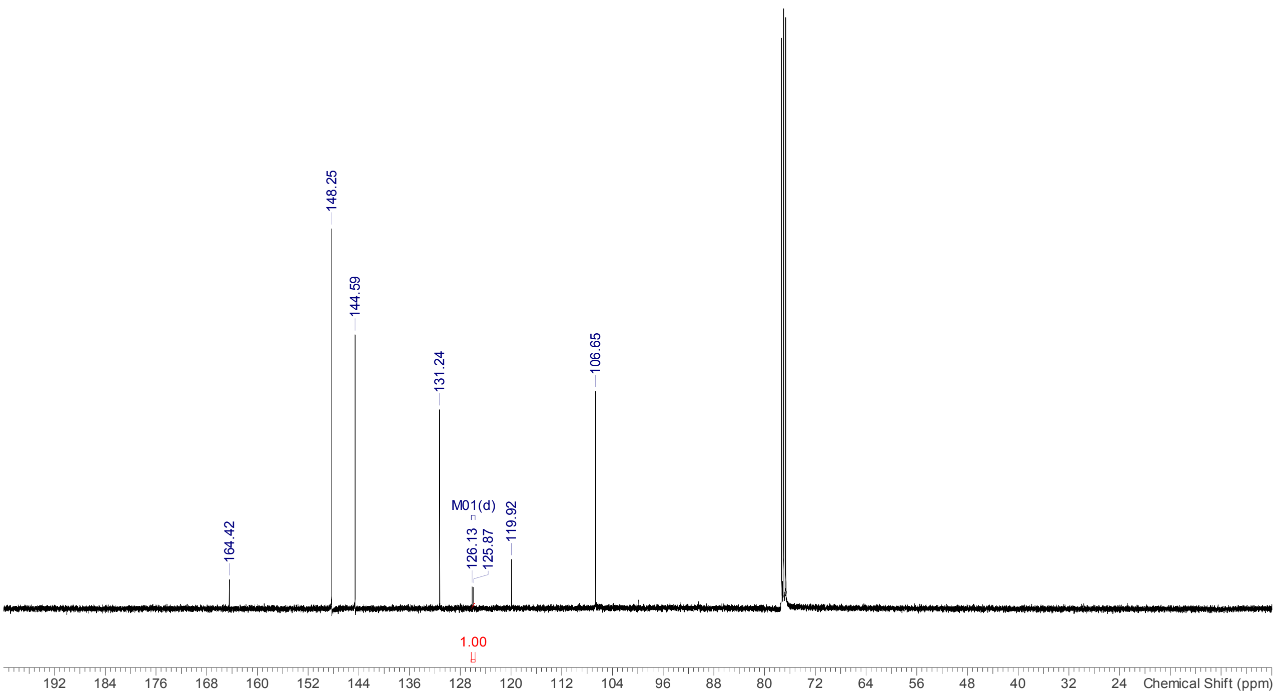
164.42
148.25
144.59
131.24
M01(d)
126.13
125.87
119.92
106.65
1.00
192
184
176
168
160
152
144
136
128
120
112
104
96
88
80
72
64
56
48
40
32
24
Chemical Shift (ppm)

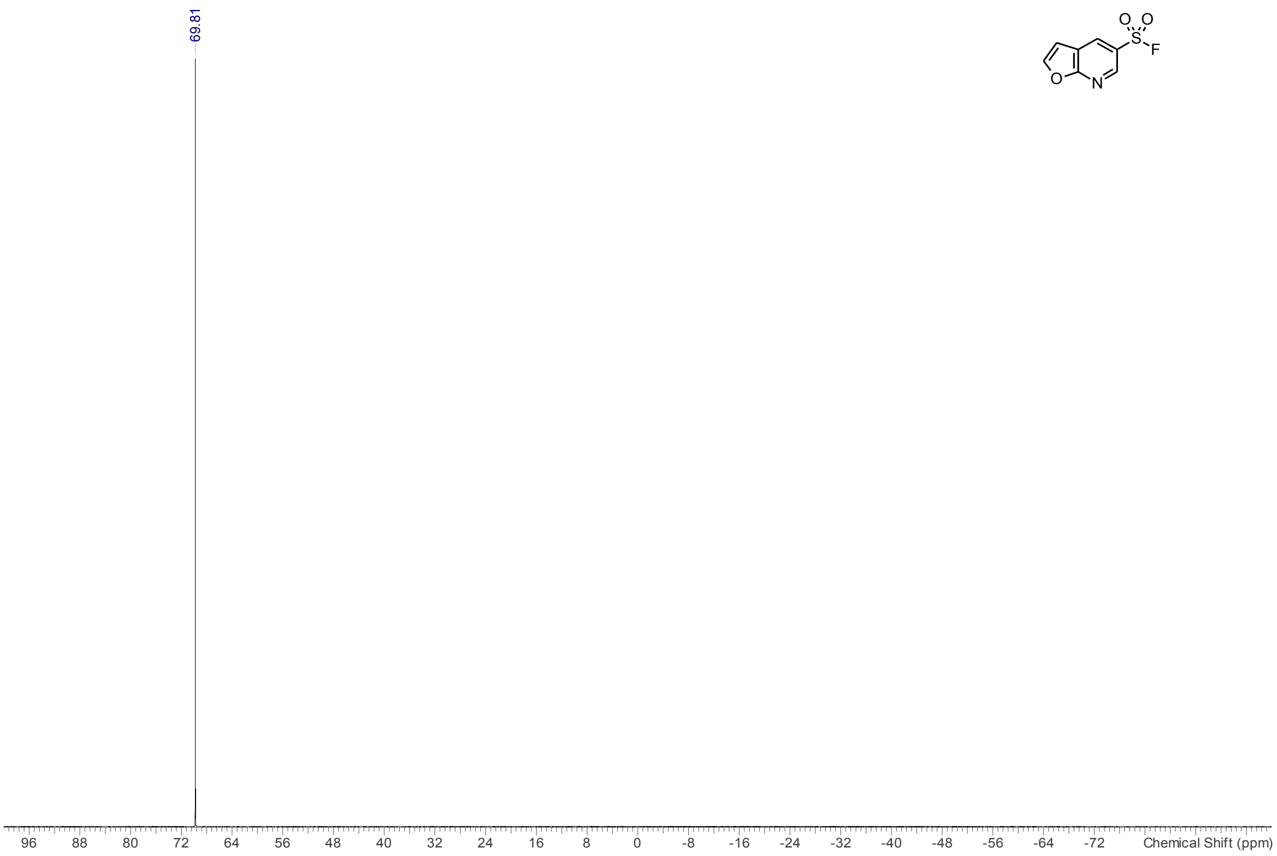
69.81
O
S
O
F
O
N
96 88 80 72 64 56 48 40 32 24 16 8 0 -8 -16 -24 -32 -40 -48 -56 -64 -72
Chemical Shift (ppm)

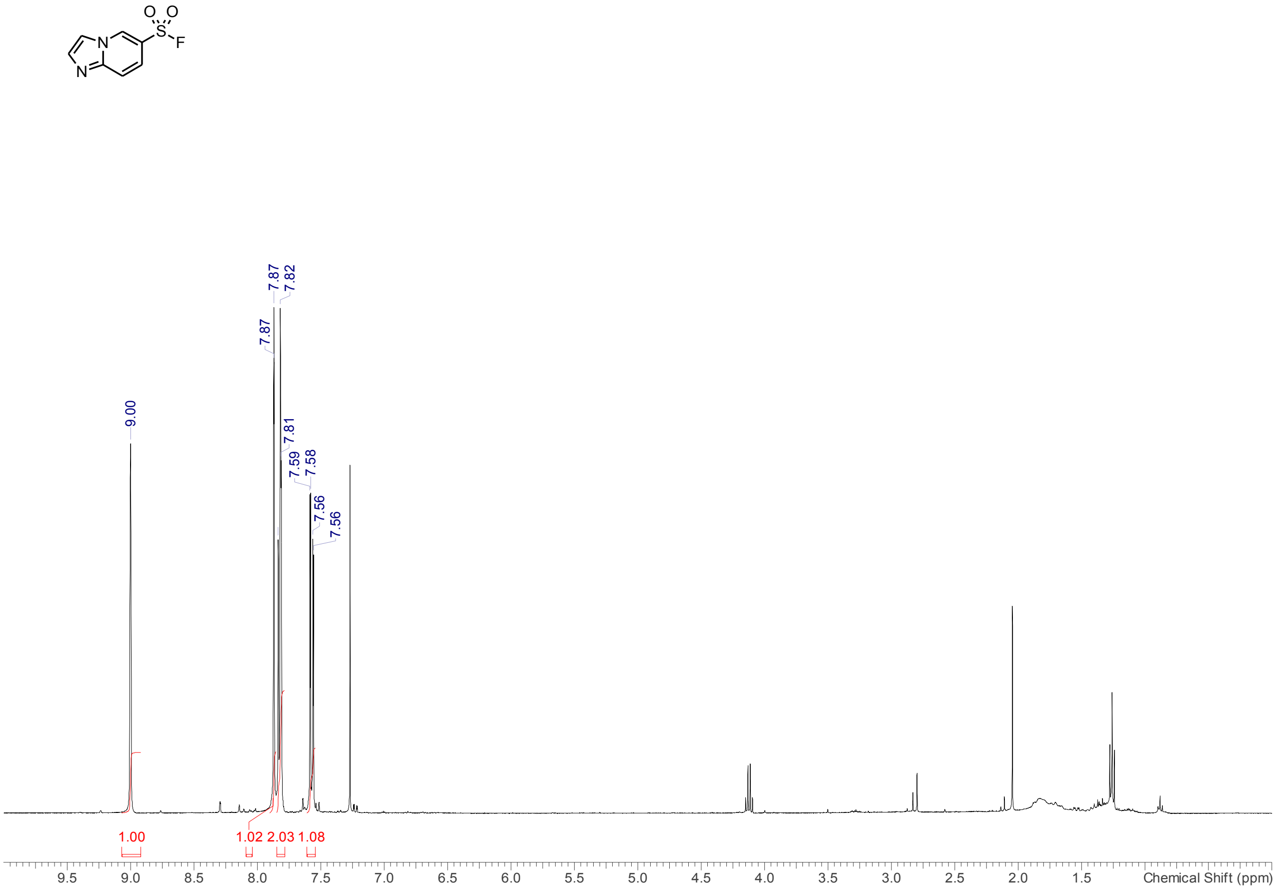

O O
S
F
N
N
9.00
7.87
7.87
7.82
7.81
7.59
7.58
7.56
7.56
1.00
1.02 2.03 1.08
9.5 9.0 8.5 8.0 7.5 7.0 6.5 6.0 5.5 5.0 4.5 4.0 3.5 3.0 2.5 2.0 1.5
Chemical Shift (ppm)

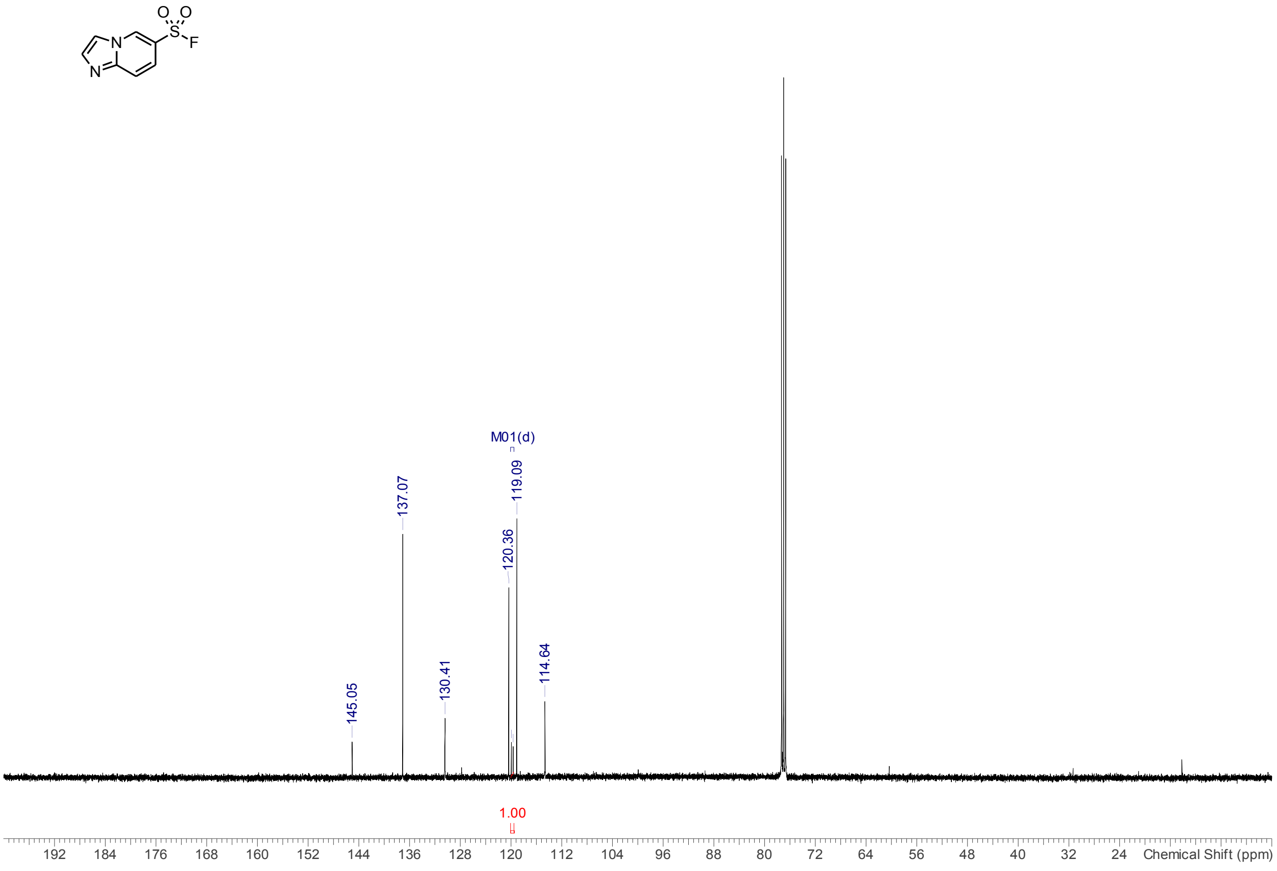

O O
S
F
N
N
M01(d)
145.05
137.07
130.41
120.36
119.09
114.64
1.00
192 184 176 168 160 152 144 136 128 120 112 104 96 88 80 72 64 56 48 40 32 24
Chemical Shift (ppm)

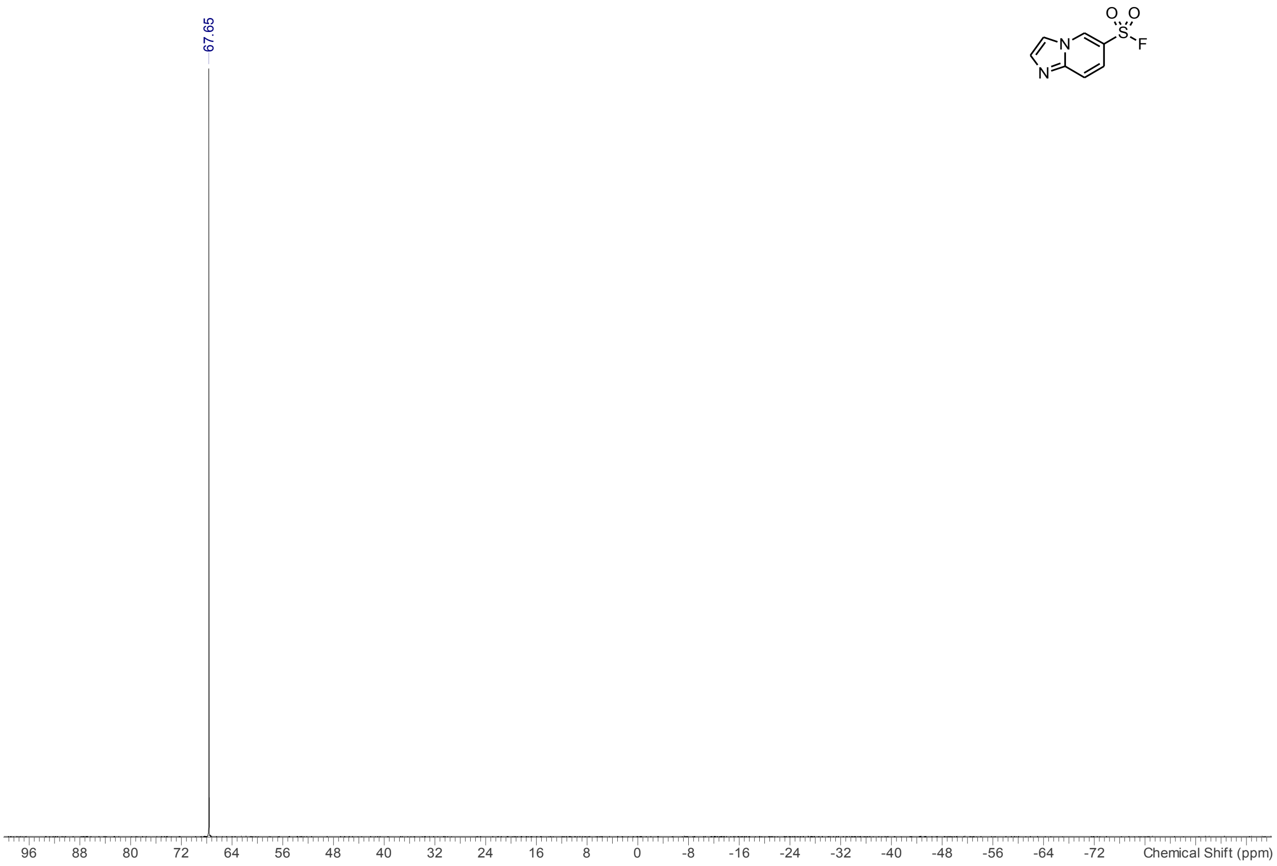
67.65
96 88 80 72 64 56 48 40 32 24 16 8 0 -8 -16 -24 -32 -40 -48 -56 -64 -72
Chemical Shift (ppm)
O O
S
F
N
N

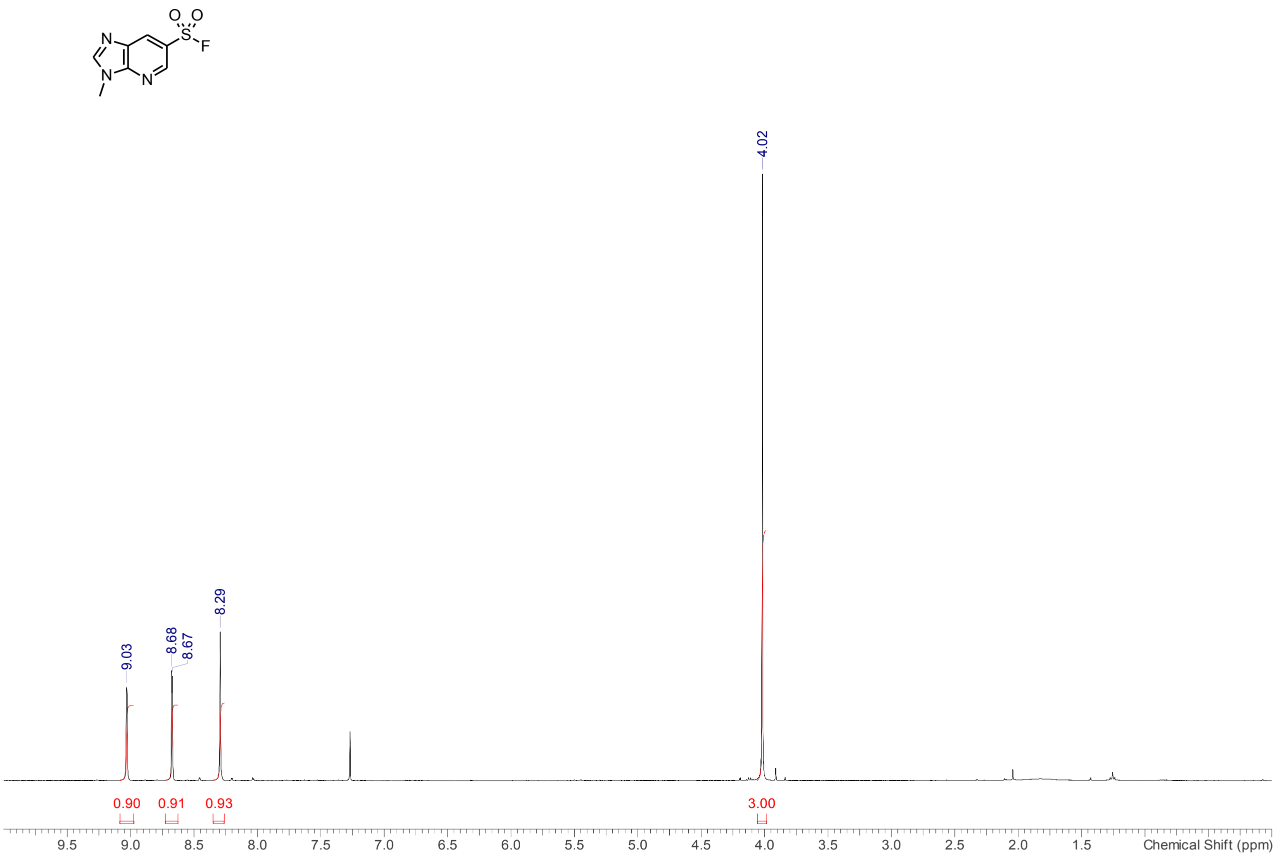
9.03
8.68
8.67
8.29
4.02
0.90
0.91
0.93
3.00
Chemical Shift (ppm)

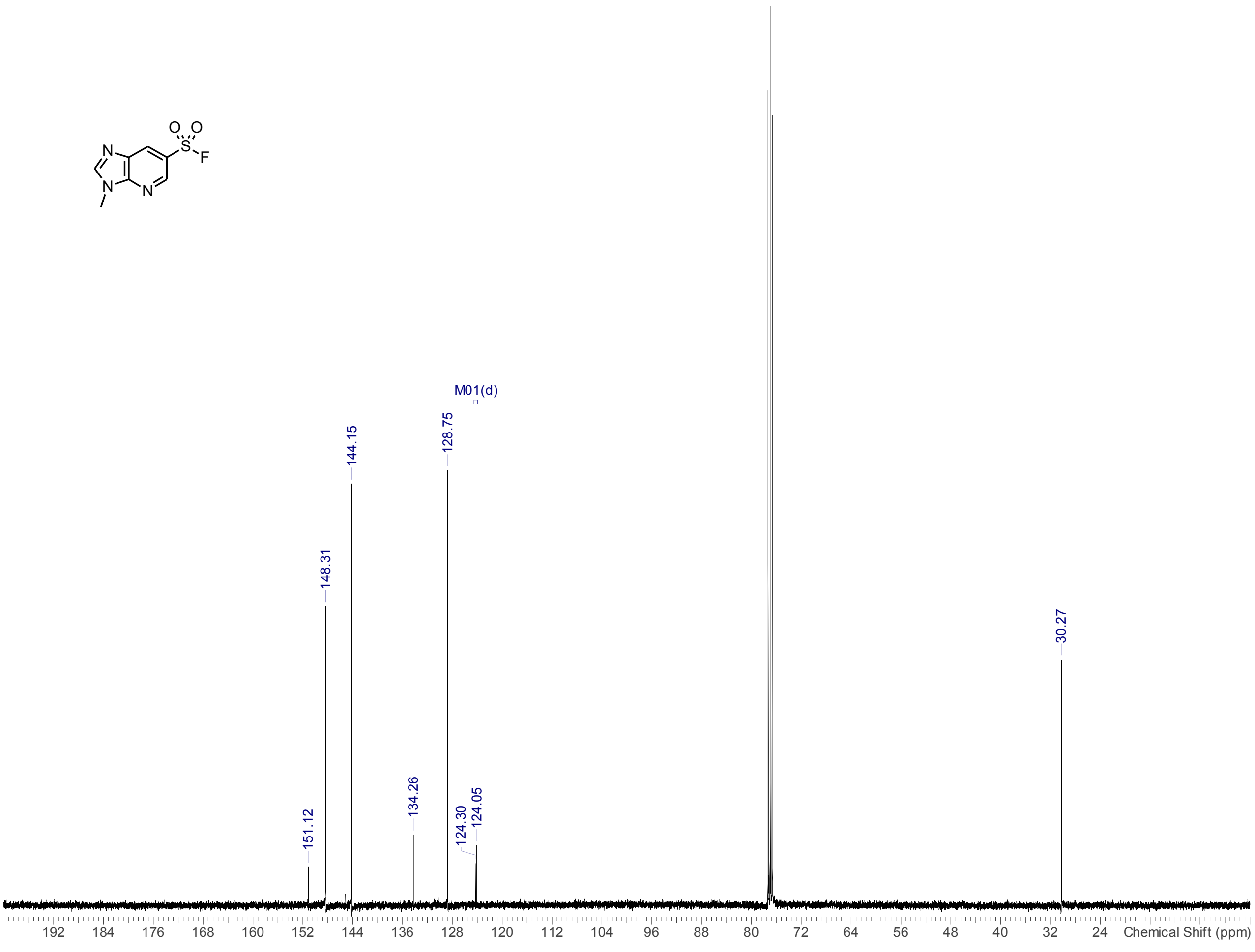
M01(d)
151.12
148.31
144.15
134.26
128.75
124.30
124.05
30.27
Chemical Shift (ppm)

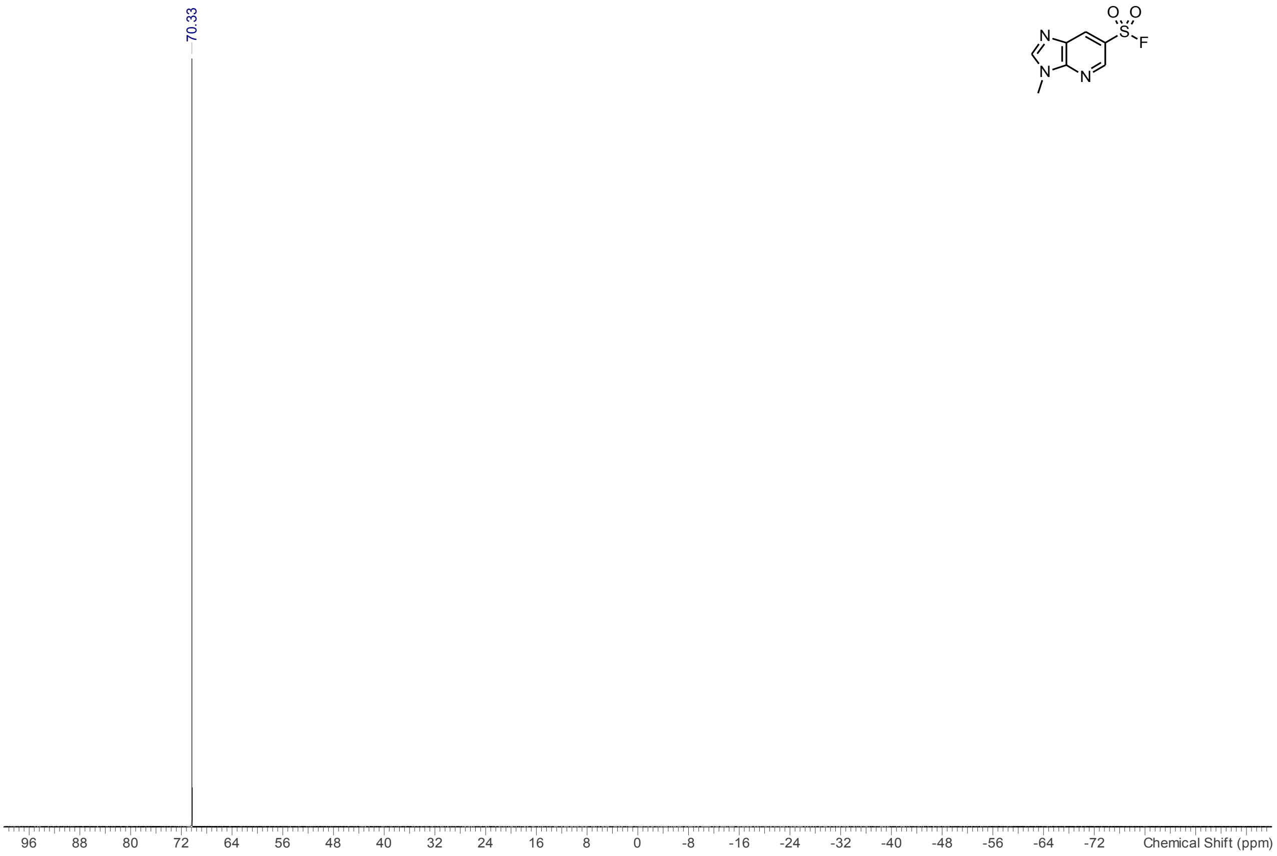

70.33
96
88
80
72
64
56
48
40
32
24
16
8
0
-8
-16
-24
-32
-40
-48
-56
-64
-72
Chemical Shift (ppm)

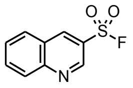
O O
S
F
N

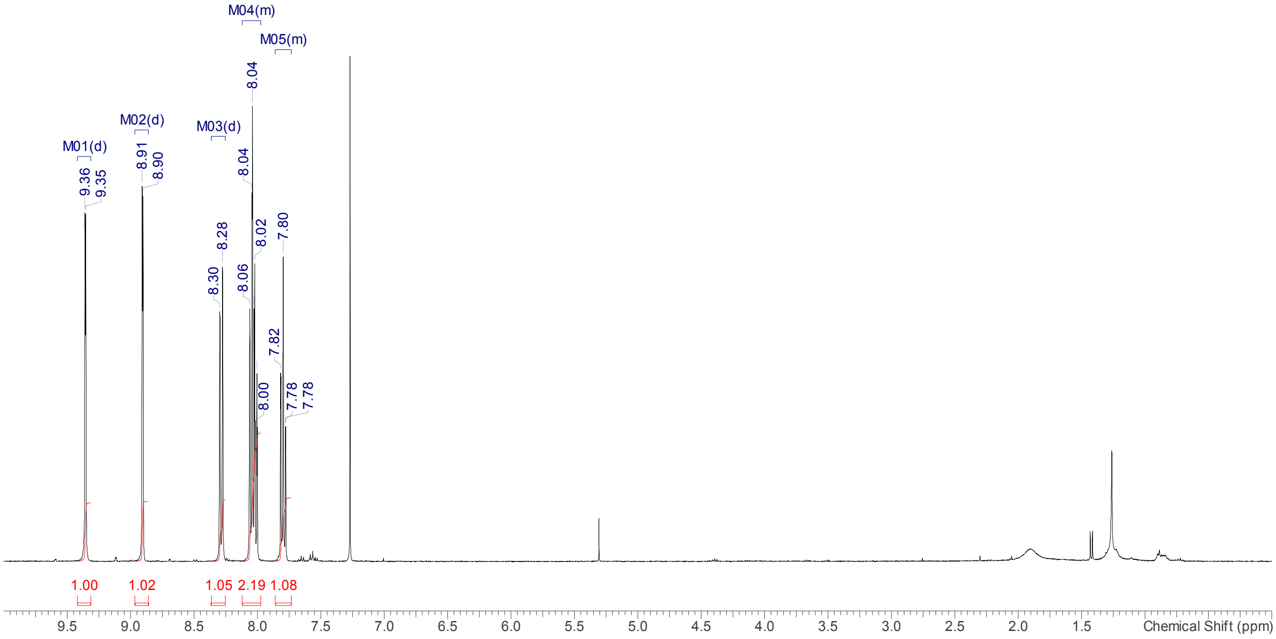
M01(d)
9.36
9.35
M02(d)
8.91
8.90
M03(d)
8.30
8.28
M04(m)
8.04
8.04
8.06
8.02
8.00
M05(m)
7.80
7.82
7.78
7.78
1.00 1.02 1.05 2.19 1.08
9.5 9.0 8.5 8.0 7.5 7.0 6.5 6.0 5.5 5.0 4.5 4.0 3.5 3.0 2.5 2.0 1.5
Chemical Shift (ppm)

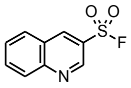
O O
S
F
N

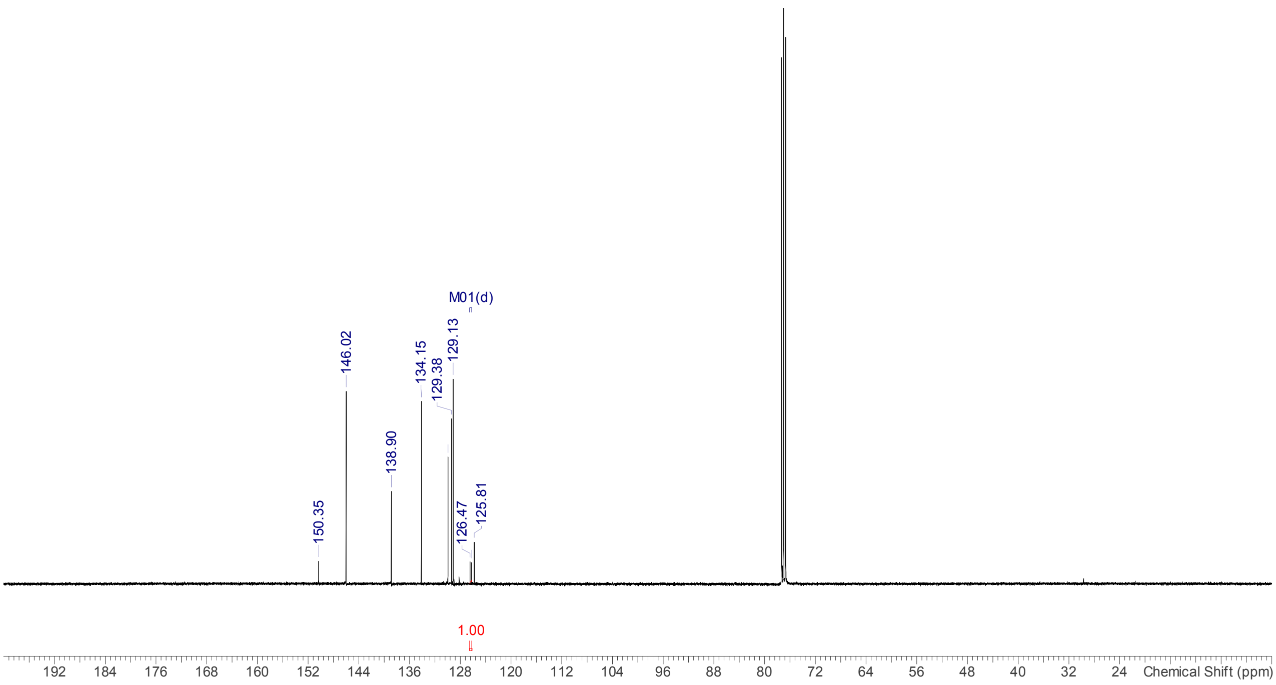
150.35
146.02
138.90
134.15
129.38
129.13
M01(d)
126.47
125.81
1.00
192 184 176 168 160 152 144 136 128 120 112 104 96 88 80 72 64 56 48 40 32 24
Chemical Shift (ppm)

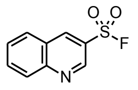

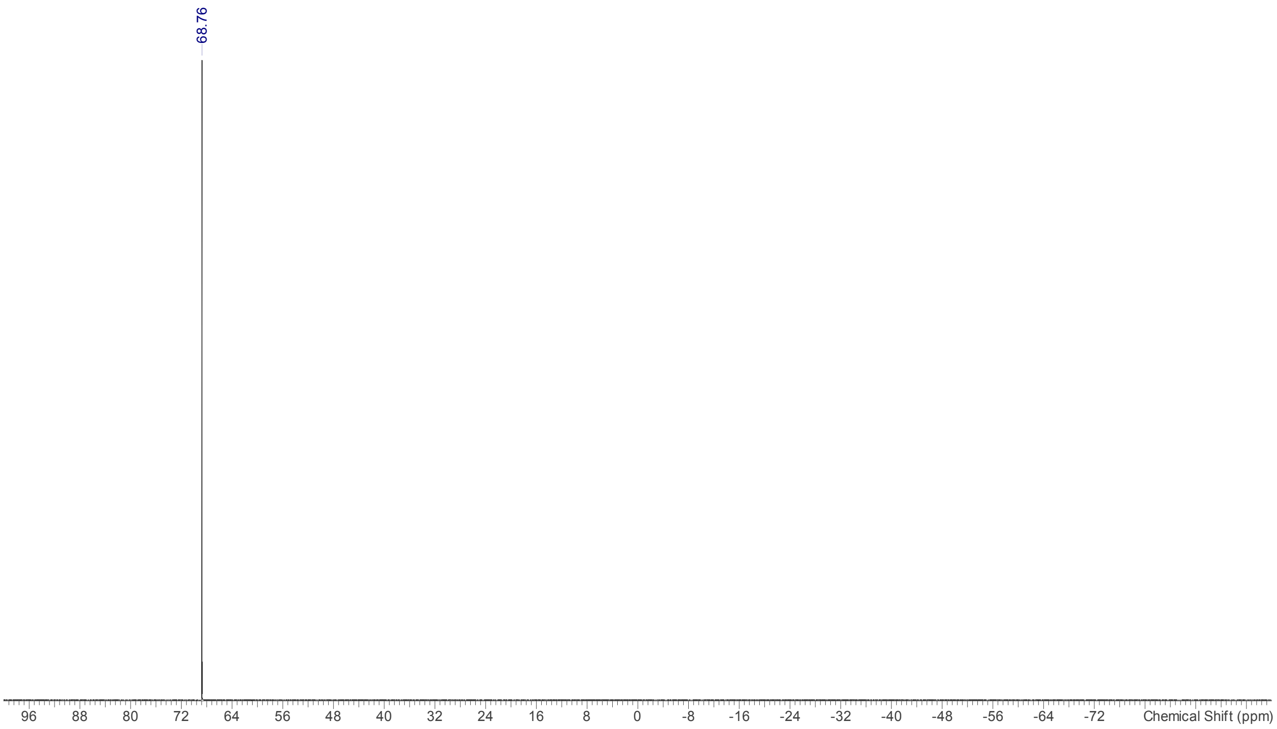

68.76
96
88
80
72
64
56
48
40
32
24
16
8
0
-8
-16
-24
-32
-40
-48
-56
-64
-72
Chemical Shift (ppm)

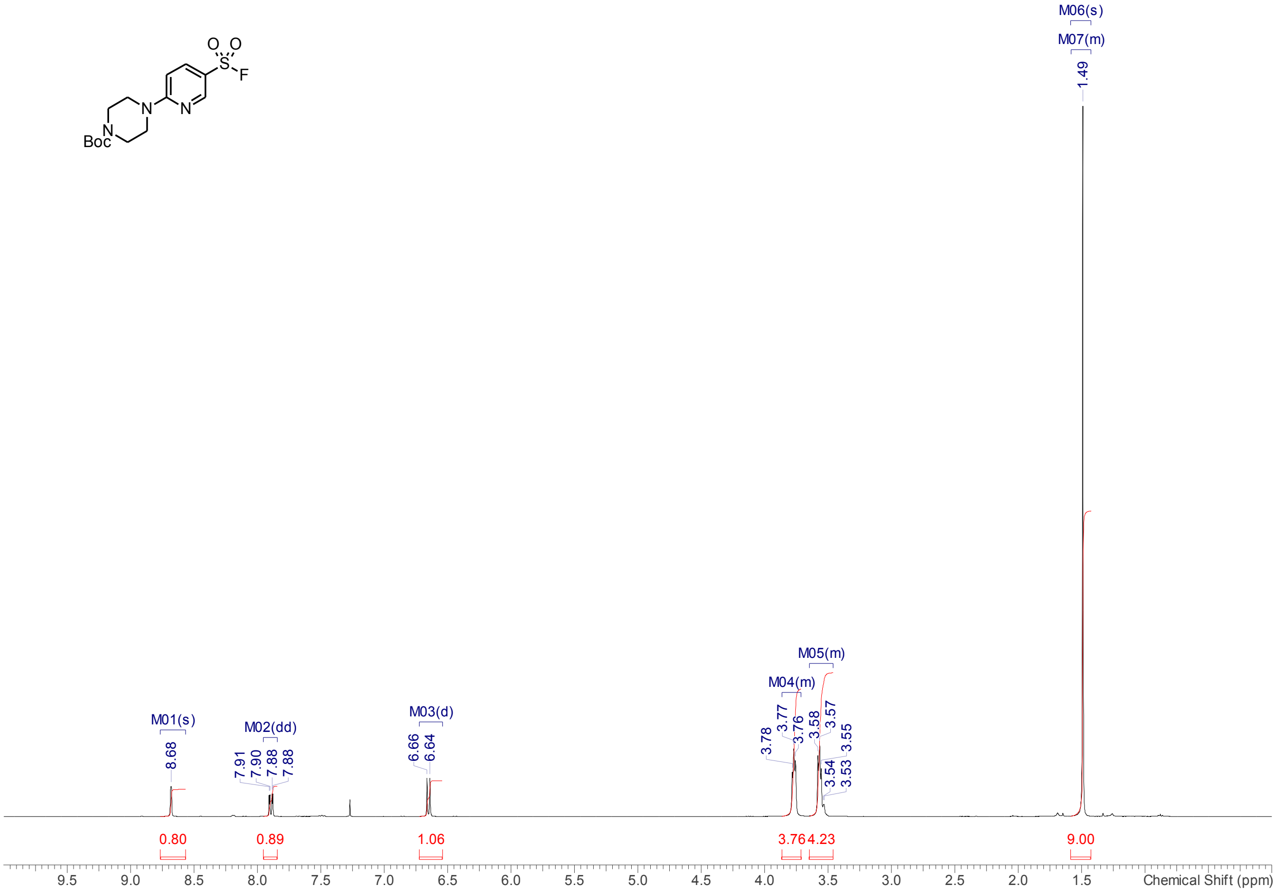
Boc
N
N
N
S
O
O
F
M06(s)
M07(m)
1.49
M01(s)
8.68
M02(dd)
7.91
7.90
7.88
7.88
M03(d)
6.66
6.64
M04(m)
3.78
3.77
3.76
M05(m)
3.58
3.57
3.55
3.54
3.53
0.80
0.89
1.06
3.76
4.23
9.00
9.5 9.0 8.5 8.0 7.5 7.0 6.5 6.0 5.5 5.0 4.5 4.0 3.5 3.0 2.5 2.0 1.5
Chemical Shift (ppm)

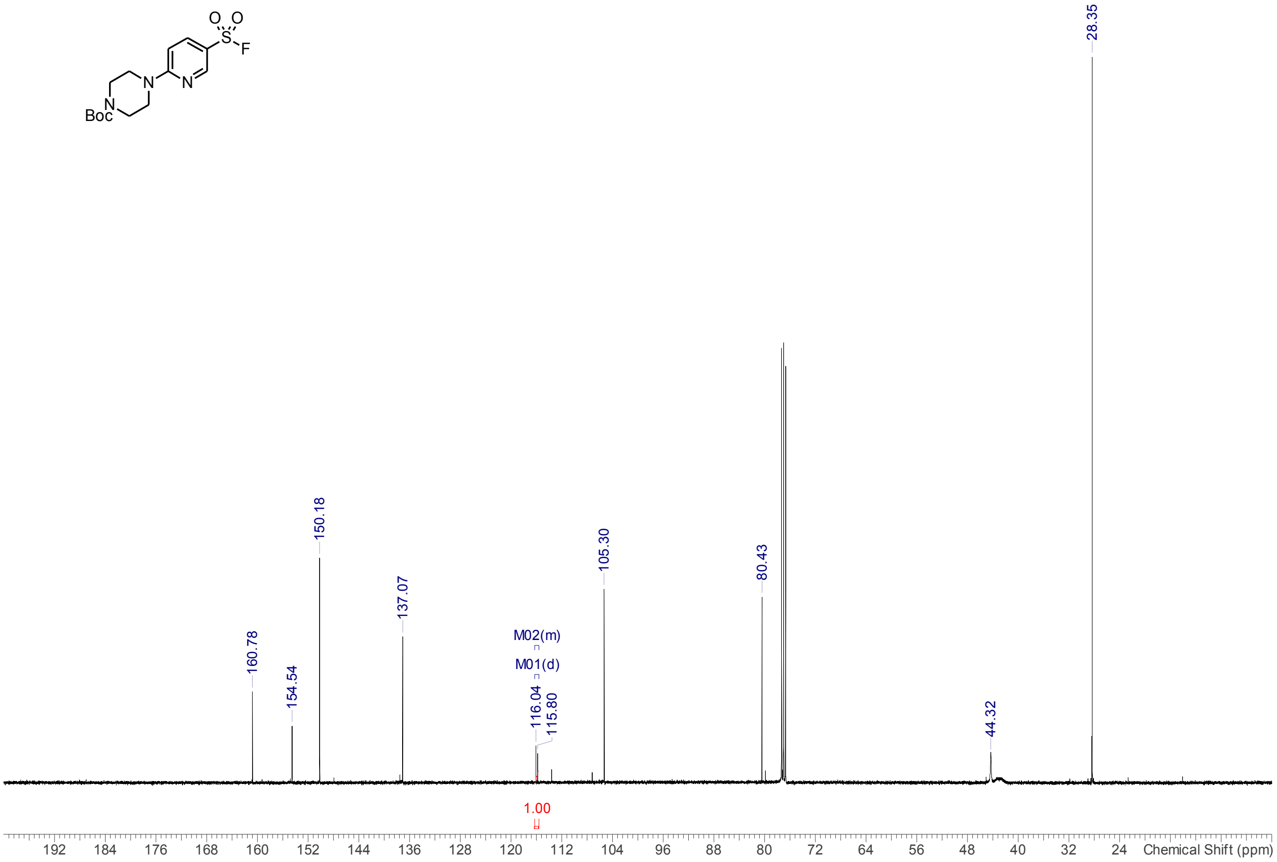
Boc
N
N
N
S
O
O
F
28.35
150.18
105.30
80.43
137.07
160.78
154.54
M02(m)
M01(d)
116.04
115.80
44.32
1.00
192 184 176 168 160 152 144 136 128 120 112 104 96 88 80 72 64 56 48 40 32 24
Chemical Shift (ppm)

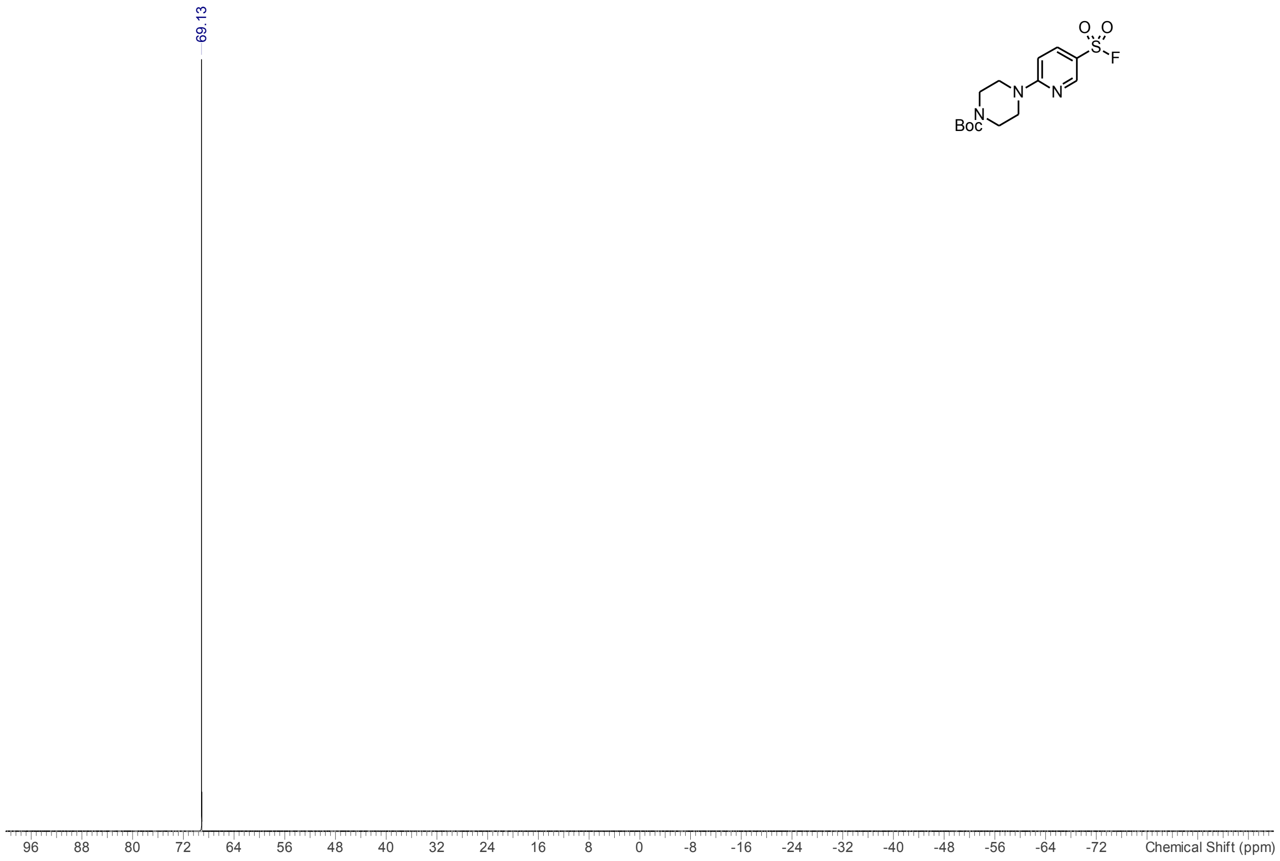
69.13
Boc
N
N
N
O O
S
F
96 88 80 72 64 56 48 40 32 24 16 8 0 -8 -16 -24 -32 -40 -48 -56 -64 -72
Chemical Shift (ppm)

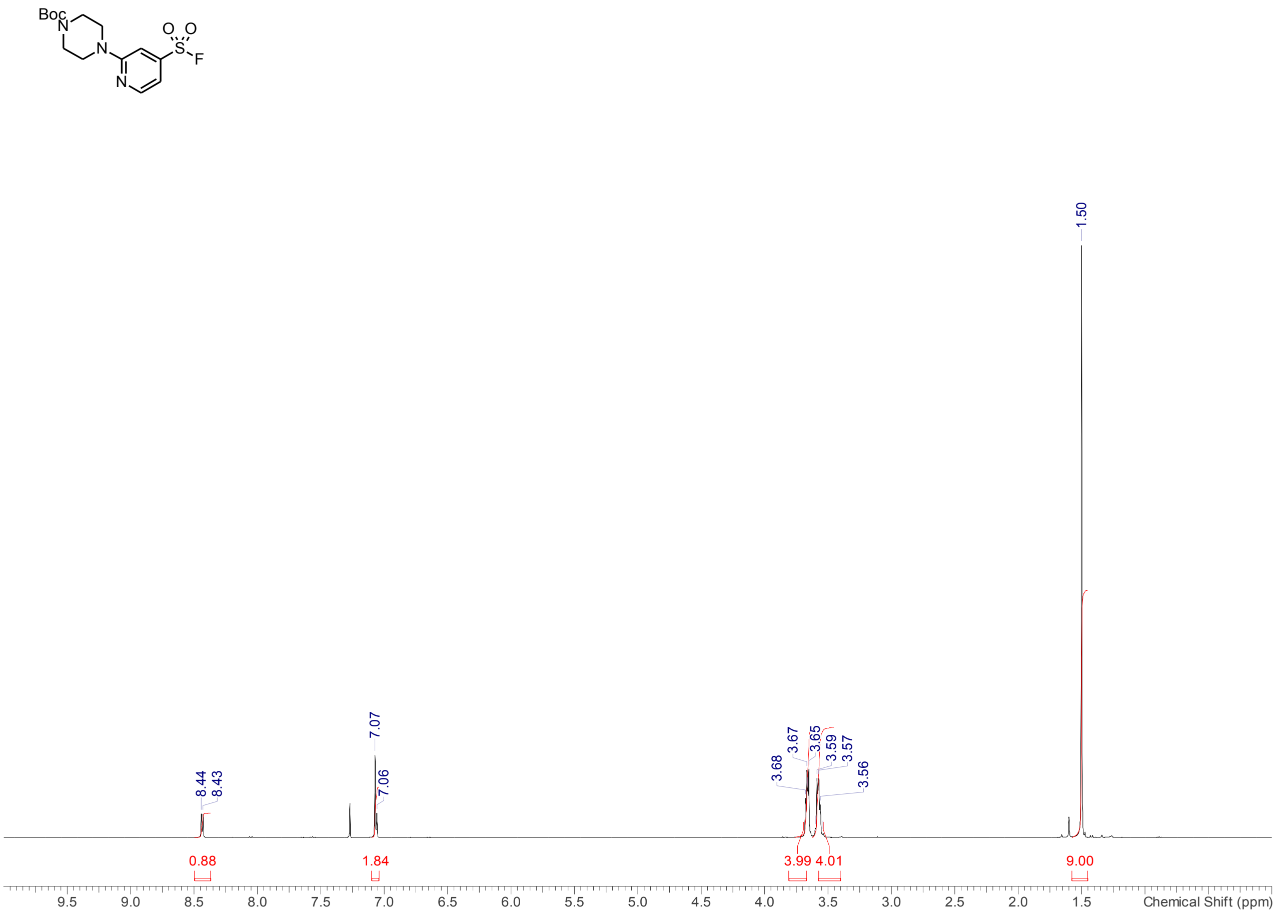
Boc
N
N
N
O O
S
F
1.50
7.07
7.06
8.44
8.43
3.68
3.67
3.65
3.59
3.57
3.56
0.88
1.84
3.99
4.01
9.00
9.5 9.0 8.5 8.0 7.5 7.0 6.5 6.0 5.5 5.0 4.5 4.0 3.5 3.0 2.5 2.0 1.5
Chemical Shift (ppm)

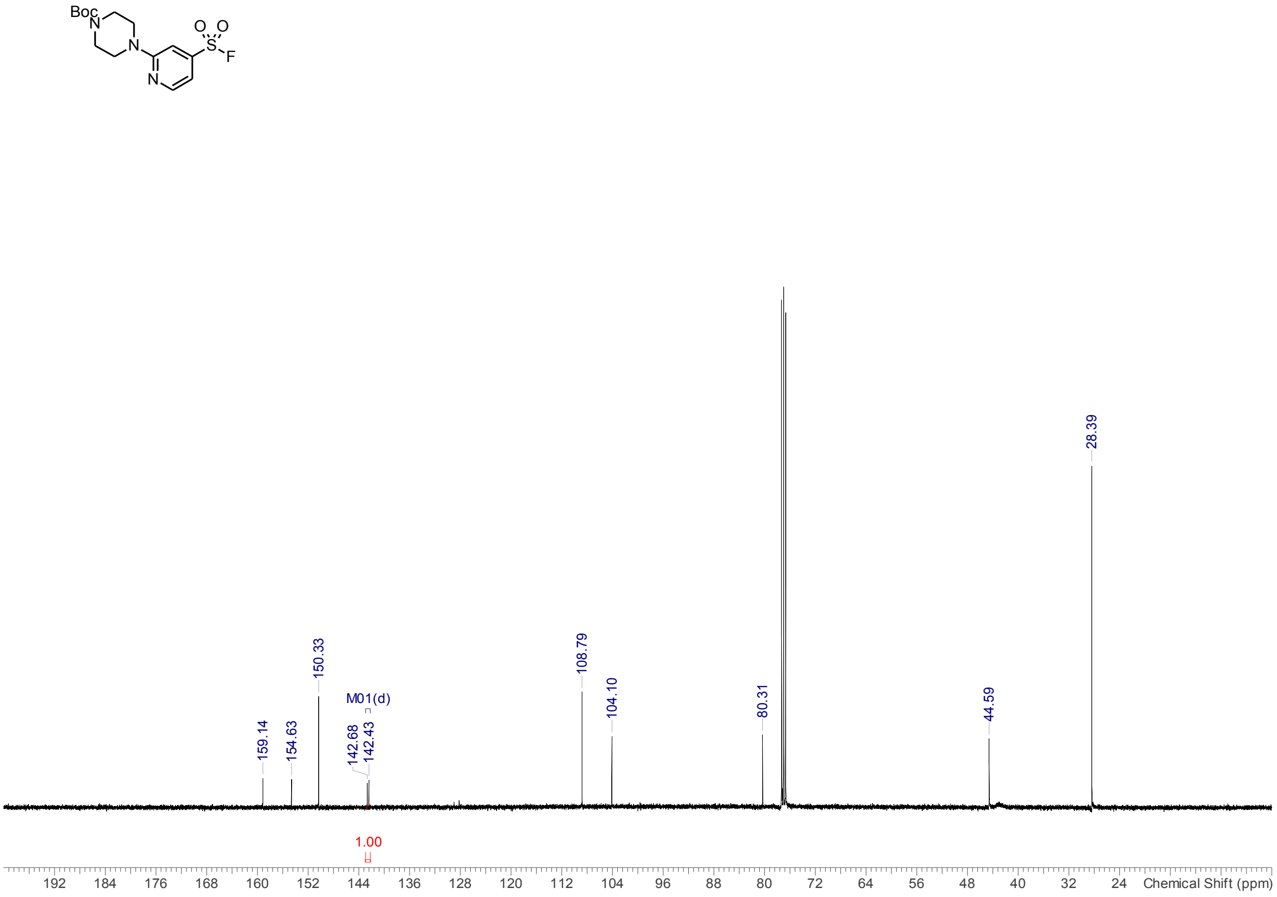
Boc
N
N
N
O O
S
F
28.39
150.33
108.79
M01(d)
104.10
80.31
44.59
159.14
154.63
142.68
142.43
1.00
192 184 176 168 160 152 144 136 128 120 112 104 96 88 80 72 64 56 48 40 32 24
Chemical Shift (ppm)

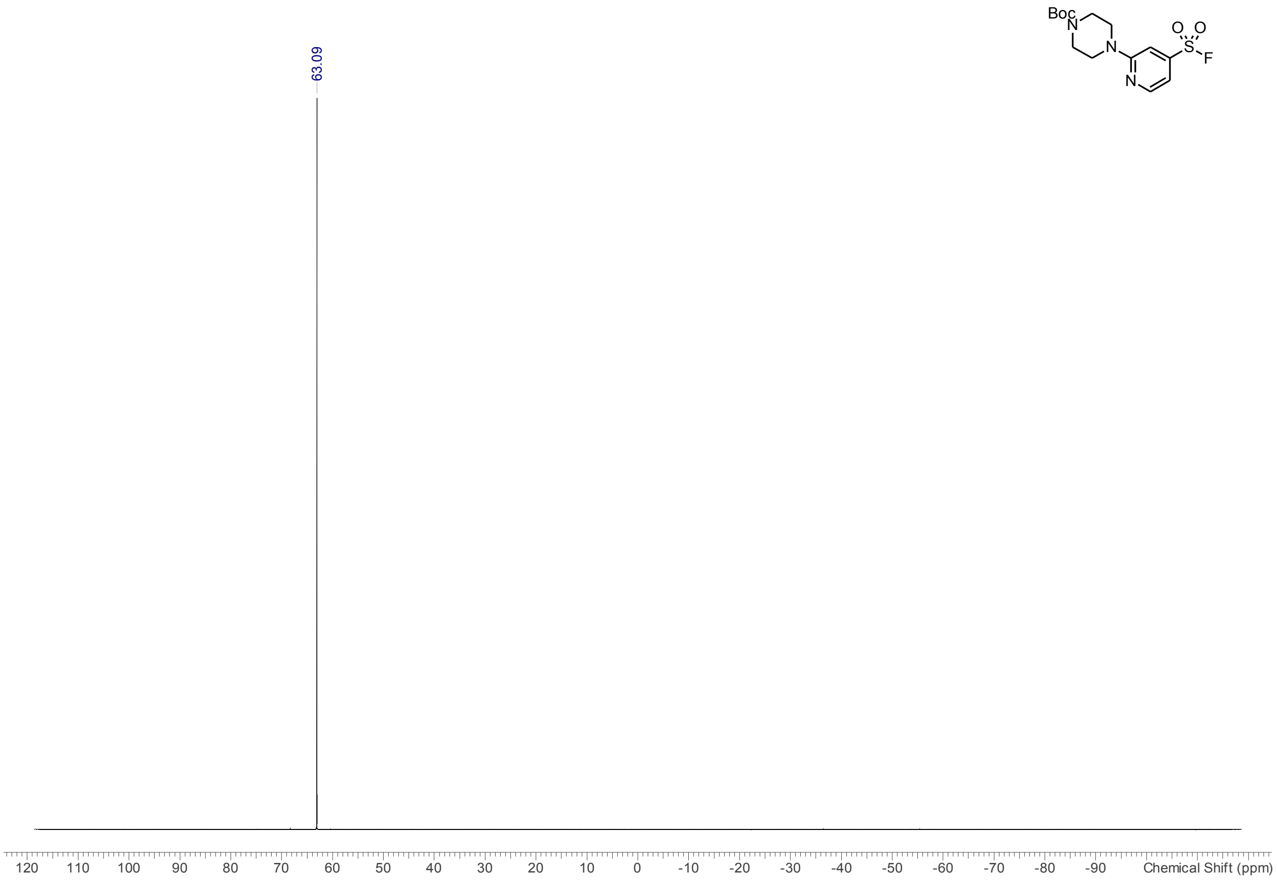
Boc
N
N
N
O
O
S
F
63.09
120
110
100
90
80
70
60
50
40
30
20
10
0
-10
-20
-30
-40
-50
-60
-70
-80
-90
Chemical Shift (ppm)

3.4 NMR Spectra of Sulfonyl Fluoride-Containing Active Pharmaceutical Ingredients

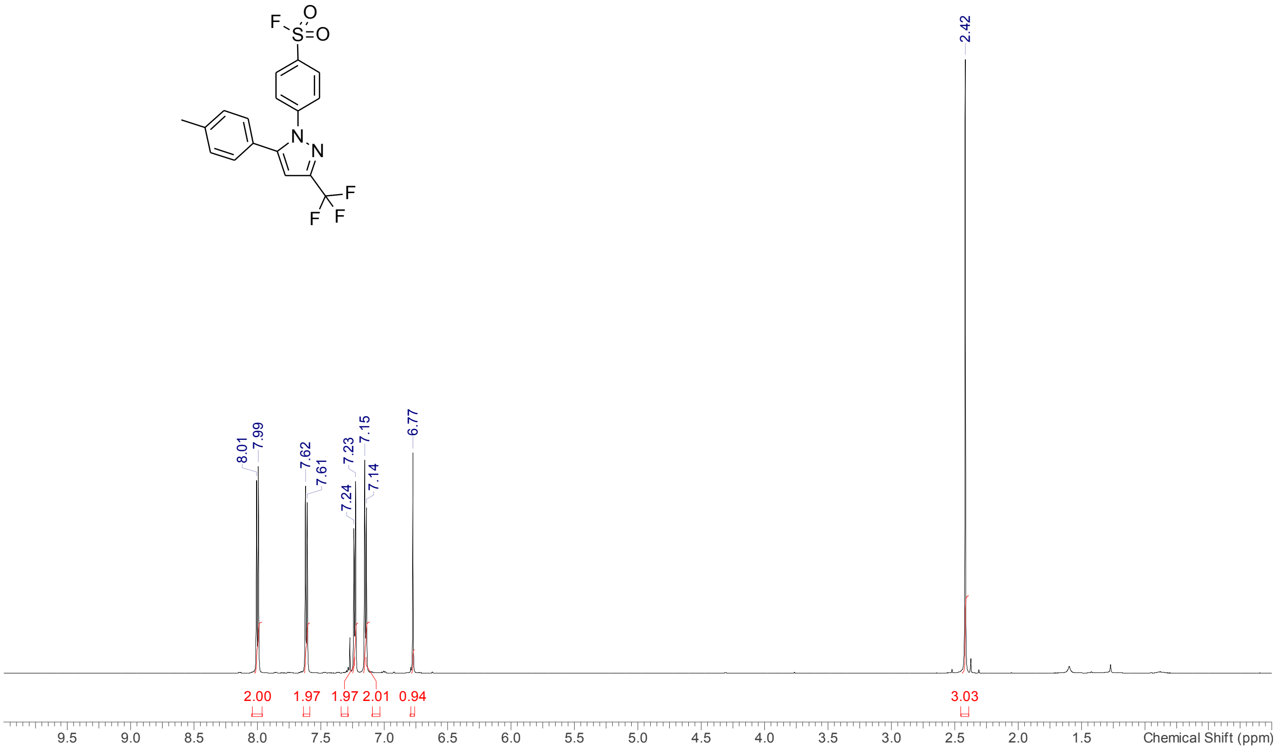

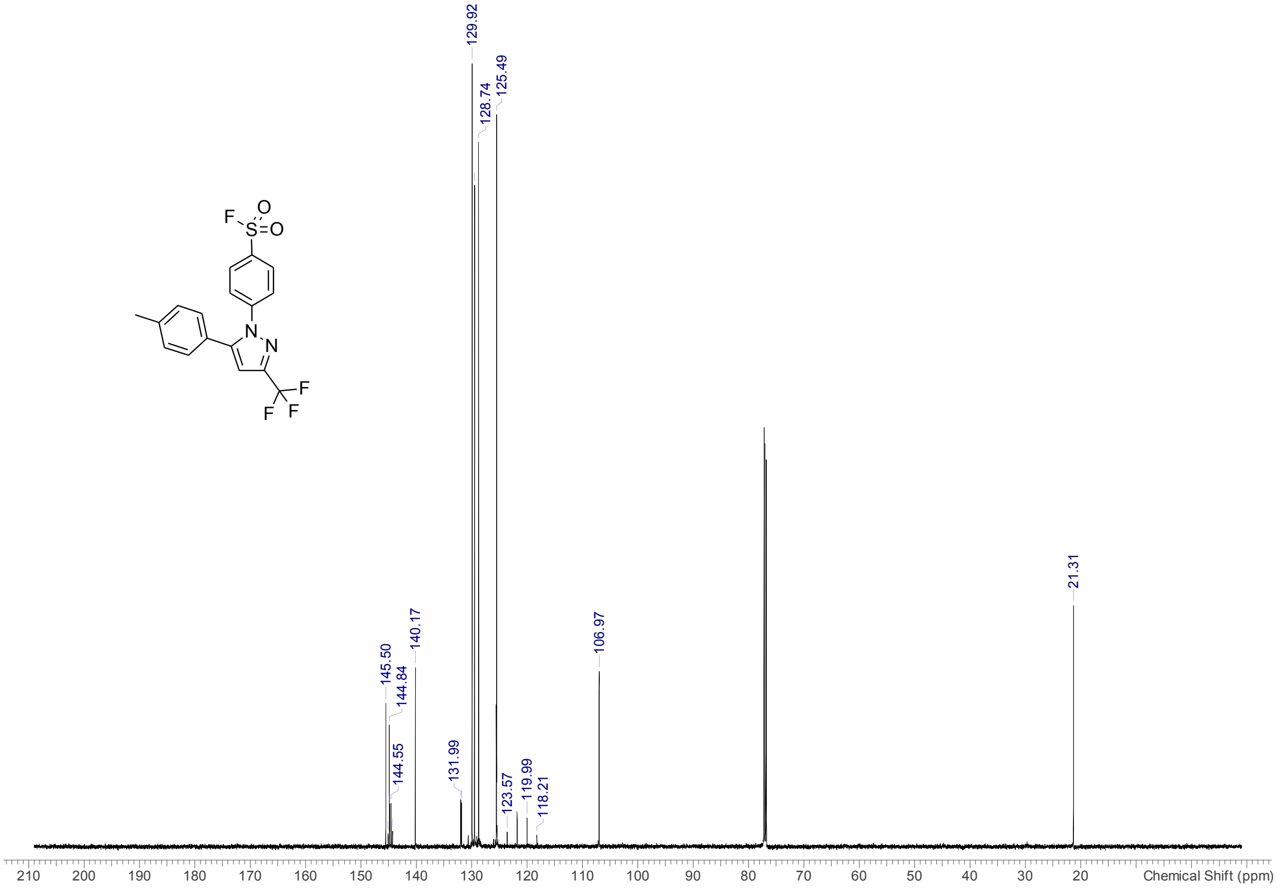

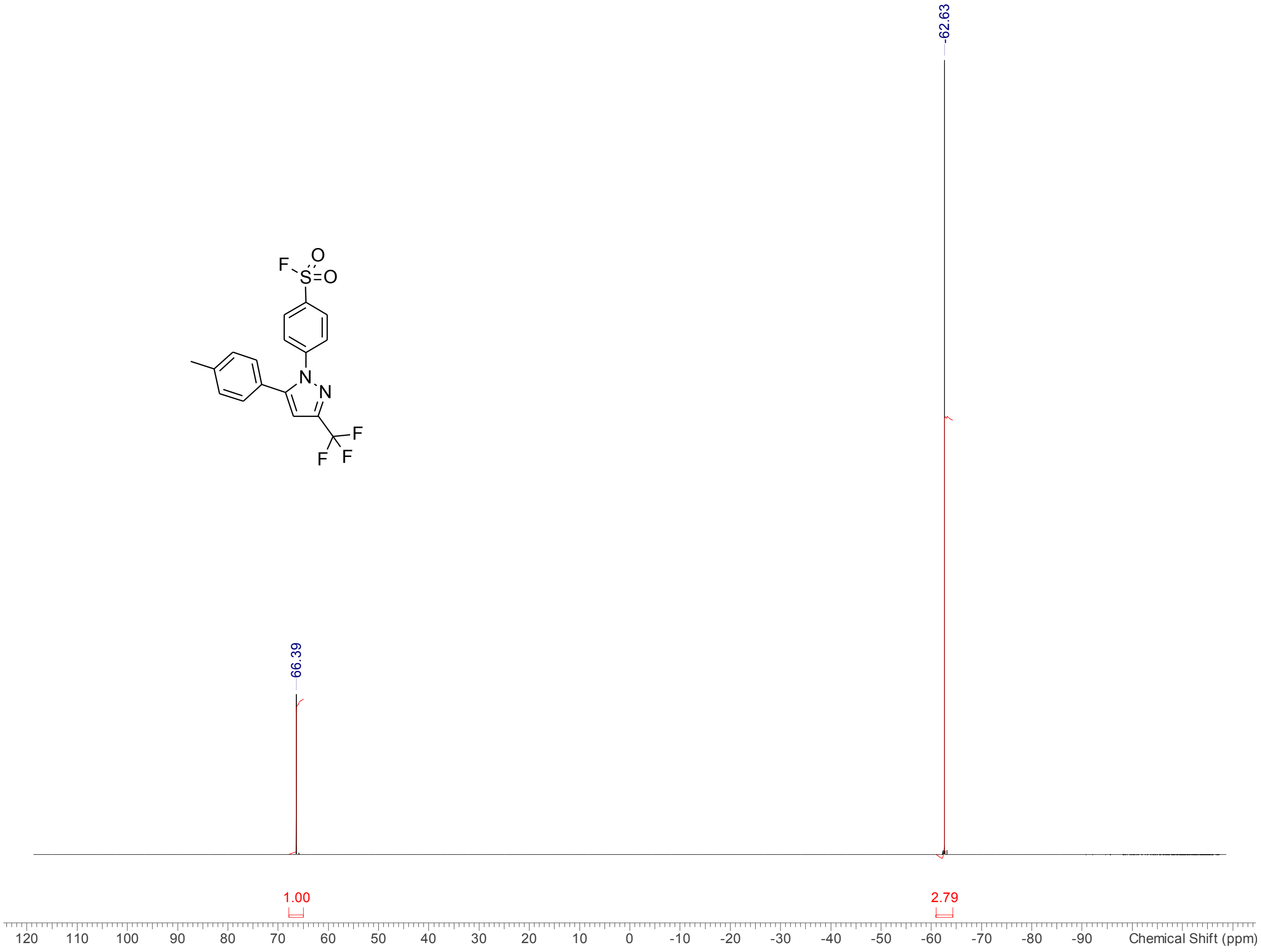
-62.63
66.39
1.00
2.79
120 110 100 90 80 70 60 50 40 30 20 10 0 -10 -20 -30 -40 -50 -60 -70 -80 -90
Chemical Shift (ppm)

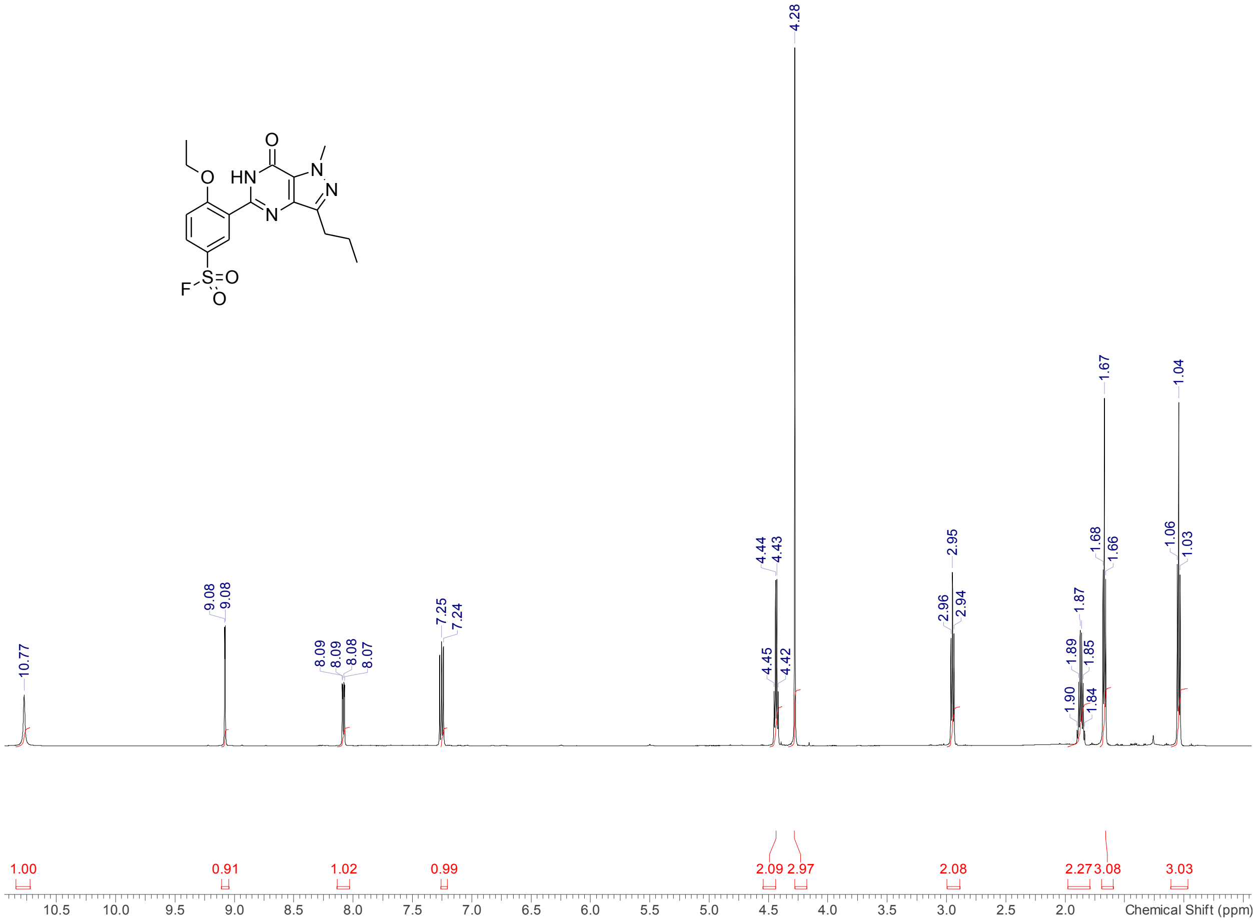
HN
N
N
N
O
O
F
S
O
O
10.77
9.08
9.08
8.09
8.09
8.08
8.07
7.25
7.24
4.45
4.44
4.43
4.42
4.28
2.96
2.95
2.94
1.90
1.89
1.87
1.85
1.84
1.68
1.67
1.66
1.06
1.04
1.03
1.00
0.91
1.02
0.99
2.09
2.97
2.08
2.27
3.08
3.03
Chemical Shift (ppm)

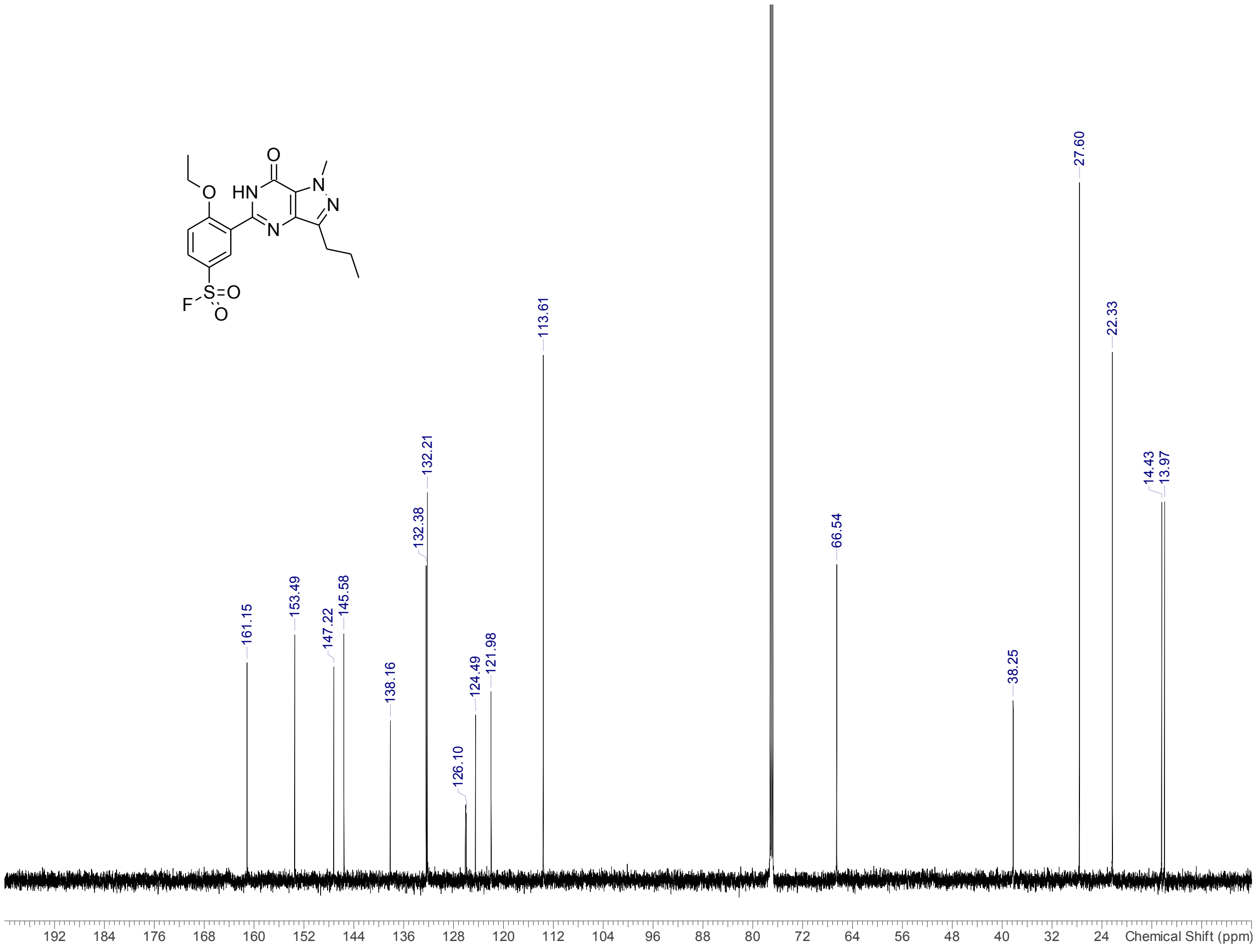
HN
N
N
N
O
O
F
S
O
O
161.15
153.49
147.22
145.58
138.16
132.38
132.21
126.10
124.49
121.98
113.61
66.54
38.25
27.60
22.33
14.43
13.97
Chemical Shift (ppm)

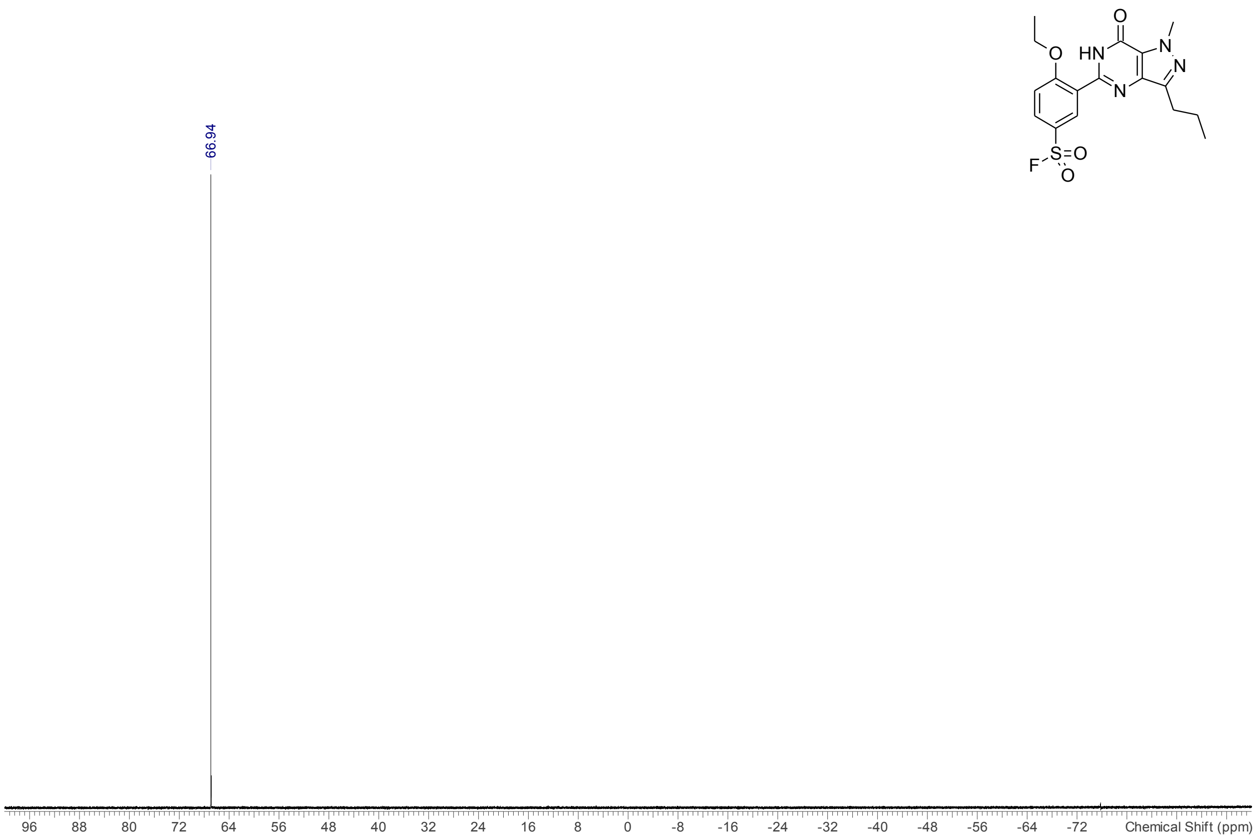
66.94
96 88 80 72 64 56 48 40 32 24 16 8 0 -8 -16 -24 -32 -40 -48 -56 -64 -72
Chemical Shift (ppm)

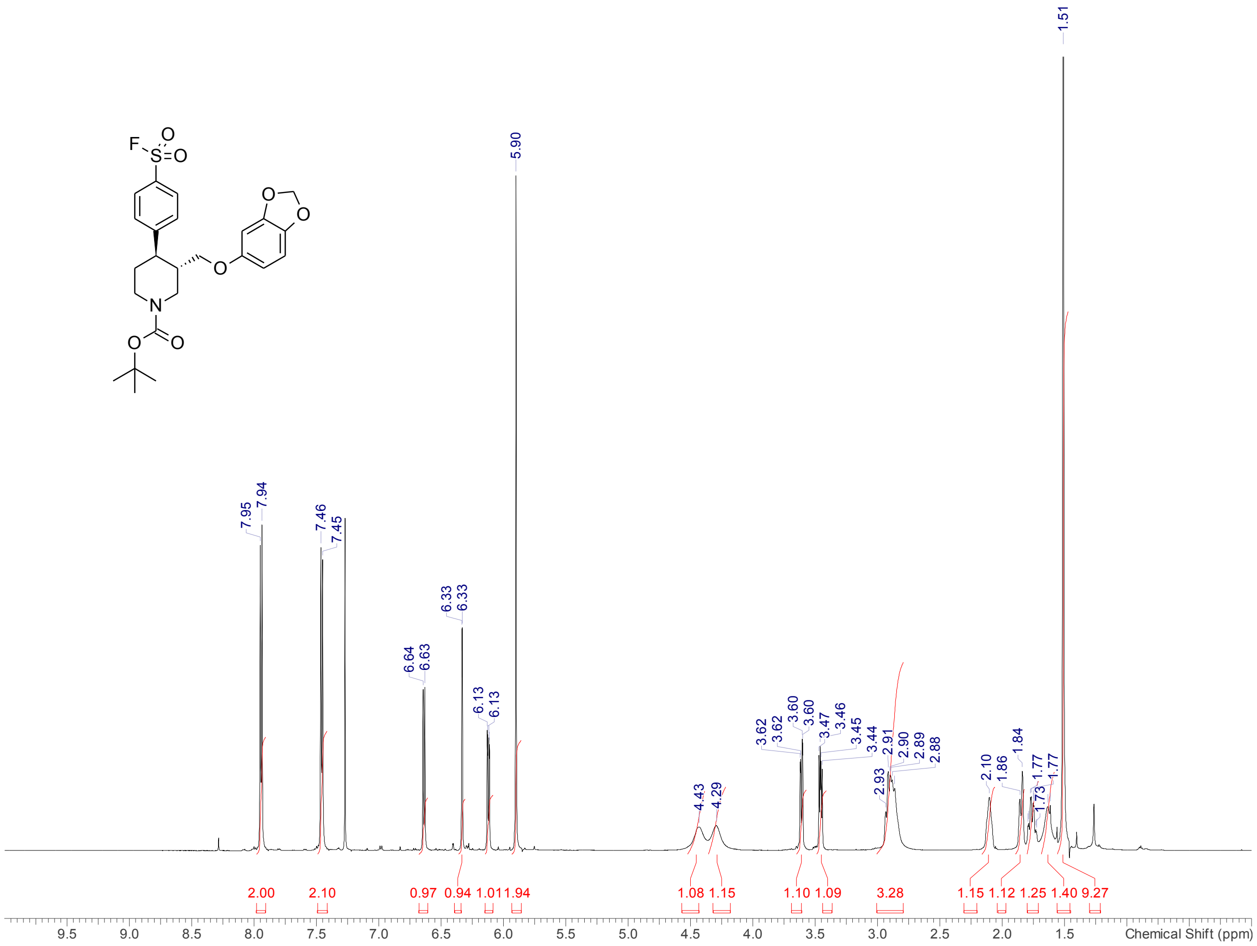

7.95
7.94
7.46
7.45
6.64
6.63
6.33
6.33
6.13
6.13
5.90
4.43
4.29
3.62
3.62
3.60
3.60
3.47
3.46
3.45
3.44
2.93
2.91
2.90
2.89
2.88
2.10
1.86
1.84
1.77
1.77
1.73
1.51
2.00
2.10
0.97
0.94
1.01
1.94
1.08
1.15
1.10
1.09
3.28
1.15
1.12
1.25
1.40
9.27
Chemical Shift (ppm)

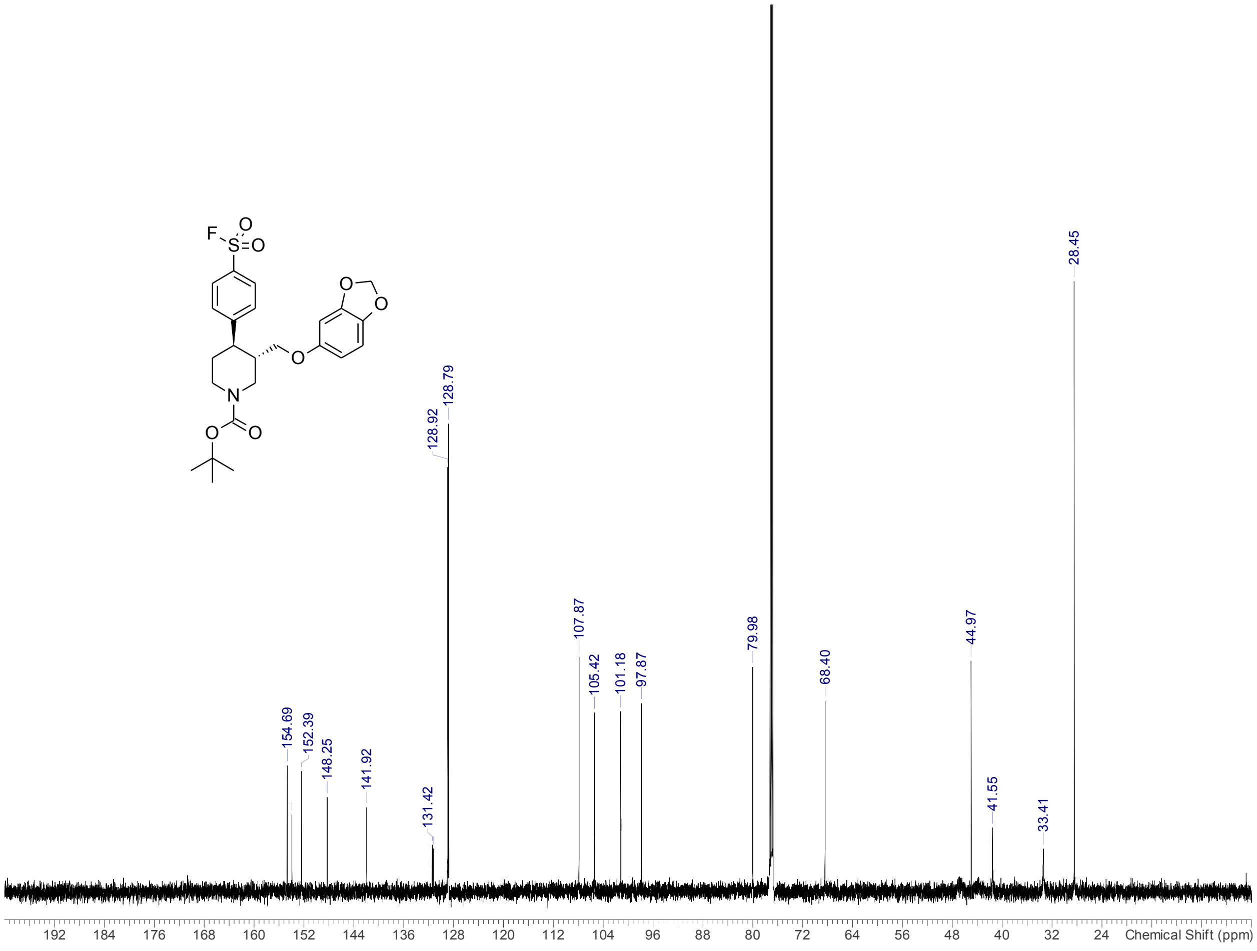

154.69
152.39
148.25
141.92
131.42
128.92
128.79
107.87
105.42
101.18
97.87
79.98
68.40
44.97
41.55
33.41
28.45
Chemical Shift (ppm)

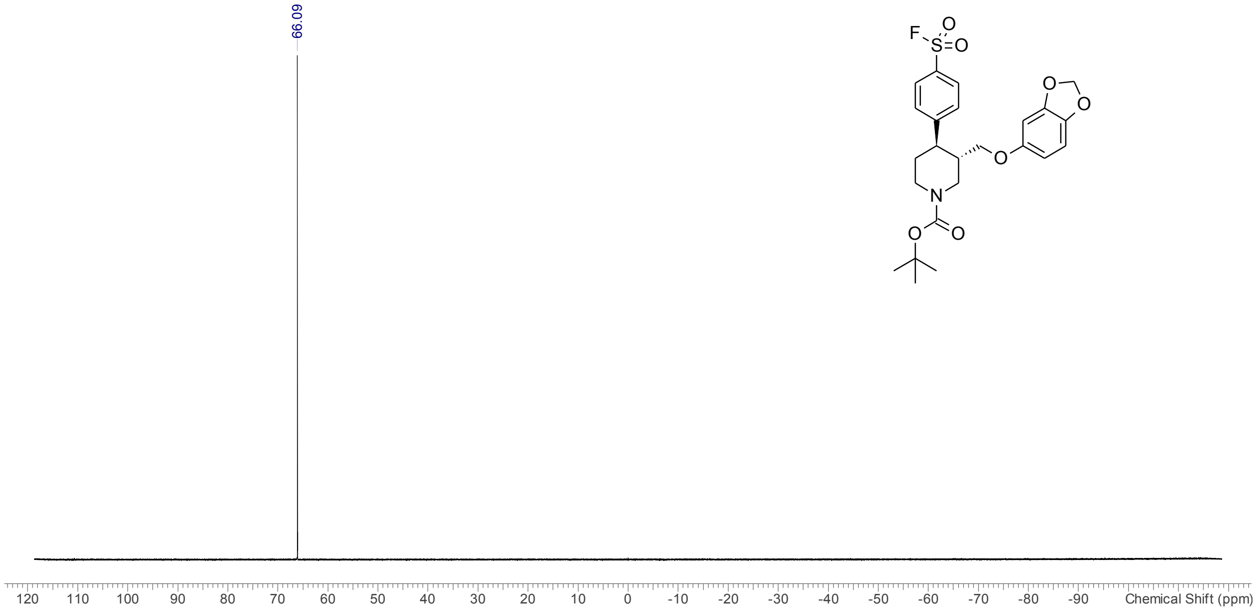
66.09
F
S
O
O
N
O
O
O
O
O
120 110 100 90 80 70 60 50 40 30 20 10 0 -10 -20 -30 -40 -50 -60 -70 -80 -90
Chemical Shift (ppm)

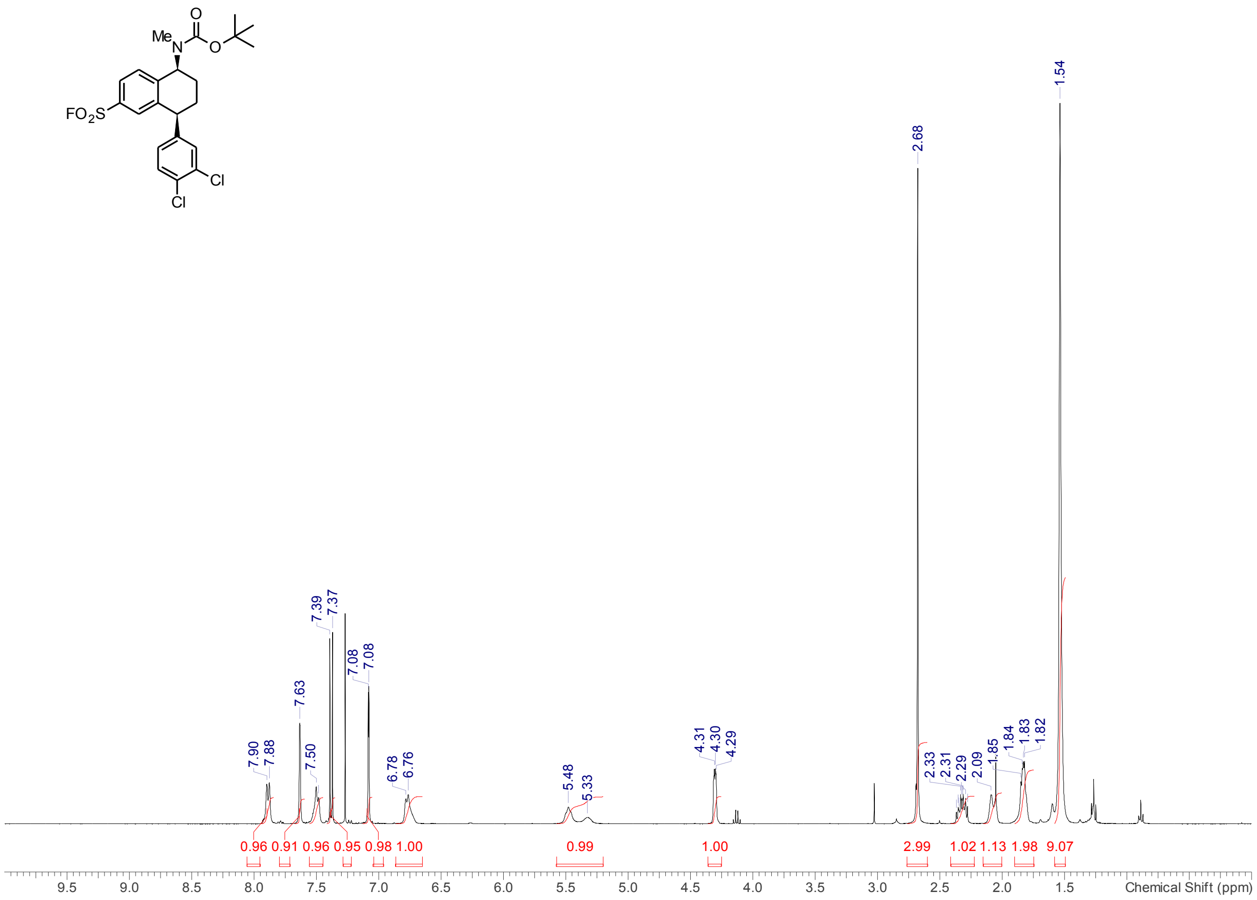
Me
N
O
O
FO_2S
Cl
Cl
7.90
7.88
7.63
7.50
7.39
7.37
7.08
7.08
6.78
6.76
5.48
5.33
4.31
4.30
4.29
2.68
2.33
2.31
2.29
2.09
1.85
1.84
1.83
1.82
1.54
0.96 0.91 0.96 0.95 0.98 1.00
0.99
1.00
2.99 1.02 1.13 1.98 9.07
9.5 9.0 8.5 8.0 7.5 7.0 6.5 6.0 5.5 5.0 4.5 4.0 3.5 3.0 2.5 2.0 1.5
Chemical Shift (ppm)

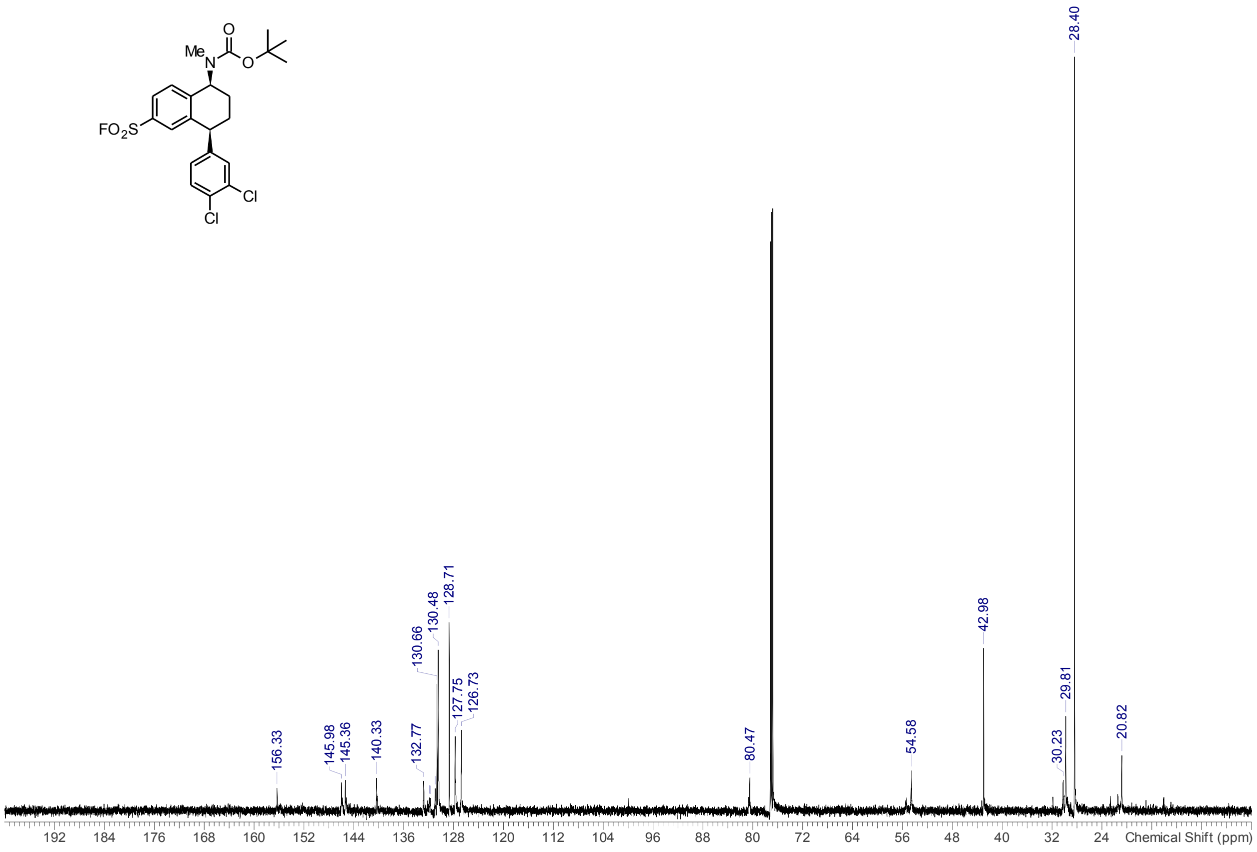
Me
N
O
O
FO_2S
Cl
Cl
156.33
145.98
145.36
140.33
132.77
130.66
130.48
128.71
127.75
126.73
80.47
54.58
42.98
30.23
29.81
28.40
20.82
192 184 176 168 160 152 144 136 128 120 112 104 96 88 80 72 64 56 48 40 32 24
Chemical Shift (ppm)

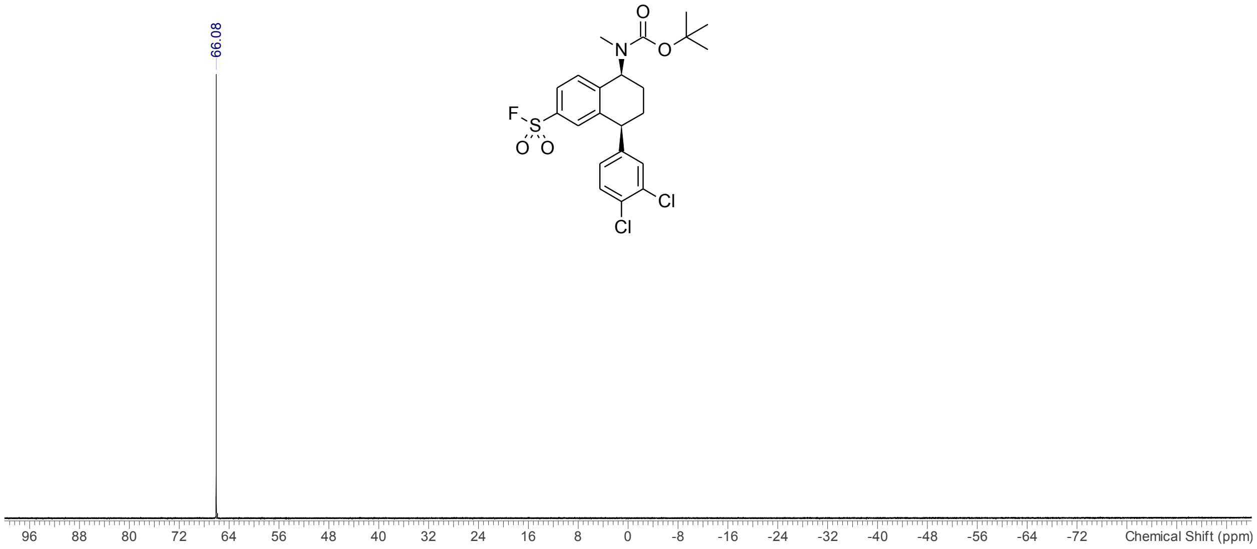
66.08
F
S
O O
N
O
O
Cl
Cl
96 88 80 72 64 56 48 40 32 24 16 8 0 -8 -16 -24 -32 -40 -48 -56 -64 -72
Chemical Shift (ppm)

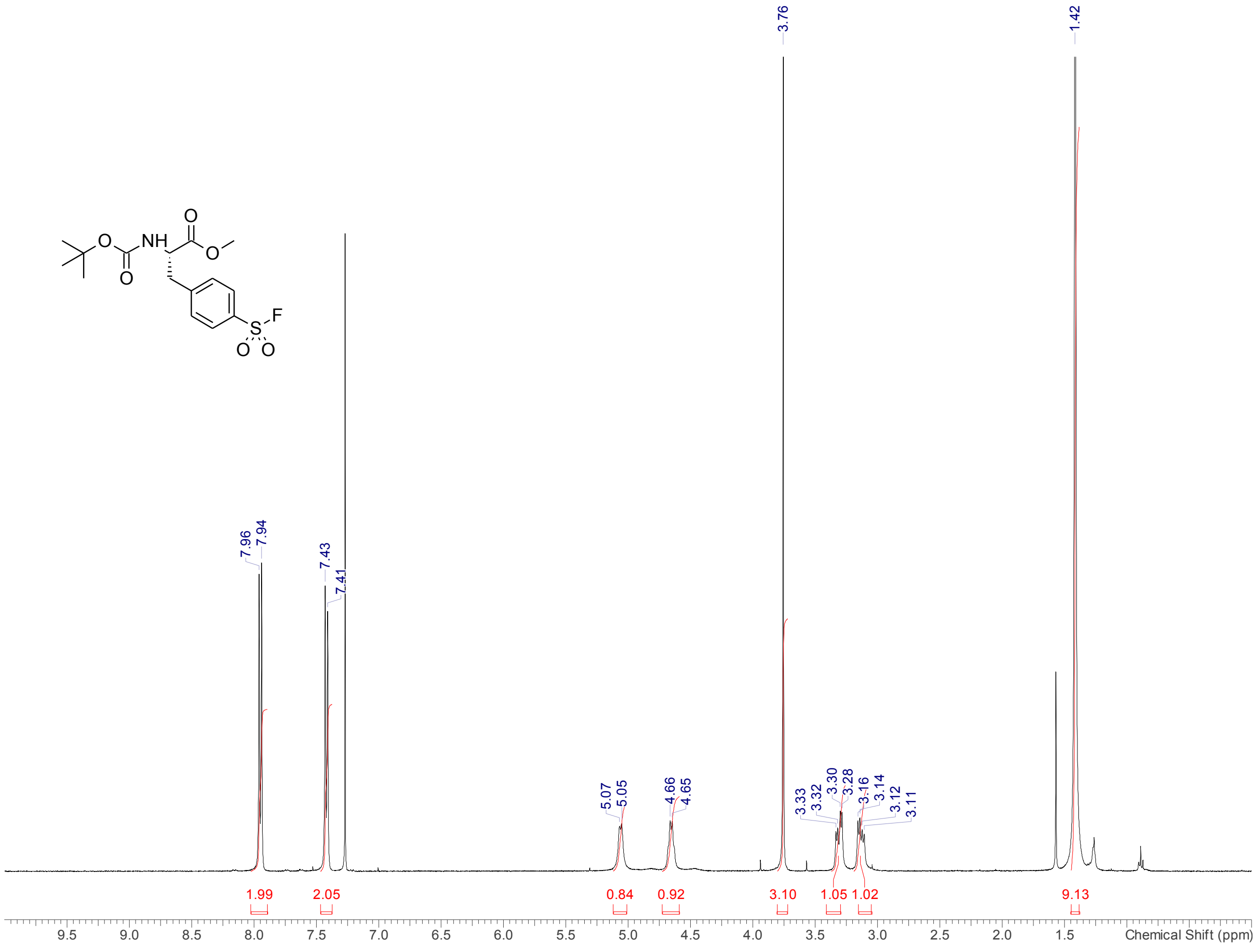
NH
O
O
O
S
F
O O
7.96
7.94
7.43
7.41
5.07
5.05
4.66
4.65
3.76
3.33
3.32
3.30
3.28
3.16
3.14
3.12
3.11
1.42
1.99
2.05
0.84
0.92
3.10
1.05
1.02
9.13
9.5 9.0 8.5 8.0 7.5 7.0 6.5 6.0 5.5 5.0 4.5 4.0 3.5 3.0 2.5 2.0 1.5
Chemical Shift (ppm)

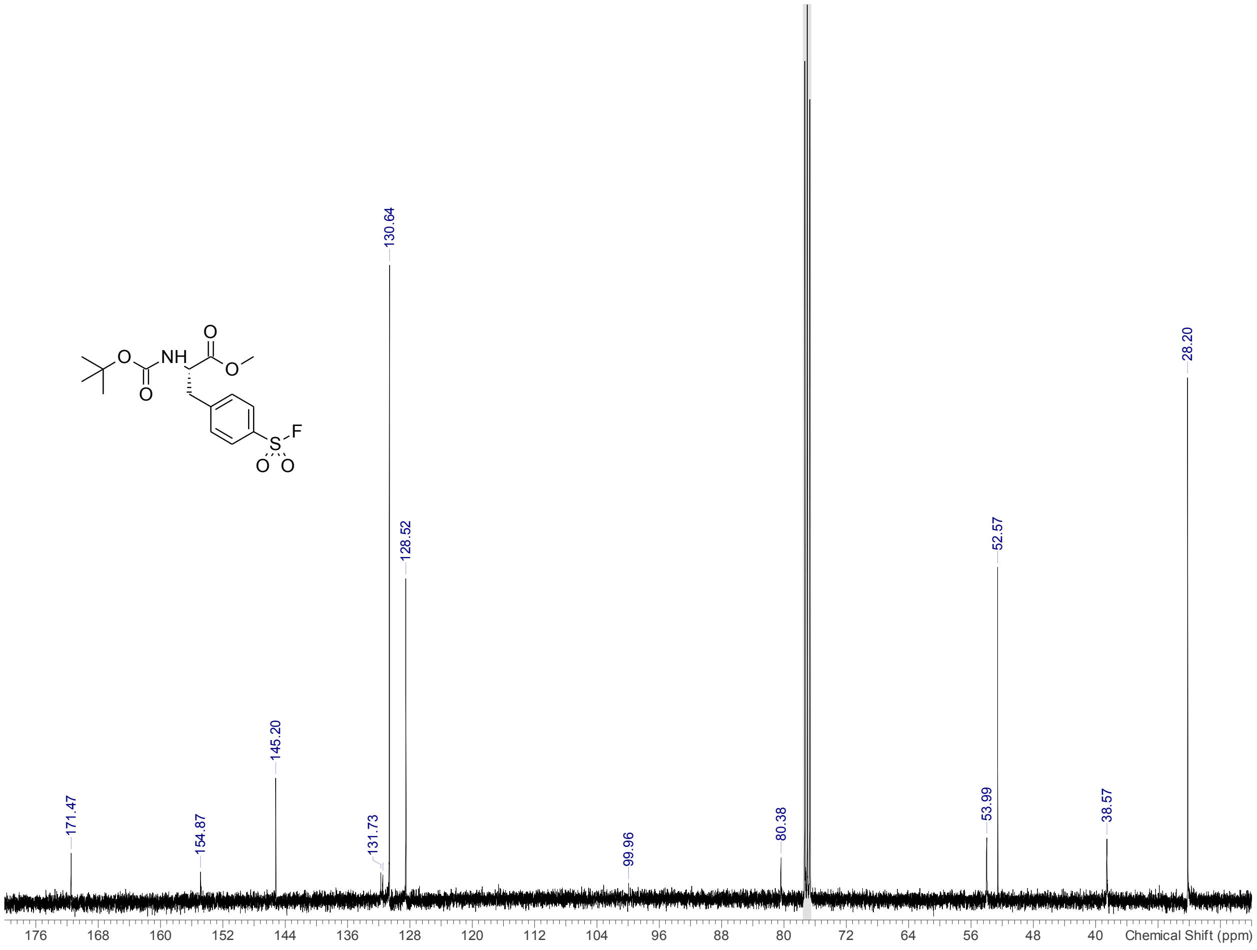
NH
O
O
O
S
F
O O
171.47
154.87
145.20
131.73
130.64
128.52
99.96
80.38
53.99
52.57
38.57
28.20
176 168 160 152 144 136 128 120 112 104 96 88 80 72 64 56 48 40
Chemical Shift (ppm)

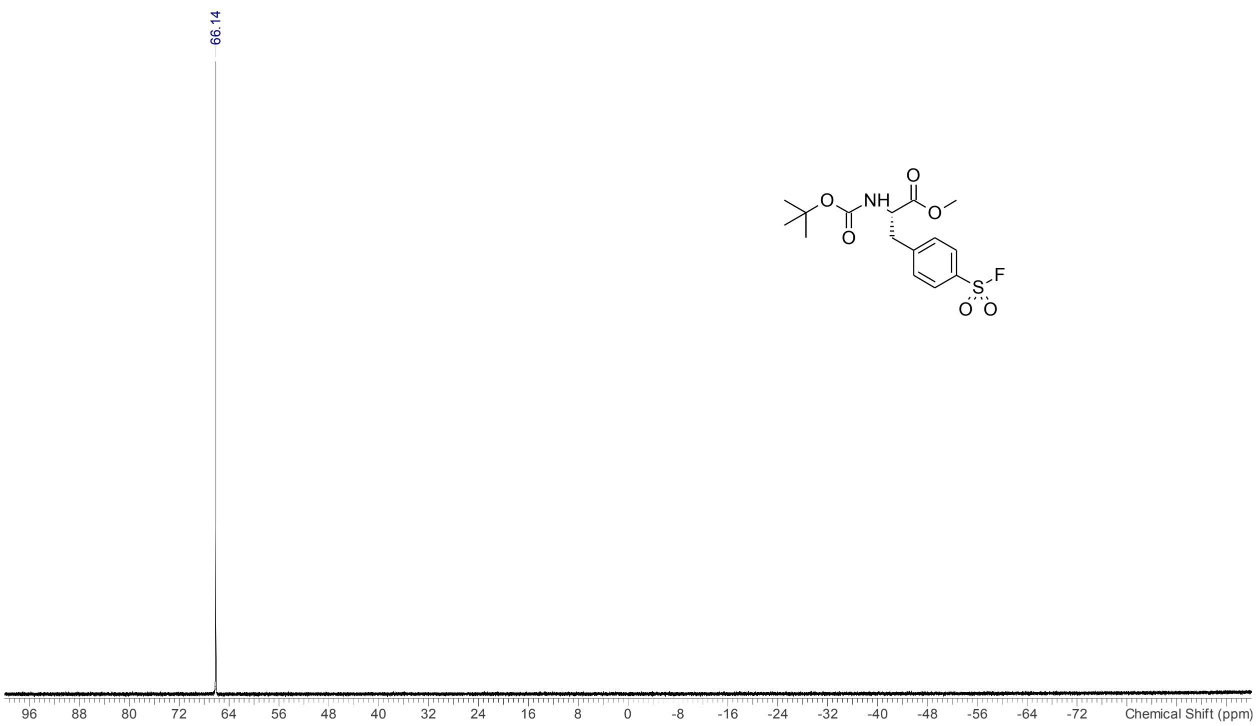
66.14
Chemical Shift (ppm)
96 88 80 72 64 56 48 40 32 24 16 8 0 -8 -16 -24 -32 -40 -48 -56 -64 -72

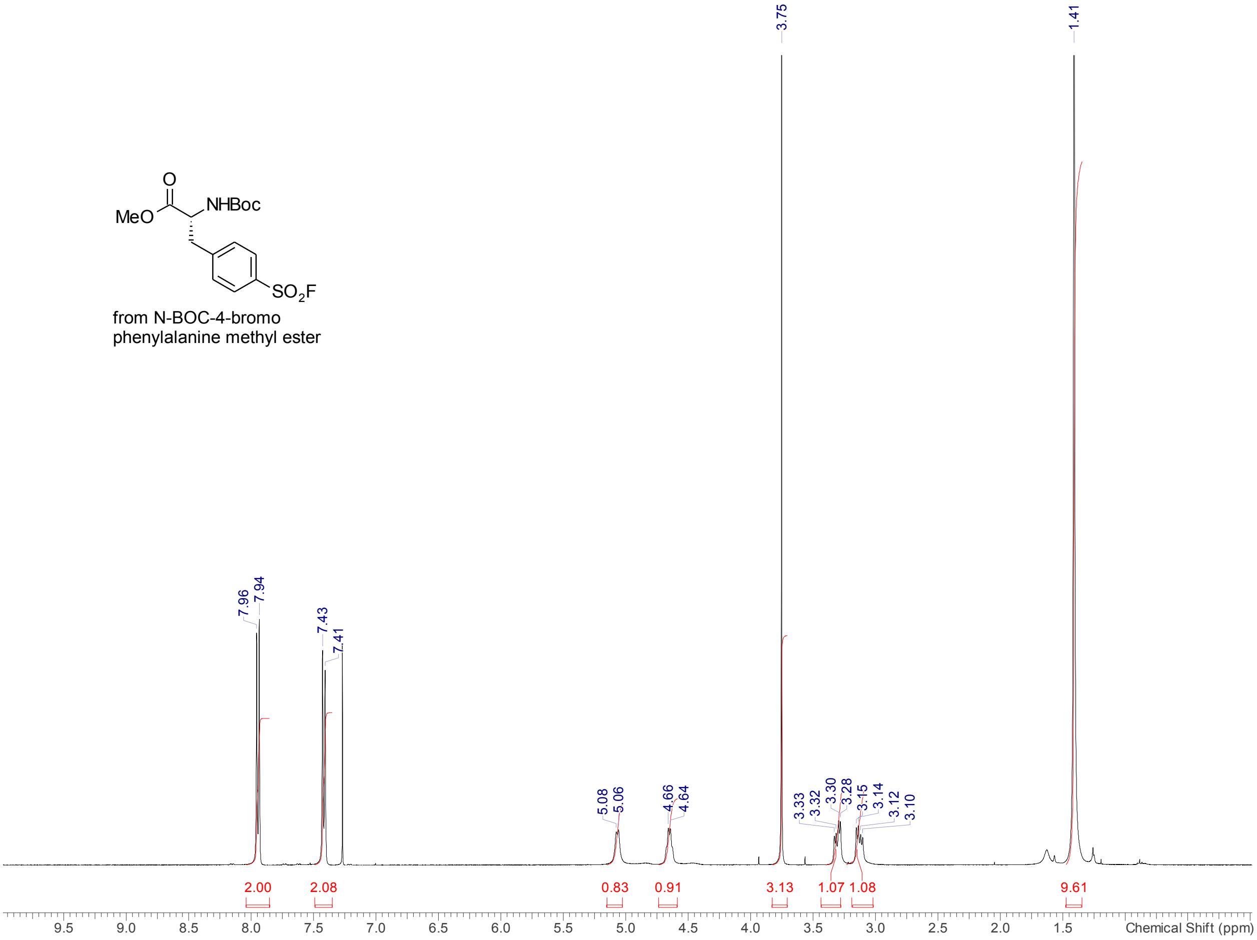
MeO
NHBoc
SO2F
from N-BOC-4-bromo
phenylalanine methyl ester
7.96 7.94 7.43 7.41 5.08 5.06 4.66 4.64 3.75 3.33 3.32 3.30 3.28 3.15 3.14 3.12 3.10 1.41
2.00 2.08 0.83 0.91 3.13 1.07 1.08 9.61
9.5 9.0 8.5 8.0 7.5 7.0 6.5 6.0 5.5 5.0 4.5 4.0 3.5 3.0 2.5 2.0 1.5
Chemical Shift (ppm)

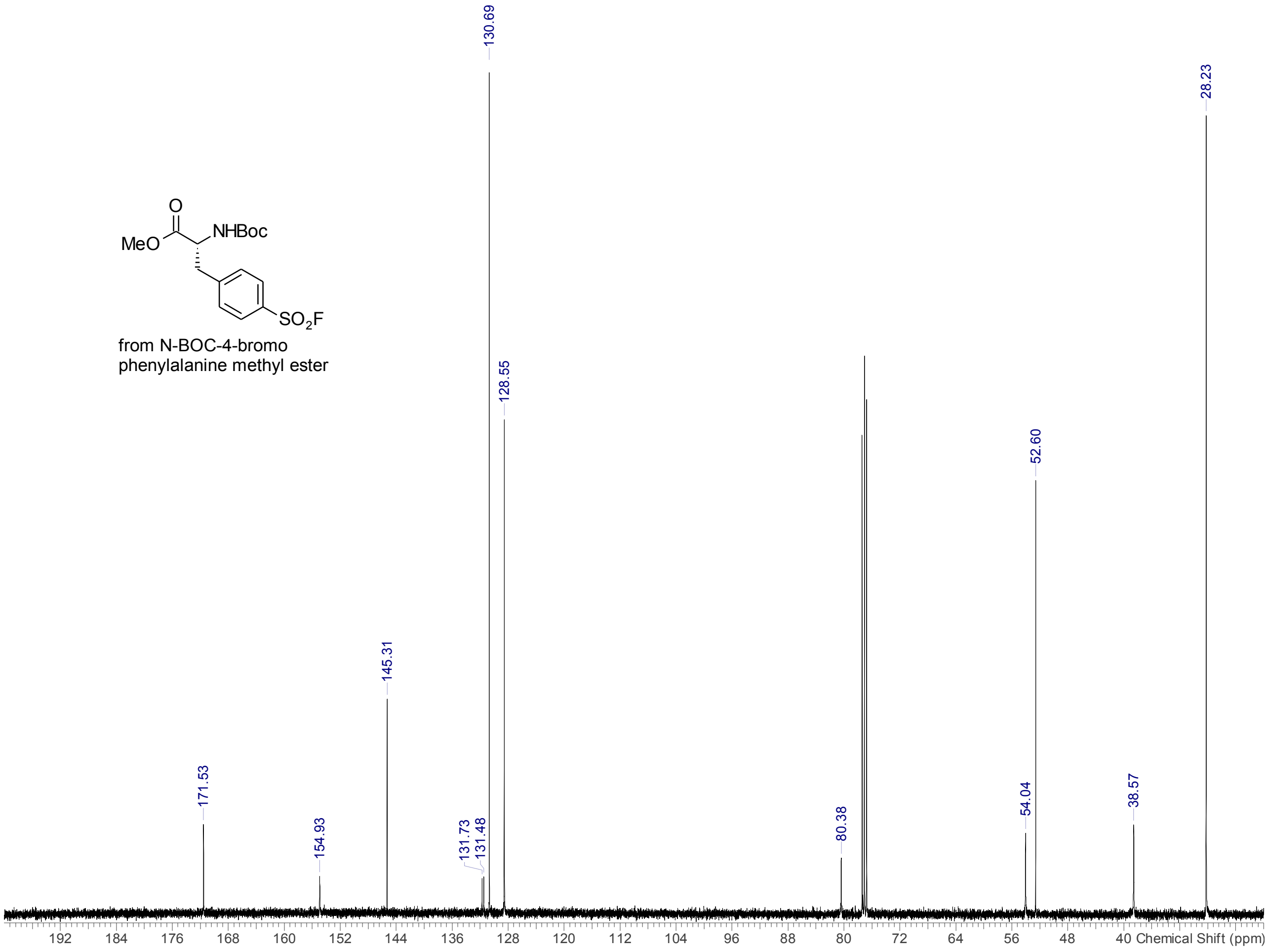
MeO
O
NHBoc
SO2F
from N-BOC-4-bromo
phenylalanine methyl ester
171.53
154.93
145.31
131.73
131.48
130.69
128.55
80.38
54.04
52.60
38.57
28.23
192 184 176 168 160 152 144 136 128 120 112 104 96 88 80 72 64 56 48 40 Chemical Shift (ppm)

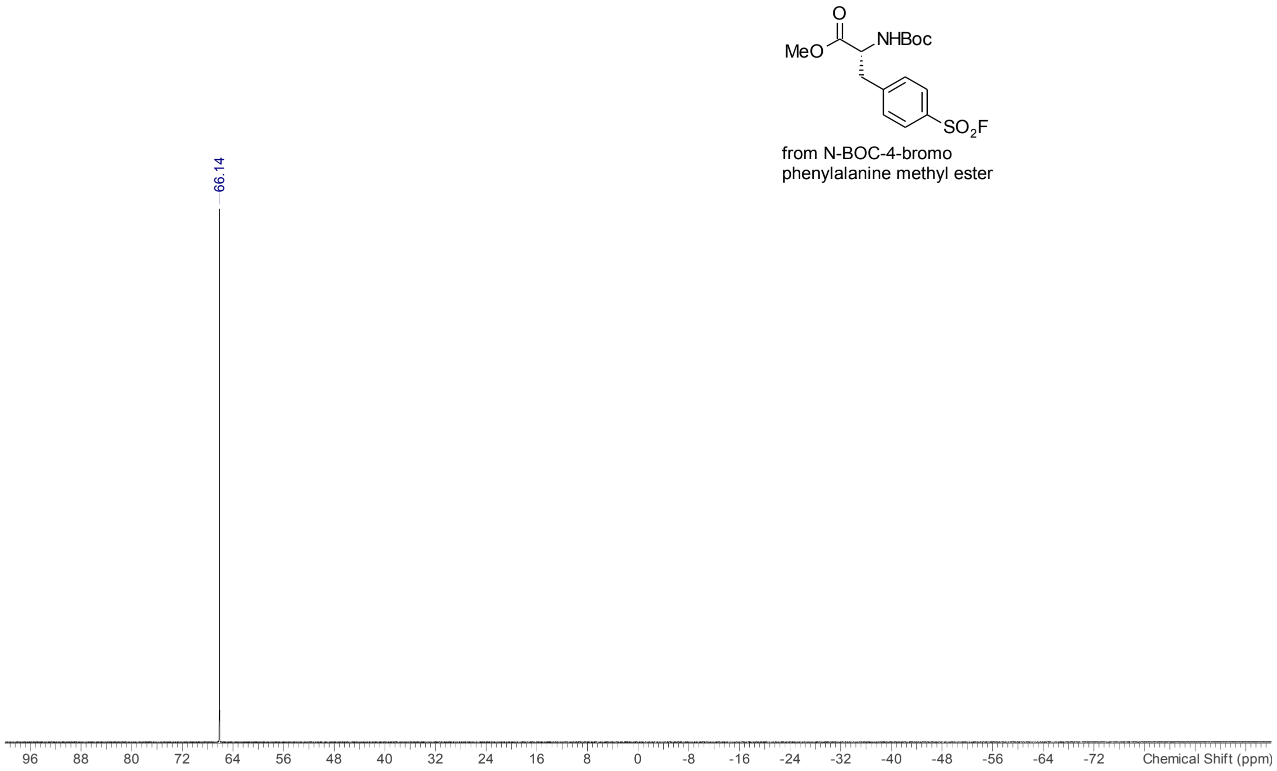
MeO
O
NHBoc
SO2F
from N-BOC-4-bromo
phenylalanine methyl ester
66.14
96 88 80 72 64 56 48 40 32 24 16 8 0 -8 -16 -24 -32 -40 -48 -56 -64 -72 Chemical Shift (ppm)

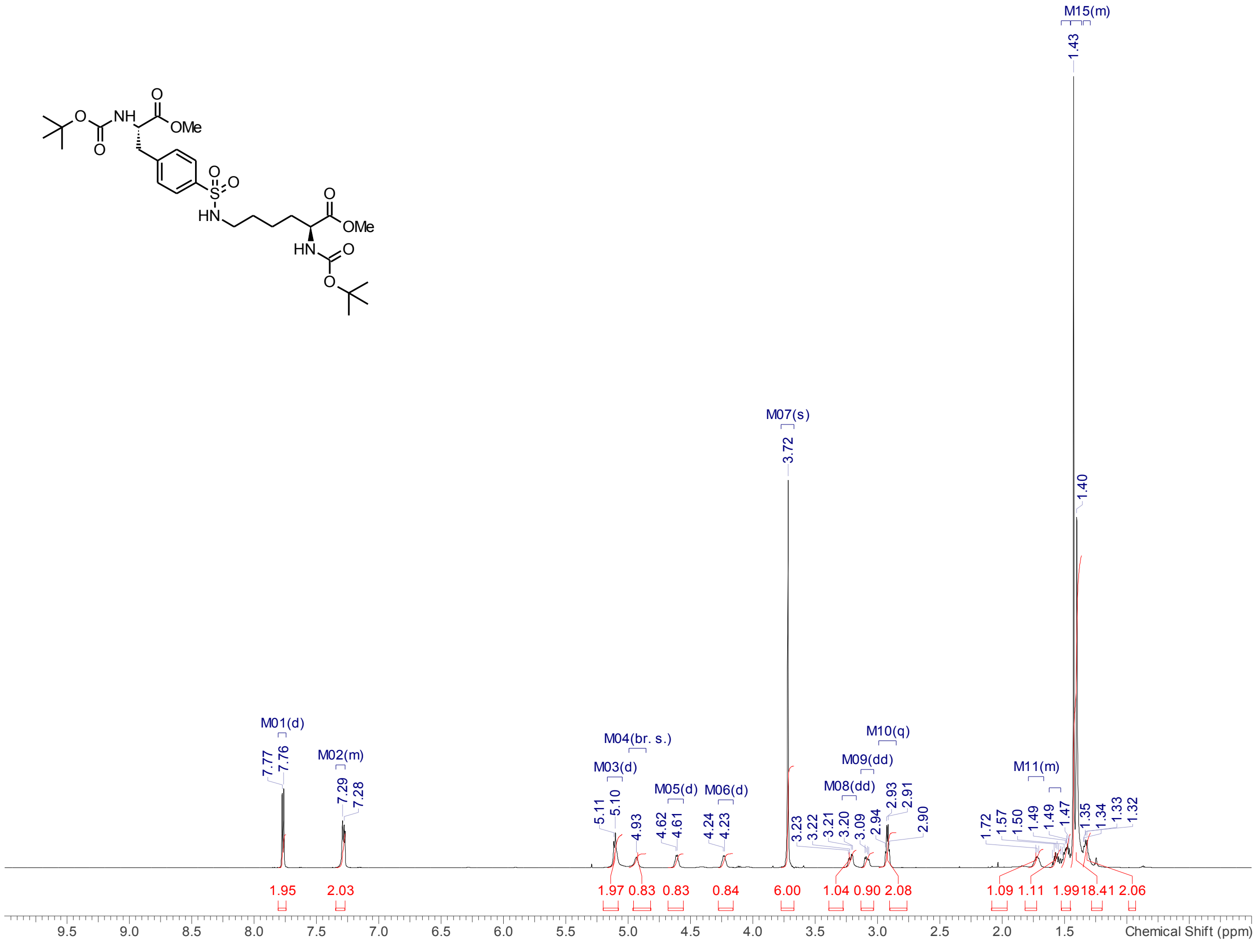

M01(d)
7.77
7.76
M02(m)
7.29
7.28
M03(d)
5.11
5.10
M04(br. s.)
4.93
M05(d)
4.62
4.61
M06(d)
4.24
4.23
M07(s)
3.72
M08(dd)
M09(dd)
M10(q)
3.23
3.22
3.21
3.20
3.09
2.94
2.93
2.91
2.90
M11(m)
1.72
1.57
1.50
1.49
1.49
1.47
M15(m)
1.43
1.40
1.35
1.34
1.33
1.32
1.95
2.03
1.97
0.83
0.83
0.84
6.00
1.04
0.90
2.08
1.09
1.11
1.99
18.41
2.06
Chemical Shift (ppm)

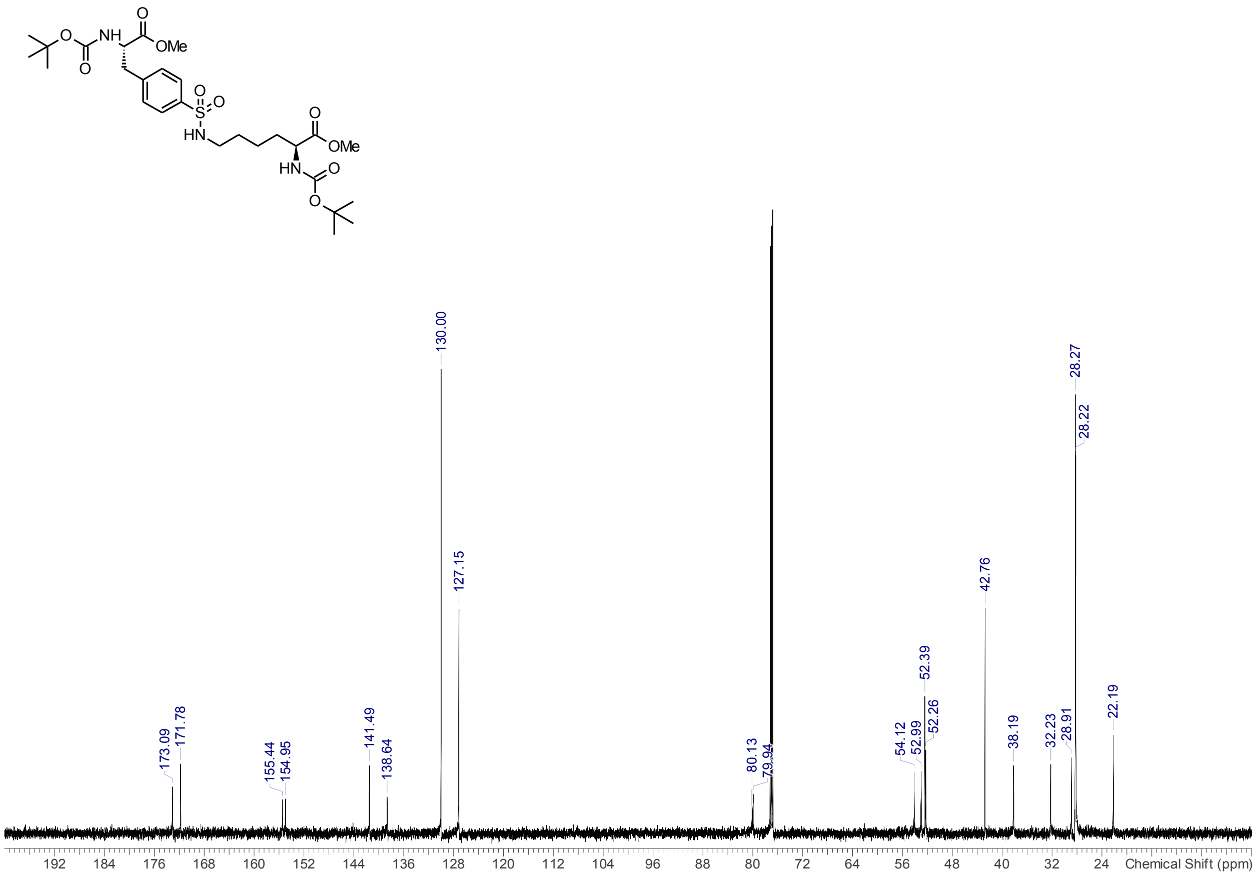

173.09
171.78
155.44
154.95
141.49
138.64
130.00
127.15
80.13
79.94
54.12
52.99
52.39
52.26
42.76
38.19
32.23
28.91
28.27
28.22
22.19
Chemical Shift (ppm)

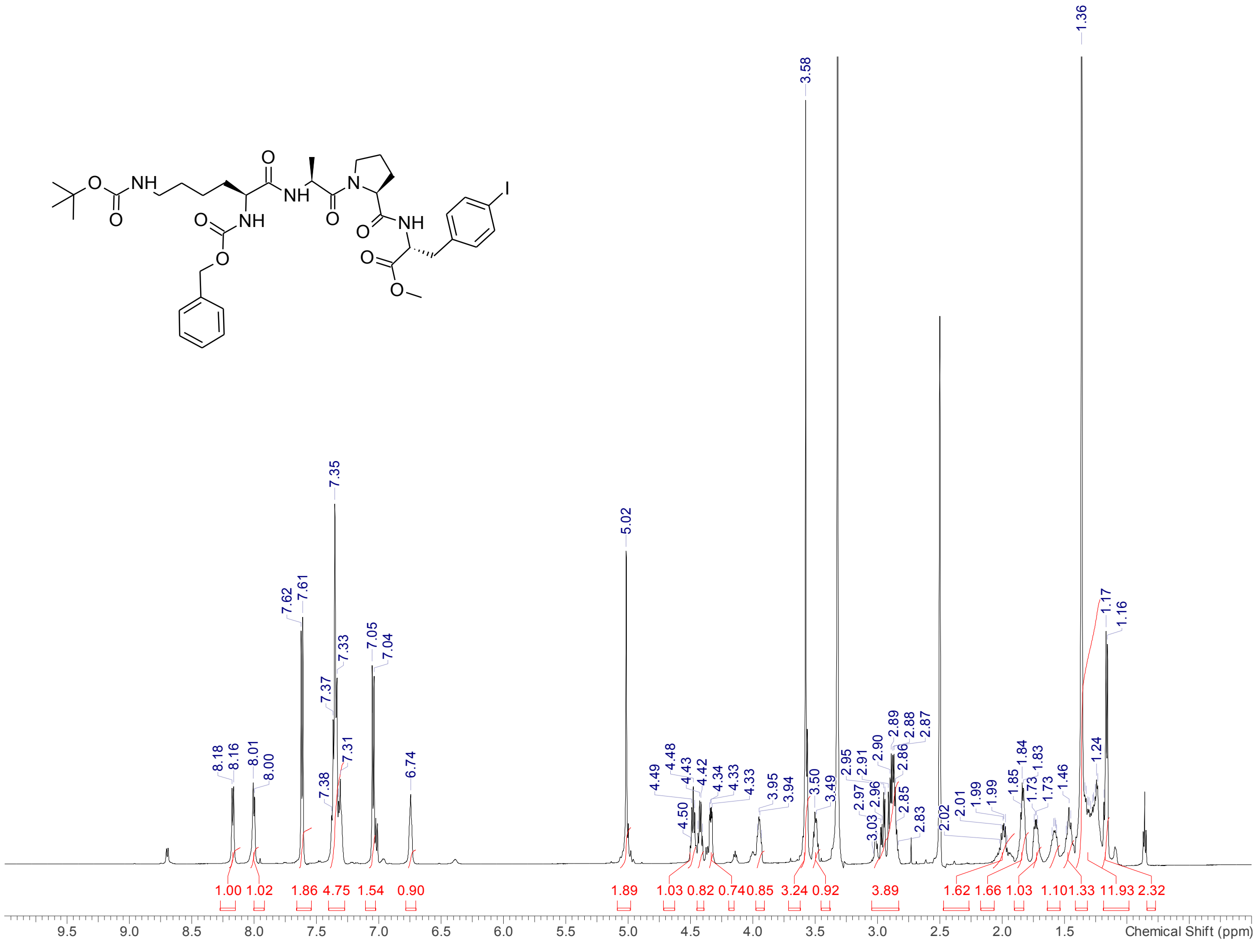
NH
O
O
NH
O
NH
N
O
O
NH
I
O
O
O
8.18 8.16 8.01 8.00 7.62 7.61 7.38 7.37 7.35 7.33 7.31 7.05 7.04 6.74 5.02 4.50 4.49 4.48 4.43 4.42 4.34 4.33 4.33 3.95 3.94 3.58 3.50 3.49 3.03 2.97 2.96 2.95 2.91 2.90 2.89 2.88 2.87 2.86 2.85 2.83 2.02 2.01 1.99 1.99 1.85 1.84 1.83 1.73 1.73 1.46 1.36 1.24 1.17 1.16
1.00 1.02 1.86 4.75 1.54 0.90 1.89 1.03 0.82 0.74 0.85 3.24 0.92 3.89 1.62 1.66 1.03 1.10 1.33 11.93 2.32
9.5 9.0 8.5 8.0 7.5 7.0 6.5 6.0 5.5 5.0 4.5 4.0 3.5 3.0 2.5 2.0 1.5
Chemical Shift (ppm)

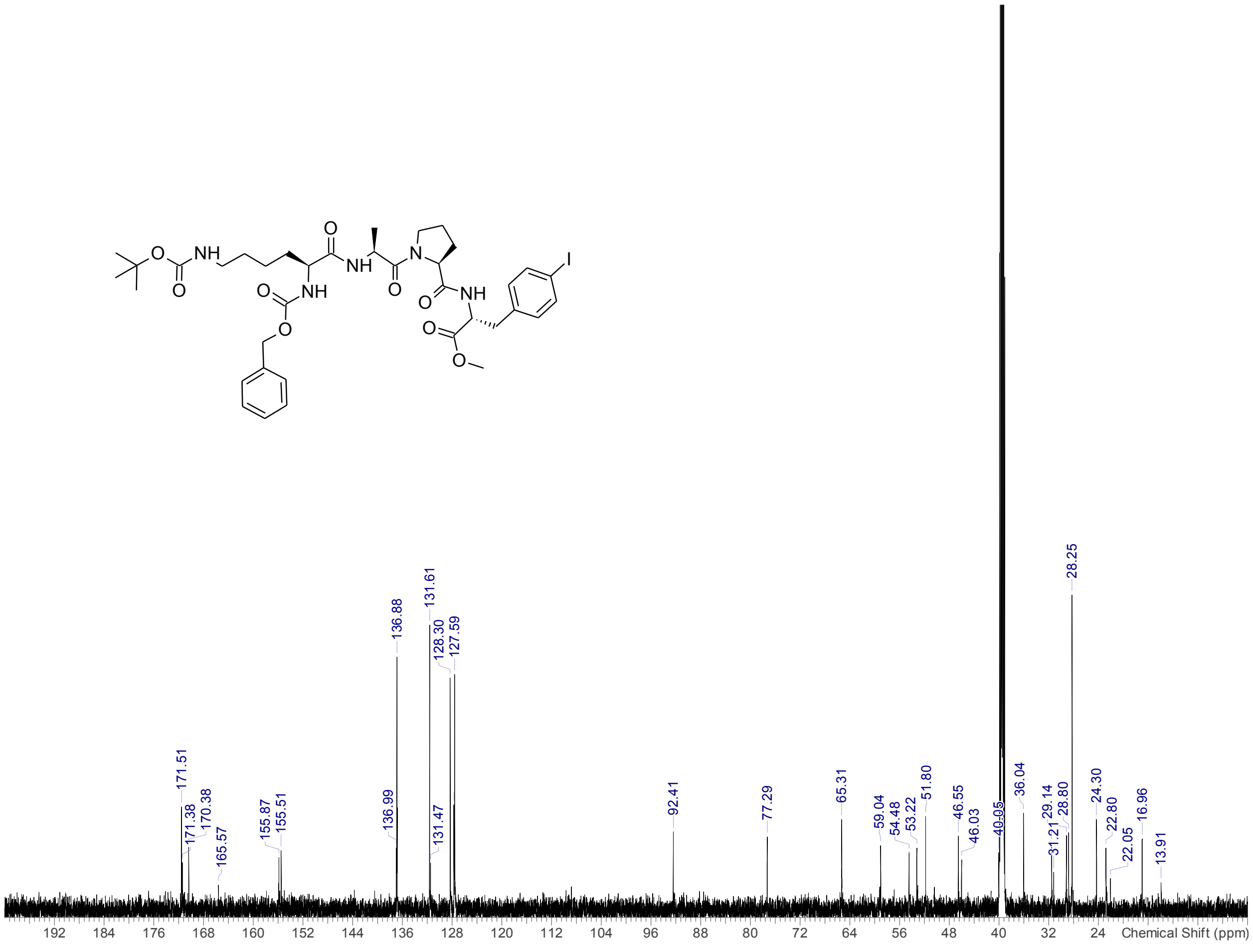
NH
O
O
NH
O
NH
N
O
O
NH
I
O
O
O
171.51 171.38 170.38 165.57 155.87 155.51 136.99 136.88 131.61 131.47 128.30 127.59 92.41 77.29 65.31 59.04 54.48 53.22 51.80 46.55 46.03 40.05 36.04 31.21 29.14 28.80 28.25 24.30 22.80 22.05 16.96 13.91
192 184 176 168 160 152 144 136 128 120 112 104 96 88 80 72 64 56 48 40 32 24
Chemical Shift (ppm)

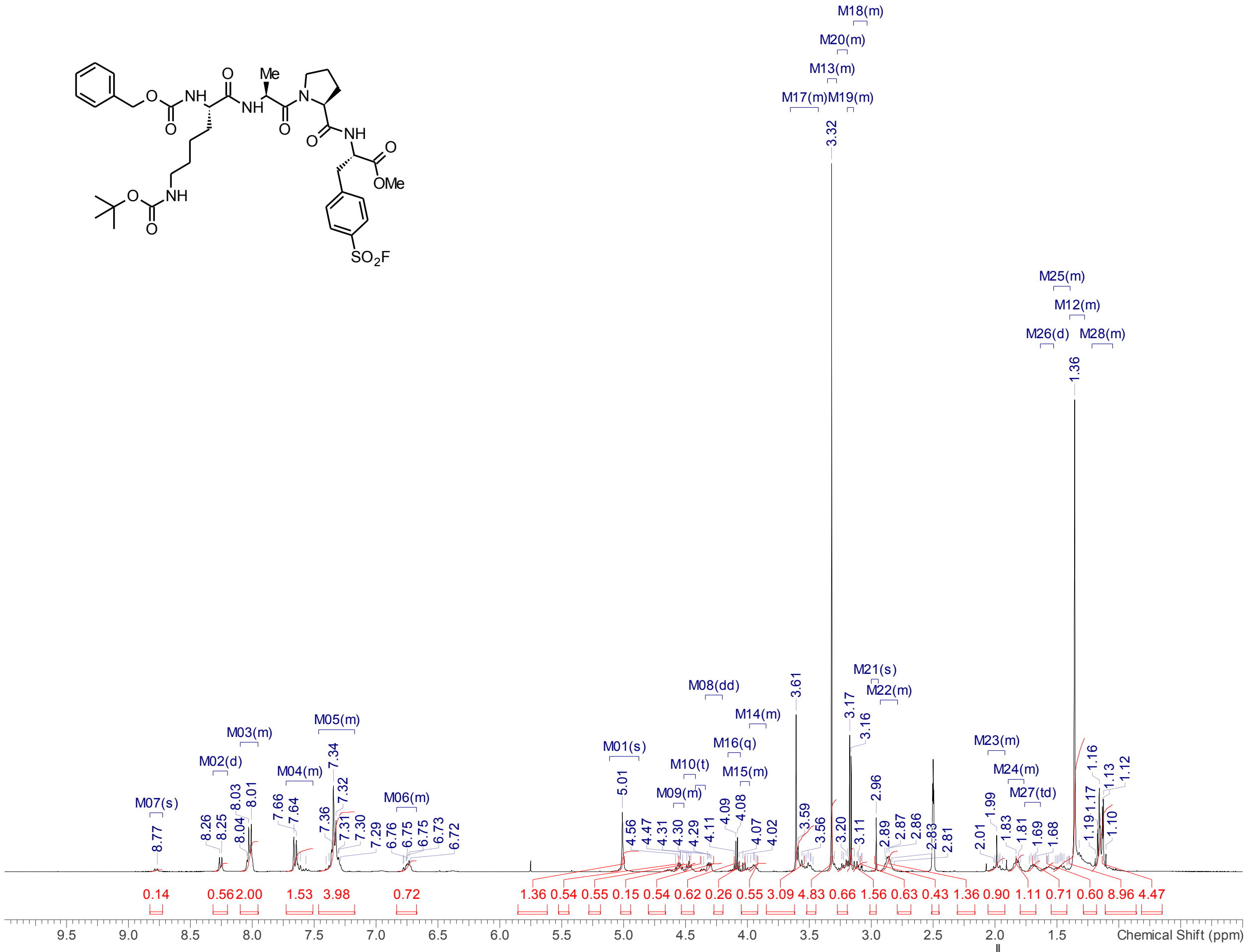

Chemical Shift (ppm)
8.77 8.26 8.25 8.04 8.03 8.01 7.66 7.64 7.36 7.34 7.32 7.31 7.30 7.29 6.76 6.75 6.75 6.73 6.72
5.01 4.56 4.47 4.31 4.30 4.29 4.11 4.09 4.08 4.07 4.02 3.61 3.59 3.56 3.32 3.20 3.17 3.16 3.11 2.96 2.89 2.87 2.86 2.83 2.81 2.01 1.99 1.83 1.81 1.69 1.68 1.36 1.19 1.17 1.16 1.13 1.12 1.10
0.14 0.56 2.00 1.53 3.98 0.72 1.36 0.54 0.55 0.15 0.54 0.62 0.26 0.55 3.09 4.83 0.66 1.56 0.63 0.43 1.36 0.90 1.11 0.71 0.60 8.96 4.47

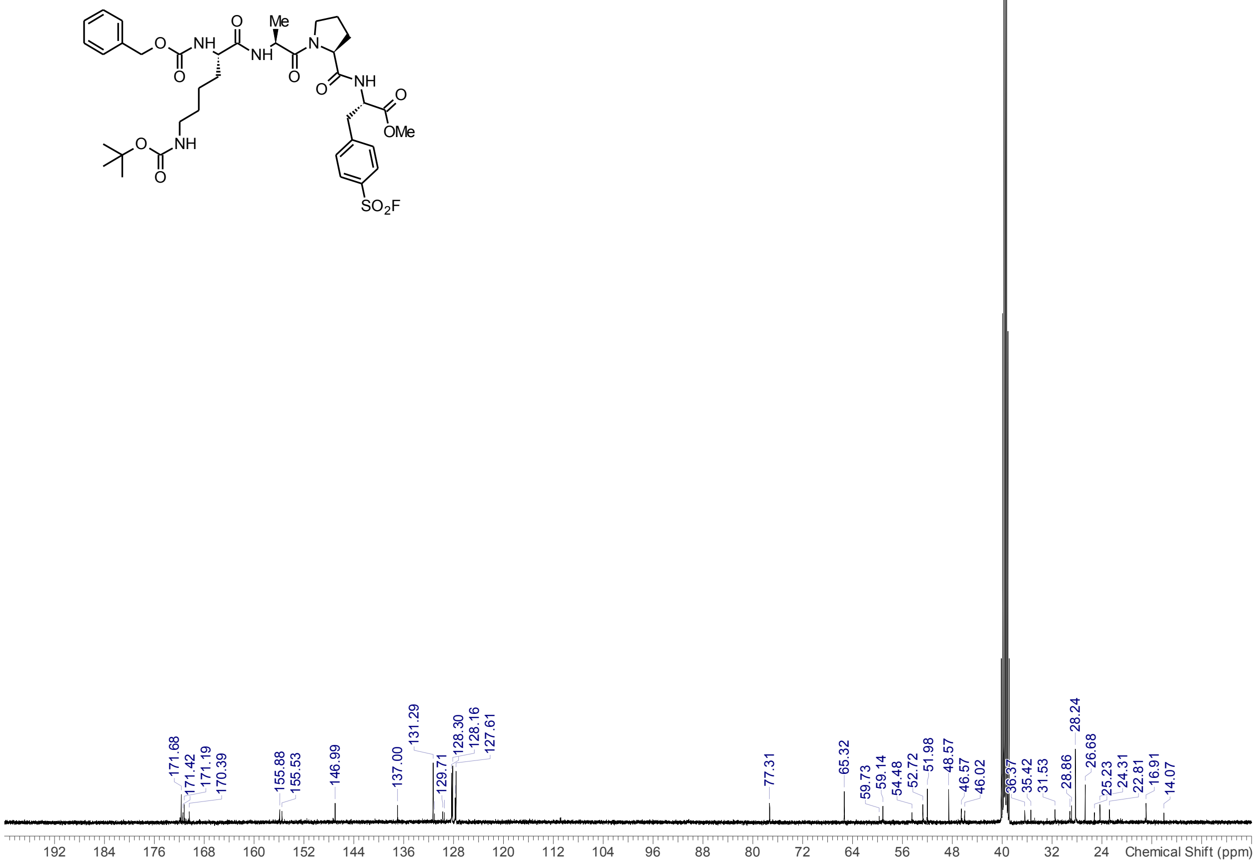

Chemical Shift (ppm)
171.68 171.42 171.19 170.39 155.88 155.53 146.99 137.00 131.29 129.71 128.30 128.16 127.61 77.31 65.32 59.73 59.14 54.48 52.72 51.98 48.57 46.57 46.02 36.37 35.42 31.53 28.86 28.24 26.68 25.23 24.31 22.81 16.91 14.07

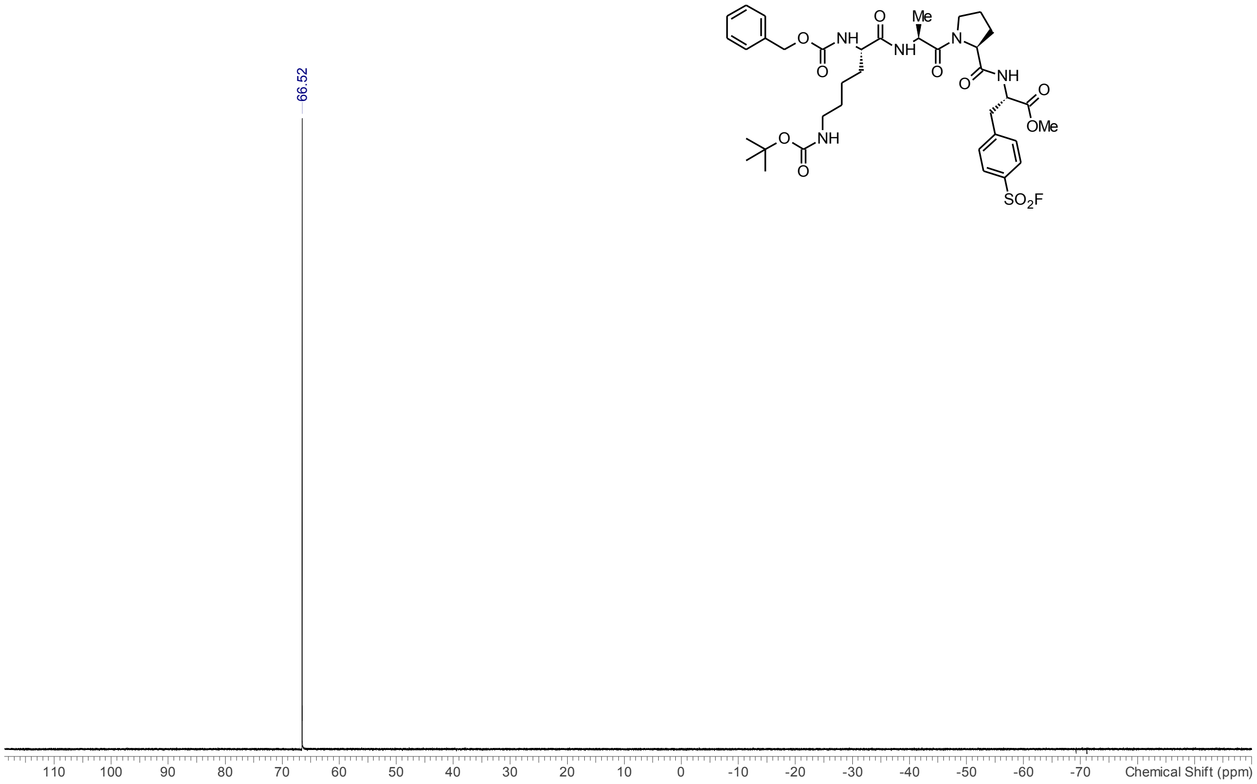
66.52
110
100
90
80
70
60
50
40
30
20
10
0
-10
-20
-30
-40
-50
-60
-70
Chemical Shift (ppm)

3.5 NMR Spectra of Sulfonyl Fluorides Synthesized from Grignard Reagents

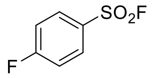

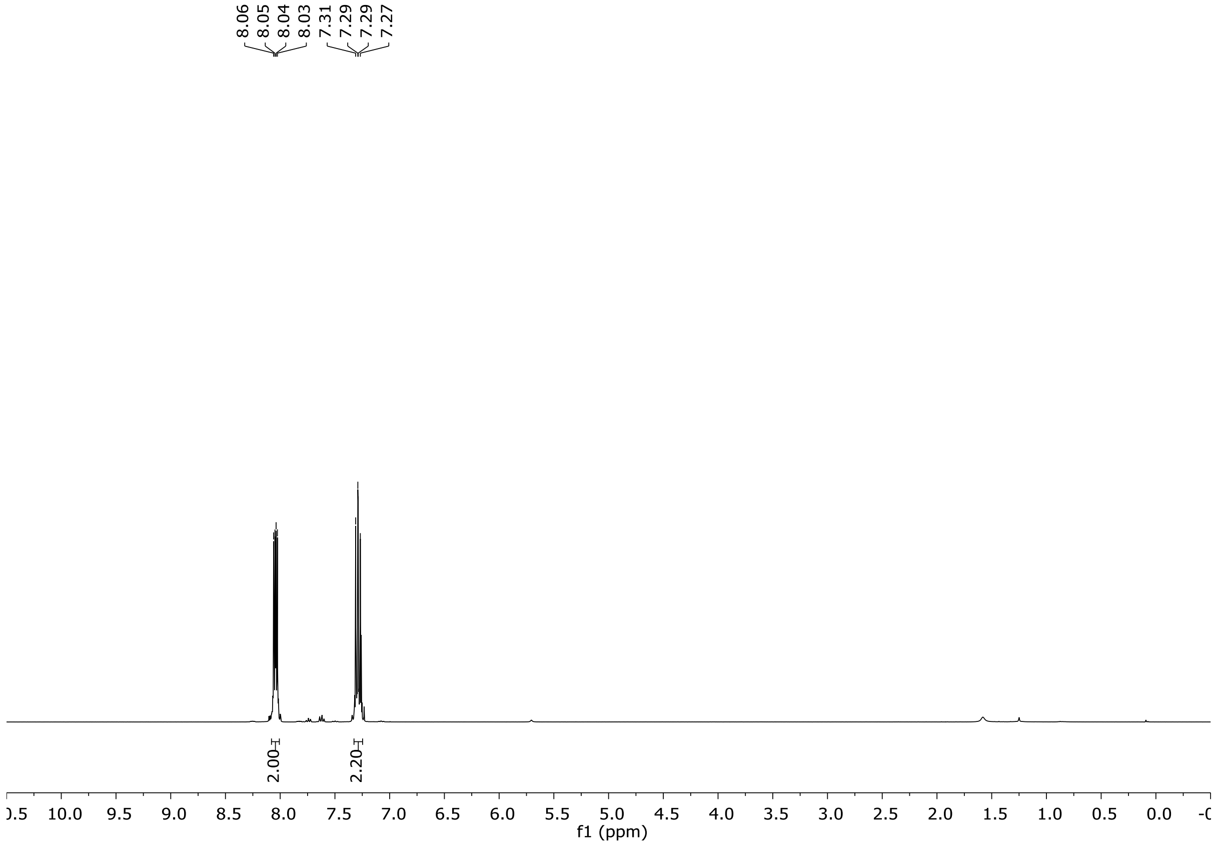

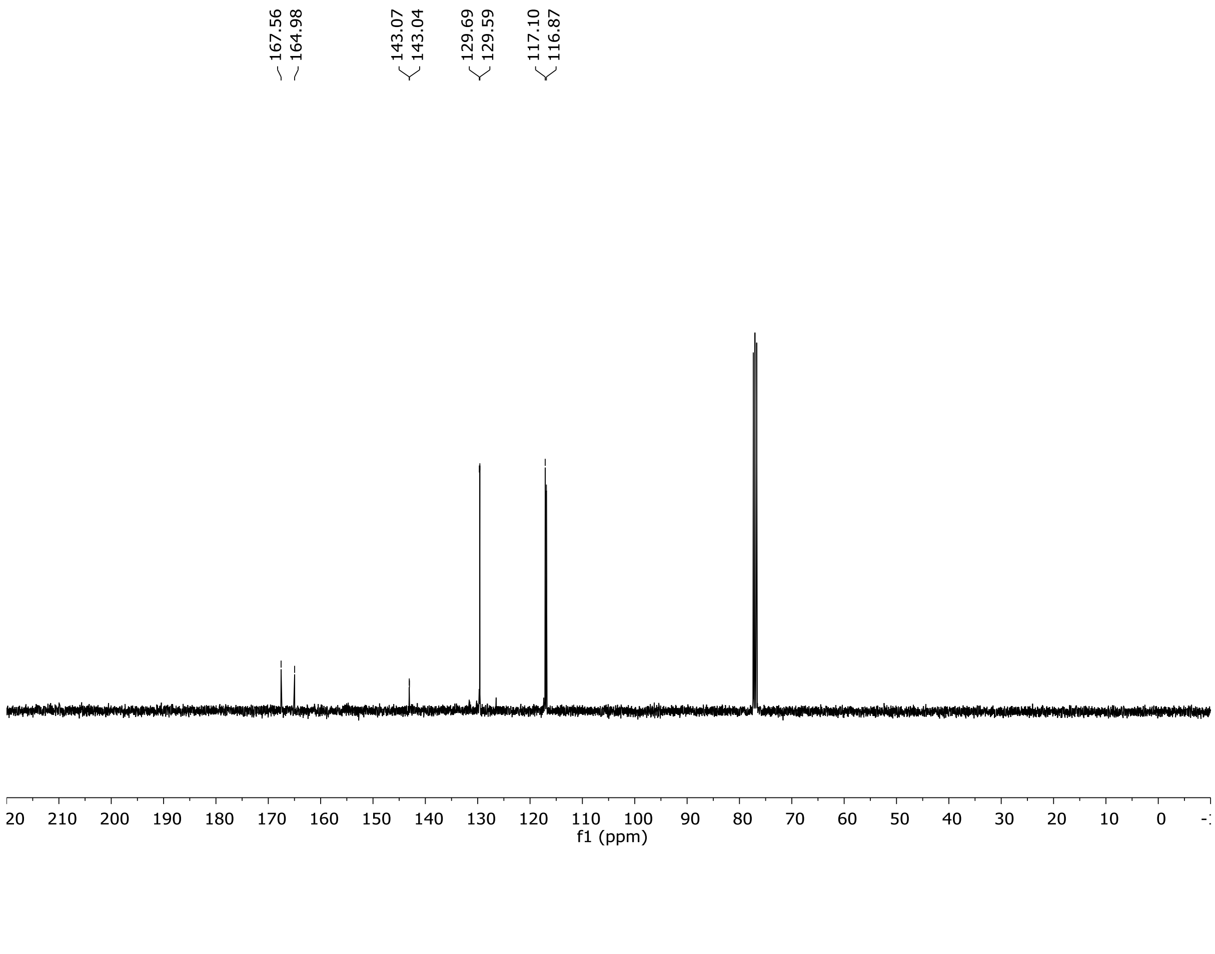

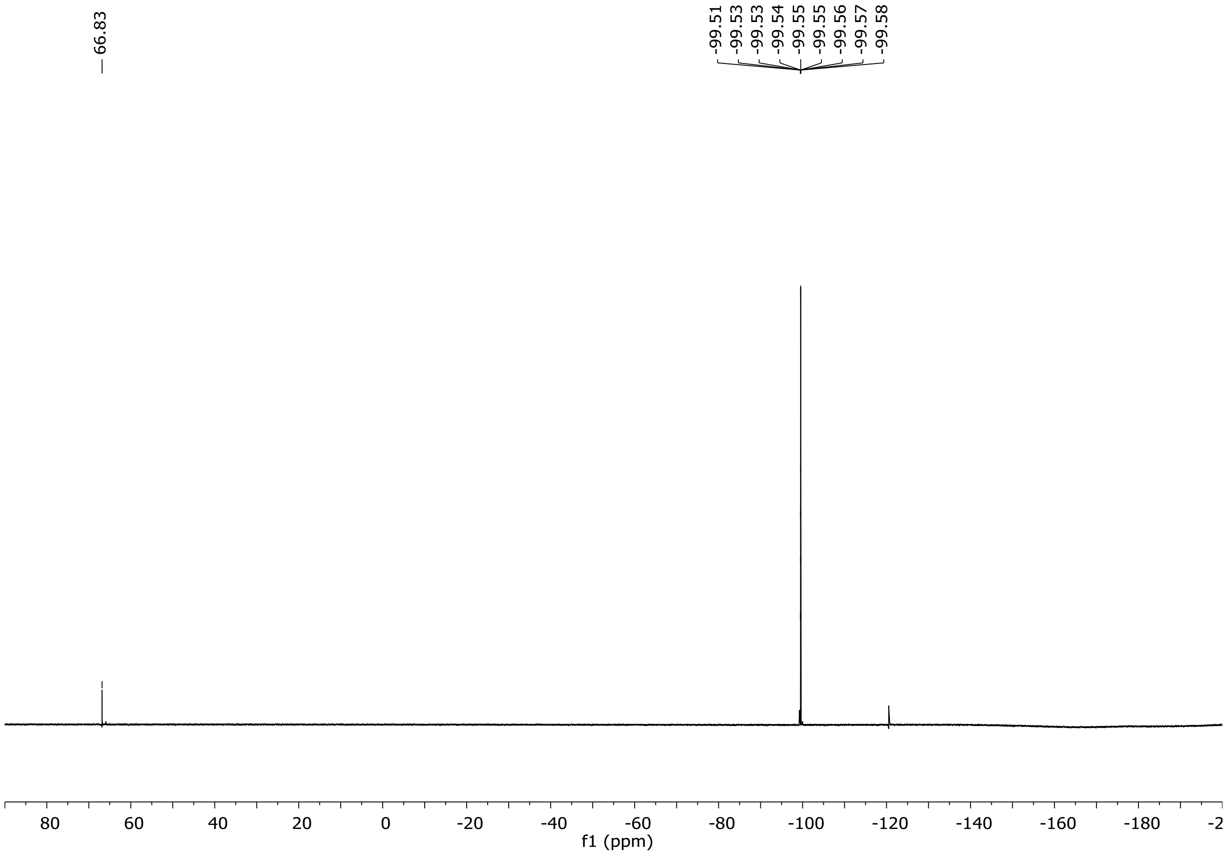

66.83
-99.51
-99.53
-99.53
-99.54
-99.55
-99.55
-99.56
-99.57
-99.58
80
60
40
20
0
-20
-40
-60
-80
-100
-120
-140
-160
-180
-2
f1 (ppm)

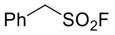
Ph
SO2F

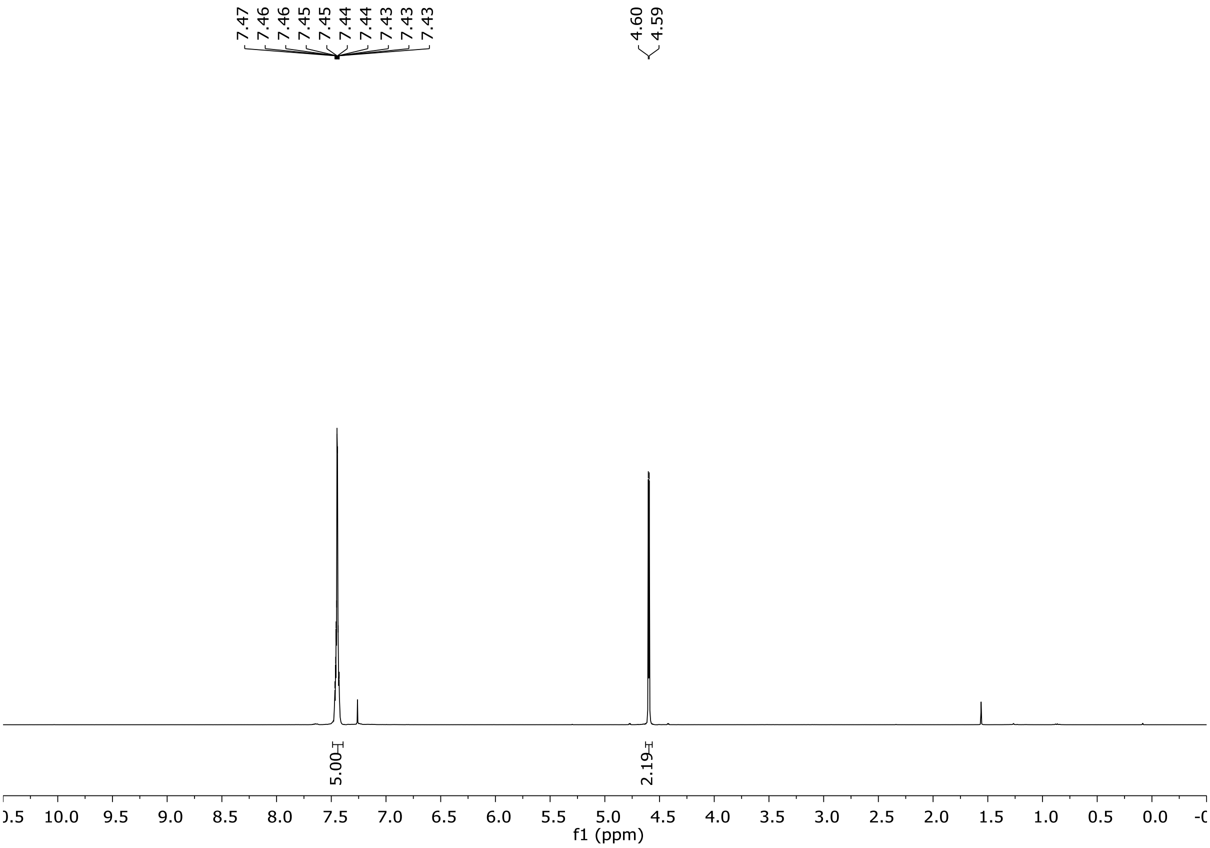
7.47
7.46
7.46
7.45
7.45
7.44
7.44
7.43
7.43
7.43
4.60
4.59
5.00
2.19
f1 (ppm)

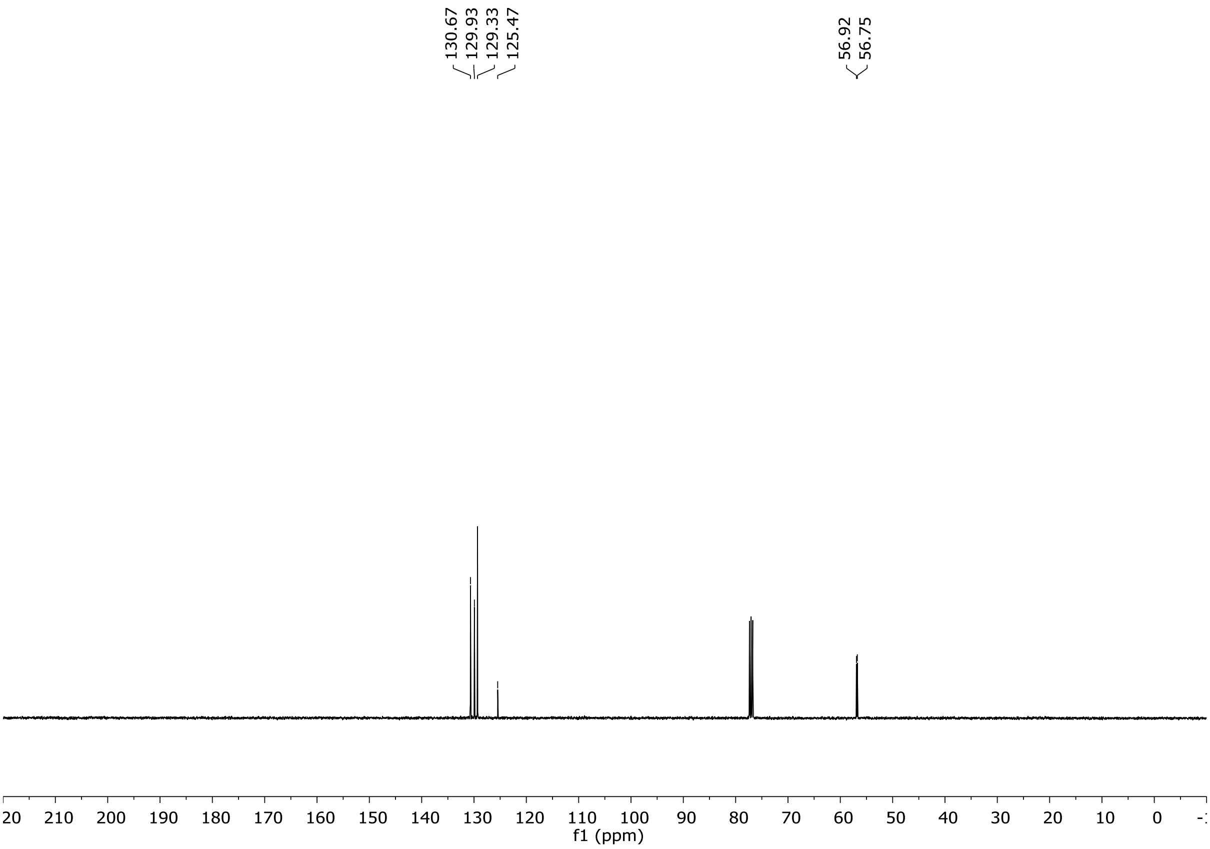
130.67
129.93
129.33
125.47
56.92
56.75
f1 (ppm)

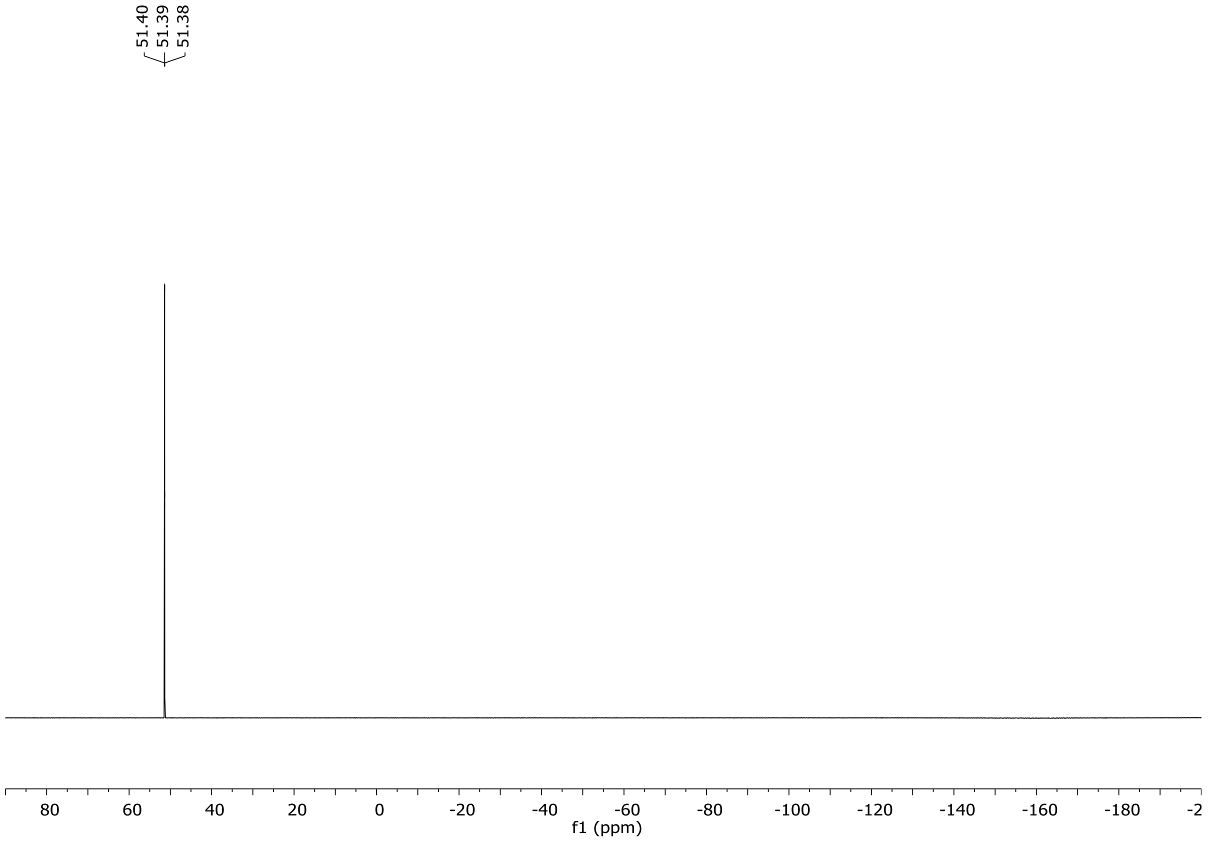
51.40
51.39
51.38
98
80 60 40 20 0 -20 -40 -60 -80 -100 -120 -140 -160 -180 -2
f1 (ppm)

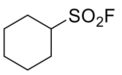
SO2F

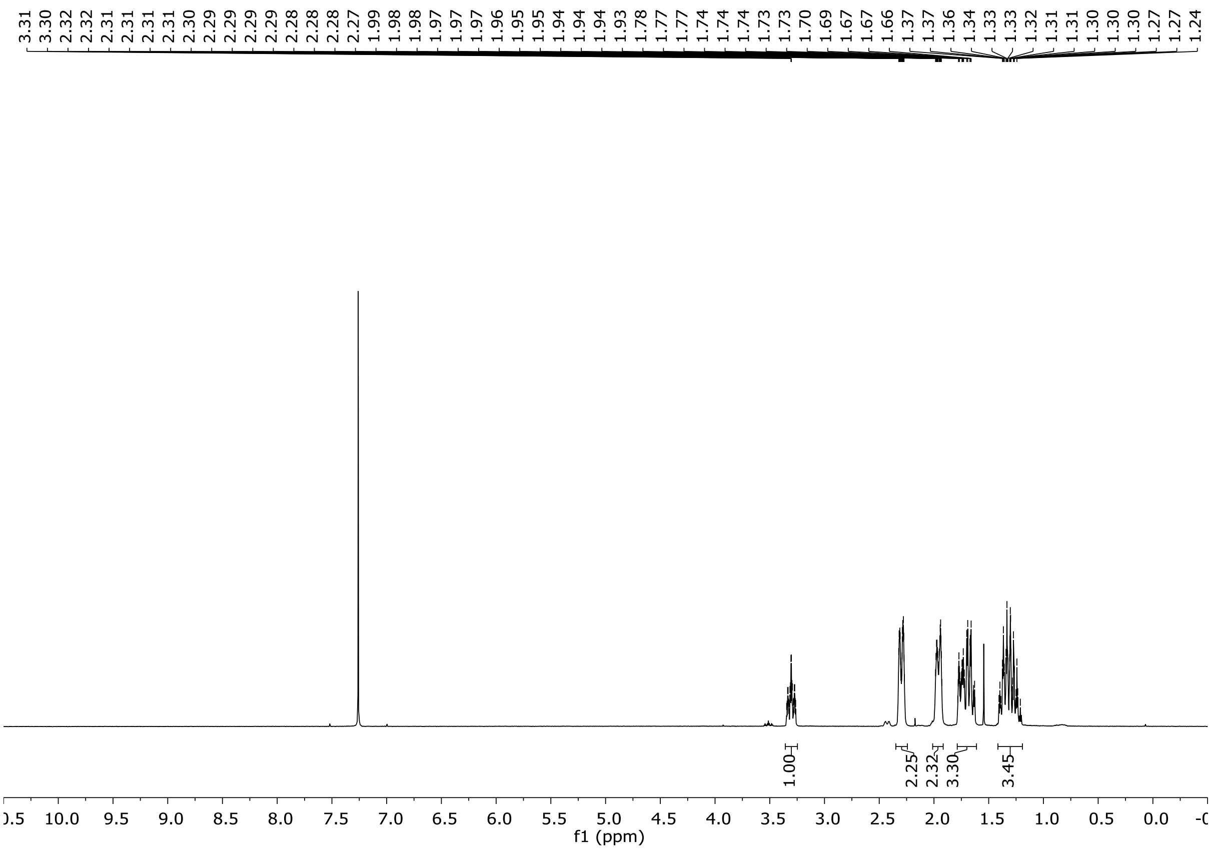
3.31 3.30 2.32 2.32 2.31 2.31 2.31 2.30 2.29 2.29 2.29 2.28 2.28 2.28 2.27 1.99 1.98 1.98 1.97 1.97 1.97 1.96 1.95 1.94 1.94 1.94 1.93 1.78 1.77 1.77 1.74 1.74 1.74 1.73 1.70 1.69 1.67 1.67 1.66 1.37 1.37 1.36 1.34 1.33 1.33 1.32 1.31 1.31 1.30 1.30 1.27 1.27 1.24
1.00
2.25
2.32
3.30
3.45
10.0 9.5 9.0 8.5 8.0 7.5 7.0 6.5 6.0 5.5 5.0 4.5 4.0 3.5 3.0 2.5 2.0 1.5 1.0 0.5 0.0
f1 (ppm)

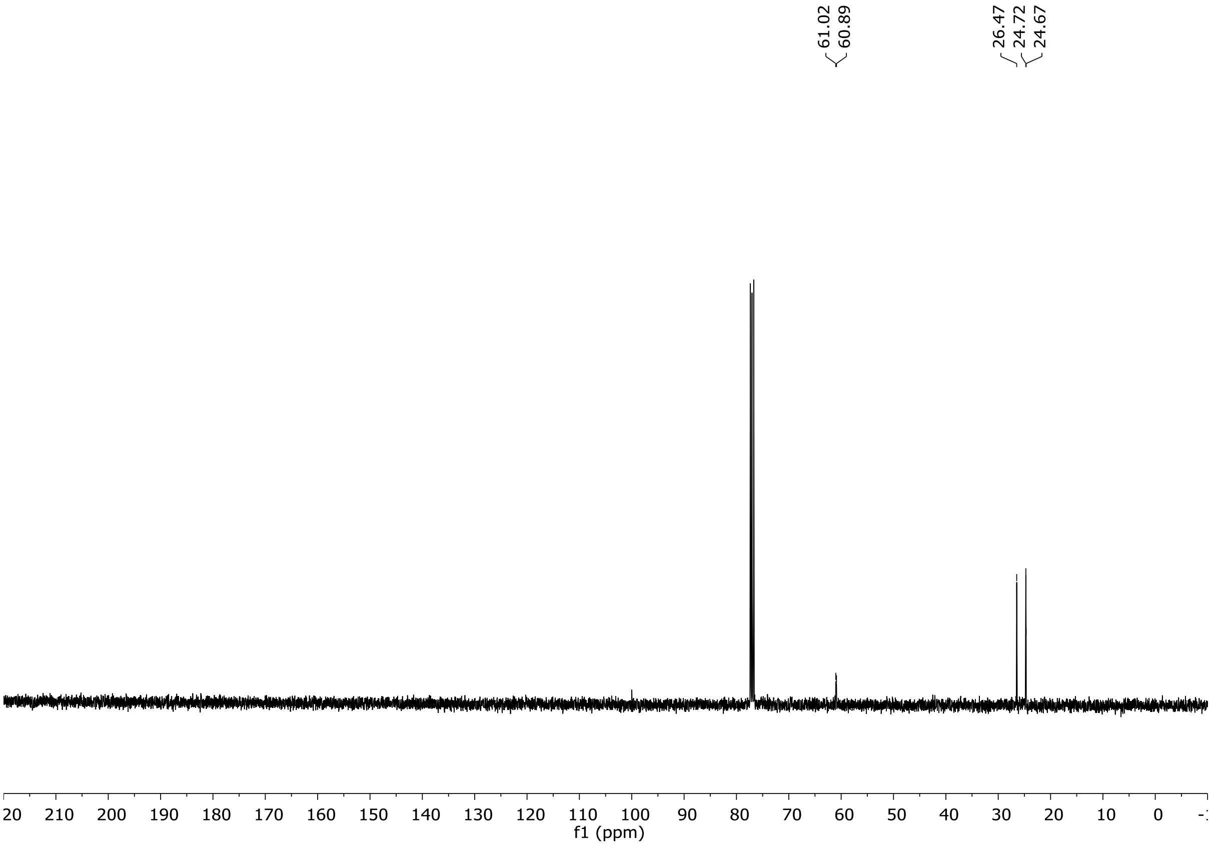
61.02
60.89
26.47
24.72
24.67
210 200 190 180 170 160 150 140 130 120 110 100 90 80 70 60 50 40 30 20 10 0
f1 (ppm)

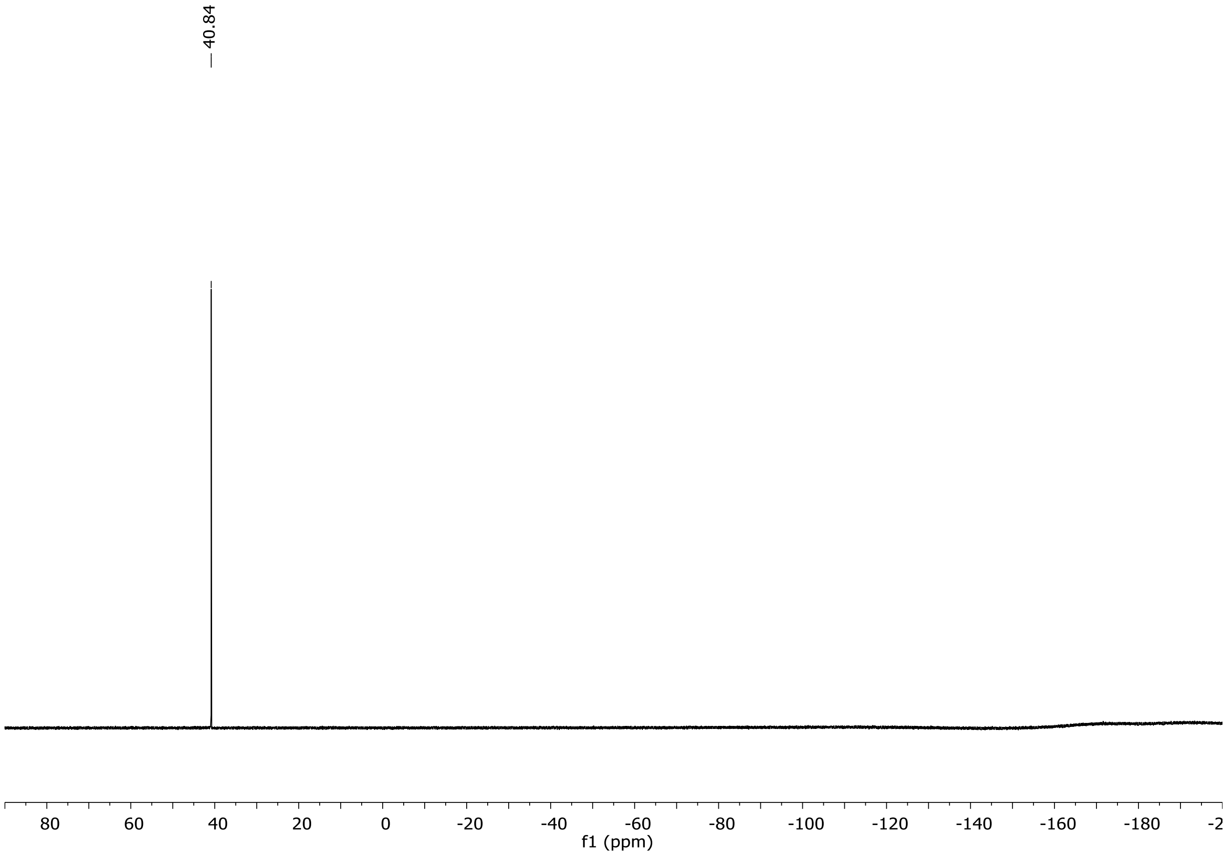
40.84
80
60
40
20
0
-20
-40
-60
-80
-100
-120
-140
-160
-180
-2
f1 (ppm)

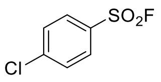
SO2F
Cl

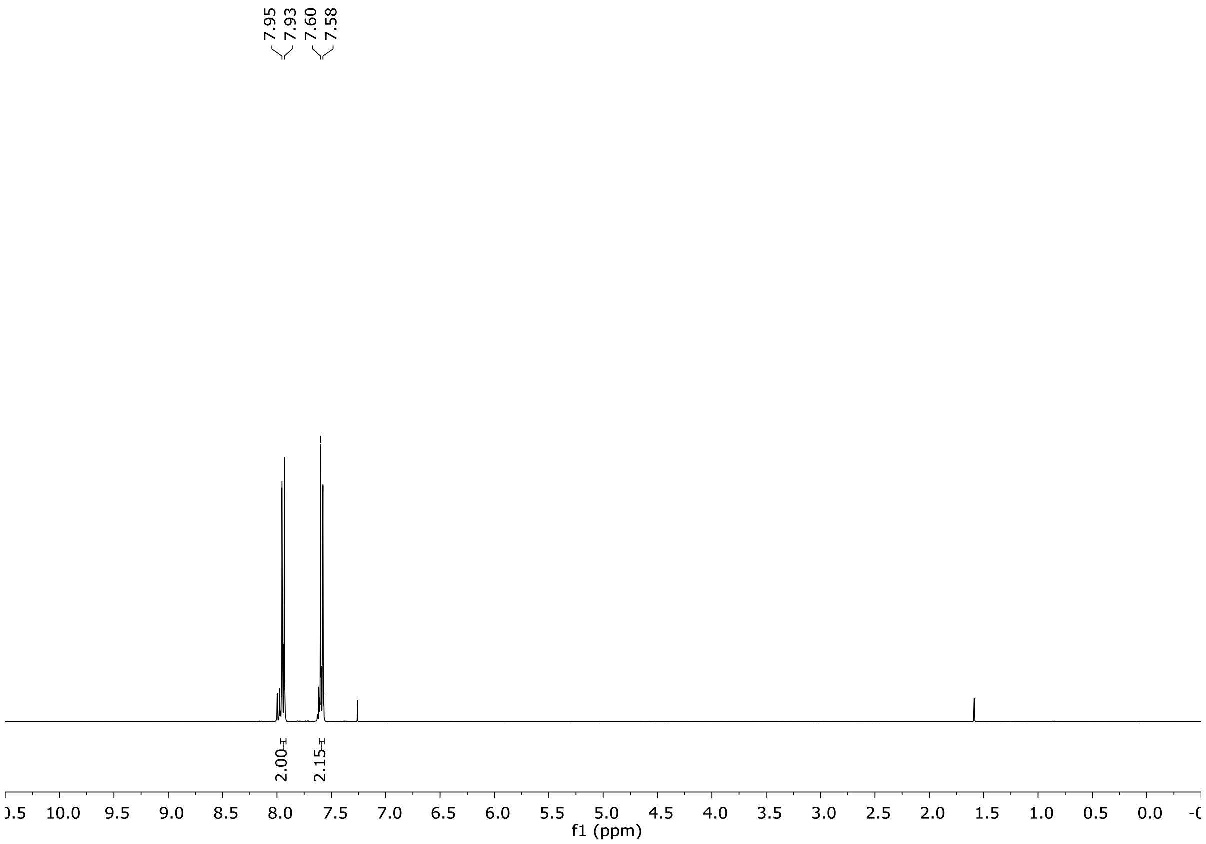
7.95
7.93
7.60
7.58
2.00
2.15
f1 (ppm)

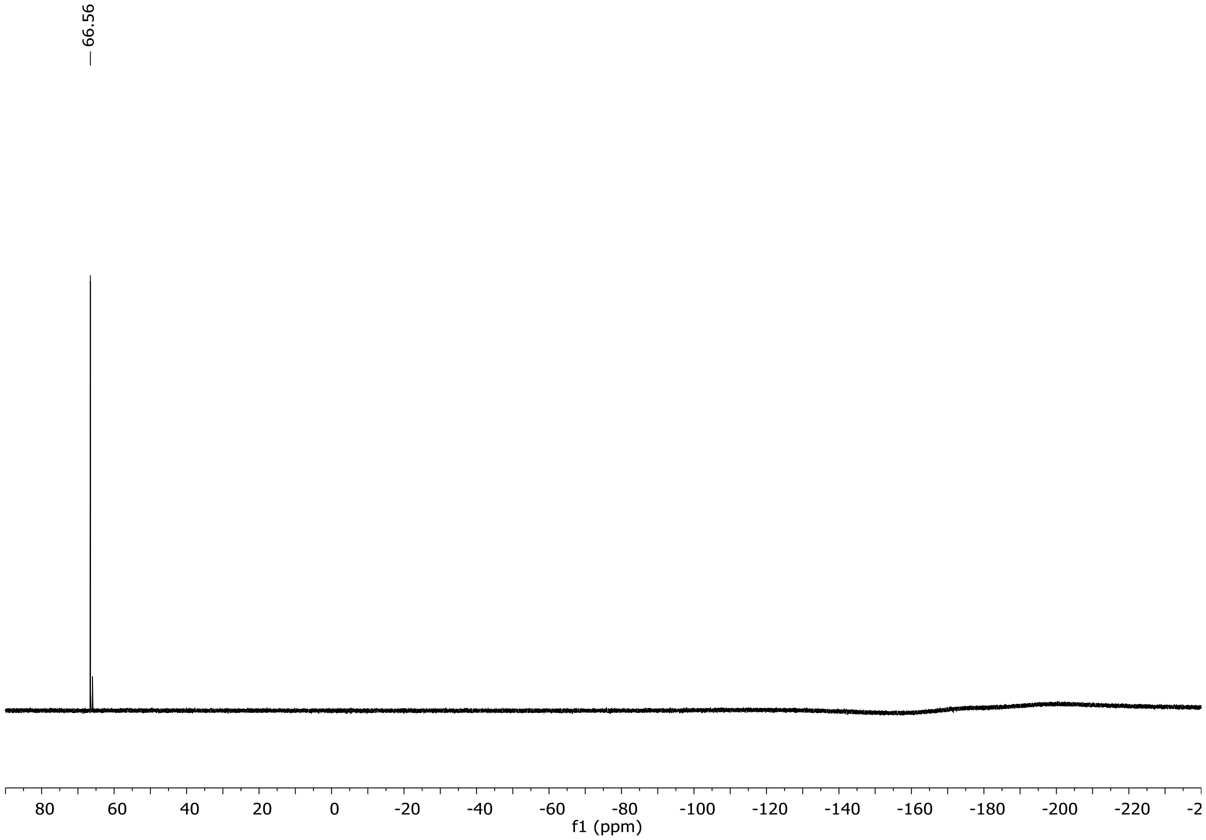
66.56
f1 (ppm)

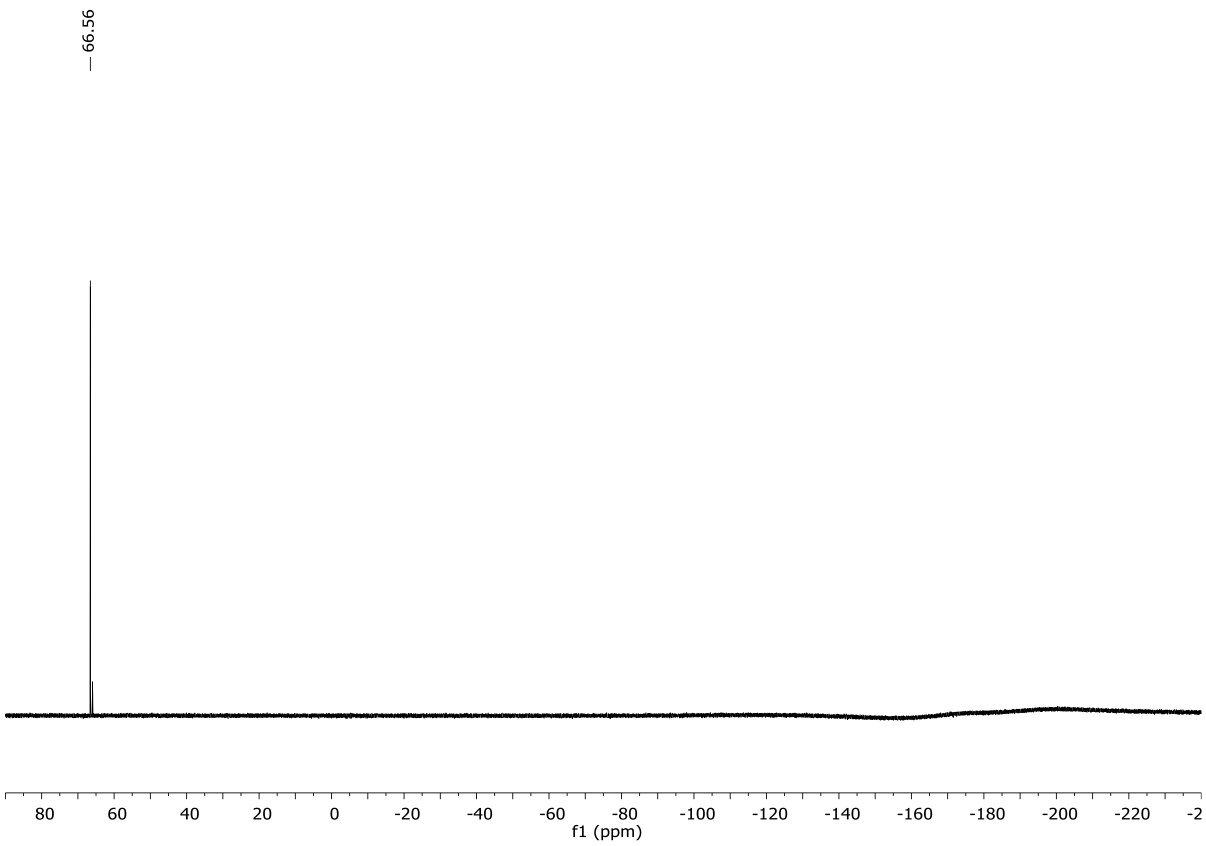
66.56
80
60
40
20
0
-20
-40
-60
-80
-100
-120
-140
-160
-180
-200
-220
-2
f1 (ppm)

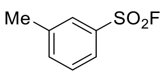
Me
SO2F

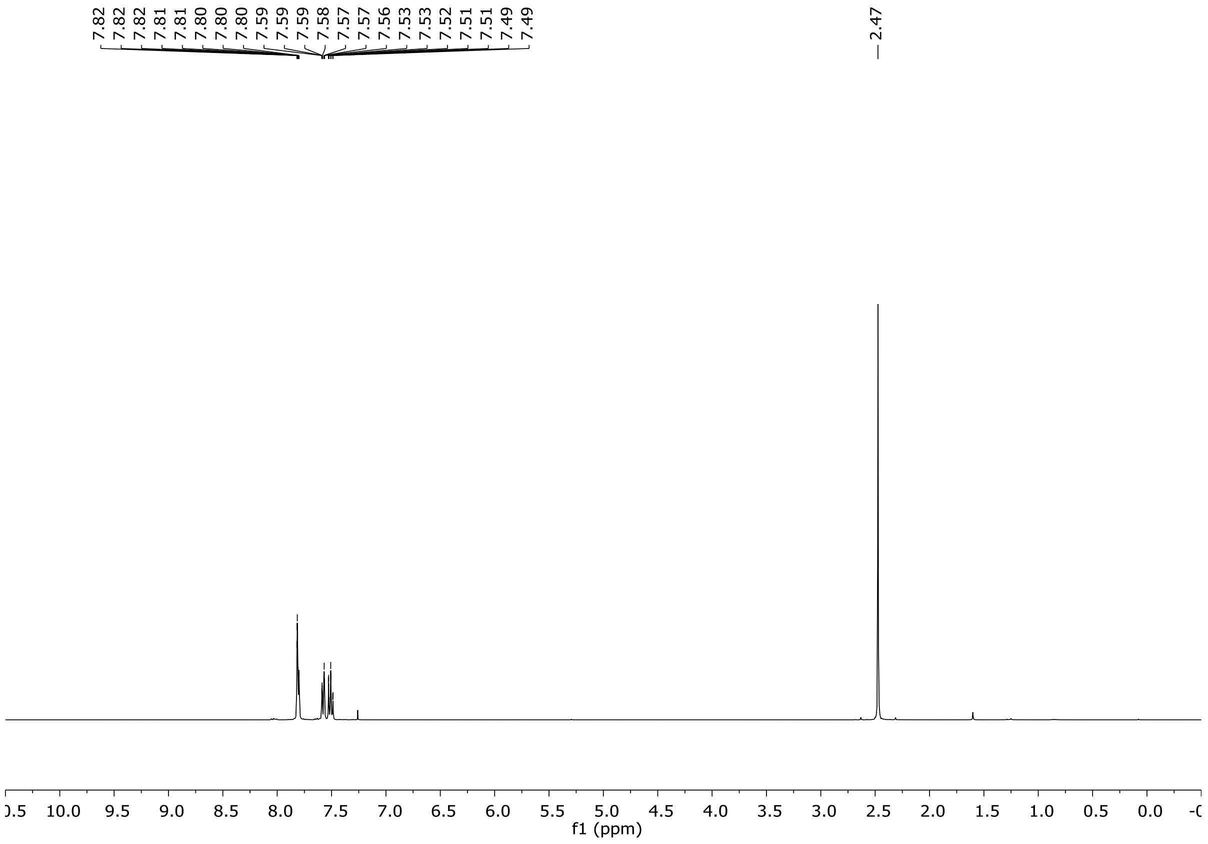
7.82 7.82 7.82 7.81 7.81 7.80 7.80 7.80 7.59 7.59 7.59 7.58 7.57 7.57 7.56 7.53 7.53 7.52 7.51 7.51 7.49 7.49
2.47
f1 (ppm)

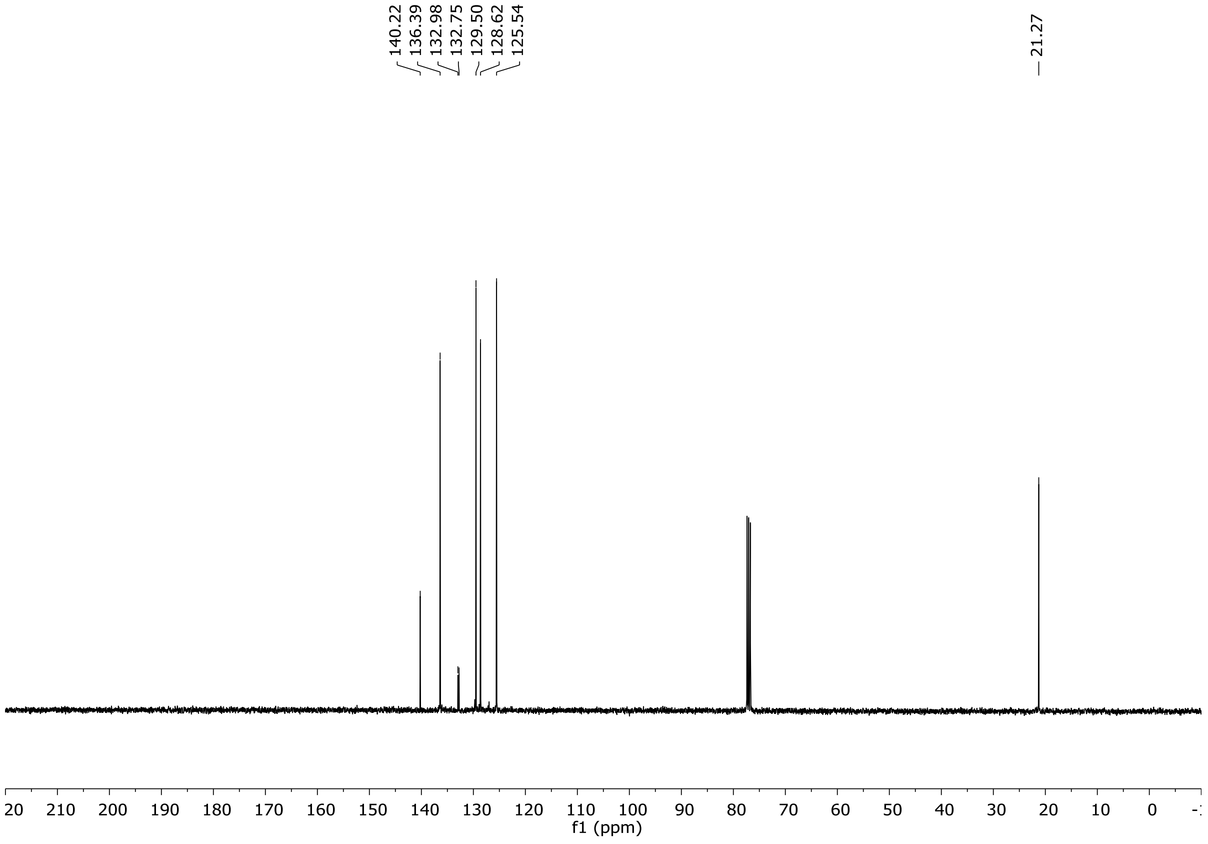
140.22 136.39 132.98 132.75 129.50 128.62 125.54
21.27
f1 (ppm)

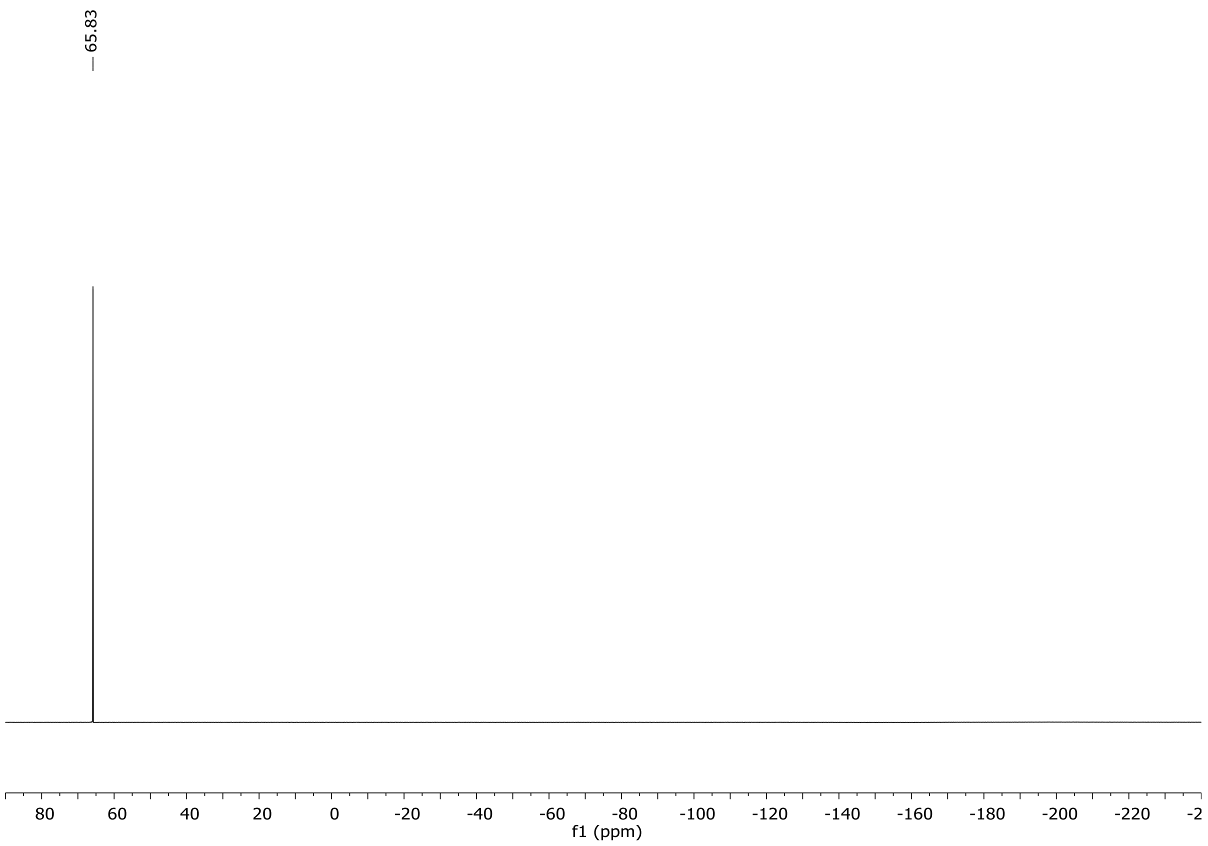
65.83
80
60
40
20
0
-20
-40
-60
-80
-100
-120
-140
-160
-180
-200
-220
-2
f1 (ppm)

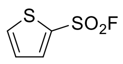
S
SO2F

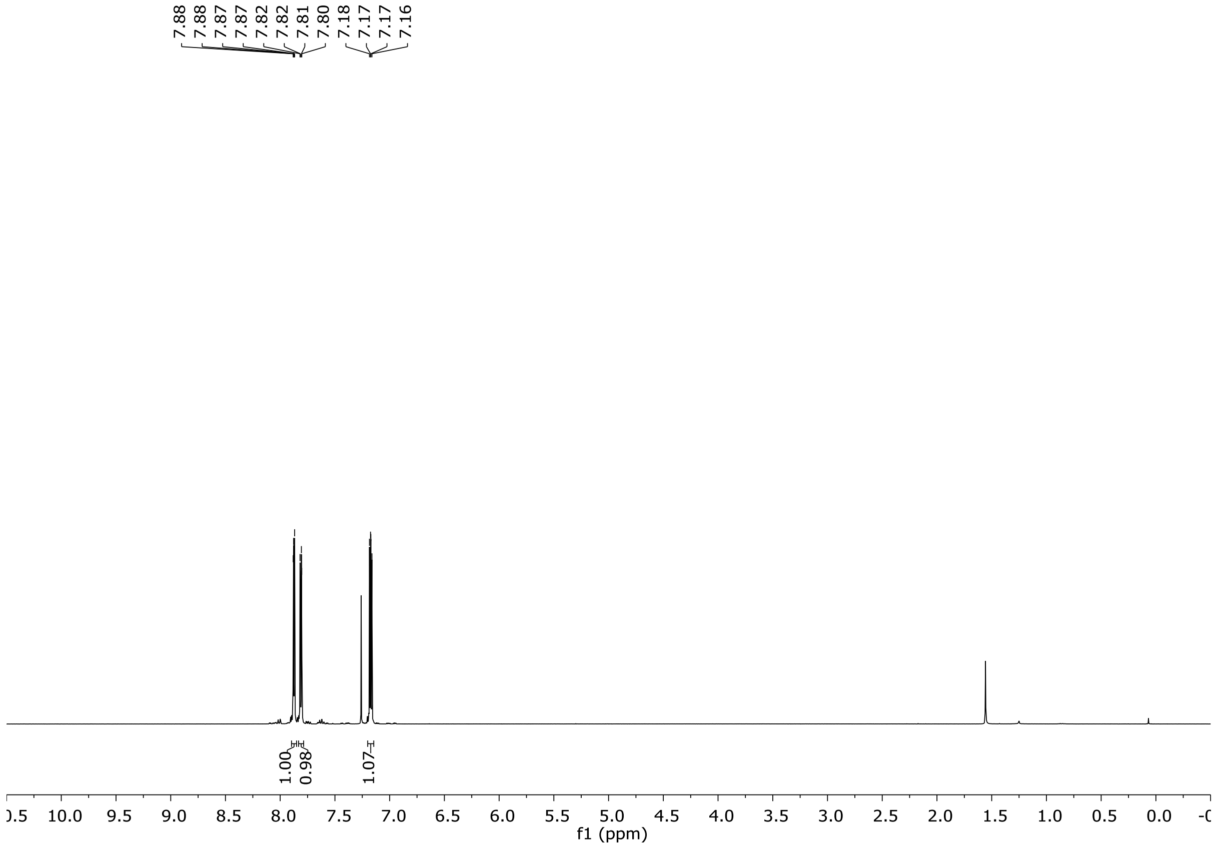
7.88
7.88
7.87
7.87
7.82
7.82
7.81
7.80
7.18
7.17
7.17
7.16
1.00
0.98
1.07
f1 (ppm)

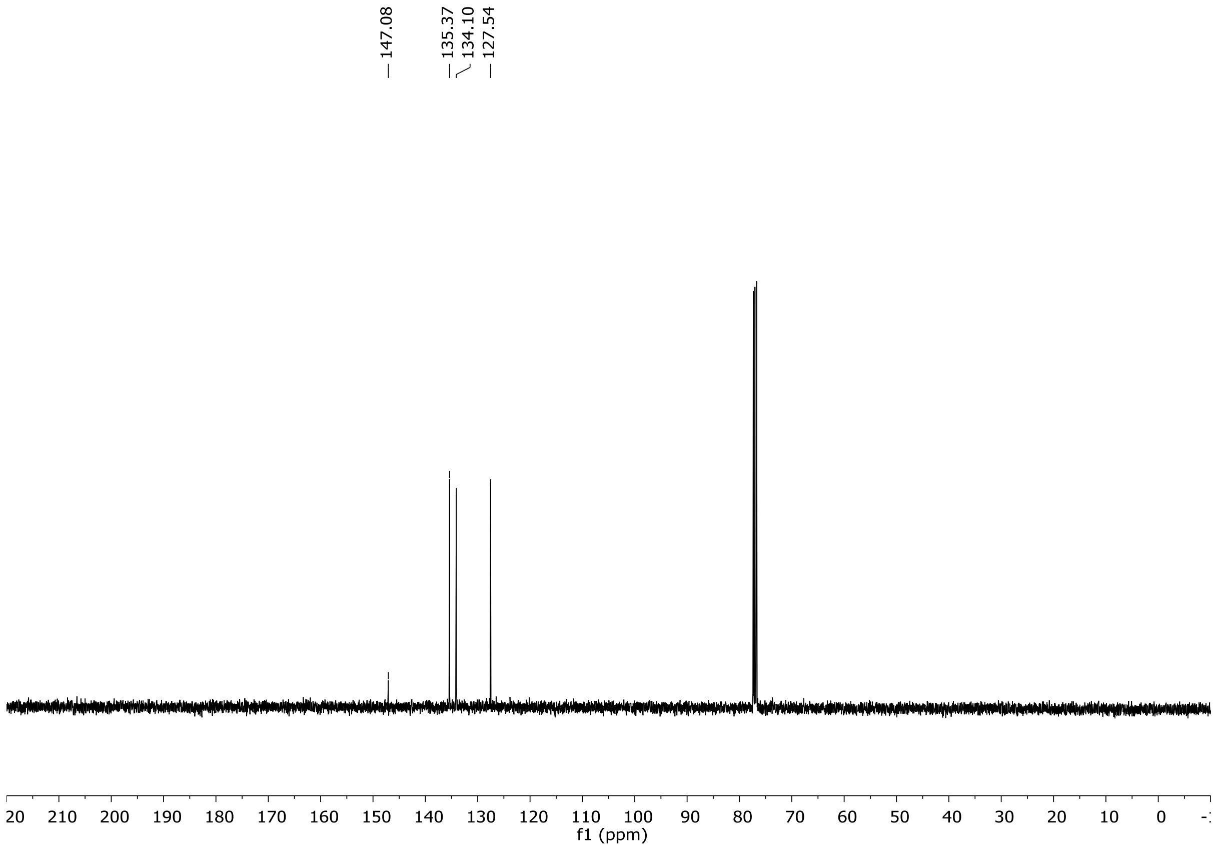
147.08
135.37
134.10
127.54
f1 (ppm)

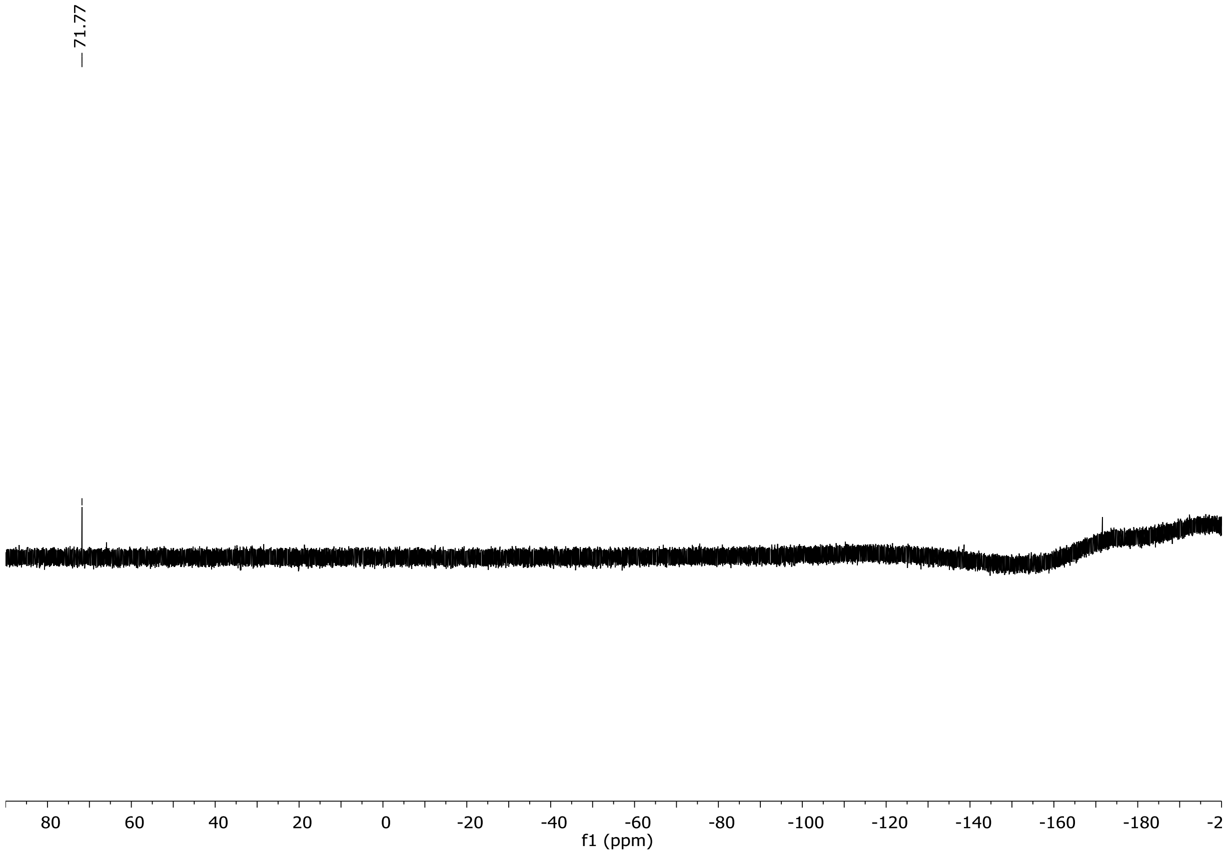

71.77
80
60
40
20
0
-20
-40
-60
-80
-100
-120
-140
-160
-180
-2
f1 (ppm)
